# FLT3-ITD Activates RSK1 to Enhance Proliferation and Survival of AML Cells by Activating mTORC1 and eIF4B Cooperatively with PIM or PI3K and by Inhibiting Bad and BIM

**DOI:** 10.3390/cancers11121827

**Published:** 2019-11-20

**Authors:** Daisuke Watanabe, Ayako Nogami, Keigo Okada, Hiroki Akiyama, Yoshihiro Umezawa, Osamu Miura

**Affiliations:** 1 Department of Hematology, Graduate School of Medical and Dental Sciences, Tokyo Medical and Dental University, Tokyo 113-8519, Japan; dwathema@tmd.ac.jp (D.W.); nogahema@tmd.ac.jp (A.N.); keighema@tmd.ac.jp (K.O.); hakihema@tmd.ac.jp (H.A.); umehema@tmd.ac.jp (Y.U.); 2 Department of Clinical Laboratory, Medical Hospital, Tokyo Medical and Dental University, Tokyo 113-8519, Japan

**Keywords:** RSK, FLT3-ITD, acute myeloid leukemia, PIM, mTOR, eIF4B, BIM, Bad

## Abstract

FLT3-ITD is the most frequent tyrosine kinase mutation in acute myeloid leukemia (AML) associated with poor prognosis. We previously found that FLT3-ITD activates the mTORC1/S6K/4EBP1 pathway cooperatively through the STAT5/PIM and PI3K/AKT pathways to promote proliferation and survival by enhancing the eIF4F complex formation required for cap-dependent translation. Here, we show that, in contrast to BCR/ABL causing Ph-positive leukemias, FLT3-ITD distinctively activates the serine/threonine kinases RSK1/2 through activation of the MEK/ERK pathway and PDK1 to transduce signals required for FLT3-ITD-dependent, but not BCR/ABL-dependent, proliferation and survival of various cells, including MV4-11. Activation of the MEK/ERK pathway by FLT3-ITD and its negative feedback regulation by RSK were mediated by Gab2/SHP2 interaction. RSK1 phosphorylated S6RP on S235/S236, TSC2 on S1798, and eIF4B on S422 and, in cooperation with PIM, on S406, thus activating the mTORC1/S6K/4EBP1 pathway and eIF4B cooperatively with PIM. RSK1 also phosphorylated Bad on S75 and downregulated BIM-EL in cooperation with ERK. Furthermore, inhibition of RSK1 increased sensitivities to BH3 mimetics inhibiting Mcl-1 or Bcl-2 and induced activation of Bax, leading to apoptosis, as well as inhibition of proliferation synergistically with inhibition of PIM or PI3K. Thus, RSK1 represents a promising target, particularly in combination with PIM or PI3K, as well as anti-apoptotic Bcl-2 family members, for novel therapeutic strategies against therapy-resistant FLT3-ITD-positive AML.

## 1. Introduction

FLT3 is a receptor-tyrosine kinase expressed on hematopoietic progenitor cells, playing important roles in regulation of proliferation, survival, and differentiation of these cells [1,2]. Internal tandem duplication mutations in the FLT3 juxtamembrane domain (FLT3-ITDs) are the most frequent mutations in acute myeloid leukemia (AML), found in 25–30% of cases and associated with a poor prognosis. Point mutations within the FLT3 tyrosine kinase domain (FLT3-TKDs), such as the most prevalent D835Y mutation, are found in 5–10% of patients with AML, whereas their significance on prognosis remains unclear. FLT3-ITD, as well as FLT3-TKD, induces ligand-independent autophosphorylation and activation of the receptor, leading to constitutive activation of various downstream signaling events involving the PI3K/AKT/mTOR and MEK/ERK pathways, as well as STAT5, to promote cytokine-independent cell survival and proliferation [1,2]. The other aberrantly activated tyrosine kinase mutants BCR/ABL and JAK2-V617F also activate these intracellular signaling events constitutively and are involved in leukemogenesis of Ph-positive leukemias, such as chronic myeloid leukemia and Ph-positive acute lymphoblastic leukemia, or Ph-negative myeloproliferative neoplasms, such as polycythemia vera or primary myelofibrosis, respectively [3,4]. Various tyrosine kinase inhibitors (TKIs) for BCR/ABL, such as imatinib, nilotinib, and dasatinib, have been in clinical use and have demonstrated unprecedented efficacy for treatment for Ph-positive leukemias, whereas the JAK1/JAK2 inhibitor ruxolitinib has been approved for clinical use against myeloproliferative neoplasms with only limited efficacies. Although several TKIs relatively specific for FLT3 are currently under development or in clinical use, clinical trials with these used as a single agent have so far shown only limited efficacies with transient responses because of emergences of resistance mutations, and through other various mechanisms in the case of quizartinib (AC-220) [1]. Therefore, novel strategies to target mutant FLT3 are needed to improve the clinical outcome of AML with FLT3 mutations.

The serine/threonine kinase mTOR forming two multiprotein complexes, mTORC1 and mTORC2, is activated by FLT3-ITD mainly through the PI3K/AKT pathway [5,6,7]. AKT phosphorylates TSC2 on five residues (S939, S981, S1130, S1132, and T1462), which is required for inactivation of this negative regulator and for full stimulation of mTORC1 [6]. While mTORC2 is involved in activation of AKT through its phosphorylation on S473, mTORC1 plays a critical role in regulation of cap-dependent translation by phosphorylating 4EBP1 to release it from the mRNA m^7^-GTP cap-binding protein eIF4E, which interacts with the scaffolding protein eIF4G to initiate the formation of the translation-initiating complex eIF4F [7]. This factor is required for the translation of mRNAs containing long 5′-UTRs, which are highly structured and have a high G + C content, such as those for c-Myc, Mcl-1, and cyclin D1. In addition, mTORC1 activates S6K, which is reported to phosphorylate eIF4B to enhance cap-dependent translation by the eIF4F complex, as well as phosphorylating S6RP to enhance translation by promoting the recruitment of the 40S ribosome to the mRNA [7,8]. Mcl-1 is a highly unstable antiapoptotic Bcl-2 family member playing a crucial role in survival of hematopoietic progenitor cells and various malignant hematopoietic cells, including AML cells by inhibiting activation of the intrinsic mitochondria-mediated apoptosis pathway triggered by initiator proapoptotic Bcl-2 members, including Bad and BIM, and executed by effectors, including Bax and Bak [9,10]. The initiator BH3-only proteins, such as Bad or the most abundant BIM isoform, extra-long BIM (BIM-EL), are post-translationally modified through phosphorylation, which regulates their function or expression levels. BH3 mimetics antagonizing antiapoptotic Bcl-2 family members, such as Bcl-2, Bcl-xL, and Mcl-1, are currently in clinical use or under development for treatment for hematological malignancies, including AML [11]. FLT3-ITD directly phosphorylates and, thus, activates STAT5 strongly, which contributes to enhance transforming potentials of FLT3-ITD, at least partly, by inducing expression of PIM serine/threonine kinases [1,2]. We previously reported that FLT3-ITD activates the mTORC1/S6K/4EBP1 pathway cooperatively through the STAT5/PIM and PI3K/AKT pathways in AML cells, which promotes proliferation and survival, as well as therapy resistance to PI3K/AKT inhibitors and proteasome inhibitors, at least partly, by upregulating cap-dependent translation by the eIF4F complex [12,13,14].

The p90 ribosomal S6 kinase (RSK) family serine/threonine protein kinases, including the predominant isoforms expressed in AML RSK1 and RSK2, possess the N- and C-terminal kinase domains (NTKD and CTKD) and are activated downstream of the MEK/ERK signaling pathway [15,16]. ERK activates CTKD by phosphorylating T573 (RSK1) or T577 (RSK2) in the activation loop, thus leading to autophosphorylation of S380 (RSK1) or S386 (RSK2) within the hydrophobic motif in the linker region between NTKD and CTKD, which serves as a docking site for PDK1 required for activation of NTKD through phosphorylation of S221 (RSK1) or S227 (RSK2) in the activation loop. ERK also phosphorylates T359/S363 (RSK1) or T365/S369 (RSK2) within the turn motif in the linker region to facilitate the recruitment of PDK1 to enhance NTKD activation. On the other hand, NTKD autophosphorylates the C-terminal region of RSK required for binding or ERK, thus resulting in downregulation of RSK activation by ERK. When activated, NTKD is reported to phosphorylate a variety of downstream substrates, such as S6RP, TSC2, GSK3α/ß, eIF4B, IkBα, and c-Fos, to promote cell growth and survival through various mechanisms [15,16]. Previously, RSK2 has been reported to be activated or upregulated through PIM in FLT3-ITD-positive AML cells, and suggested to be required for leukemogenesis by FLT3-ITD but not by BCR/ABL [17,18]. However, these studies analyzed activation of only CTKD but not NTKD, examined effects of mainly the CTKD inhibitor FMK, which we show in the present study inhibits FLT3-ITD as an off-target effect, and did not fully examine the downstream signaling mechanisms mediated by RSK in FLT3-ITD-positive leukemic cells.

Here we show that FLT3-ITD activates NTKDs of RSK1 and RSK2 distinctively in contrast to BCR/ABL, and that RSK1 predominantly mediates signaling required for proliferation and survival of FLT3-ITD-dependent, but not BCR/ABL-dependent, leukemic cells, including primary AML cells from one patient we could examine. Mechanistically, FLT3-ITD activated RSK through activation of not only the MEK/ERK signaling pathway, but also PDK1, while RSK1 phosphorylated substrates, including TSC2, S6RP, eIF4B, GSK3α/ß, Gab2, and Bad, on specific sites and decreased BIM-EL expression, thus enhancing the mTORC1/S6K/4EBP1/eIF4 signaling pathway cooperatively with PIM or PI3K and preventing apoptosis through the intrinsic mitochondrial pathway. Thus, the present study suggests that RSK1 may represent a promising molecular target, along with PIM or PI3K, for novel therapeutic strategies against therapy-resistant FLT3-ITD-positive AML with a poor prognosis.

## 2. Results

### 2.1. RSKs Play a Crucial Role in Proliferation of Leukemic Cells Dependent on FLT3-ITD But Not BCR/ABL or JAK2-V617F

#### 2.1.1. RSK Inhibitors FMK and LJI308 Inhibit Proliferation of Leukemic Cell Lines Dependent on FLT3-ITD But Not BCR/ABL or JAK2-V617F

**Figure 1:**
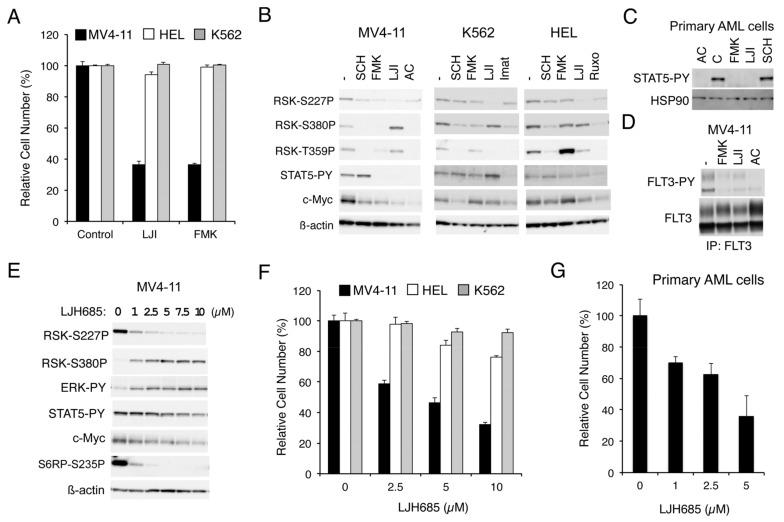
Inhibition of RSKs remarkably inhibits proliferation of leukemic cells dependent on FLT3-ITD but not BCR/ABL or JAK2-V617F. (**A**) MV4-11, HEL, or K562 cells were cultured for 48 h in the absence (Control) or presence of 20 µM LJI308 (LJI) or 5 µM FMK, as indicated. Viable cell numbers were measured by the colorimetric assay. Each column represents the mean of triplicate cultures, with error bars indicating standard errors, and is expressed as a percentage to the control cell numbers. (**B**) MV4-11, K562, or HEL cells were left untreated as control (−) or treated for 6 h with 0.5 µM SCH772984, 5 µM FMK, 20 µM LJI308, 10 nM quizartinib (AC), 1 µM imatinib (Imat), or 1 µM ruxolitinib (Ruxo), as indicated. Cells were then lysed and subjected to immunoblot analysis with antibodies against indicated proteins. ß-actin was used for a loading control. Abbreviations: RSK-S227P, phospho-S227-RSK2; RSK-S380P, phospho-S380-RSK1; RSK-T359P, phospho-T359/S363-RSK1; STAT5-PY, phospho-Y694-STAT5. (**C**) Primary acute myeloid leukemia (AML) cells expressing FLT3-ITD were left untreated as control (C) or treated for 6 h with 3 nM quizartinib, 5 µM FMK, 20 µM LJI308, or 0.5 µM SCH772984, as indicated, and analyzed. HSP90 was used for a loading control. (**D**) MV4-11 cells were left untreated as control (−) or treated for 6 h with 5 µM FMK, 20 µM LJI308, or 1 nM quizartinib, as indicated, and lysed. Cell lysates were subjected to immunoprecipitation (IP) with ant-FLT3 antibody, followed by immunoblot analysis. FLT3-PY: Phospho-Y591-FLT3. (**E**) MV4-11 cells were treated for 6 h with indicated concentrations of LJH685 and subjected to immunoblot analysis. Abbreviations: ERK-PY, phospho-T202/Y204-Erk; S6RP-S235P, phospho-S235/S236-S6RP. (**F**) MV4-11, HEL, or K562 cells were cultured for 48 h with indicated concentrations of LJH685 and subjected to the colorimetric assay. (**G**) Primary FLT3-ITD-positive AML cells were cultured for 48 h with indicated concentrations of LJH685, and viable cell numbers were counted after trypan blue staining. Each column represents the mean of triplicate cultures, with error bars indicating standard errors.

To explore the roles RSKs play in leukemic cells harboring the aberrant tyrosine kinase FLT3-ITD, BCR/ABL, or JAK2-V617F, we first examined the effect of RSK inhibitors on proliferation of representative cell lines expressing these leukemogenic kinases. As shown in Figure 1A, LJI308 or FMK, which inhibits RSK NTKD or CTKD, respectively, prominently reduced proliferation of MV4-11 expressing FLT3-ITD but not HEL, or K562 expressing JAK2-V617F or BCR/ABL, respectively. Other cell lines expressing BCR/ABL, KU812, and MOLM-1 were also relatively resistant to FMK as compared with MV4-11, while proliferation of these cells was efficiently inhibited by the BCR/ABL inhibitor imatinib (Figure S1A). It was also confirmed that other JAK2-V617F-positive cell lines, PVTL-1 and PVTL-2, were also relatively refractory to FMK similar to HEL, whereas these cells responded variably to the JAK1/2 inhibitor ruxolitinib (Figure S1B). These results suggest that RSK may play a critical role in proliferation of leukemic cells transformed by FLT3-ITD, but not in those transformed by BCR/ABL or JAK2-V617F.

We next examined the effects of these inhibitors on RSKs and intracellular signaling events in these cells by Western blot analysis. As expected, SCH772984, which specifically inhibits ERK [19], and FMK reduced the CTKD-dependent phosphorylation of RSK1 on S380 or RSK2 on S386 in these cells, as examined by anti-phospho-S380-RSK1, although the effect of FMK in HEL was observed only modestly (Figure 1B). On the other hand, activation-specific phosphorylation of NTKD in the activation loop of RSK1 on S221 or RSK2 on S227, as examined by anti-phospho-S227-RSK2, was reduced by these inhibitors distinctly in MV4-11 cells, but only modestly in HEL or K562. In contrast, LJI308 inhibited the activation-specific phosphorylation of NTKD in all of these cells, as expected, whereas it activated CTKD in MV4-11 but not in HEL or K562. Interestingly, these inhibitors prominently reduced c-Myc expression only in MV4-11, except that the ERK inhibitor SCH772984 reduced c-Myc in K562. Furthermore, although inhibition of FLT3-ITD or JAK2-V617F by quizartinib or ruxolitinib, respectively, in relevant cells not only inhibited RSK CTKD, but also downregulated NTKD, inhibition of BCR/ABL by imatinib in K562 inhibited only CTKD but not NTKD (Figure 1B). 

#### 2.1.2. FMK or LJI308 Inhibits FLT3-ITD through an Off-Target Effect

Unexpectedly, FMK or LJI308 inhibited activation-specific phosphorylation of STAT5 in MV4-11, but not in HEL or K562 (Figure 1B). Furthermore, these RSK inhibitors inhibited STAT5 phosphorylation also in primary AML cells with FLT3-ITD, from one patient we could examine, in a similar manner with the FLT3 inhibitor quizartinib (Figure 1C and Figure S1C). Because STAT5 is phosphorylated and activated, at least in part, directly by FLT3-ITD, as well as BCR/ABL or JAK2-V617F to play important roles in regulation of proliferation and survival of leukemic cells, we next examined whether FMK or LJI308 at a concentration required to inhibit RSK NTKD may also inhibit FLT3-ITD in MV4-11 cells. As shown in Figure 1D, FMK and, to a lesser extent, LJI308 inhibited the activation specific phosphorylation of FLT3-ITD on Y591 in MV4-11 cells, thus confirming their off-target effect on FLT3-ITD, which should make it complicated to interpret the effects of these inhibitors on FLT3-ITD-expressing cells in the present, as well as previously published studies [17,18]. 

#### 2.1.3. The Specific RSK Inhibitor LJH685 or Knockout of RSK in MV4-11 Inhibits Proliferation of MV4-11 and a Model Cell Line Transformed by FLT3-ITD

**Figure 2:**
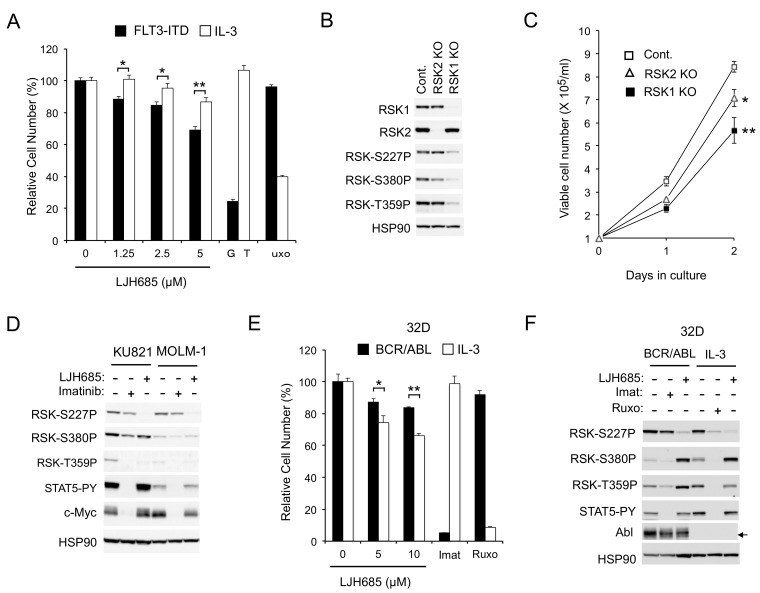
RSKs play a crucial role in proliferation of cells transformed by FLT3-ITD but not BCR/ABL. (**A**) 32D/FLT3-ITD cells inducibly expressing FLT3-ITD (FLT3-ITD) and cultured without IL3 or cultured with IL3 (IL3) without expressing FLT3-ITD were cultured for 48 h with indicated concentrations of LJH685, 50 nM gilteritinib (GRT), or 2 µM ruxolitinib (Ruxo), as indicated, and subjected to the colorimetric assay. Each column represents the mean of triplicate cultures, with error bars indicating standard errors, and is expressed as a percentage to the control cell numbers. The asterisks indicate statistically significant differences from control cells determined by Student’s *t*-test (* *p* < 0.05, ** *p* < 0.01). (**B**) MV4-11 cells knocked out (KO) of RSK1 or RSK2, as well as vector control cells (Cont.), as indicated, were subjected to immunoblot analysis. Abbreviations: RSK-S227P, phospho-S227-RSK2; RSK-S380P, phospho-S380-RSK1; RSK-T359P, phospho-T359/S363-RSK1. (**C**) MV4-11 cells knocked out (KO) of RSK1 or RSK2, as well as vector control cells (Cont.), as indicated, were cultured for indicated days, and viable cell numbers were counted and plotted. Each data point represents the mean of triplicate determinations, with error bars indicating standard errors. * *p* < 0.05, ** *p* < 0.005. (**D**) KU821 or MOLM-1 cells were treated for 6 h with 2 µM imatinib or 5 µM LJH685, as indicated, and analyzed. STAT5-PY: Phospho-Y694-STAT5. (**E**) 32D cells expressing BCR/ABL (BCR/ABL) and cultured without IL-3 or parental 32D cells cultured with IL-3 (IL-3) were cultured for 48 h with indicated concentrations of LJH685, 1 µM imatinib (Imat), or 1 mM ruxolitinib (Ruxo), as indicated, and analyzed. * *p* = 0.054, ** *p* < 0.0005. (**F**) 32D cells described in (**E**) were treated for 6 h with 5 µM LJH685, 3 µM imatinib, or 3 µM ruxolitinib, as indicated, and analyzed.

Thus, we next examined the effects of LJH685, reported to be the most specific and potent pan-RSK inhibitor [20,21], on MV4-11 cells. As shown in Figure 1E, LJH685 inhibited RSK NTKD at concentrations as low as 1 µM, with its inhibitory effect reaching the maximal level at 5 µM without distinctly inhibiting activation of STAT5. Interestingly, activation of RSK CTKD by LJH685 was observed reciprocally with inhibition of RSK NTKD and accompanied by activation of ERK. Nevertheless, it was confirmed that phosphorylation of the well-established RSK target site S235/S236 in S6RP was inhibited by LJH685 at as low as 1 µM. It was also confirmed that LJH685 prominently reduced proliferation of MV4-11, as well as expression of c-Myc, at concentrations barely affecting activation of STAT5 (Figure 1E,F). Furthermore, viable cell numbers of primary FLT3-ITD-positive AML cells from one patient we could examine were also dose-dependently decreased when cultured with LJH685 (Figure 1G). On the other hand, JAK2-V617F-dependent proliferation of HEL was reduced only modestly by LJH685 (Figure 1F). Consistent with this, LJH685 inhibited proliferation of a model hematopoietic 32D cell line, 32D/ITD, more significantly when proliferating dependently on FLT3-ITD than on IL-3 (Figure 2A), which mainly mediates proliferation signals through JAK2 coupled with the IL-3 receptor. 

To rule out the possibility that the RSK inhibitor LJH685 might have inhibited proliferation through off-target effects, and to evaluate the significance of RSK1 and RSK2 separately, we examined the effects of knockout (KO) of RSK1 or RSK2 on proliferation of MV4-11 cells. As shown in Figure 2B, the activation-specific phosphorylation of RSK NTKD and CTKD, as well as phosphorylation of the ERK target sites, was remarkably reduced in RSK1 KO cells, but only modestly in RSK2 KO cells, which suggests that RSK1 may be the isoform predominantly activated in MV4-11 cells. Consistent with this, proliferation of MV4-11 cells was inhibited substantially by RSK1 KO and, to a lesser degree, by RSK2 KO (Figure 2C).

As expected, LJH685 only modestly affected BCR/ABL-dependent proliferation of K562, KU812, or MOLM-1 cells (Figure 1F and Figure S1A). Consistent with this, JLH685, as well as LJI308, inhibited RSK kinases without affecting c-Myc expression in BCR/ABL-transformed KU812 and MOLM-1 human leukemic cells, as well as in K562, while imatinib abrogated c-Myc expression without distinctly inhibiting RSKs (Figure 1B and Figure 2D). Furthermore, inhibition of RSK by LJH685 less significantly reduced proliferation of 32D cells dependent on BCR/ABL than on IL-3 (Figure 2E). However, in the another frequently used model cell line, BaF3, LJH685 reduced proliferation more prominently when cells were dependent on BCR/ABL rather than on IL-3 (Figure S1D). Nevertheless, in both model cell lines, RSK NTKD was distinctly inhibited by the JAK1/2 inhibitor ruxolitinib under the IL-3-dependent condition, but not by the BCR/ABL inhibitor imatinib when transformed by this mutant, while it was inhibited by LJH685 under both conditions (Figure 2F and Figure S1E). Thus, RSK activation may not be significantly dependent on BCR/ABL, but could play a significant role in BCR/ABL-dependent proliferation under certain cellular contexts. Together, these results suggest that FLT3-ITD and, to a lesser extent, JAK2-V617F, but not BCR/ABL, activate RSK to transduce proliferation signals, including those regulating c-Myc expression, in leukemic cells.

### 2.2. FLT3-ITD Activates RSKs through Activation of the MEK/ERK Pathway and PDK1

**Figure 3:**
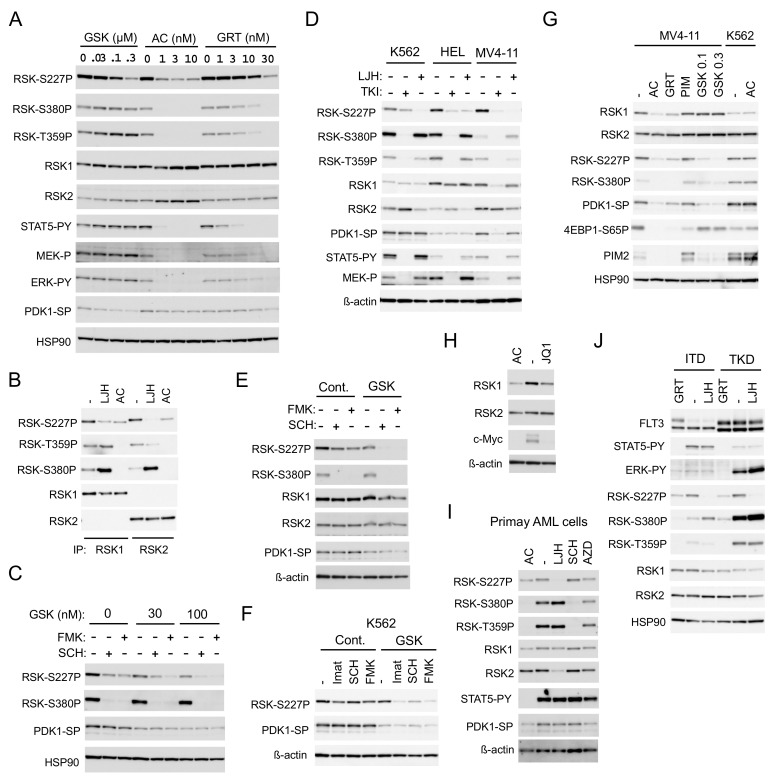
FLT3-ITD activates RSKs through activation of the MEK/ERK pathway and PDK1. (**A**) MV4-11 cells were treated for 6 h with indicated concentrations of GSK-2334470 (GSK), quizartinib (AC), or gilteritinib (GRT), as indicated. Cells were then lysed and subjected to immunoblot analysis with antibodies against indicated proteins. Abbreviations: RSK-S227P, phospho-S227-RSK2; RSK-S380P, phospho-S380-RSK1; RSK-T359P, phospho-T359/S363-RSK1; STAT5-PY, phospho-Y694-STAT5; MEK-P, phospho-S217/221-MEK; ERK-PY, phospho-T202/Y204-Erk; PDK1-SP, phospho-S241-PDK1. (**B**) MV4-11 cells were treated for 8 h with or without 5 µM LJH685 (LJH) or 50 nM quizartinib, as indicated, and lysed. Cell lysates were subjected to immunoprecipitation with ant-RSK1 or anti-RSK2 antibody, as indicated, followed by immunoblot analysis. (**C**) MV4-11 cells were treated for 6 h with or without 0.1 µM SCH772984 (SCH) or 1 µM FMK, as indicated, with indicated concentrations of GSK-2334470 and analyzed. (**D**) K562, HEL, or MV4-11 cells were treated for 16 h with or without 3 µM LJH685 or the relevant tyrosine kinase inhibitor (TKI: 2 µM imatinib for K562, 2 µM ruxolitinib for HEL, or 10 nM quizartinib for MV4-11), as indicated, and analyzed. ß-actin was used for a loading control. (**E**) MV4-11 cells were treated for 16 h with or without 0.2 µM SCH772984, 1 µM FMK, or 25 nM GSK-2334470, as indicated, and analyzed. (**F**) K562 cells were treated for 16 h with or without 1 µM imatinib (Imat), 0.2 µM SCH772984, or 1 µM FMK in the presence or absence of 100 nM GSK-2334470, as indicated, and analyzed. (**G**) MV4-11, HEL, or K562 cells were cultured for 16 h with or without 10 nM quizartinib, 30 nM gilteritinib, 2 µM PIM-447 (PIM), or indicated concentrations (µM) of GSK-2334470, as indicated, and analyzed. 4EBP1-S65P: Phospho-S65-4EBP1. (**H**) MV4-11 cells were treated for 24 h with or without 20 nM quizartinib or 1 µM JQ1, as indicated, and analyzed. **(I**) Primary FLT3-ITD-positive AML cells were treated for 24 h with or without 30 nM quizartinib, 5 µM LJH685, 0.5 mM SCH772984, or 1 µM AZD1208 (AZD), as indicated, and analyzed. (**J**) 32D/FLT3-ITD or -TKD cells inducibly expressing FLT3-ITD (ITD) or FLT3-TKD (TKD) and cultured without IL-3 were treated for 6 h with or without 3 µM LJH685 or 30 nM gilteritinib, as indicated, and analyzed.

To confirm that FLT3-ITD is involved in activation of RSKs and to explore the mechanisms involved, we examined effects of the two clinically relevant FLT3 TKIs, quizartinib and gilteritinib, on RSKs in detail. While quizartinib inhibited RSK NTKD remarkably at a very low concentration, as low as 1 nM in 6 h, more than a 10-fold higher concentration of gilteritinib was required to inhibit RSK NTKD (Figure 3A). The inhibitory effects of these inhibitors on RSK NTKD correlated with those on RSK CTKD and ERK, as well as on phosphorylation of RSK on the ERK target sites T359/S363 in RSK1 or T365/S369 in RSK2. On the other hand, gilteritinib inhibited FLT3-ITD-mediated activation of STAT5 much more efficiently than that of ERK, though a several-fold higher concentration of gilteritinib was required to inhibit STAT5 as compared with quizartinib. It was further confirmed that quizartinib inhibited both RSK1 and RSK2 comparably, although RSK2 was inhibited more profoundly than RSK1 by LJH685 (Figure 3B). As expected, inhibition of PDK1, which is required for activation of RSK NTKD, by its specific inhibitor GSK-2334470 reduced activation-specific phosphorylation of PDK1 on S241 and inhibited RSK NTKD without affecting RSK CTKD (Figure 3A). Intriguingly, quizartinib, as well as gilteritinib, at higher concentrations also reduced the PDK1 phosphorylation, though only modestly. Furthermore, GSK-2334470 enhanced inhibition of NTKD induced by the ERK inhibitor SCH772984 or the CTKD inhibitor FMK (Figure 3C), which raises the possibility that FLT3 inhibitors inhibit RSK cooperatively through inhibition of ERK and PDK1. 

We then examined longer-term effects of inhibition of the leukemogenic tyrosine kinases on RSK activation. Inhibition of BCR/ABL, JAK2-V617F, or FLT3-ITD in K562, HEL, or MV4-11 cells, respectively, by a relevant TKI for 16 h drastically inhibited RSK CTKD and phosphorylation of RSK on the ERK target sites, as well as activation of MEK and STAT5 (Figure 3D). However, inhibition of BCR/ABL inhibited RSK NTKD only modestly, while that of JAK2-V617F showed a more distinctive inhibitory effect. As expected, inhibition of FLT3-ITD for 16 h drastically inhibited RSK NTKD in MV4-11. Intriguingly, the expression level of RSK1, but not RSK2, and the activation-specific phosphorylation of PDK1 were significantly reduced by a long-term inhibition of FLT3-ITD, but not by that of BCR/ABL or JAK2-V617F in relevant cells. LJH685 prominently inhibited RSK NTKD in all of these cell lines and modestly decreased the expression level of RSK2, but not RSK1 in these cells. On the other hand, SCH772984 and FMK showed only modest long-term inhibitory effects on RSK NTKD in MV4-11, which, however, was drastically enhanced by inhibition of PDK1 by GSK-2334470 at a low concentration (Figure 3E). Furthermore, inhibition of PDK1 by GSK-2334470 at a concentration that does not affect RSK NTKD alone synergistically inhibited RSK NTKD with inhibitors for BCR/ABL, ERK, or RSK CTKD in K562 cells (Figure 3F). This implies that inhibition of PDK1 and that of ERK or CTKD may synergistically inhibit NTKD in these cells, which may, at least partly, explain the potent and selective inhibitory effect of FLT3-ITD inhibition on RSK NTKD.

Because PIM kinases have been reported to upregulate RSK2 expression through c-Myc in MV4-11 cells [18], we next examined the possible effect of PIM on the decrease in RSK1 expression induced by FLT3-ITD inhibition. As expected, inhibition of FLT3-ITD by quizartinib and gilteritinib reduced PIM2 expression, while inhibition of PIM by the specific inhibitor PIM-447 [22] upregulated PIM2 expression and reduced phosphorylation of 4EBP1 on S65, in accordance with our previous report (Figure 3G) [12]. However, unlike a long-term inhibition of FLT3-ITD, that of PIM did not show any effect on expression of RSK1 or RSK2 and activation of RSK NTKD, whereas inhibition of c-Myc by the BRD4 inhibitor JQ1 reduced expression of RSK1 but not RSK2 (Figure 3H). Quizartinib inhibited RSK, as well as PDK1, and reduced RSK1 expression in MV4-11 cells, but not in K562 cells, thus indicating that quizartinib inhibited these kinases through inhibition of FLT3-ITD, but not through off-target effects (Figure 3G). On the other hand, a long-term inhibition of PDK1 by GSK-2334470 at high concentrations inhibited RSK NTKD without affecting expression of RSK1 or RSK2 in MV4-11. It was confirmed in primary AML cells that a long-term inhibition of FLT3-ITD by quizartinib inhibited RSK NTKD, as well as PDK1, and reduced RSK1 expression, while LJH685 inhibited RSK NTKD and reduced RSK2 expression (Figure 3I). Inhibition of PIM by its specific inhibitor AZD1208 [23] failed to affect expression levels of RSK1 and RSK2 also in primary AML cells, although it slightly inhibited RSK NTKD and CTKD. These results suggest that FLT3-ITD may upregulate RSK1 expression, independent of PIM kinases, and activate RSK1 and RSK2 through activation of not only the MEK/ERK pathway, but also PDK1.

We next examined and compared the effects of FLT3-TKD and FLT3-ITD on RSK in model 32D cell lines inducibly expressing these mutants. FLT3-ITD or FLT3-TKD preferentially activated STAT5 or the MEK/ERK pathway, respectively, as reported previously (Figure 3J) [12,13]. In accordance with this, the ERK-dependent phosphorylation of RSK kinases, as well as activation of RSK CTKD, was much more prominently observed in cells expressing FLT3-TKD than FLT3-ITD. However, RSK NTKD was activated comparably in these cells and inhibited prominently by inhibition of FLT3-TKD, as well as FLT-ITD by gilteritinib and by LJH685, thus indicating that FLT3-TKD also activates RSK kinases similarly with FLT3-ITD.

### 2.3. SHP2 Interacting with Gab2 Mediates Activation of the MEK/ERK Pathway and Its Negative Feedback Regulation by RSKs in FLT3-ITD-Positive AML Cells

**Figure 4:**
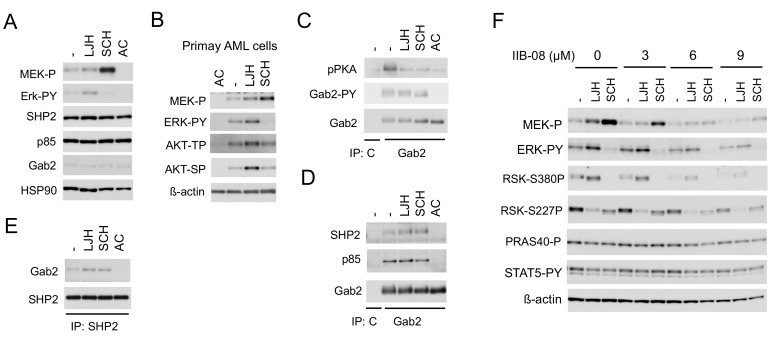
SHP2 interacting with Gab2 mediates activation of the MEK/ERK pathway and its negative feedback regulation by RSKs in in FLT3-ITD-positive AML cells. (**A**,**C–E**) MV4-11 cells were treated for 2 h with 5 µM LJH685 (LJH), 0.5 µM SCH772984 (SCH), or 10 nM quizartinib (AC), as indicated, and lysed. Cell lysates were subjected to immunoprecipitation with anti-Gab2, anti-SHP2, or control murine IgG (C), as indicated. Total cell lysates or indicated immunoprecipitates (IP) were subjected to immunoblot analysis, as indicated. Abbreviations: MEK-P, phospho-S217/221-MEK; ERK-PY, phospho-T202/Y204-Erk; pPKA, phospho-S/T-PKA substrates; Gab2-PY, phospho-Y452-Gab2. (**B**) Primary FLT3-ITD-positive AML cells were treated for 16 h with or without 20 nM quizartinib or 5 µM LJH685, as indicated, and analyzed. Abbreviations: AKT-TP, phospho-T308-AKT; AKT-SP, phospho-S473-AKT. (**F**) MV4-11 cells were pretreated for 15 min with indicated concentrations of IIB-08 and then treated for 30 min with or without 2.5 µM LJH685 or 0.5 µM SCH772984, as indicated, for immunoblot analysis. Abbreviations: RSK-S380P, phospho-S380-RSK1; RSK-S227P, phospho-S227-RSK2; PRAS40-P, phospho-T246-PRAS40.

We next examined the mechanisms involved in enhancement of ERK activation leading to activation of RSK CTKD by inhibition of RSK NTKD in cells expressing FLT3-ITD. The RSK inhibitor LJH685 enhanced activation of not only ERK, but also its upstream activator MEK, while the ERK inhibitor SCH772984 drastically enhanced activation of MEK, but not ERK in MV4-11, as well as primary FLT3-ITD-positive AML cells (Figure 4A,B). In addition, LJH685 also enhanced activation of AKT in primary AML cells, but not in MV4-11 cells. It has been reported that the adaptor protein Gab2 undergoes tyrosine phosphorylation by receptor tyrosine kinases to play a role in enhancement of the MEK/ERK or PI3K pathway through interaction with SHP2 or p85, respectively [24], and that Gab2 is phosphorylated by FLT3-ITD to interact with SHP2 and p85 [25]. Consistent with this, Gab2 in MV4-11 was phosphorylated on Y452, which was abrogated by inhibition of FLT3-ITD, but not affected by that of RSK or ERK (Figure 4C). Furthermore, phosphorylation of Gab2 was detected by using the anti-phospho-PKA substrate (R/KXXS/T) antibody reactive with phosphorylated motifs for RSK [26], which was inhibited by the inhibitor for RSK, ERK, or FLT3, as expected. Interestingly, inhibition of RSK or ERK enhanced the physical association of Gab2 with SHP2, but not p85 in these cells, while inhibition of FLT3-ITD abrogated binding of Gab2 with SHP2 and p85 (Figure 4D,E), which is mediated through interaction between the SH2 domain and tyrosine phosphorylated Gab2 [24,27]. Consistent with the idea that SHP2 is involved in activation of the MEK/ERK pathway downstream of FLT3-ITD and in the negative feedback inhibition through RSK, the specific SHP2 inhibitor IIB-08 [28] downregulated activation of this pathway in MV4-11 cells in the absence or presence of LJH685 or SCH772984, without affecting STAT5 activation by FLT3-ITD (Figure 4F). These data indicate that FLT3-ITD phosphorylates Gab2 on tyrosine to induce its binding with SHP2 and p85, and that RSKs may phosphorylate Gab2 to reduce its binding with SHP2 in these cells to downregulate the MEK/ERK pathway to cause the negative feedback regulation. 

### 2.4. RSK1 Activates the mTORC1 Pathways in FLT3-ITD-Positive AML Cells

**Figure 5:**
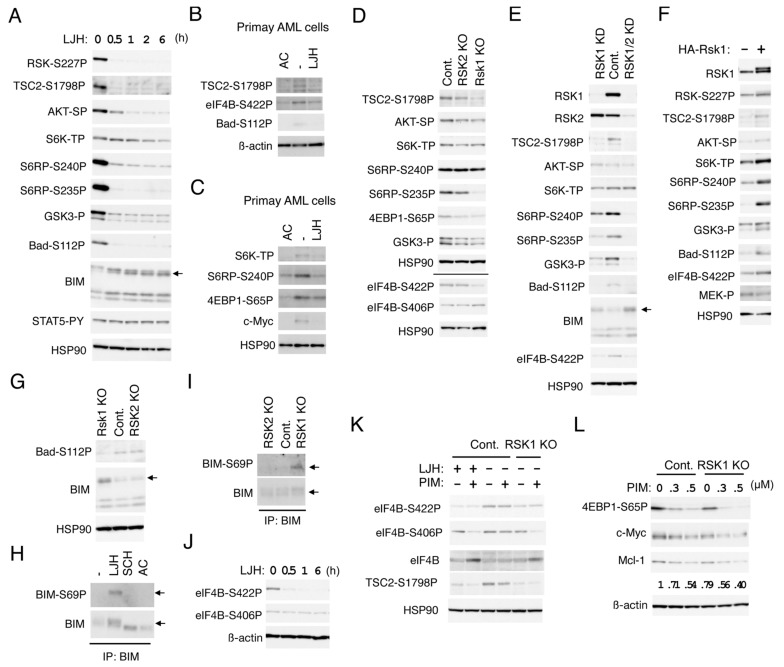
RSK1 activates the mTOR pathways and eIF4B cooperatively with PIM and negatively regulates Bad and BIM-EL in FLT3-ITD-positive AML cells. (**A**) MV4-11 cells were treated with 5 µM LJH685 (LJH) for indicated times. Cells were then lysed and subjected to immunoblot analysis with antibodies against indicated proteins. An arrow indicates the position of BIM-EL. Abbreviations: RSK-S227P, phospho-S227-RSK2; TSC2-S1798P, phospho-S1798-TSC2; AKT-SP, phospho-S473-AKT; S6K-TP, phospho-T389-p70S6K; S6RP-S240P, phospho-S240/S244-S6RP; S6RP-S235P, phospho-S235/S236-S6RP; GSK3-P, phospho-S21/9-GSK3α/ß; Bad-S112P, phospho-S112-Bad; STAT5-PY, phospho-Y694-STAT5. (**B**) Primary FLT3-ITD-positive AML cells were treated for 24 h with or without 30 nM quizartinib (AC) or 5 µM LJH685, as indicated, and analyzed. eIF4B-S422P: Phospho-S422-eIF4B. (**C**) Primary FLT3-ITD-positive AML cells were treated for 6 h with or without 20 nM quizartinib or 5 µM LJH685, as indicated, and analyzed. 4EBP1-S65P: Phospho-S65-4EBP1. (**D**) MV4-11 cells knocked out (KO) of RSK1 or RSK2, as well as vector control cells (Cont.), as indicated, were analyzed. The results obtained from duplicate gels are shown above or below a horizontal line. eIF4B-S406P: Phospho-S406-eIF4B. (**E**) MV4-11 cells knocked down (KD) of RSK1 or both RSK1 and RSK2 (RSK1/2), as well as vector control cells (Cont.), as indicated, were analyzed. An arrow indicates the position of BIM-EL. (**F**) MV4-11 cells expressing HA-RSK1 (+) of vector control cells (−), as indicated, were analyzed. (**G**) Cells indicated were analyzed. An arrow indicates the position of BIM-EL. (**H**) MV4-11 cells were left untreated as control (−) or treated for 16 h with 5 µM LJH685, 0.5 µM SCH772984, or 10 nM quizartinib, as indicated, and lysed. Cell lysates were subjected to immunoprecipitation (IP) with ant-BIM antibody, followed by immunoblot analysis. An arrow indicates the position of BIM-EL. BIM-S69P: Phospho-S69-BIM. (**I**) Cells indicated were analyzed. (**J**) MV4-11 cells were treated with 5 µM LJH685 for indicated times and analyzed. (**K**) Vector control MV4-11 cells or RKS KO cells were treated for 6 h with 1 µM LJH685 or 0.1 µM PIM-447 (PIM), as indicated, and analyzed. (**L**) Indicated cells were treated for 6 h with indicated concentrations of PIM-447 and analyzed. Relative expression levels of Mcl-1 were determined by densitometric analysis and are shown below the panel.

To explore substrates of RSK and downstream signaling pathways mediated by RSK in FLT3-ITD-positive AML cells, we first examined time-course effects of the most specific RSK inhibitor, LJH685, on phosphorylation of candidates of substrates in MV4-11 cell. At a concentration not affecting activation of STAT5, LJH685 rapidly inhibited RSK NTKD and reduced phosphorylation of TSC2 on S1798, S6RP on S235/S236, as well as S240/S244, and GSK3α/ß on S21/9, which was observed more rapidly than when FLT3-ITD was inhibited by quizartinib, and correlated with the time course of RSK inhibition (Figure 5A and Figure S2A,B). On the other hand, phosphorylation of AKT on S473 and S6K on T389, known to be phosphorylated by mTORC1 or mTORC2, respectively, were reduced more gradually. Thus, it is speculated that these sites of TSC2, S6RP, and GSK3α/ß may be directly phosphorylated by RSK, while the mTORC1 or mTORC2 pathway may be activated indirectly downstream of direct substrates including theses and presumably in collaboration with other signaling pathways activated by FLT3-ITD. It was confirmed that LJH685 reduced phosphorylation of TSC2 on S1798, as well as S6RP on S240/S244, inhibited the mTORC1 pathway as judged by decreases in phosphorylation of S6K on T389 and 4EBP1 on S65P, and reduced c-Myc expression also in primary FLT3-ITD-positive AML cells from one patient we could examine (Figure 5B,C). 

To avoid possible off-target effects of LJH685 and to evaluate the relative roles of RSK1 and RSK2 in phosphorylation of substrates identified using LJH685, we then analyzed RSK1 KO or RSK2 KO MV4-11 cells (Figure 5D). Phosphorylation of TSC2 on S1798 or S6RP on S235/6 was reduced distinctively in RSK1 KO cells, but only modestly in RSK2 KO cells as compared with vector control cells. On the other hand, phosphorylation of GSK3α/ß on S21/9 or 4EBP1 on S65 was reduced only modestly in both RSK1 and RSK2 KO cells, while that of AKT on S473, S6K on T389, or S6RP on S240/1 was not distinctly affected in these cells. Because we failed to obtain RSK1/RSK2 double KO cells, we generated and analyzed the double KD MV4-11 cells, in which both RSK1 and RSK2 were knocked down. While RSK1 KD cells showed similar phosphorylation patterns of substrates with RSK1 KO cells, phosphorylation of S6RP on 240/244 and GSK3α/ß on S21/9 were more remarkably reduced in RSK1/RSK2 double KD cells than in RSK1 KD cells (Figure 5E). However, phosphorylation of AKT on S473 or S6K on T389 was not clearly affected, even in double KD cells. We also generated and examined MV4-11 cells overexpressing RSK1 and confirmed that phosphorylation of TSC2 on S1798, S6RP on S235/6, as well as S240/S244, GSK3α/ß on S21/9, AKT on S473, or S6K on T389 was increased in these cells as compared with vector control cells, while activation-specific phosphorylation of MEK was faintly reduced by overexpression of RSK1 (Figure 5F). Together, these results suggest that RSK1 may play a dominant role in phosphorylation of TSC2 on S1798, S6RP on S235/6, while RSK2 may also phosphorylate S6RP on S240/S244 and GSK3α/ß on S21/9, along with RSK1. It is speculated that activation of the mTOR pathways was compensated by other activation mechanisms downstream of FLT3-ITD in cells deficient in RSK. 

### 2.5. RSK1 Negatively Regulates Bad and BIM-EL in FLT3-ITD-Positive AML Cells 

We next examined the possibility that RSKs may be involved in regulation of the Bcl-2 family members, regulating survival of FLT3-ITD-driven AML cells. Inhibition of RSKs by LJH685 reduced phosphorylation of Bad on S75 corresponding to S112 in mice much more rapidly than inhibition of FLT3-ITD by quizartinib in MV4-11 cells (Figure 5A and Figure S2A). Consistent with this, phosphorylation of Bad on S75 was decreased in RSK1 KO or KD cells as compared with vector control cells, while it was increased by overexpression of RSK1 (Figure 5E–G). LJH685, as well as quizartinib, inhibited phosphorylation of Bad on S75 also in primary FLT3-ITD-positive AML cells (Figure 5B). On the other hand, inhibition of RSKs or KO of RSK1 did not affect phosphorylation of Bad on S99, corresponding to S136 in mice (Figure S2C and negative data not shown). These data suggest that RSK1 may mediate phosphorylation of Bad on S75 downstream of FLT3-ITD in AML cells, which is known to inactivate this pro-apoptotic Bcl-2 family member by preventing its binding to anti-apoptotic proteins [29].

On the other hand, expression levels of BIM, particularly that of BIM-EL, were rapidly increased by LJH685 and were also increased in RSK1 KO or KD cells and, more conspicuously, in RSK1/RSK2 KD cells (Figure 5A,E,G). SCH772984 or quizartinib also increased expression of BIM-EL, which, however, migrated faster than when RSK was inhibited (Figure 5H and Figure S2B). Moreover, ERK inhibition caused BIM-EL accumulated in RSK1 KO cells to migrate faster (Figure S2C). Thus, we next examined phosphorylation of the ERK target site S69 in BIM-EL. As shown in Figure 5H, BIM-EL phosphorylation at S69 was observed in MV4-11 cells treated with LJH685, but not with SCH772984 or quizartinib. Consistent with this, BIM-EL phosphorylated on S69 was observed only in RSK1 KO cells, and not in RSK2 KO or vector control MV4-11 cells (Figure 5I). These results are consistent with the idea that RSK1 may promote degradation of BIM-EL phosphorylated on S69 by ERK in FLT3-ITD-positive AML cells, which may play an anti-apoptotic role by reducing this pro-apoptotic member of the Bcl-2 family. 

### 2.6. RSK1 and PIM Play Cooperative Roles Downstream of FLT3-ITD in Enhancing the mTORC1/eIF4F Pathway and Phosphorylation of eIF4B

We next examined phosphorylation of eIF4B, which promotes cap-dependent translation mediated through eIF4F complex regulating expression of various proteins important for cell proliferation and survival, including Mcl-1 [7]. Phosphorylation of eIF4B on S422 was decreased more rapidly by LJH685 than by quizartinib in MV4-11 cells and correlated with the expression level of RSK1 in KO or KD cells, as well as in overexpressing cells (Figure 5D–F,J and Figure S2A), thus suggesting that it is directly mediated by RSK1. LJH685 reduced eIF4B phosphorylation at S422 also in primary FLT3-ITD-positive AML cells (Figure 5B). On the other hand, phosphorylation of eIF4B on S406 was not affected by LJH685, but was decreased gradually but remarkably by quizartinib, thus indicating that it is induced downstream of FLT3-ITD (Figure 5J and Figure S2A). Furthermore, although inhibition of PIM alone by PIM-447 did not affect phosphorylation of eIF4B on either S406 or S422, it remarkably reduced phosphorylation of eIF4B on S406 in combination with LJH685 or in RSK1 KO cells (Figure 5K and Figure S2D). These results suggest that, in addition to phosphorylating eIF4B on S422, RSK1 collaborates with PIM to phosphorylate eIF4B on S406, which is expected to enhance eIF4F-mediated cap-dependent translation [7]. On the other hand, the PI3K inhibitor also inhibited phosphorylation of eIF4B on S422, as well as activation of AKT, without affecting phosphorylation of eIF4B on S406, TSC2 on S1798, Bad on S112, and GSK3α/ß on S21/9 (Figure S2D). We also examined the combined effects of PIM and RSK inhibition on the mTORC1 pathway regulating formation of eIF4F. PIM-447 inhibited S6K and decreased phosphorylation of 4EBP1 on S65 in MV4-11 cells (Figure 5L and Figure S2E), in accordance with our previous report [12]. PIM-447 also reduced phosphorylation of TSC2 on S1798, though only faintly (Figure 5K and Figure S2D). Furthermore, the inhibitory effects of PIM-447 on the mTORC1 pathway were more remarkably observed in combination with LJH685 or in RSK1 KO cells (Figure 5L and Figure S2E). Consistent with this, inhibition of PIM and RSK1 cooperatively decreased Mcl-1, as well as c-Myc expression in these cells. Together, these results imply that RSK1 and PIM play cooperative roles downstream of FLT3-ITD in enhancing eIF4F-mediated cap-dependent translation through phosphorylation of eIF4B and 4EBP1.

### 2.7. Inhibition of RSK and That of PIM or PI3K Cooperatively Inhibit Proliferation and Cooperatively Induce Apoptosis through the Mitochondrial Apoptotic Pathway in FLT3-ITD-Positive AML Cells

**Figure 6:**
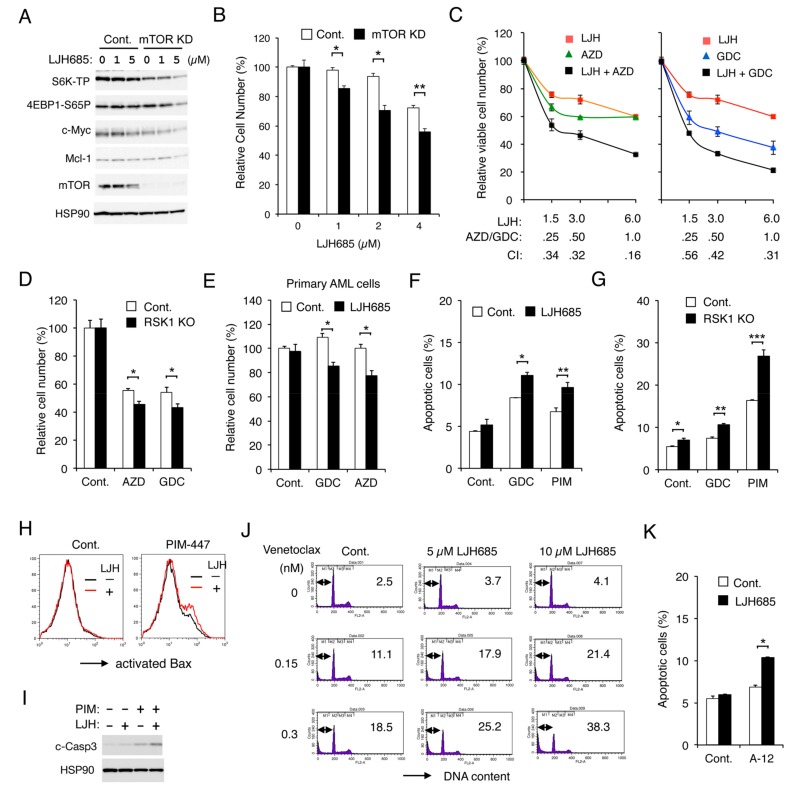
Inhibition of RSK and that of PIM or PI3K cooperatively inhibit proliferation and cooperatively induce apoptosis through the mitochondrial apoptotic pathway in FLT3-ITD-positive AML cells. (**A**) MV4-11 cells knocked down of mTOR (mTOR KD) or vector control cells (Cont.), as indicated, were treated for 6 h with indicated concentrations of LJH685 and subjected to immunoblot analysis. (**B**) Indicated cells were cultured for 48 h with indicated concentrations of LJH685, and viable cell numbers were measured by the colorimetric assay. Each column represents the mean of triplicate cultures, with error bars indicating standard errors, and is expressed as a ratio to the control cell numbers. The asterisks indicate statistically significant differences from control cells determined by Student’s *t*-test (* *p* < 0.005, ** *p* < 0.01). (**C**) MV4-11 cells were cultured for 48 h with indicated concentrations (µM) of LJH685 (LJH), AZD1208 (AZD), and GDC-941 (GDC), and viable cell numbers were measured in triplicate. Combination index (CI) values are indicated. (**D**) MV4-11 cells knocked out of RSK1(RSK1 KO) or vector control cells (Cont.) were cultured in quadruplicate for 48 h with or without 0.3 µM AZD1208 or GDC-0941, as indicated, and viable cell numbers were measured. * *p* < 0.05. (**E**) Primary AML cells expressing FLT3-ITD were cultured in the absence (Cont.) or presence of 2.5 µM LJH685 with 0.2 µM GDC-0941 or AZD1208, as indicated, for 24 h in quadruplicate, and viable cell numbers were counted after trypan blue staining. * *p* < 0.005. NS: Not significant. (**F**) MV4-11 cells were left untreated as control (Cont.) or treated with 2.5 µM LJH685, 0.5 µM GDC-0941, or 1 µM PIM-447 (PIM), as indicated, for 16 h, and analyzed. The means of percentages of apoptotic cells with sub-G1 DNA content from triplicate measurements are plotted. * *p* < 0.01, ** *p* < 0.05. (**G**) MV4-11 cells knocked out of RSK1 (RSK1 KO) or vector control cells (Cont.) were left untreated as control (Cont.) or treated with 2 µM GDC-0941 or 3 µM PIM-447, as indicated, for 16 h, and analyzed. * *p* < 0.05, ** *p* < 0.001, *** *p* < 0.005. (**H**) MV4-11 cells were treated with or without 2 µM LJH685 in the presence of 3 µM PIM-447 or in its absence (Cont.) for 16 h and analyzed for Bax activation by flow cytometry. (**I**) MV4-11 cells were treated for 16 h with 5 µM LJH685 and 3 µM PIM-447, as indicated, and analyzed. c-Casp3: Cleaved Caspase-3. (**J**) MV4-11 cells were treated in the absence (Cont.) or presence of LJH685 with indicated concentrations of venetoclax for 4 h. The cellular DNA content was measured by flow cytometry, and percentages of apoptotic cells with sub-G1 DNA content are indicated. (**K**) MV4-11 cells were left untreated as control (Cont.) or treated with 2.5 µM LJH685 or 10 nM A-1210477, as indicated, for 16 h, and analyzed. * *p* < 0.0001.

We previously reported that FLT3-ITD activates the mTORC1/S6K/4EBP1 pathway cooperatively through PIM and PI3K in AML cells to promote proliferation and prevent apoptosis mediated through the intrinsic mitochondria-mediated pathway. Thus, we examined whether RSK1 and PIM or PI3K may also cooperatively promote proliferation and survival through upregulation of the mTORC1 pathway. First, we confirmed that the mTORC1 pathway should play a role in RSK-mediated enhancement of FLT3-ITD-dependent proliferation by revealing that the mTORC1 pathway was more remarkably downregulated by LJH685 in mTOR KD MV4-11 than vector control cells, which correlated with decreases in Mcl-1 and c-Myc expression, as well as inhibition of proliferation (Figure 6A,B). Next, we revealed that LJH685 and AZD1208 or GDC-0941 synergistically reduced viable cell numbers of MV4-11, as judged by combination index values obtained by the method of Chuo and Talalay [30] being less than 1 at all the concentrations examined (Figure 6C). In accordance with this, AZD1208 or GDC-0941 reduced viable cell numbers of RSK1 KO MV4-11 cells more significantly than vector control cells (Figure 6D). It was further confirmed that, under the conditions where LJH685 alone did not show any significant effect, it significantly reduced viable cell numbers of primary FLT3-ITD-positive AML cells in combination with AZD1208 or GDC-0941 (Figure 6E).

Inhibition of RSK alone induced apoptosis only modestly in MV4-11 (Figure S3A). However, under the conditions where LJH685 alone did not significantly induce apoptosis, it significantly enhanced apoptosis induced by GDC-0941 or PIM-447 (Figure 6F). In accordance with this, RSK1 KO cells showed increased sensitivities to GDC-0941 or, more remarkably, to PIM-447 for induction of apoptosis as compared with control cells (Figure 6G). We also examined the mechanisms of induction of apoptosis and found that LJH685 enhanced Bax activation induced by PIM-447 when LJH685 alone did not active Bax in MV4-11 (Figure 6H). LJH685 also enhanced the activation/cleavage of Caspase-3 induced by PIM-447 (Figure 6I), while the pan-Caspase inhibitor Boc-d-FMK abolished activation of Caspase-3 and induction of apoptosis in MV4-11 co-treated with these inhibitors (Figure S3B). Furthermore, inhibition of RSKs also increased sensitivities of MV4-11 to the BH3 mimetics venetoclax and A-1210477, which specifically inhibit the antiapoptotic Bcl-2 family members Bcl-2 and Mcl-1, respectively (Figure 6J,K). Consistent with this, overexpression of Bcl-xL or Mcl-1 endowed MV4-11 with resistance to apoptosis induced by the combined inhibition of RSK and PI3K or PIM (Figure S3C). Together, these results suggest that RSK1 cooperates with PI3K and PIM to promote proliferation of leukemic cells downstream of FLT3-ITD and to prevent apoptosis, at least partly, through activation of the mTORC1 pathway and inhibition of the mitochondrial apoptotic pathway regulated by the Bcl-2 family members.

## 3. Discussion

**Figure 7:**
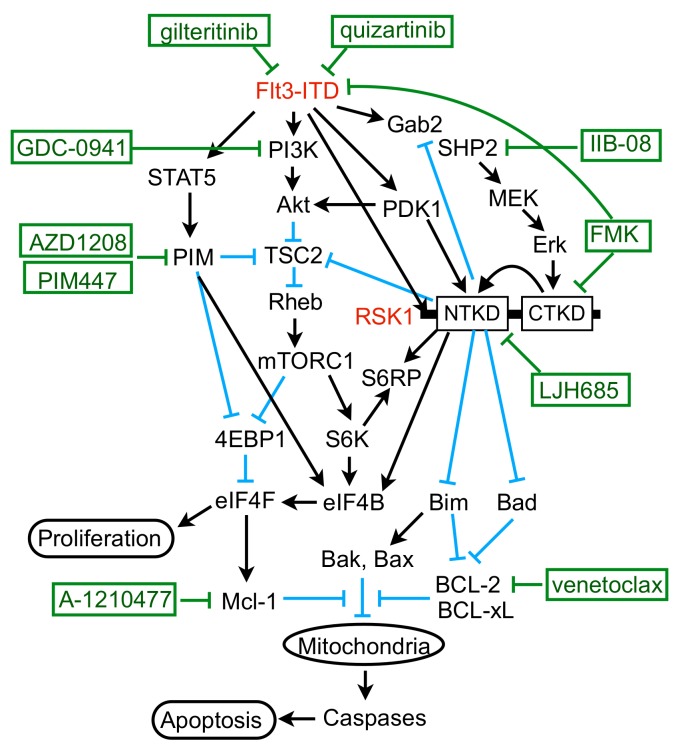
Schematic model for molecular mechanisms by which FLT3-ITD enhances proliferation and survival of AML cells through activation of RSK1.

In the present study, we have revealed that FLT3-ITD, but not BCR/ABL, distinctively activates RSK1, as well as RSK2, in leukemic cells. Previously, RSK1 or RSK2 was reported to be activated in various leukemic cells or by BCR/ABL or FLT3-ITD [17,31]. In these studies, however, only the activation-specific phosphorylation of CTKD, which plays a role in activation of NTKD, but not in phosphorylation of downstream signaling molecules, has been examined to demonstrate activation of RSKs. In contrast, we have evaluated the activation specific phosphorylation of NTKD and confirmed that RSKs were activated in several leukemic and model cell lines expressing FLT3-ITD, BCR/ABL, or JAK2-V617F, as well as in the primary FLT3-ITD-positive AML cells we could examine (Figure 1B, Figure 2D,F and Figure S1E). However, although activation of CTKD, as well as ERK-dependent phosphorylation of RSK in the turn motif in the linker legion, was dependent on these leukemogenic tyrosine kinases, that of NTKD was not significantly inhibited by the BCR/ABL inhibitor imatinib in several Ph-positive leukemic cells or model cell lines transformed by BCR/ABL (Figure 1B, Figure 2D,F and Figure S1E). In contrast, inhibition of the FLT3 mutants or JAK2-V617F distinctly or moderately inhibited RSK NTKD, respectively, in leukemic cells, including primary AML cells or a model hematopoietic cell line transformed by FLT3-ITD, as well as FLT3-TKD (Figure 1B and Figure 2). In this regard, it is notable that the ERK inhibitor SCH772984 or the RSK CTKD inhibitor FMK clearly inhibited RSK NTKD only in FLT3-ITD-driven MV4-11 cells, but not in BCR/ABL-driven K562 or JAK2-V617F-driven HEL cells, whereas it remarkably inhibited CTKD and the ERK-dependent phosphorylation of RSK in all these cells (Figure 1B). In addition, inhibition of FLT3-ITD, but not BCR/ABL or JAK2-V617F, in the relevant cells was found to reduce activation specific phosphorylation of PDK1 in MV4-11, as well as primary FLT3-ITD-positive AML cells (Figure 3A,D,F). Furthermore, downregulation of PDK1 activity by its specific inhibitor GSK-2334470 increased sensitivities of RSK NTKD to SCH772984 and FMK in MV4-11 cells, and made RSK NTKD in K562 sensitive to not only these inhibitors, but also to the BCR/ABL inhibitor imatinib (Figure 3C,E,F). Thus, the present study suggests that FLT3-ITD may activate RSKs through activation of not only the MEK/ERK pathway, but also PDK1 in FLT3-ITD-dependent cells, while BCR/ABL may activate only RSK CTKD through activation of the MEK/ERK pathway, although the mechanisms involved in upregulation of PDK1 activity by FLT3-ITD remain to be examined in future studies (Figure 7).

The present study has also shown that a long-term inhibition of FLT3-ITD decreased expression of RSK1, but not RSK2 in MV4-11, as well as in primary FLT3-ITD-positive AML cells from one patient we could examine (Figure 3A,G,I). On the other hand, Hospital M-A et al. [18] recently reported that inhibition of FLT3-ITD or KD of PIM2 decreased RSK2 expression in FLT3-ITD-positive AML cell line MOLM-14, although PIM2 inhibition decreased RSK2 expression in various AML cells regardless of their FLT3 mutational status, and its possible effect on RSK1 expression was not addressed. PIM2 KD also downregulated c-Myc expression, and downregulation of c-Myc by BRD4 inhibitors decreased RSK2 expression in MOLM-14 cells. Furthermore, PIM2 induced phosphorylation of the activation loop of RSK2 CTKD when these kinases were transiently overexpressed in 293T cells. Thus, the authors speculated that FLT3-ITD upregulates RSK2 through PIM2 by enhancing c-Myc-dependent transcription, as well as phosphorylation of CTKD [18]. In the present study, RSK1, but not RSK2, was decreased by inhibition of FLT3-ITD in MV4-11 and primary AML cells, while inhibition of PIM did not show any effect on expression level of RSK1 or RSK2 in these cells (Figure 3G). However, inhibition of PIM slightly inhibited activation of RSK CTKD, as well as NTKD, in primary FLT3-ITD-positive AML cells, but not in MV4-11, while the BRD4 inhibitor JQ1 downregulated expression of RSK1, but not RSK2 in MV4-11 cells (Figure 3G,H,I). Reasons for the discrepancies between our data and those reported by Hospital, M-A. et al. [18] remain to be clarified and might be, at least partly, due to differences in cellular context. In this regard, it should be noted that AML is heterogeneous and we could perform detailed investigations in only one case of FLT3-ITD-positive AML in addition to MV4-11. Nevertheless, the present study revealed that, in addition to activating RSK1 and RSK2, FLT3-ITD is able to increase expression of RSK1, but not RSK2, through mechanisms possibly involving c-Myc, but not PIM, in some AML cells.

The present study has revealed that RSKs, mostly RSK1, play an important role in promotion of proliferation and survival of cells transformed by FLT3-ITD, but not BCR/ABL. This is partly compatible with a previous report by Elf, S. et al. [17] that RSK2 is essential for development of the myeloproliferative disorder in mice induced by FLT3-ITD, but dispensable for that induced by BCR/ABL. In contrast to our study, however, the authors reported that both BCR/ABL and FLT3-ITD activated RSK2 in leukemic cells, which is most likely because only activation specific phosphorylation of RSK CTKD was examined. The authors also reported that activation of RSK2 played a role in proliferation and survival of leukemic cells transformed by FLT3-ITD, but not BCR/ABL, such as MV4-11 and K562, respectively. However, this was mostly based on experiments using the RSK CTKD inhibitor FMK, which was revealed in the present study to barely inhibit RSK NTKD in K562 and to inhibit FLT3-ITD very efficiently at high concentrations, such as 12.94 µM reported as the cellular IC_50_ value for MV4-11 [17]. On the other hand, we used the highly specific RSK NTKD inhibitor LJH685 to reveal the selective dependence of FLT3-ITD transformed cells. but not BCR/ABL-transformed leukemic cells on RSKs. Elf, S. et al. [17] further demonstrated that transient KD of RSK2, but not RSK1, induced apoptosis remarkably in FLT3-ITD-transformed cells including MV4-11, whereas KO of RSK1 reduced proliferation of MV4-11 cells more significantly than KO of RSK2 without remarkably inducing apoptosis in the present study (Figure 2C and Figure 6G). This discrepancy may be partly due to the differences in experimental procedures, and the significance of RSK1 and RSK2 in FLT3-ITD-positive AML cells might be relative and conditional. Although molecular mechanisms underlying the difference in dependence on RSKs observed between FLT3-ITD- and BCR/ABL-transformed cells remain to be examined, it may be important that c-Myc expression was dependent on RSKs in the former cells, but not in the latter cells (Figure 1B and Figure 2D). 

We previously revealed that FLT3-ITD activates the mTORC1/S6K/4EBP1 pathway cooperatively through the STAT5/PIM and PI3K/AKT pathways to enhance proliferation and survival of AML cells [12,13], which was extended in the present study by revealing that the MEK/ERK/RSK pathway also works cooperatively with these pathways. RSK has been reported to activate mTORC1 by phosphorylating TSC2 on S1798 [32,33], which was confirmed in FLT3-ITD-positive AML cells in the present study (Figure 5A,B,D–F). Previously, PIM was also reported to inactivate TSC2 by phosphorylating S1798 of this negative regulator for mTORC1 in multiple myeloma cells [34]. Consistent in part with this, the present study has shown that PIM inhibition in MV4-11 cells reduced this phosphorylation, which, however, was much less remarkable than that observed with RSK inhibition (Figure 5K and Figure S2D). On the other hand, we and others have reported that PIM upregulates the mTORC1/S6K/4EBP1 pathway through various other mechanisms involving PRAS40, 4EBP1, and AMPK [12,35,36,37]. Thus, future studies are warranted to examine possible involvement of these and other mechanisms, such as Raptor phosphorylation [38], downstream of RSK to upregulate the mTORC1 pathway cooperatively with PIM or AKT (Figure 7).

In addition to upregulating the mTORC1/S6K/4EBP1/eIF4F pathway, RSK1 was demonstrated to phosphorylate not only S6RP, but also eIF4B cooperatively with PIM in FLT3-ITD-positive AML cells, which is known to enhance protein synthesis, along with eIF4F [7]. It has been reported that S6K phosphorylates S6RP on S235, S236, S240, and S244 downstream of mTORC1, while RSK phosphorylates it exclusively on S235 and S236 independently of mTORC1, thus promoting recruitment of the 40S ribosome to the mRNA [8]. The present study has revealed that RSK may contribute to phosphorylation of all the four serine residues of S6RP indirectly through upregulation of the mTORC1/S6K pathway or directly through phosphorylation of S240/S244, in addition to S235/S236, as judged by the time course of inhibition by LJH685 or by its inhibition in RSK1/RSK2 double KD cells (Figure 5A,E). Previously, RSK and S6K have been reported to phosphorylate eIF4B on S422 in cell type- and stimulus-dependent manners [39,40] and also implicated in its phosphorylation on S406 upon mitogen stimulation, because it was abrogated by inhibition of both MEK and mTORC1 [41]. We have also reported that inhibition of S6K modestly reduced phosphorylation of eIF4B on S406 and S422 in MV4-11 cells [14]. On the other hand, Cen B et al. [42] reported that phosphorylation of eIF4B on S406 was not inhibited by inhibition of both MEK and mTORC1, but blocked by inhibition of PIM in various tumor cells, including AML cells. Furthermore, PIM was reported to phosphorylate eIF4B mainly on S422 and, to a lesser extent, on S406 in cells transformed by Abl mutants, including BCR/ABL [43]. Thus, RSK, S6K, and PIM cooperatively contribute in different ways depending on types of cells and stimuli to phosphorylation of eIF4B on S422 or S406, which is required for its activation to stimulate the eIF4F activities and for its binding to the eIF3 translation initiation complex [7]. The present study revealed that RSK1 plays a critical role in phosphorylation of eIF4B on S422 and a cooperative role with PIM in that on S406. Thus, RSK1 should play a central role in upregulation of translation by activating eIF4B, in addition to enhancing the eIF4F complex formation through phosphorylation of 4EBP1 and inducing S6RP phosphorylation in FLT3-ITD-positive AML cells (Figure 7). 

The present study has revealed the involvement of SHP2, which interacts with Gab2 phosphorylated by FLT3-ITD, in activation of the MEK/ERK pathway, as well as its negative feedback regulation by RSK in FLT3-ITD-positive AML cells (Figure 7). Interaction of SHP2 or p85 with tyrosine phosphorylated Gab2 through their SH2 domains has been well established to play a role in activation of the MEK/ERK or PI3K/AKT pathway, respectively, downstream of several tyrosine-kinase or cytokine receptors [24,27]. Furthermore, it was reported that FLT3-ITD tyrosine phosphorylated Gab2 to induce interaction with SHP2, as well as p85 [25], and that KD of Gab2 in MV4-11 cells attenuated activation of the MEK/ERK and PI3K/AKT pathways, as well as STAT5 [44]. The present study has extended these observations by revealing that the SHP2 phosphatase activity is required for FLT3-ITD-induced activation of the MEK/ERK pathway, but not that of the PI3K/AKT pathway or STAT5 (Figure 4F). Interaction of SHP2 with Gab2 has also been reported to underlie the negative feedback regulation mechanisms of the MEK/ERK/RSK pathway. In this regard, ERK and RSK have been reported to phosphorylate Gab2 on serine residues near the SH2 domain-binding sites for SHP2, thus downregulating its interaction with Gab2 and activation of the MEK/ERK pathway [45,46]. Although we could not identify the phosphorylation sites of Gab2 by RSKs or ERK, our results are consistent with these previous reports (Figure 4C,D). Gab2 phosphorylation by ERK was also reported to inhibit tyrosine phosphorylation of Gab2 required for recruitment of p85, thus negatively regulating PI3K activation in mast cells [47]. Although we could not observe any effect of ERK or RSK inhibition on interaction of p85 with Gab2 or on its phosphorylation on Y452 in MV4-11 cells (Figure 4C,E), it should be noted that inhibition of RSKs remarkably enhanced activation of AKT in primary FLT3-ITD-positive AML cells we could examine (Figure 4B). Thus, activation of these signaling events induced by RSK inhibition needs to be carefully addressed before developing therapies targeting RSKs.

RSK1 was previously shown in IL-3-dependent model cell lines to mediate the MEK/ERK pathway cell survival signal through phosphorylation of Bad on S112 in mice [48], which abrogates its pro-apoptotic function by promoting its dissociation from the anti-apoptotic Bcl-2 family members [10]. FLT3-ITD was also shown in 32D cell transformants to induce Bad phosphorylation at S112 through the MEK/ERK pathway, which contributed to cell survival [49]. Furthermore, it was later reported that KD of RSK1, as well as inhibition of the MEK/ERK pathway, reduced Bad phosphorylation at S112 in FLT3-ITD-transformed BaF3 cells [31]. Consistent with these studies, the present study has clearly demonstrated that RSK1 activated by FLT3-ITD plays a critical role in phosphorylation of Bad on S75 corresponding to S112 in mice, because it was rapidly abrogated by LJH685 in parallel with inhibition of RSK NTKD in MV4-11 cells, was inhibited by LJH685 also in primary FLT3-ITD-positive AML cells from one patient we could examine, and was remarkably reduced or enhanced by RSK1 deletion or overexpression, respectively, in MV4-11 cells (Figure 5A,B,E–G). The present study further demonstrated that inhibition or depletion of RSK1 increased BIM-EL expression in the S69-phosphorylated form, while inhibition of ERK or FLT3-ITD increased BIM-EL in the S69-non-phosphorylated form (Figure 5H,I). This is consistent with a previous report that phosphorylation of BIM-EL on S69 by ERK facilitated its phosphorylation by RSKs leading to degradation by the ubiquitin/proteasome system [50]. Intriguingly, although rapidly degraded when phosphorylated by ERK, BIM-EL phosphorylated on S69 by the stress-induced MAP kinase JNK or p38 was reported to show an increased pro-apoptotic activity [51]. BIM is reported to be downregulated by FLT3-ITD by the FOXO3a-dependent transcriptional mechanism and to play a critical role in TKI-induced apoptosis in FLT3-ITD-positive AML cells [52,53]. The present study has revealed that RSK1 contributes profoundly to downregulation of BIM-EL through the post-translational mechanism, which should play a significant role in survival of FLT3-ITD-positive AML cells. In contrast to a previous report [18], inhibition of RSKs did not increase Bax expression in FLT3-ITD-positive AML cells (Figure S2B). LJH685 alone also hardly activated Bax (Figure 6H), which BIM was reported to activate preferentially [54]. However, in the presence of PIM inhibitor, inhibition of RSKs distinctly increased activation of Bax and Caspase-3, as well as induction of apoptosis in MV4-11 cells, which was abrogated by overexpression of Bcl-xL or Mcl-1, as well as by inhibition of caspases (Figure 6H and Figure S3B,C). Furthermore, inhibition of RSKs sensitized these cells to BH3 mimetics, inhibiting Mcl-1 or Bcl-2 (Figure 6J,K). Taken together, the present study suggests that RSK1 may protect FLT3-ITD-positive AML cells from apoptosis induced through the intrinsic mitochondrial pathway by negatively regulating Bad, as well as BIM, and by upregulating Mcl-1 or other anti-apoptotic proteins through activation of the mTORC1/eIF4F pathway and eIF4B cooperatively with the STAT5/PIM and PI3K/AKT pathways (Figure 7). Thus, RSK1 should represent a promising molecular target, particularly in combination with PIM or PI3K, for novel therapeutic strategies against therapy-resistant FLT3-ITD-positive AML.

## 4. Materials and Methods 

### 4.1. Cells and Reagents

MV4-11 cells were purchased from ATCC and cultured in Iscove’s modified Dulbecco medium (IMDM) containing 10% FCS. MV4-11 cells overexpressing Mcl-1 or Bcl-xL, MV4-11/Mcl-1 or MV4-11/Bcl-xL, respectively, as well as those knocked down of mTOR, MV4-11/sh-mTOR, have been described previously [12,55]. Murine IL-3-dependent 32Dcl3 cells, Ton.32D/FLT3-ITD (32D/ITD) or Ton.32D/FLT3-D835Y (32D/TKD), inducibly expressing FLT3-ITD or FLT3-D835Y, respectively, when cultured with doxycycline have been described previously [56], and were maintained in RPMI 1640 medium supplemented with 10% FCS and 5 U/ml recombinant murine IL-3 (PeproTech, Rocky Hill, NJ, USA). Before analyses, these cells were cultured in medium containing doxycycline without IL-3 to proliferate dependent on FLT3-ITD or FLT3-TKD and independent of IL-3. Ton.32D cells derived from 32Dcl3 and Ton.32D210 cells expressing BCR/ABL have been described previously [57]. Ton.B210, a clone of murine IL-3-dependent BaF3 cells inducibly expressing BCR/ABL when cultured with doxycycline, was kindly provided by George Daley [58]. JAK2-V617F-positive leukemic cell lines PVTL-1 and PVTL-2 have previously been reported [59,60]. KU812 and K562 were obtained from the Riken cell bank (Ibaraki, Japan). HEL and MOLM-1 cells were obtained from the Fujisaki Cell Center (Okayama, Japan). These leukemic cells were maintained in RPMI 1640 medium supplemented with 10% FCS.

Quizartinib and venetoclax were purchased from LC laboratories (Woburn, MA, USA). Gilteritinib, GDC-0941, and AZD1208 were from Active Biochem (Kowloon, Hong Kong, China). LJH685, SCH772984, and JQ1 were from Cayman Chemical (Ann Arbor, MI, USA). PIM-447 was from Selleckchem (Houston, TX, USA). A-1210477 was from Chemietek (Indianapolis, IN, USA). LJI308, doxycycline, propidium iodide, and antibody against β-actin (A1978) were from Sigma-Aldrich (St. Louis, MO, USA). Imatinib was kindly provided by Novartis (Basel, Switzerland). FMK was from MedChem Express (Monmouth Junction, NJ, USA). Ruxolitinib and IIB-08 were from Calbiochem (La Jolla, CA, USA). GSK-2334470 was from LKT Laboratories (St. Paul, MN, USA). Boc-d-FMK was from BioVision (Milpitas, CA, USA). 

Monoclonal antibody against activated Bax (TACS-2281) was purchased from Trevigen (Gaithersburg, MD, USA). The anti-mouse IgG APC-conjugated antibody was from Biotech R&D systems (Minneapolis, MN, USA). Anti-RSK2 (PGI 23762-1-AP) was from Proteintech Group, Inc. (Rosemont, IL). Antibodies against FLT3 (SC479), phospho-S1798-TSC2 (SC293149), RSK1 (SC231), Gab2 (SC365590), c-Abl (SC131), Bax (SC493), Bad (SC7869), SHP2 (SC280), and HSP90 (SC13119) were purchased from Santa Cruz Biotechnology (Santa Cruz, CA). Antibodies against phospho-Y591-FLT3 (CS3461), eIF4B (CS3592), phospho-S422-eIF4BP (CS3591), phospho-S406-eIF4BP (CS8151), phospho-Y694-STAT5 (CS9359), phospho-T37/46-4EBP1 (CS2855), phospho-S65-4EBP1 (CS9451), phospho-T389-p70S6K (CS9234), phospho-T308-AKT (CS9275), phospho-S473-AKT (CS13038), phospho-S217/221-MEK (CS-9121), phospho-T202/Y204-ERK (CS-9106), Pim-2 (CS4730), phospho-S235/S236-S6RP (CS4858), phospho-S240/S244-S6RP (CS5364), phospho-S227-RSK2 (CS3556), phospho-S380-RSK (CS9335), phospho-T359/S363-RSK (CS9344), phospho-Y452-Gab2 (CS3882), phospho-T246-PRAS40 (CS2997), cleaved Caspase-3 (CS-9661), phospho-S241-PDK1 (CS3438), phospho-S/T-PKA substrates (CS9621), phospho-S21/9-GSK3α/ß (CS8566), phospho-S69-Bim (CS4585), phospho-S112-Bad (CS5284), phospho-S136-Bad(CS4366), Bim (CS2933), mTOR (CS2983), p85 (CS4257), Mcl-1(CS5453), and c-Myc (CS5605) were purchased from Cell Signaling (Beverly, MA, USA). 

### 4.2. Expression Plasmids, Transfection, and Infection

A retroviral expression plasmid for HA-RSK1, pMXs-IG-HA-RSK1, was constructed by subcloning the SalI/KpnI (blunted) fragment from pKH3-human RSK1, a gift from John Blenis (Addgene plasmid # 13841) [61], into the XhoI/NotI (blunted) site of the pMXs-IG vector, a gift from Toshio Kitamura. To obtain MV4-11 cells overexpressing RSK1, MV4-11 cells were infected with the recombinant retrovirus obtained from PLAT-A transfected with pMXs-IG-HA-RSK1 and sorted for GFP expression using BD FACSAria (BD Biosciences, San Jose, CA, USA), essentially as described previously [56]. 

To obtain MV4-11 cells knocked out of RSK1 or RSK2, MV4-11 cells were first infected with the recombinant lentivirus obtained from 293T cells transfected with lentiCas9-EGFP, a gift from Phil Sharp and Feng Zhang (Addgene plasmid # 63592) [62], along with psPAX2 and pMD2.G, as described previously [12]. Single cells were then sorted for GFP expression by flow cytometry. An isolated clone expressing GFP at a high level was then transduced with the lentiviral gRNA plasmid targeting RSK1 (RPS6KA1 gRNA: BRDN0001148481) or RSK2 (RPS6KA3 gRNA: BRDN0001144943) or with non-targeting control gRNA (BRDN0001148862), gifts from John Doench and David Root (Addgene plasmid #75499, 75942, and 80263) [63], with the same infection method. After selection with puromycin, KO of RSK1 or RSK2 was confirmed by immunoblot analysis.

To obtain MV4-11 cells knocked down of RSK1 or RSK2, MV4-11 cells were transduced with the MISSION lentiviral shRNA vector targeting RSK1, pLKO.1-RSK1 (TRCN1385), alone or along with that targeting RSK2, pLKO.1-RSK2 (TRCN194851), purchased from Sigma-Aldrich. The lentiviral vector targeting GFP for knockdown, pLKO.1-puro-GFP-siRNA, was a gift from Bob Weinberg (Addgene plasmid #12273) [64] and used as a control vector. Cells were selected with puromycin, and KD or RSK1 or RSK2 was confirmed by immunoblot analysis. For RSK1/RSK2 double KD cells, several clones were isolated by flow cytometry, and the clone expressing the lowest levels of RSK1 and RSK2 was used for analysis.

### 4.3. Analyses of Cell Proliferation and Apoptosis

Cell proliferation was assessed by using a Cell counting kit-8 (CCK-8) (Dojindo molecular technologies, Kumamoto, Japan) after cultured for indicated times in complete medium, according to the manufacturer’s instructions, and analyzed by SoftMax Pro software (Molecular devices). Flow cytometric analyses for cell cycle and apoptosis, activation of Bax, and cleavage of Caspase-3 were performed essentially as described previously [13,65] after treating cells under indicated conditions in serum-free ASF104 medium (Ajinomoto, Tokyo, Japan). An unpaired two-tailed Student’s *t*-test was used to calculate differences between means; differences were considered significant when *p* < 0.05. All the data shown are representative of experiments repeated at least three times. 

### 4.4. Immunoprecipitation and Immunoblotting

For immunoprecipitation and immunoblotting experiments, cells were treated under indicated conditions in serum-free ASF104 medium or in culture medium without FCS, and analyzed essentially as described previously [55]. All the data shown are representative of experiments repeated at least three times. 

### 4.5. Analyses of Primary AML Cells

Peripheral blood mononuclear cells containing 96% leukemic cells were isolated from a patient with AML with FLT3-ITD allelic ratio of 9.08 and subjected to immunoblot analysis and viability assays as described previously [13]. In brief, cell proliferation was assessed after culturing cells under indicated conditions in IMDM medium containing 10% FCS by counting viable cell numbers by the trypan blue-dye exclusion method. All the data shown using primary AML cells are representative of two repeated experiments, except for those shown in Figure 5B, which were from a single experiment. The study was approved by the ethical committee of Tokyo Medical and Dental University. Written informed consent was obtained from the patient, in compliance with the Declaration of Helsinki.

## 5. Conclusions

We have shown for the first time that the FLT3-ITD, but not BCR/ABL, activates RSK to transduce signals required for proliferation and survival of leukemic cells. We have further revealed that activation of not only the MEK/ERK pathway, but also PDK1 should be involved in the RSK activation by FLT3-ITD, while activation of the MEK/ERK pathway by FLT3-ITD and its negative feedback regulation by RSK were mediated by Gab2/SHP2 interaction. We have further elucidated the molecular mechanisms by which mainly RSK1 activates the mTORC1/S6K/4EBP1 and eIF4B in cooperation with the STAT5/PIM and PI3K/AKT pathways downstream of FLT3/ITD, and downregulates the pro-apoptotic Bcl-2 family members Bad and BIM to promote proliferation and prevent apoptosis in AML cells. Thus, the present study has elucidated the important differences of signaling mechanisms from the two most important leukemogenic tyrosine kinase mutants, and proposes RSK1 as a promising target, particularly in combination with PIM or PI3K, as well as the anti-apoptotic Bcl-2 family members, for novel therapeutic strategies against therapy-resistant FLT3-ITD-positive AML.

## Supplementary Materials

The following are available online at https://www.mdpi.com/2072-6694/11/12/1827/s1: Figure S1. Effects of RSK inhibitors on proliferation of cells transformed by FLT3-ITD, BCR/ABL, or JAK2-V617F; Figure S2. RSK1 negatively regulates Bad and BIM-EL and activates the mTOR pathways and eIF4B cooperatively with PIM in FLT3-ITD-positive AML cells; Figure S3. Inhibition of RSK and PIM or PI3K cooperatively activates the intrinsic pathway.
