# Supplementary material for: FLT3-ITD Activates RSK1 to Enhance Proliferation and Survival of AML Cells by Activating mTORC1 and eIF4B Cooperatively with PIM or PI3K and by Inhibiting Bad and BIM
Source: Cancers (Basel). 2019 Nov 20;11(12):1827. doi: 10.3390/cancers11121827 (PMC6966435; doi:10.3390/cancers11121827)
Supplement: Supplementary file 1 [file cancers-11-01827-s001.zip › cancers-637799-Supplementary-Figures.pdf]

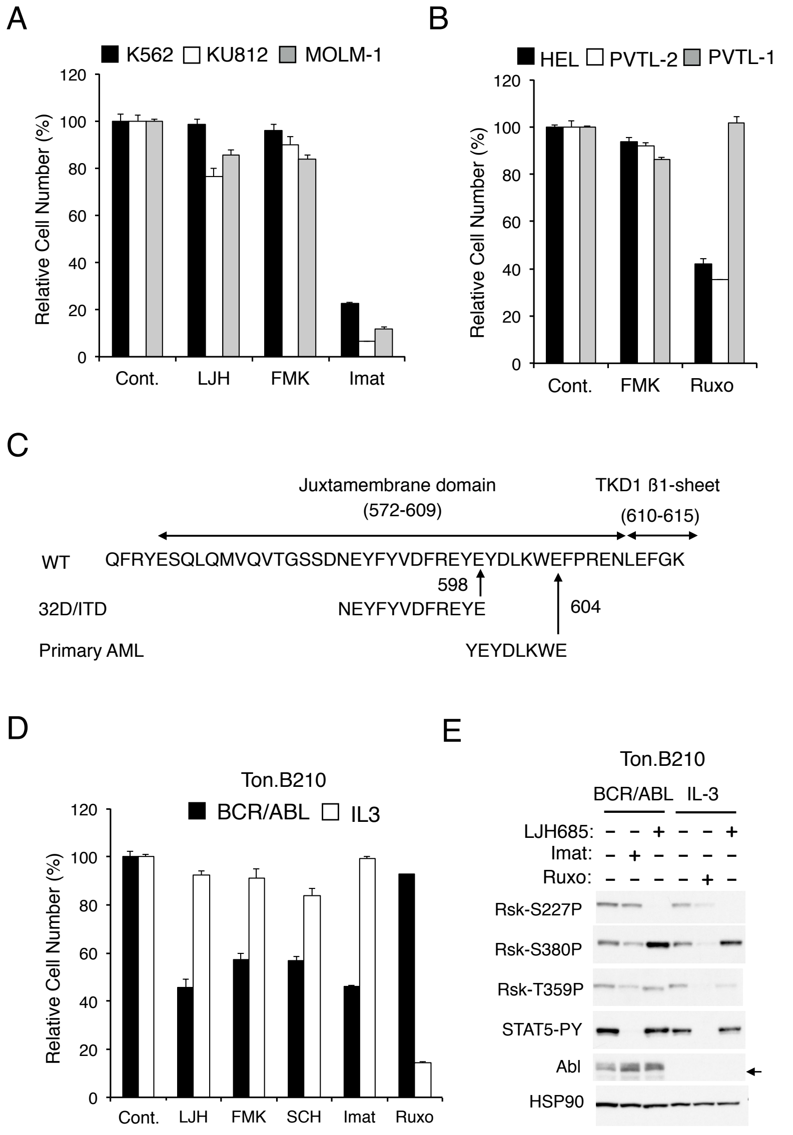

Figure S1. Effects of RSK inhibitors on proliferation of cells transformed by FLT3-ITD, BCR/ABL, or JAK2-V617F. (A) K562, KU812, or MOLM-1 cells were cultured for 48 h in the absence (Cont.) or presence of 5 μM LJH685 (LJH), 2.5 μM FMK, or 1 μM imatinib (Imat), as indicated. Viable cell numbers were measured by the colorimetric assay. Each column represents the mean of triplicate cultures, with error bars indicating standard errors, and is expressed as a ratio to the control cell numbers. (B) HEL, PVTL-2, or PVTL-2 cells were cultured for 48 h in the absence (Cont.) or presence of 5 μM FMK, or 1 μM ruxolitinib (Ruxo), as indicated, and analyzed. (C) Partial amino acid sequences of wild-type FLT3 (WT) and inserted amino acid sequences of FLT3-ITD expressed in 32D/ITD or in primary AML cells analyzed in the present study. The insertion sites are indicated by arrows and amino-acid numbers. (D) Ton.B210 cells inducibly expressing BCR/ABL (BCR/ABL) and cultured without IL-3 or cultured with IL-3 (IL-3) without expressing BCR/ABL were cultured for 48 h with 10 μM LJH685, 5 μM FMK, 0.5 μM SCH772984 (SCH), 1 μM imatinib, or 1 mM ruxolitinib, as, indicated, and analyzed. (E) Ton.B210 cells inducibly expressing BCR/ABL (BCR/ABL) and cultured without IL-3 or cultured with IL-3 (IL-3) without expressing BCR/ABL treated for 6 h with 5 μM LJH685, 3 μM imatinib, or 3 μM ruxolitinib, as indicated. Cells were then lysed and subjected to immunoblot analysis with antibodies against indicated proteins. HSP90 was used for a loading control. An arrow indicates the position of BCR/ABL. Abbreviations: RSK-S227P, phospho-S227-RSK2; RSK-S380P, phospho-S380-RSK1; RSK-T359P, phospho-T359/S363-RSK1; STAT5-PY, phospho-Y694-STAT5.

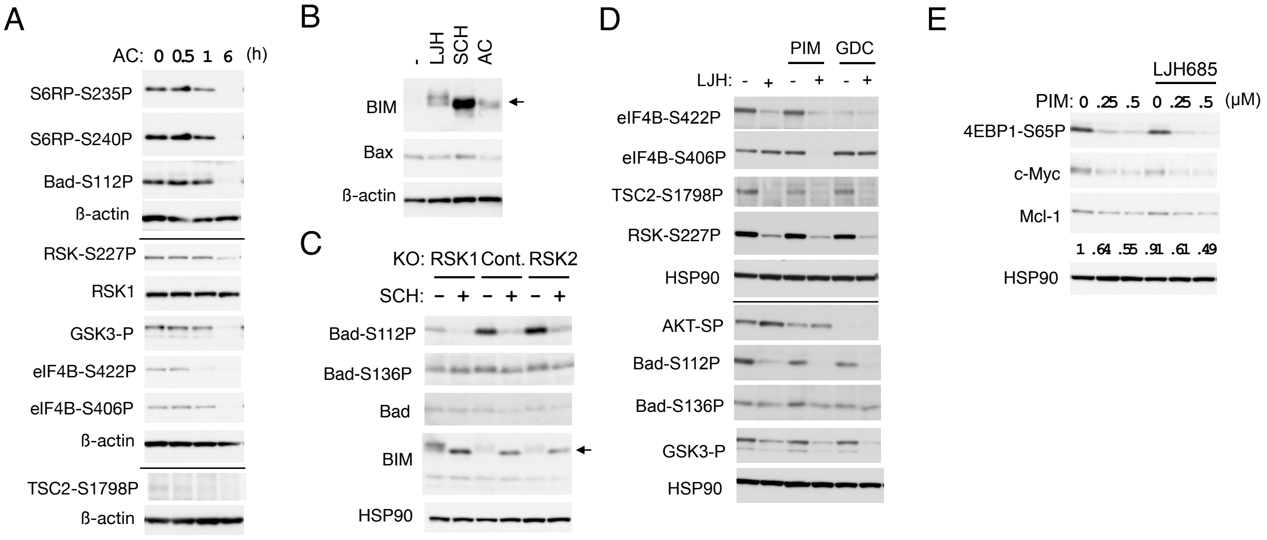

Figure S2. RSK1 negatively regulates Bad and BIM-EL and activates the mTOR pathways and eIF4B cooperatively with PIM in FLT3-ITD-positive AML cells. (A) MV4-11 cells were treated with 10 nM quizartinib (AC) for indicated times. Cells were then lysed and subjected to immunoblot analysis with antibodies against indicated proteins. ß-actin was used for a loading control. The results obtained from triplicate gels are distinguished by horizontal lines. Abbreviations: S6RP-S235P, phospho-S235/S236-S6RP; S6RP-S240P, phospho-S240/S244-S6RP; Bad-S112P, phospho-S112-Bad; GSK3-P, phospho-S21/9-GSK3a/b; eIF4B-S422P, phospho-S422-eIF4B; eIF4B-S406P, phospho-S406-eIF4B; TSC2-S1798P, phospho-S1798-TSC2. (B) MV4-11 cells were left untreated as control (-) or treated for 16 h with 5 µM LJH685 (LJH), 0.5 µM SCH772984 (SCH), or 10 nM quizartinib, as indicated, and analyzed. An arrow indicates the position of BIM-EL. (C) MV4-11 cells knocked out (KO) of RSK1 or RSK2 as well as vector control cells (Cont.), as indicated, were treated for 6 h with or without 0.5 µM SCH772984, as indicated and analyzed. Bad-S136P: phospho-S136-Bad. (D) MV4-11 cells were treated for 6 h with 5 µM LJH685, 1 µM PIM-447 (PIM), or 1 µM GDC-0941 (GDC), as indicated, and analyzed. The results obtained from duplicate gels are shown above or below a horizontal line. (E) MV4-11 cells were treated for 6 h with indicated concentrations of PIM-447 and 1 µM LJH685, as indicated and analyzed. Relative expression levels of Mcl-1 were determined by densitometric analysis and are shown below the panel. 4EBP1-S65P: phospho-S65-4EBP1.

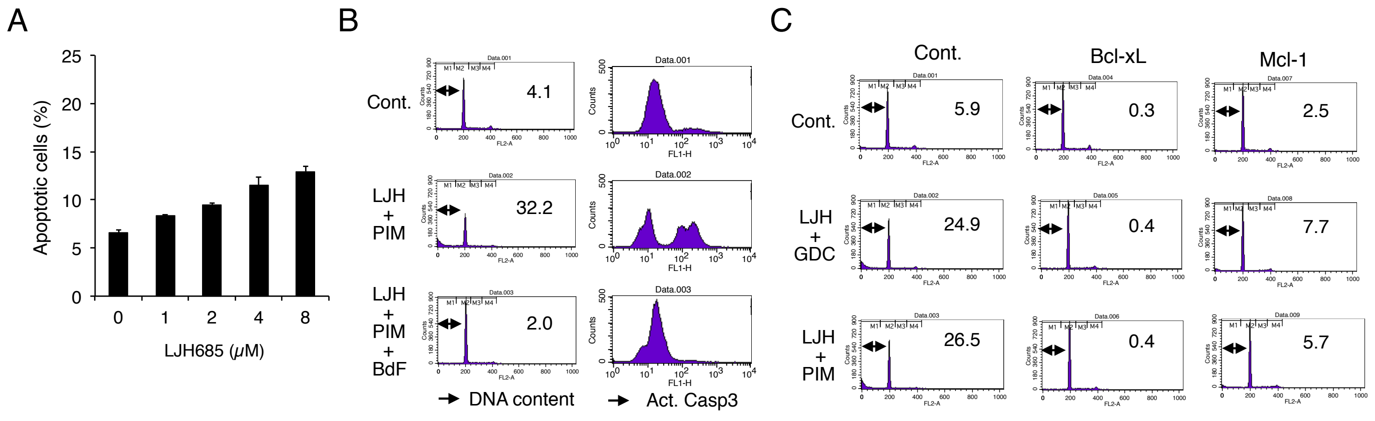

Figure S3. Inhibition of RSK and PIM or PI3K cooperatively activates the intrinsic pathway of apoptosis in FLT3-ITD-positive AML cells. (A) MV4-11 cells were treated with indicated concentrations of LJH685 for 24 h and analyzed for the cellular DNA content by flow cytometry. The means of percentages of apoptotic cells with sub-G1 DNA content from triplicate measurements are plotted. (B) MV4-11 cells were treated for 16 h with 5 µM LJH685 (LJH), 3 µM PIM-447 (PIM), or 100 µM Boc-d-FMK (BdF), as indicated, and analyzed for cellular DNA content or Caspase-3 activation by flow cytometry. Percentages of apoptotic cells with sub-G1 DNA content are indicated. (C) MV4-11 cells overexpressing Bcl-XL (Bcl-XL) or Mcl-1 (Mcl-1) as well as vector control cells (Cont.) were left untreated as control (Cont.) or treated with 5 µM LJH685, 3 µM GDC-0941 (GDC), or 3 µM PIM-447, as indicated, for 16 h, and analyzed.
