# Supplementary material for: FLT3-ITD Activates RSK1 to Enhance Proliferation and Survival of AML Cells by Activating mTORC1 and eIF4B Cooperatively with PIM or PI3K and by Inhibiting Bad and BIM
Source: Cancers (Basel). 2019 Nov 20;11(12):1827. doi: 10.3390/cancers11121827 (PMC6966435; doi:10.3390/cancers11121827)
Supplement: Supplementary file 1 [file cancers-11-01827-s001.zip › cancers-637799-western blot.pdf]

Figure 1B

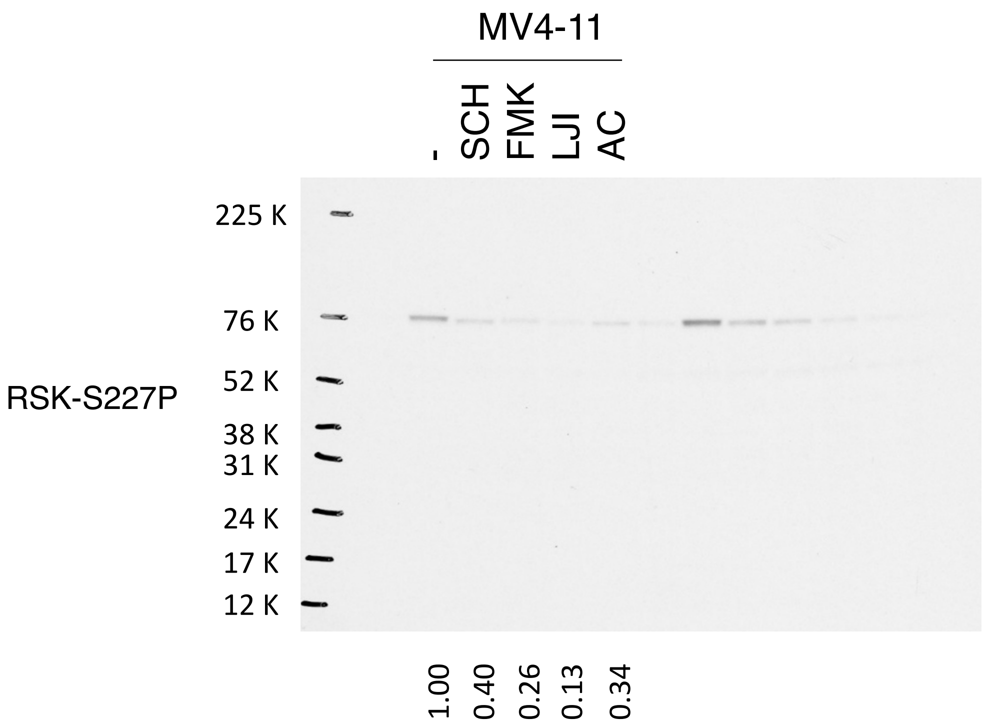
MV4-11
-
SCH
FMK
LJI
AC
225 K
76 K
52 K
38 K
31 K
24 K
17 K
12 K
RSK-S227P
1.00
0.40
0.26
0.13
0.34

Figure 1B

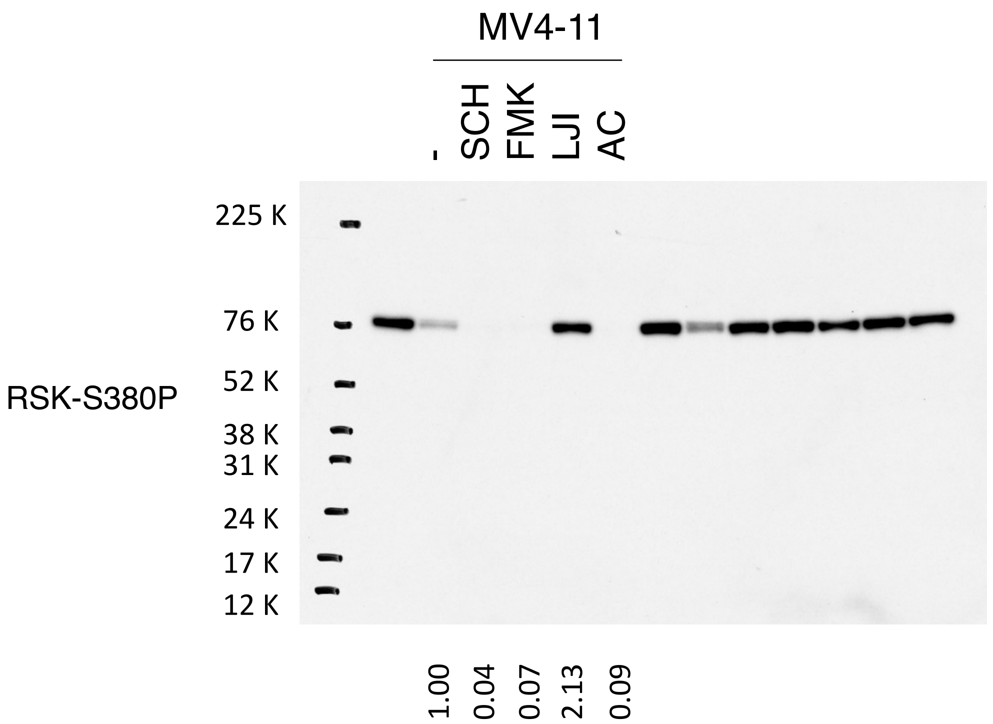
MV4-11
-
SCH
FMK
LJI
AC
225 K
76 K
52 K
38 K
31 K
24 K
17 K
12 K
RSK-S380P
1.00
0.04
0.07
2.13
0.09

Figure 1B

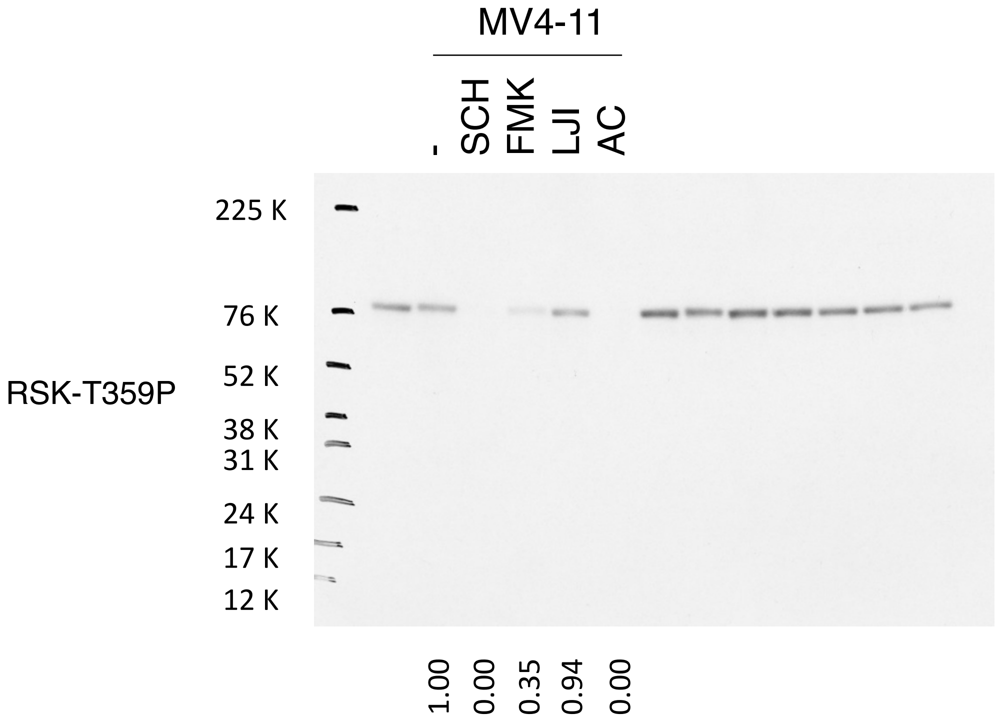
MV4-11
-
SCH
FMK
LJI
AC
225 K
76 K
52 K
38 K
31 K
24 K
17 K
12 K
RSK-T359P
1.00
0.00
0.35
0.94
0.00

Figure 1B

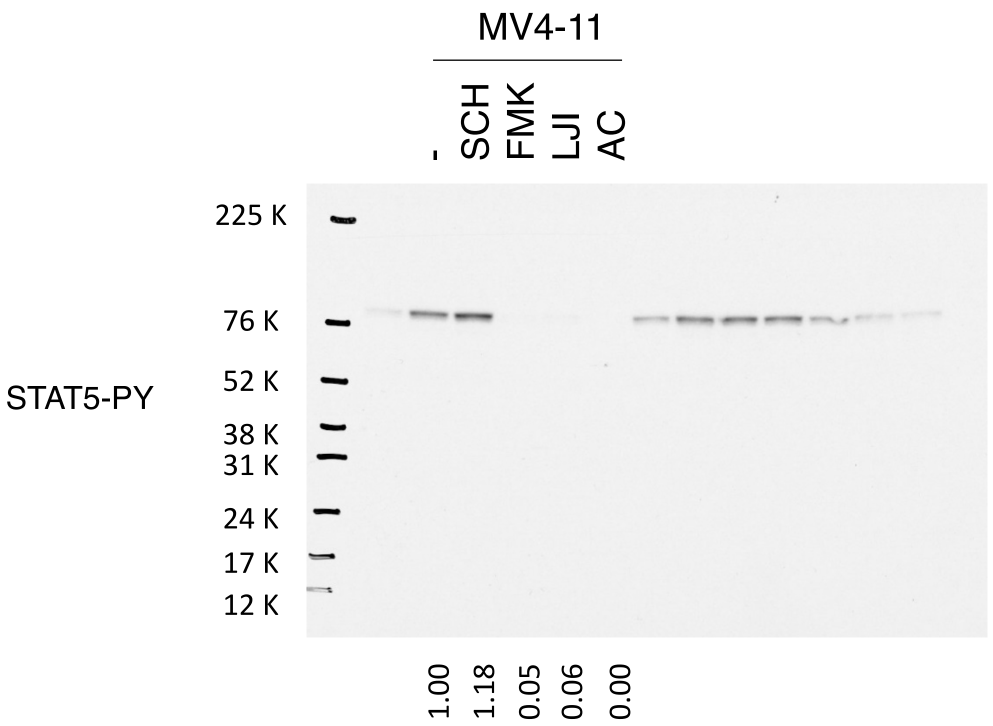
MV4-11
-
SCH
FMK
LJI
AC
225 K
76 K
52 K
38 K
31 K
24 K
17 K
12 K
STAT5-PY
1.00
1.18
0.05
0.06
0.00

Figure 1B

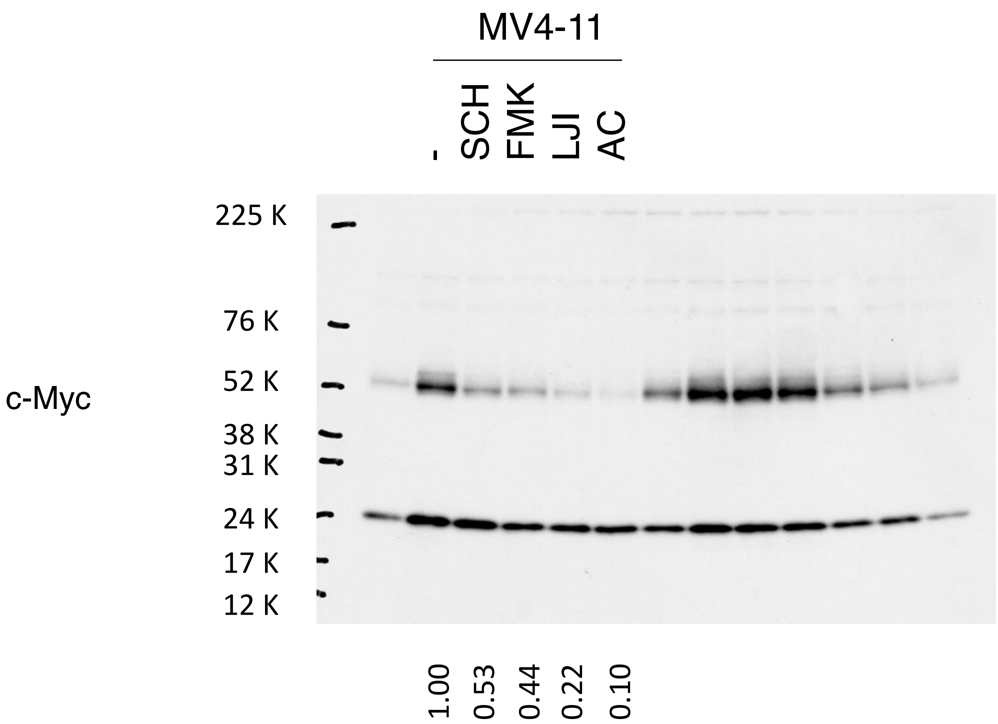
MV4-11
-
SCH
FMK
LJI
AC
225 K
76 K
52 K
38 K
31 K
24 K
17 K
12 K
c-Myc
1.00
0.53
0.44
0.22
0.10

Figure 1B

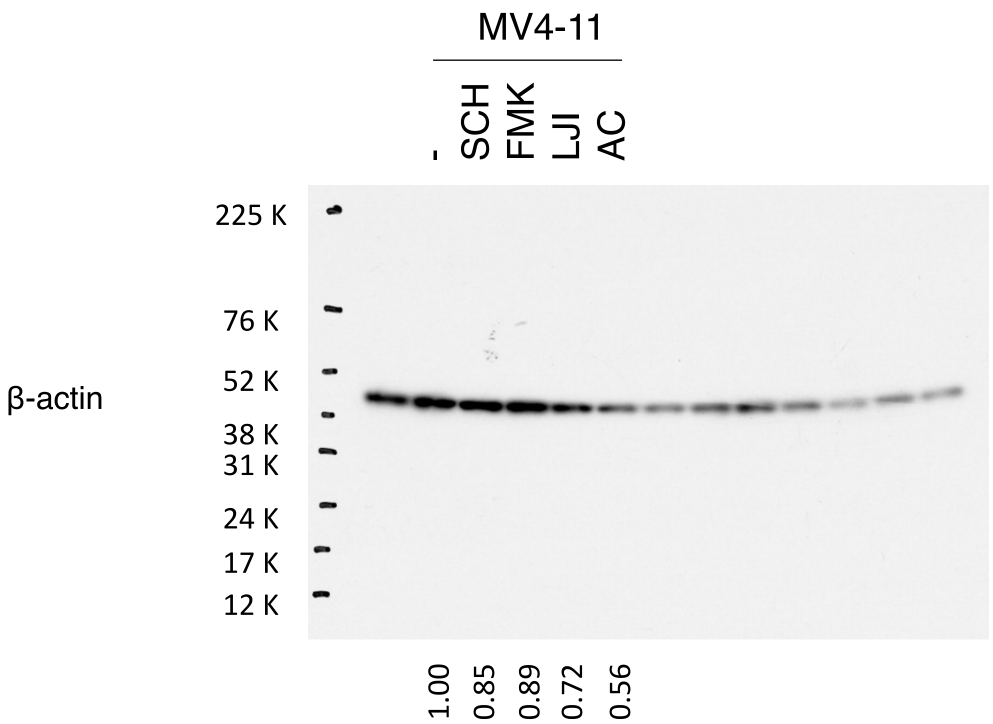
MV4-11
-
SCH
FMK
LJI
AC
225 K
76 K
52 K
38 K
31 K
24 K
17 K
12 K
β-actin
1.00
0.85
0.89
0.72
0.56

Figure 1B

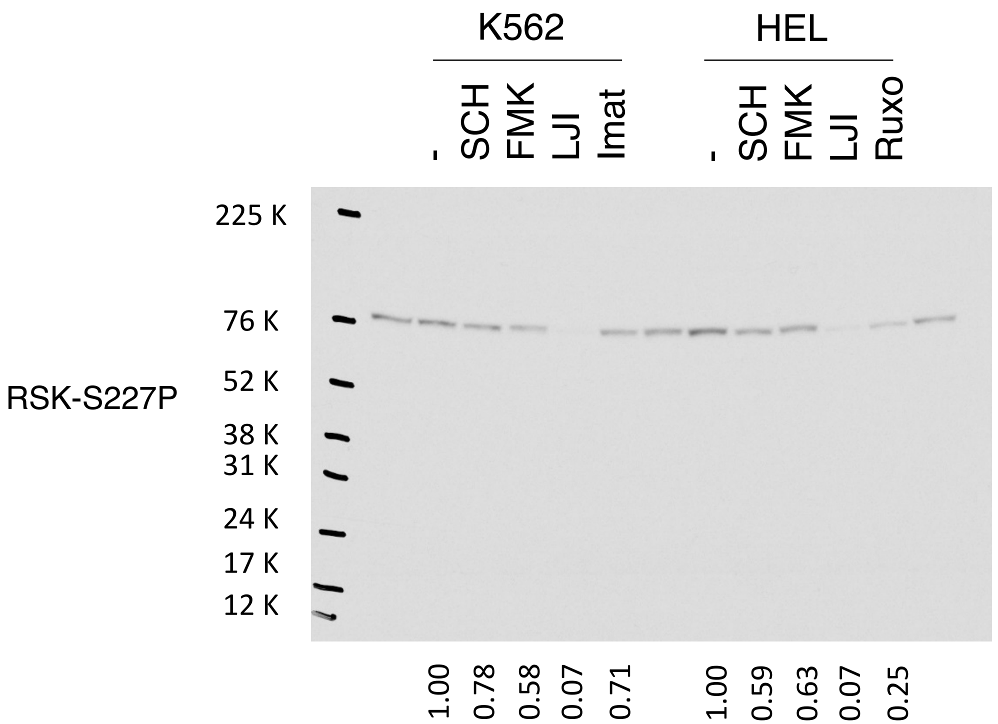
K562
HEL
-
SCH
FMK
LJI
Imat
-
SCH
FMK
LJI
Ruxo
225 K
76 K
52 K
38 K
31 K
24 K
17 K
12 K
RSK-S227P
1.00
0.78
0.58
0.07
0.71
1.00
0.59
0.63
0.07
0.25

Figure 1B

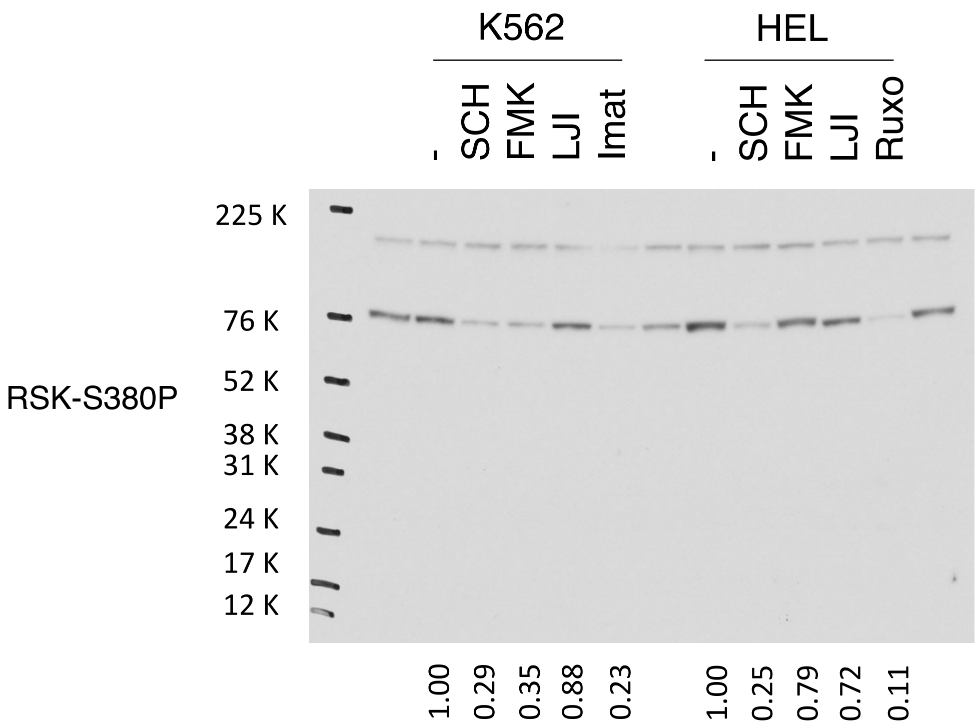
K562
HEL
-
SCH
FMK
LJI
Imat
-
SCH
FMK
LJI
Ruxo
225 K
76 K
52 K
38 K
31 K
24 K
17 K
12 K
RSK-S380P
1.00
0.29
0.35
0.88
0.23
1.00
0.25
0.79
0.72
0.11

Figure 1B

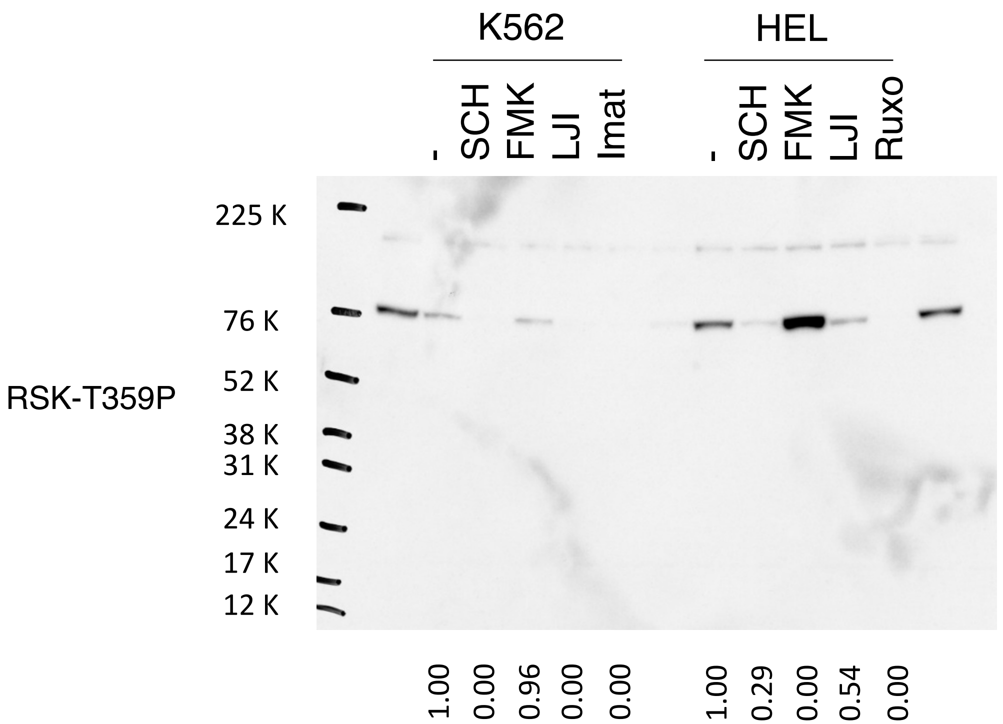
K562
HEL
-
SCH
FMK
LJI
Imat
-
SCH
FMK
LJI
Ruxo
225 K
76 K
52 K
38 K
31 K
24 K
17 K
12 K
RSK-T359P
1.00
0.00
0.96
0.00
0.00
1.00
0.29
0.00
0.54
0.00

Figure 1B

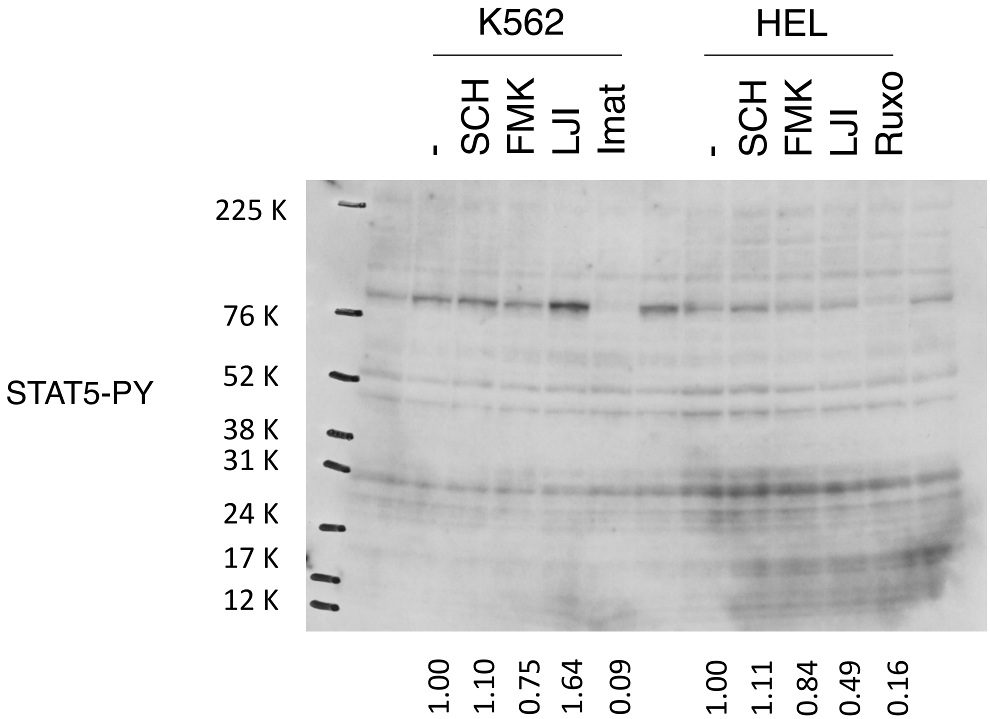
K562
HEL
-
SCH
FMK
LJI
Imat
-
SCH
FMK
LJI
Ruxo
225 K
76 K
52 K
38 K
31 K
24 K
17 K
12 K
STAT5-PY
1.00
1.10
0.75
1.64
0.09
1.00
1.11
0.84
0.49
0.16

Figure 1B

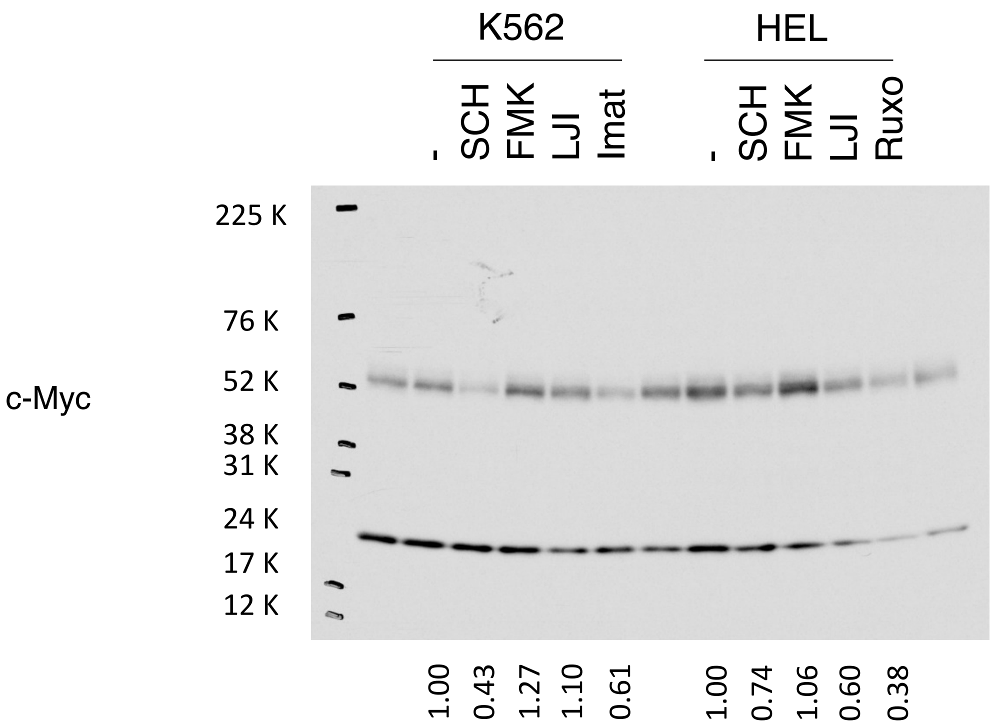
K562
HEL
-
SCH
FMK
LJI
Imat
-
SCH
FMK
LJI
Ruxo
225 K
76 K
52 K
38 K
31 K
24 K
17 K
12 K
c-Myc
1.00
0.43
1.27
1.10
0.61
1.00
0.74
1.06
0.60
0.38

Figure 1B

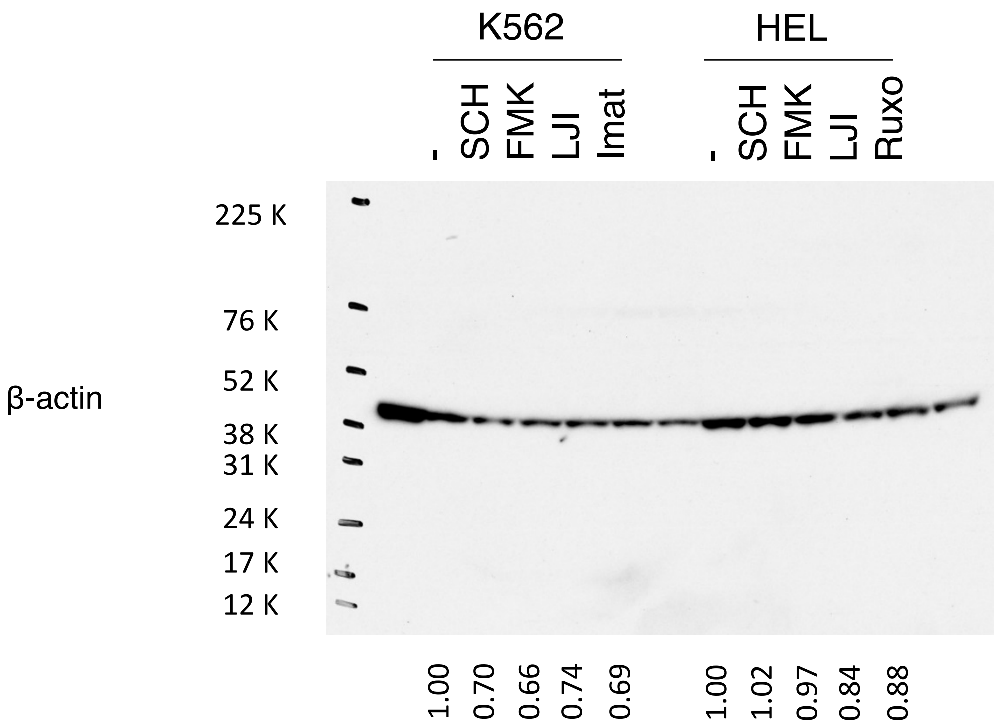

K562
HEL
-
SCH
FMK
LJI
Imat
-
SCH
FMK
LJI
Ruxo
225 K
76 K
52 K
38 K
31 K
24 K
17 K
12 K
β-actin
1.00
0.70
0.66
0.74
0.69
1.00
1.02
0.97
0.84
0.88

Figure 1C

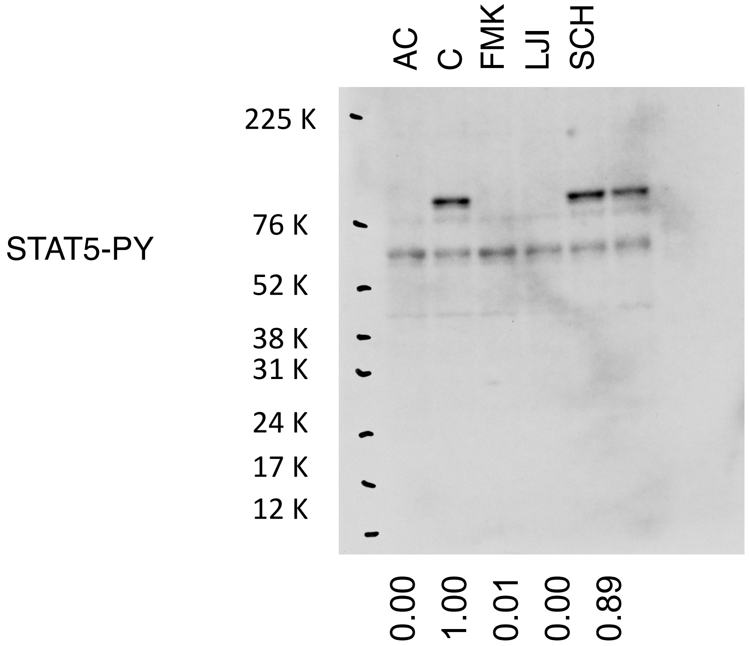

STAT5-PY
AC
C
FMK
LJI
SCH
225 K
76 K
52 K
38 K
31 K
24 K
17 K
12 K
0.00
1.00
0.01
0.00
0.89

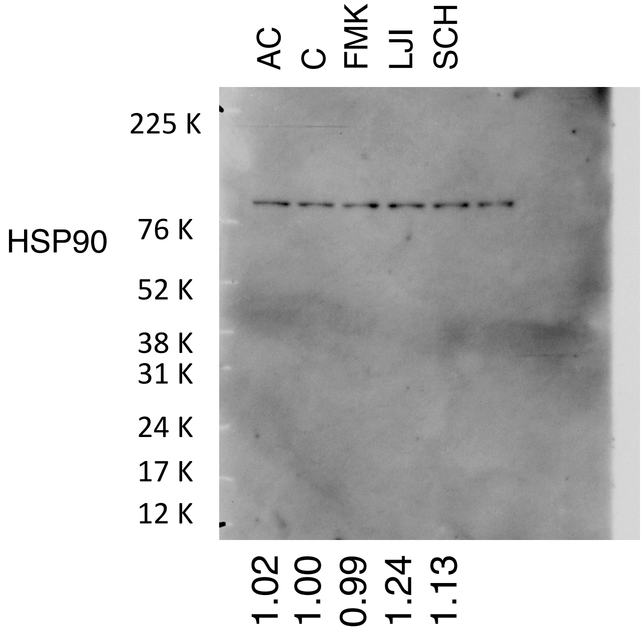

HSP90
AC
C
FMK
LJI
SCH
225 K
76 K
52 K
38 K
31 K
24 K
17 K
12 K
1.02
1.00
0.99
1.24
1.13

Figure 1D

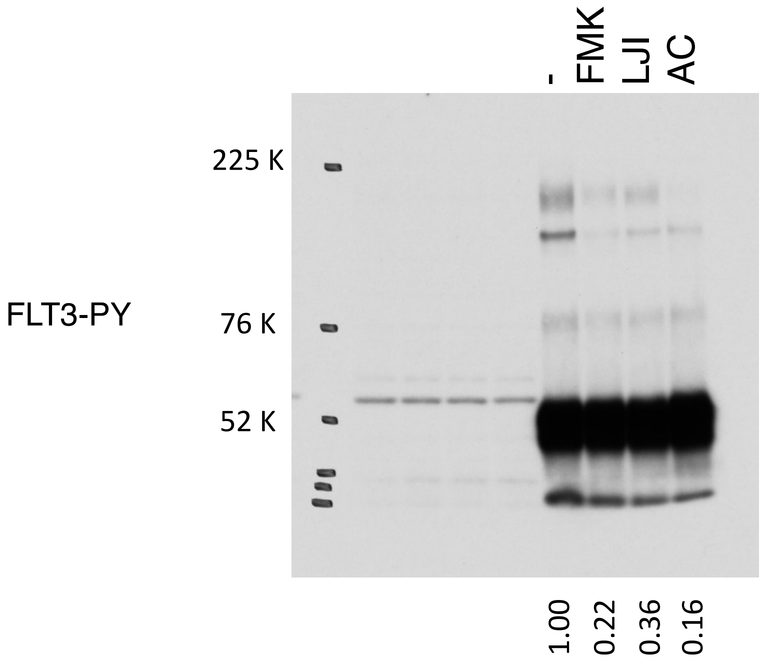

FLT3-PY
-
FMK
LJI
AC
225 K
76 K
52 K
1.00
0.22
0.36
0.16

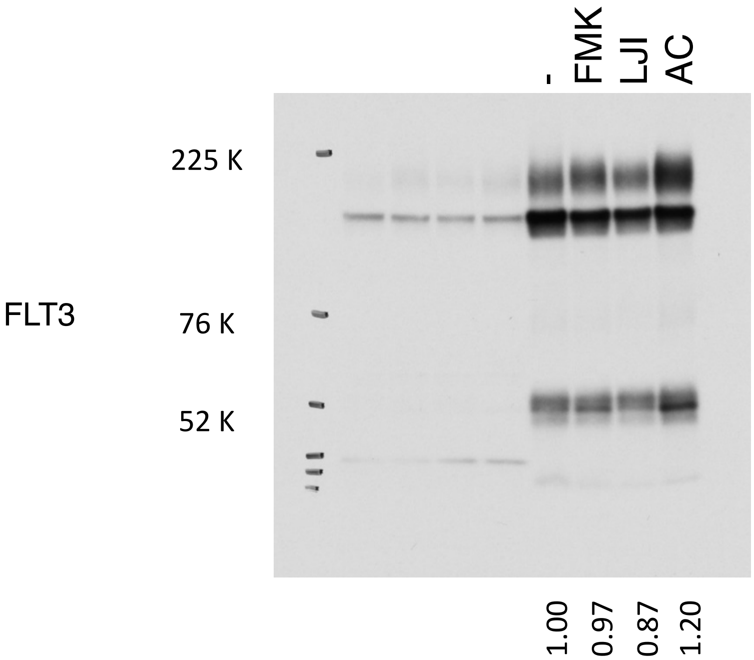

FLT3
-
FMK
LJI
AC
225 K
76 K
52 K
1.00
0.97
0.87
1.20

Figure 1E

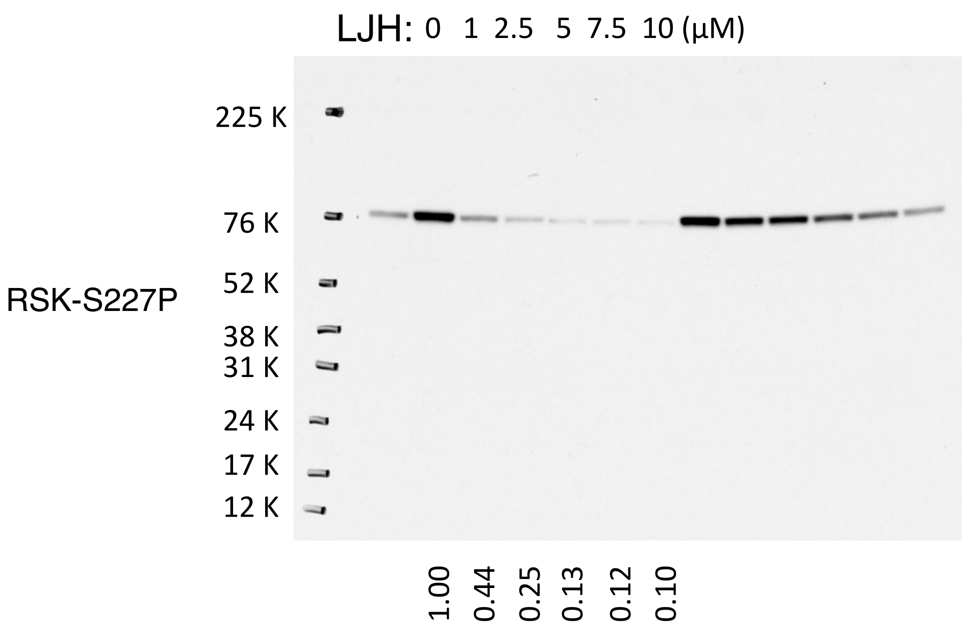
LJH: 0 1 2.5 5 7.5 10 (µM)
225 K
76 K
52 K
38 K
31 K
24 K
17 K
12 K
RSK-S227P
1.00
0.44
0.25
0.13
0.12
0.10

Figure 1E

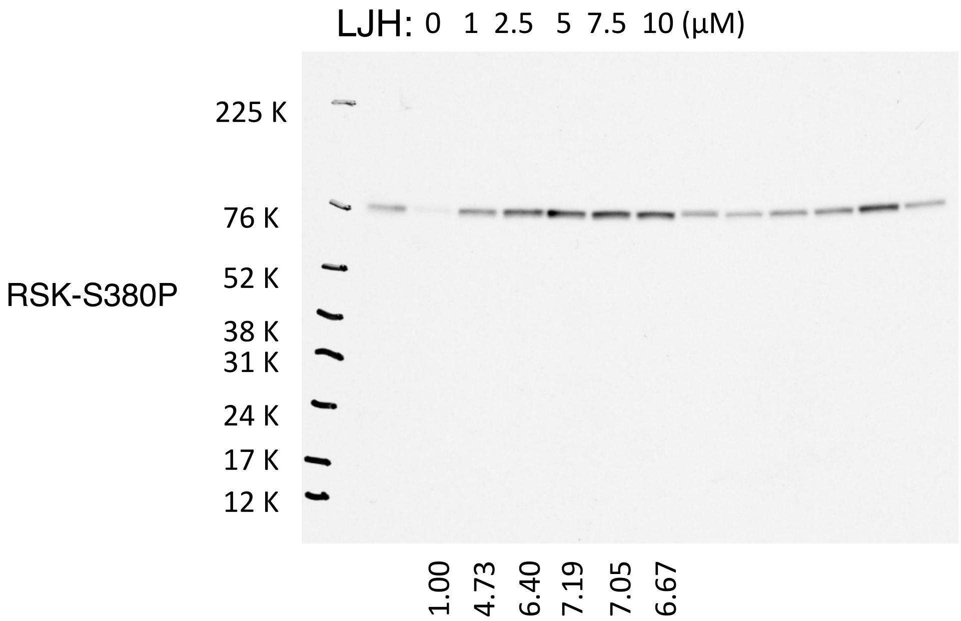
LJH: 0 1 2.5 5 7.5 10 (μM)
225 K
76 K
52 K
38 K
31 K
24 K
17 K
12 K
RSK-S380P
1.00
4.73
6.40
7.19
7.05
6.67

Figure 1E

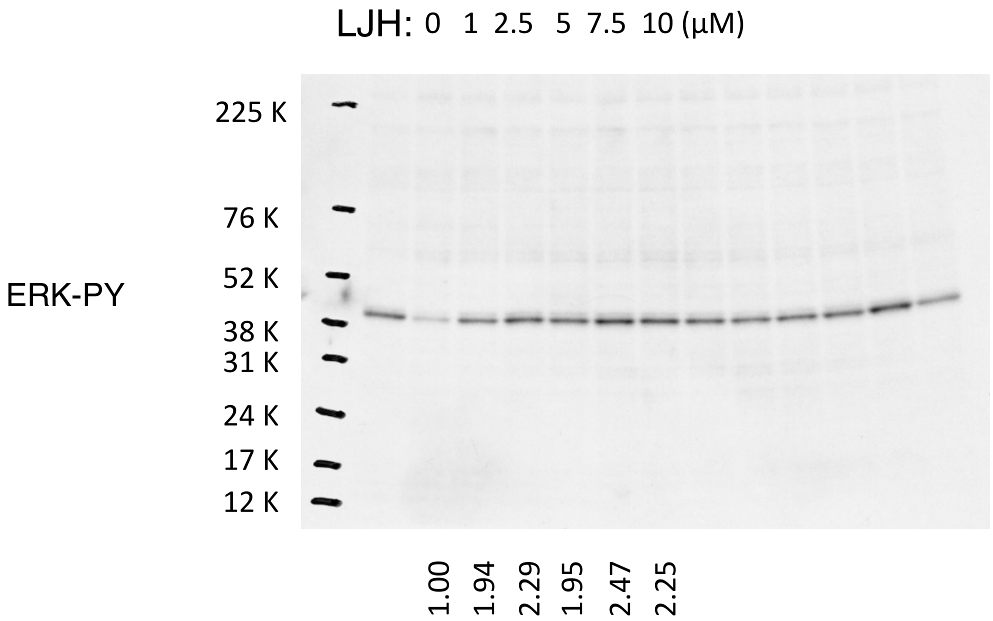
LJH: 0 1 2.5 5 7.5 10 (μM)
ERK-PY
225 K
76 K
52 K
38 K
31 K
24 K
17 K
12 K
1.00
1.94
2.29
1.95
2.47
2.25

Figure 1E

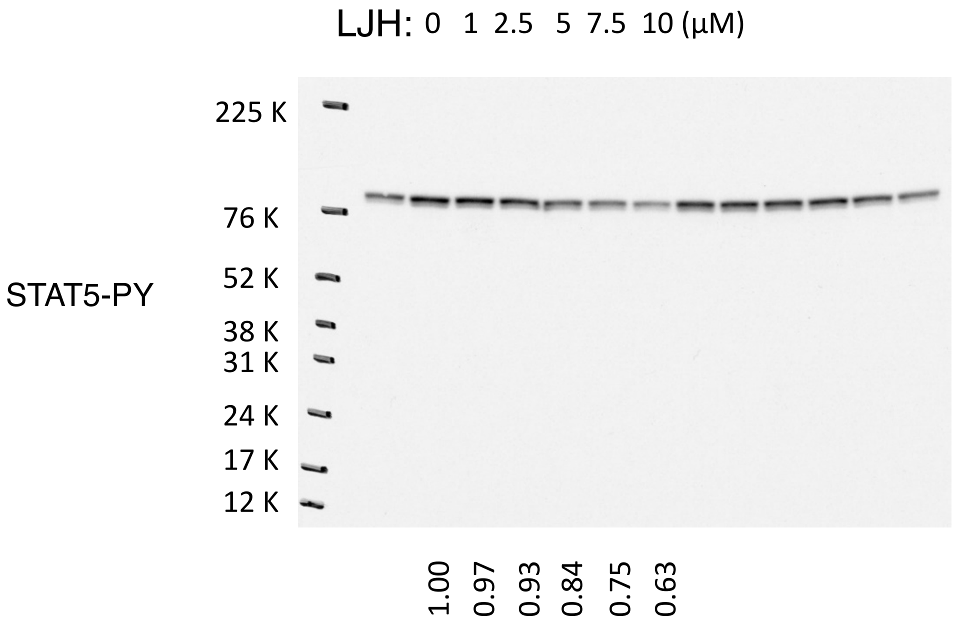
LJH: 0 1 2.5 5 7.5 10 (μM)
225 K
76 K
52 K
38 K
31 K
24 K
17 K
12 K
STAT5-PY
1.00
0.97
0.93
0.84
0.75
0.63

Figure 1E

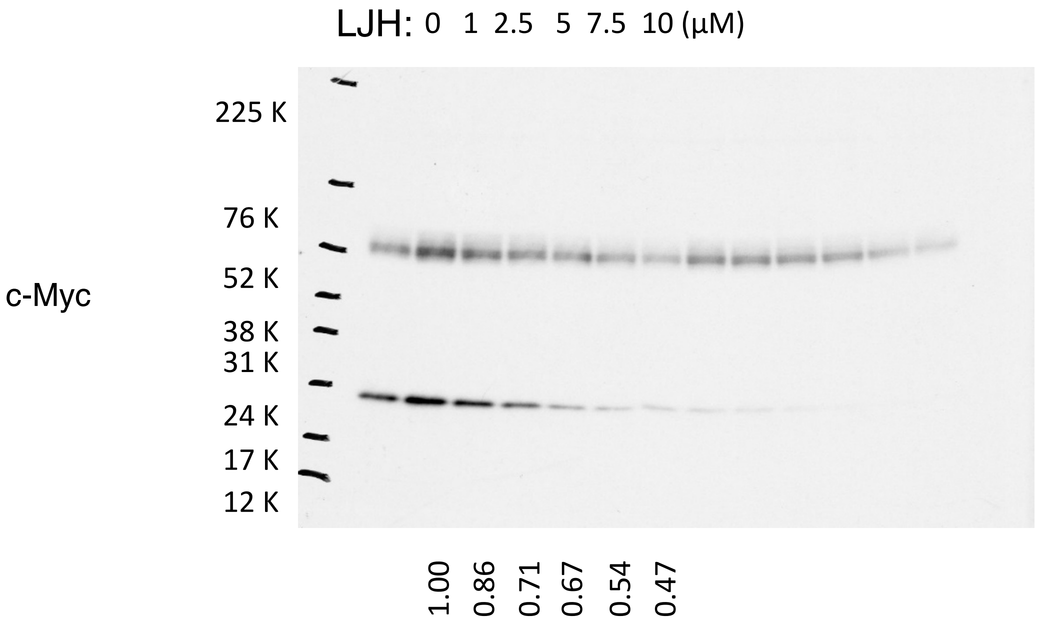

LJH: 0 1 2.5 5 7.5 10 (µM)
225 K
76 K
52 K
38 K
31 K
24 K
17 K
12 K
c-Myc
1.00
0.86
0.71
0.67
0.54
0.47

Figure 1E

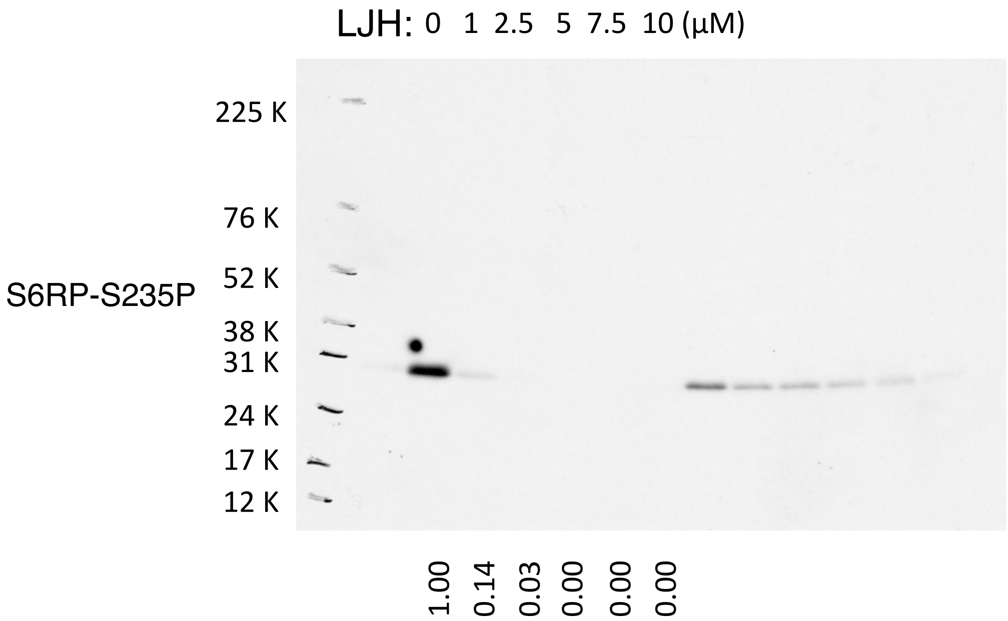

LJH: 0 1 2.5 5 7.5 10 (μM)
225 K
76 K
52 K
38 K
31 K
24 K
17 K
12 K
S6RP-S235P
1.00
0.14
0.03
0.00
0.00
0.00

Figure 1E

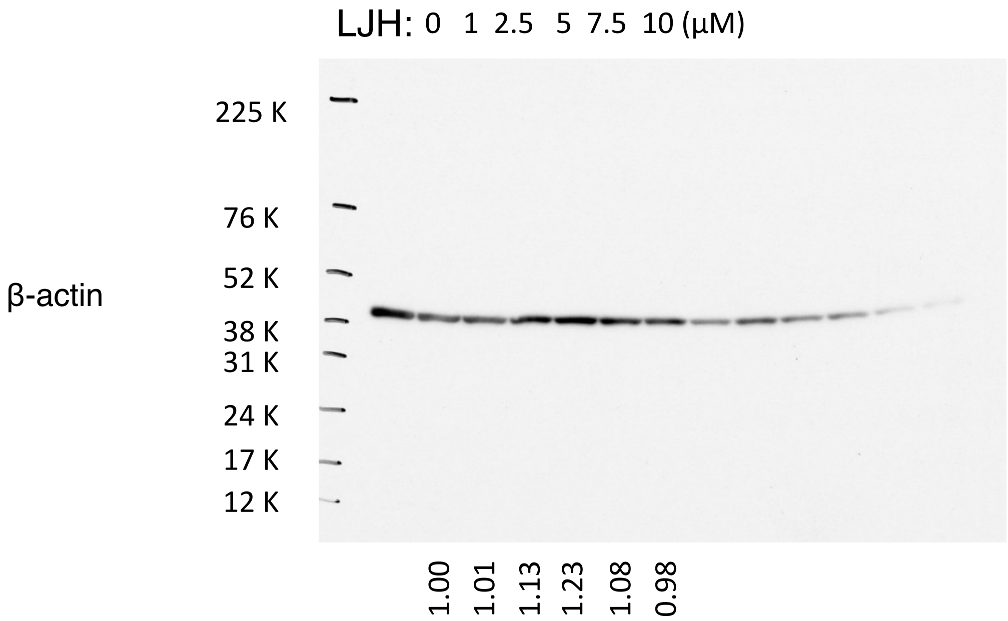
LJH: 0 1 2.5 5 7.5 10 (μM)
225 K
76 K
52 K
38 K
31 K
24 K
17 K
12 K
β-actin
1.00
1.01
1.13
1.23
1.08
0.98

Figure 2B

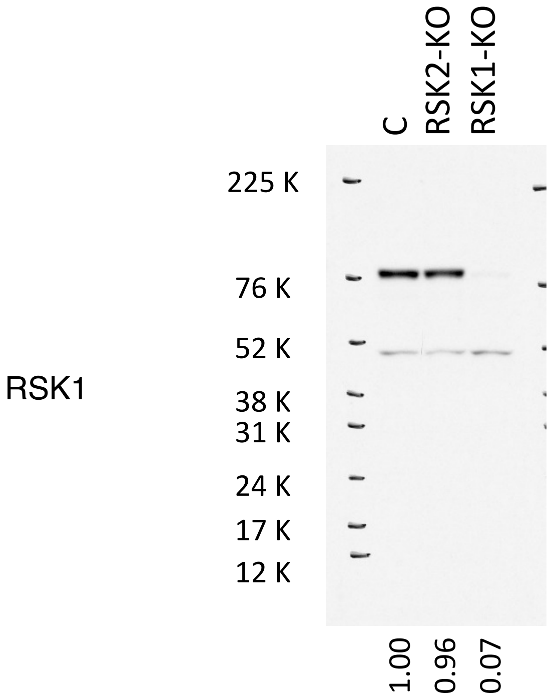
RSK1
C
RSK2-KO
RSK1-KO
225 K
76 K
52 K
38 K
31 K
24 K
17 K
12 K
1.00
0.96
0.07

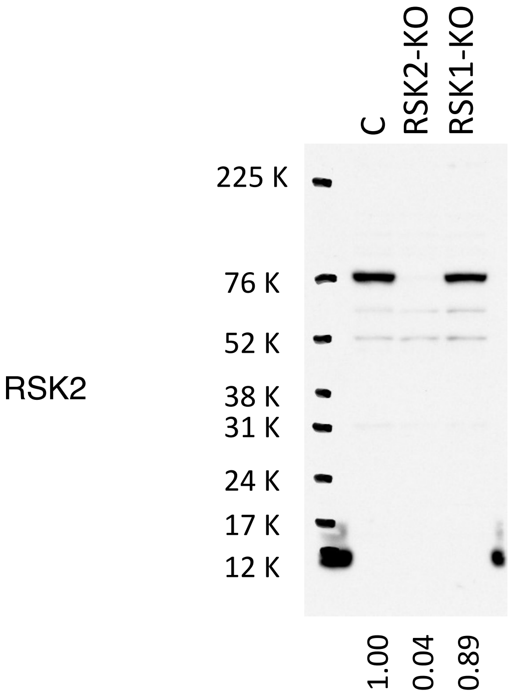
RSK2
C
RSK2-KO
RSK1-KO
225 K
76 K
52 K
38 K
31 K
24 K
17 K
12 K
1.00
0.04
0.89

Figure 2B

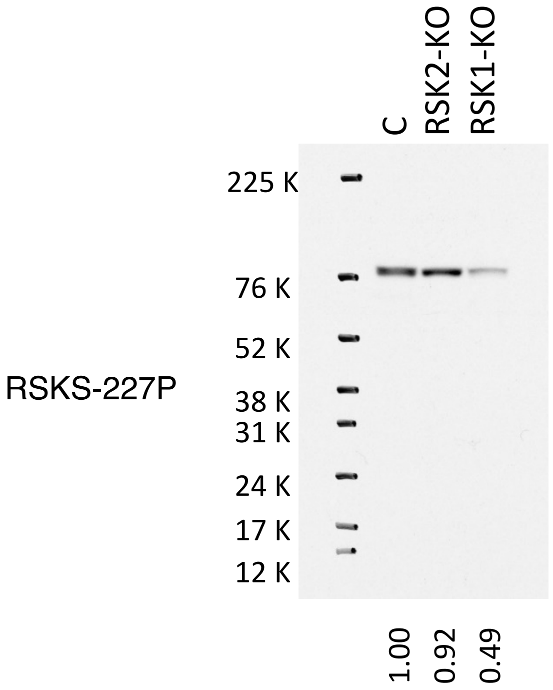

C
RSK2-KO
RSK1-KO
225 K
76 K
52 K
RSKS-227P
38 K
31 K
24 K
17 K
12 K
1.00
0.92
0.49

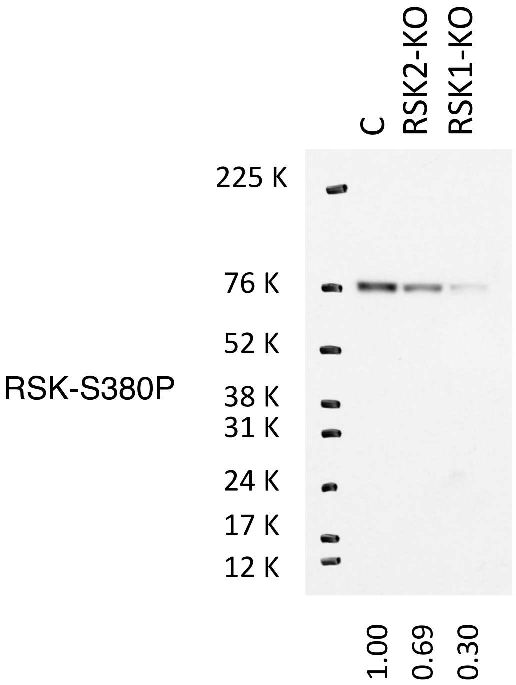

C
RSK2-KO
RSK1-KO
225 K
76 K
52 K
RSK-S380P
38 K
31 K
24 K
17 K
12 K
1.00
0.69
0.30

Figure 2B

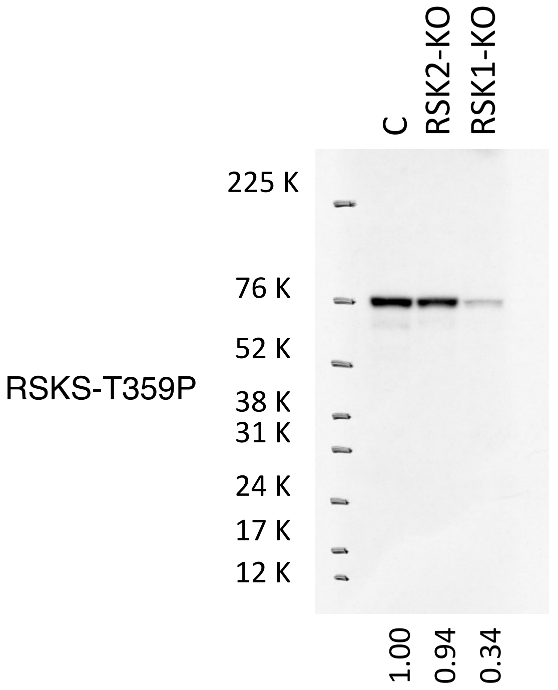
RSKS-T359P
C
RSK2-KO
RSK1-KO
225 K
76 K
52 K
38 K
31 K
24 K
17 K
12 K
1.00
0.94
0.34

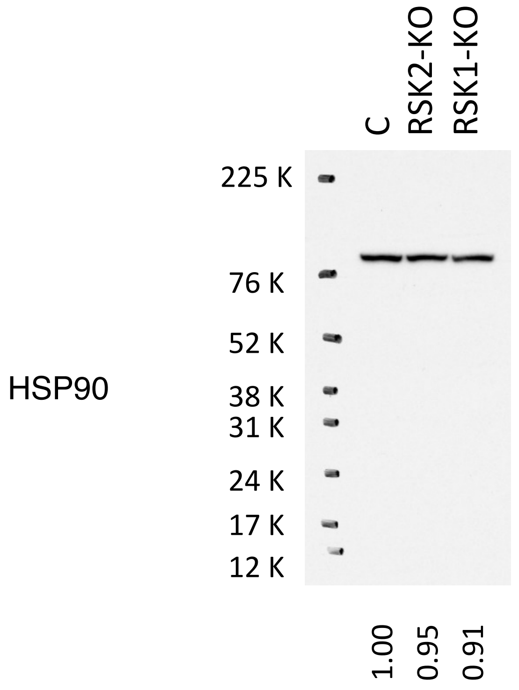
HSP90
C
RSK2-KO
RSK1-KO
225 K
76 K
52 K
38 K
31 K
24 K
17 K
12 K
1.00
0.95
0.91

Figure 2D

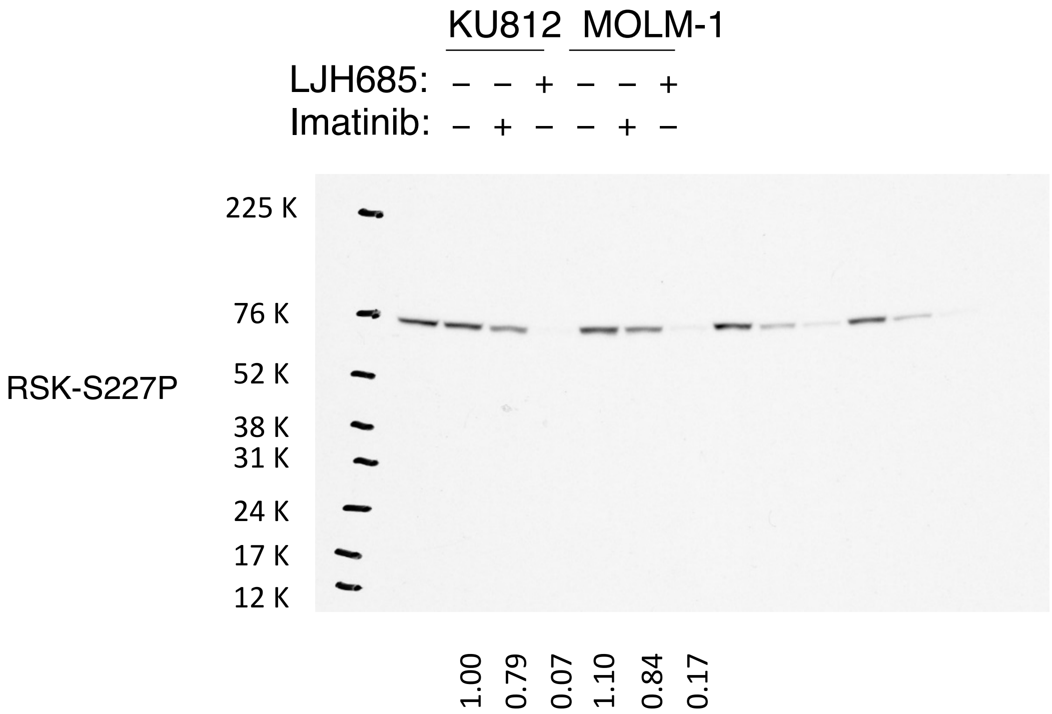
KU812
MOLM-1
LJH685: − − + − − +
Imatinib: − + − − + −
225 K
76 K
52 K
38 K
31 K
24 K
17 K
12 K
RSK-S227P
1.00
0.79
0.07
1.10
0.84
0.17

Figure 2D

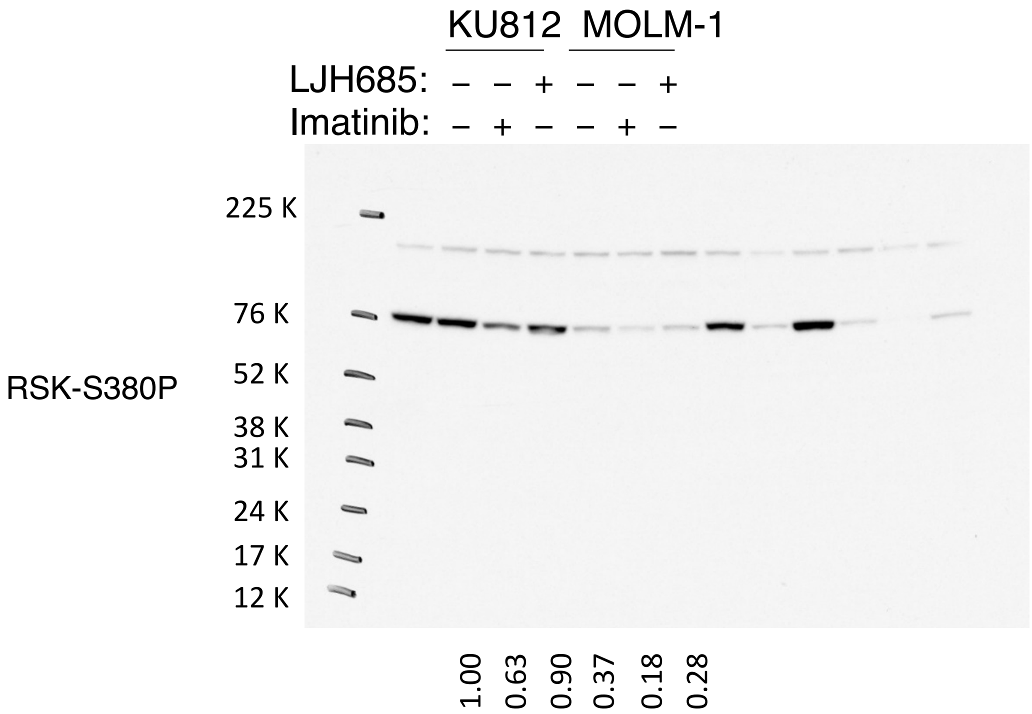
KU812
MOLM-1
LJH685: – – + – – +
Imatinib: – + – – + –
225 K
76 K
52 K
38 K
31 K
24 K
17 K
12 K
RSK-S380P
1.00
0.63
0.90
0.37
0.18
0.28

Figure 2D

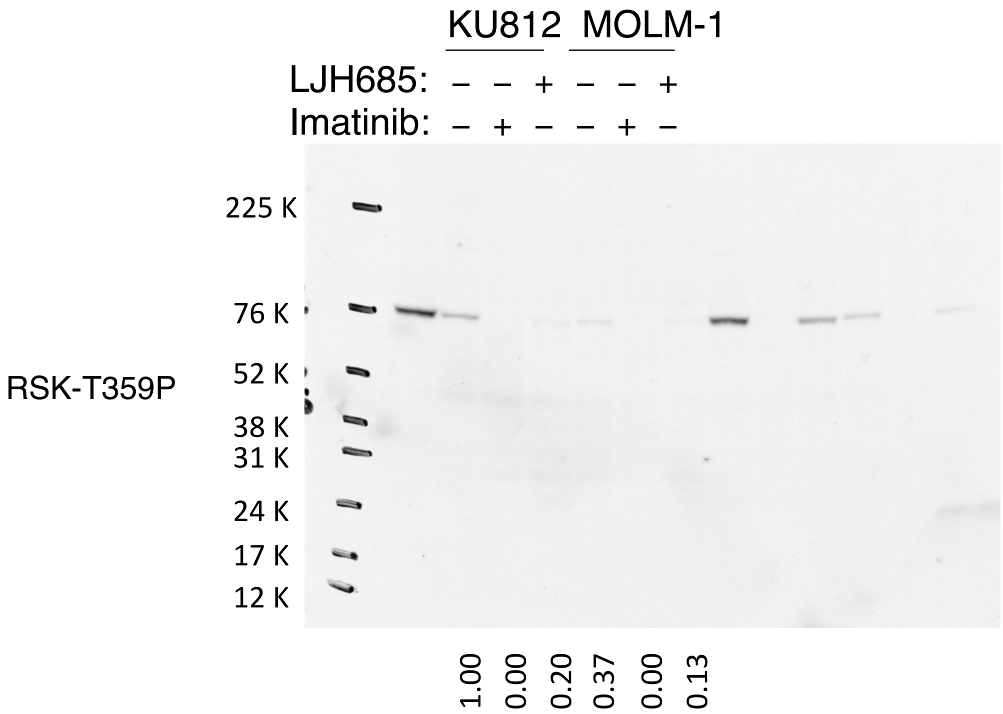
KU812
MOLM-1
LJH685: – – + – – +
Imatinib: – + – – + –
225 K
76 K
52 K
38 K
31 K
24 K
17 K
12 K
RSK-T359P
1.00
0.00
0.20
0.37
0.00
0.13

Figure 2D

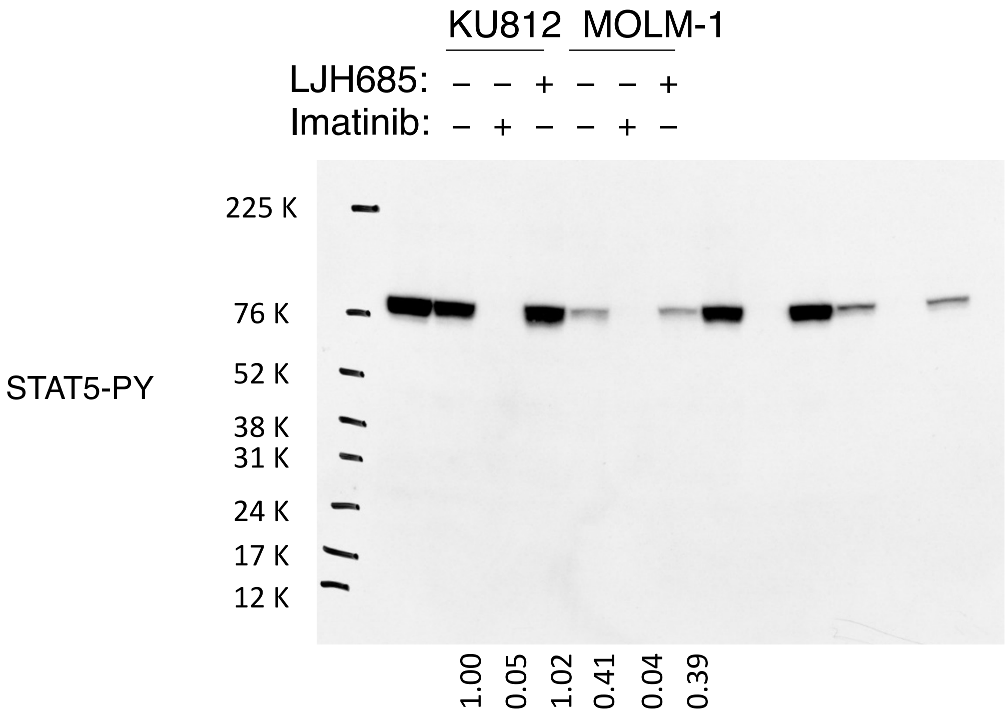
KU812
MOLM-1
LJH685: – – + – – +
Imatinib: – + – – + –
225 K
76 K
52 K
38 K
31 K
24 K
17 K
12 K
STAT5-PY
1.00
0.05
1.02
0.41
0.04
0.39

Figure 2D

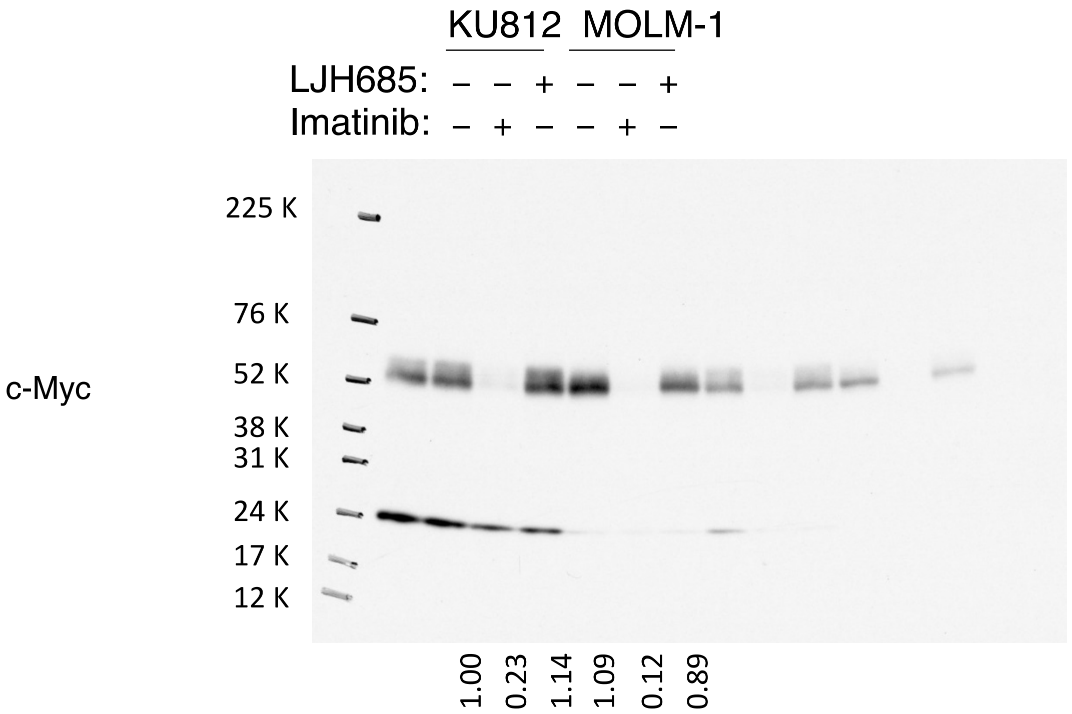

KU812 MOLM-1
LJH685: – – + – – +
Imatinib: – + – – + –
225 K
76 K
52 K
38 K
31 K
24 K
17 K
12 K
c-Myc
1.00
0.23
1.14
1.09
0.12
0.89

Figure 2D

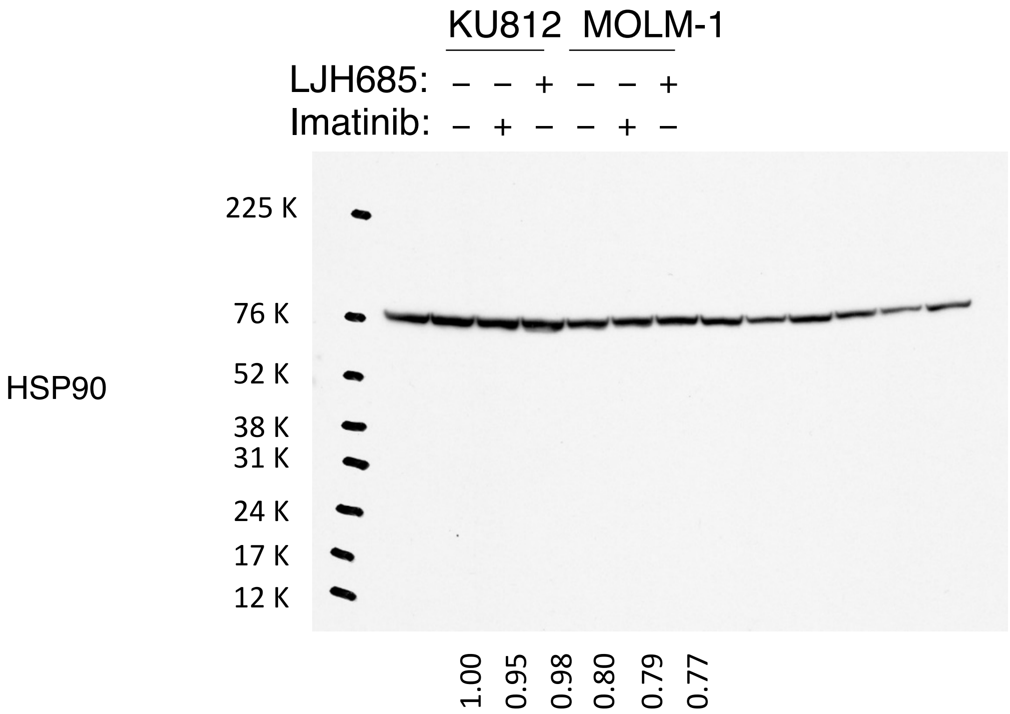
KU812
MOLM-1
LJH685: – – + – – +
Imatinib: – + – – + –
225 K
76 K
52 K
38 K
31 K
24 K
17 K
12 K
HSP90
1.00
0.95
0.98
0.80
0.79
0.77

Figure 2F

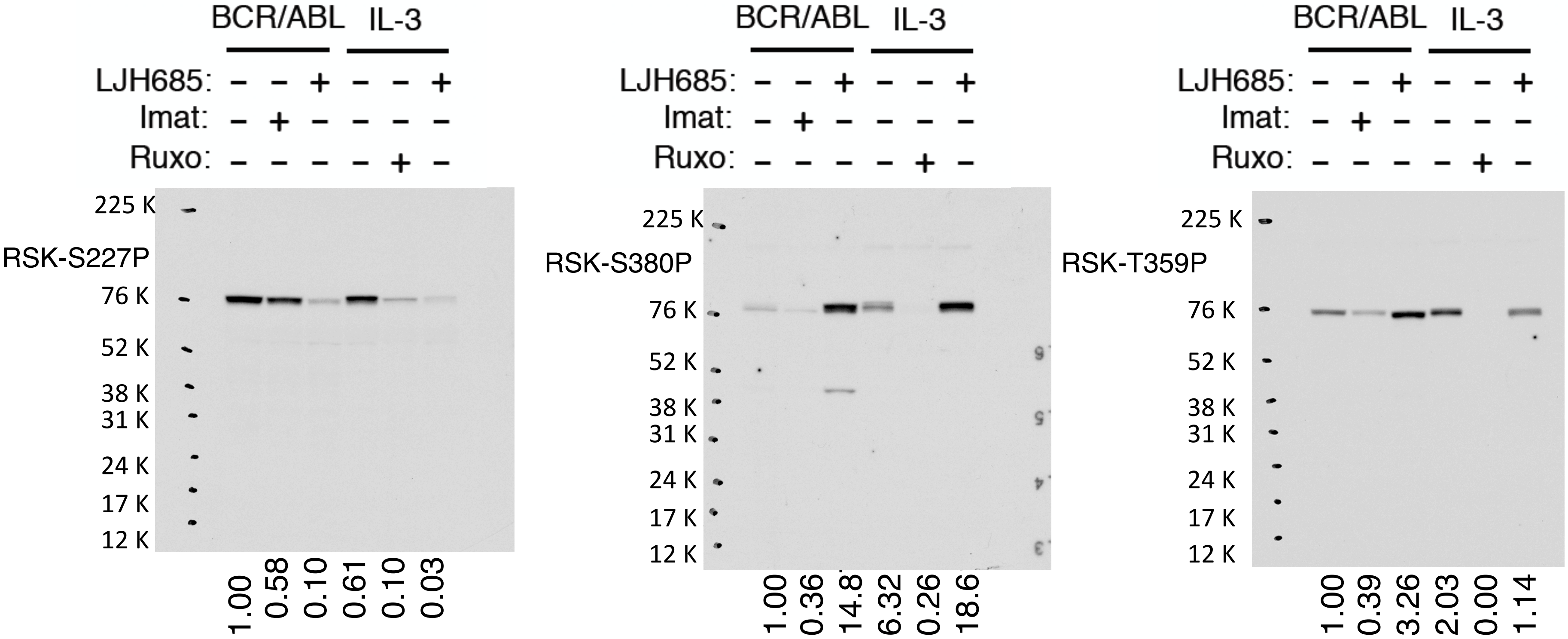
BCR/ABL
IL-3
LJH685: − − + − − +
Imat: − + − − − −
Ruxo: − − − − + −
225 K
RSK-S227P
76 K
52 K
38 K
31 K
24 K
17 K
12 K
1.00 0.58 0.10 0.61 0.10 0.03
RSK-S380P
1.00 0.36 14.8 6.32 0.26 18.6
RSK-T359P
1.00 0.39 3.26 2.03 0.00 1.14

Figure 2F

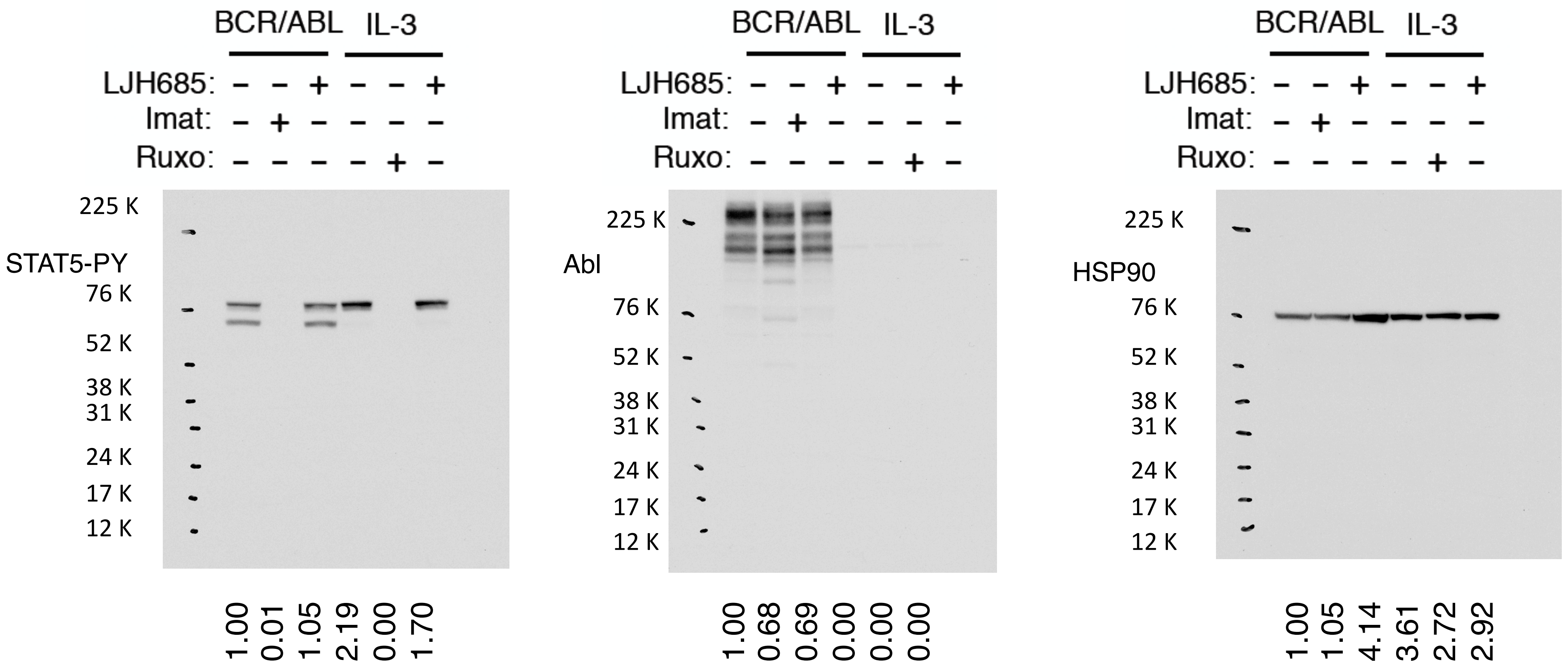
BCR/ABL
IL-3
LJH685: − − + − − +
Imat: − + − − − −
Ruxo: − − − − + −
225 K
STAT5-PY
76 K
52 K
38 K
31 K
24 K
17 K
12 K
1.00 0.01 1.05 2.19 0.00 1.70
Abl
1.00 0.68 0.69 0.00 0.00 0.00
HSP90
1.00 1.05 4.14 3.61 2.72 2.92

Figure 3A

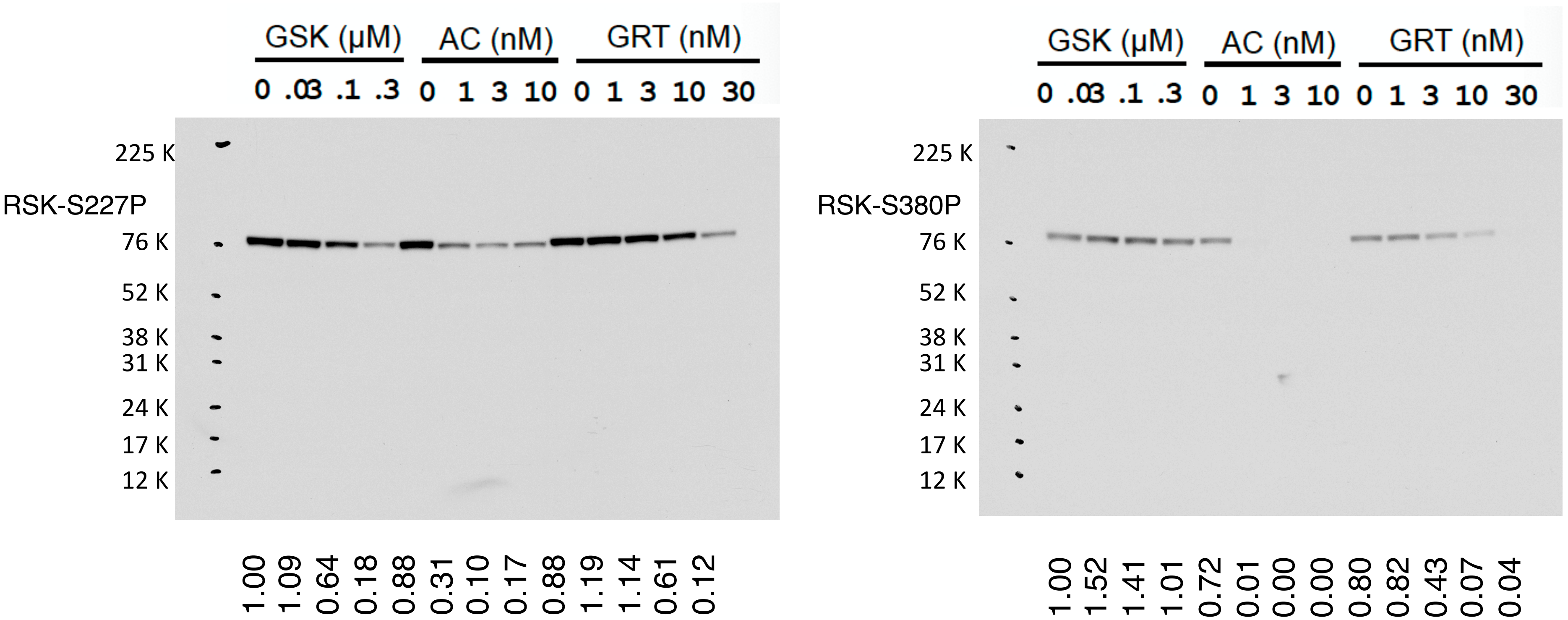
GSK (μM)
AC (nM)
GRT (nM)
0 .03 .1 .3 0 1 3 10 0 1 3 10 30
225 K
RSK-S227P
76 K
52 K
38 K
31 K
24 K
17 K
12 K
1.00 1.09 0.64 0.18 0.88 0.31 0.10 0.17 0.88 1.19 1.14 0.61 0.12
GSK (μM)
AC (nM)
GRT (nM)
0 .03 .1 .3 0 1 3 10 0 1 3 10 30
225 K
RSK-S380P
76 K
52 K
38 K
31 K
24 K
17 K
12 K
1.00 1.52 1.41 1.01 0.72 0.01 0.00 0.00 0.80 0.82 0.43 0.07 0.04

Figure 3A

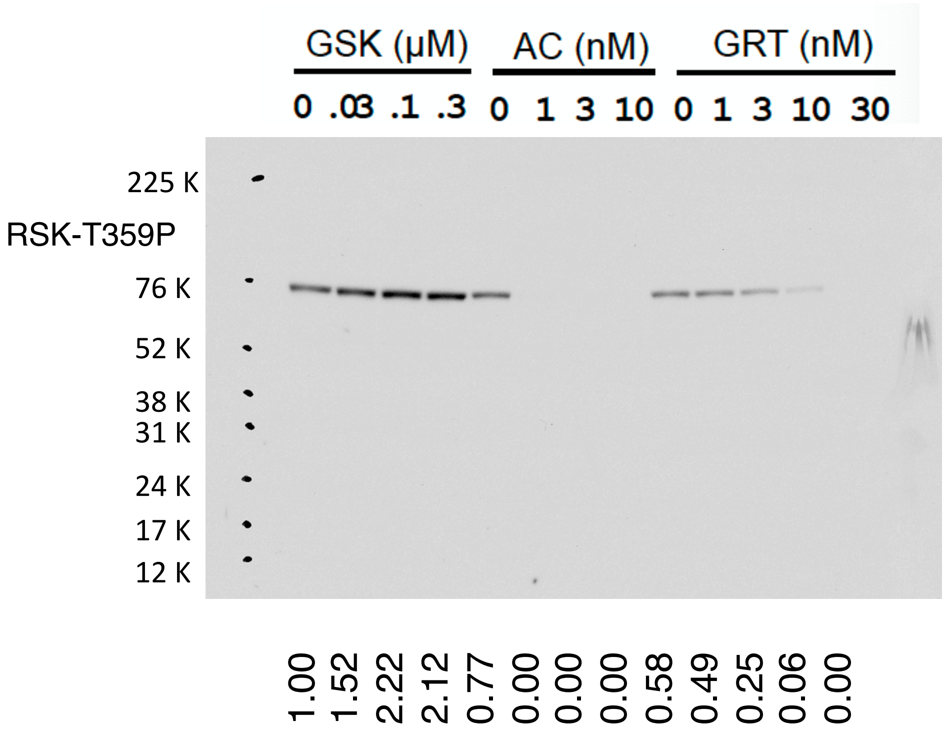
GSK (μM)
AC (nM)
GRT (nM)
0 .03 .1 .3 0 1 3 10 0 1 3 10 30
225 K
RSK-T359P
76 K
52 K
38 K
31 K
24 K
17 K
12 K
1.00 1.52 2.22 2.12 0.77 0.00 0.00 0.00 0.58 0.49 0.25 0.06 0.00

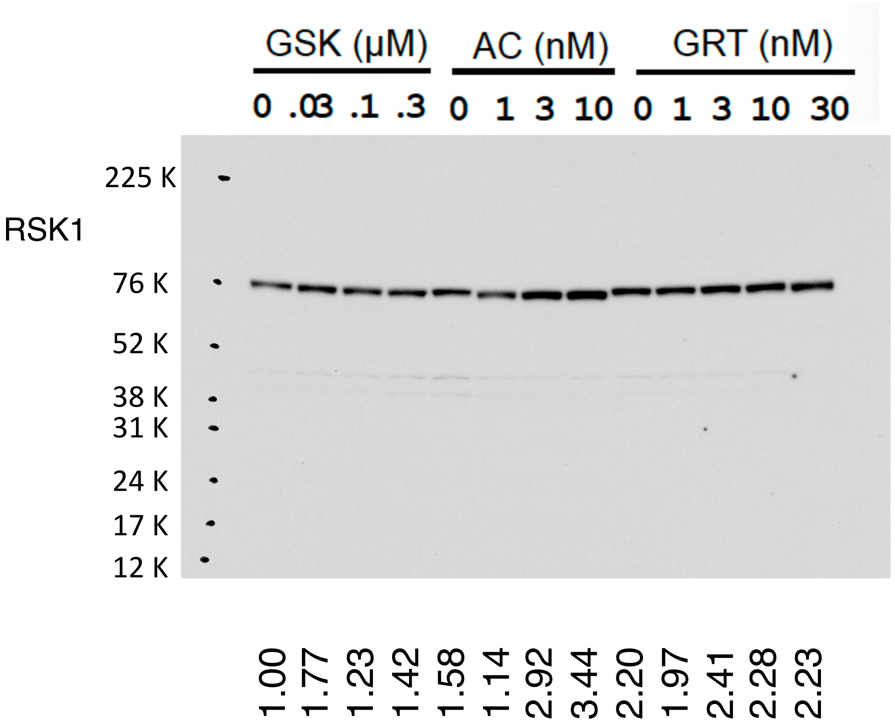
GSK (μM)
AC (nM)
GRT (nM)
0 .03 .1 .3 0 1 3 10 0 1 3 10 30
225 K
RSK1
76 K
52 K
38 K
31 K
24 K
17 K
12 K
1.00 1.77 1.23 1.42 1.58 1.14 2.92 3.44 2.20 1.97 2.41 2.28 2.23

Figure 3A

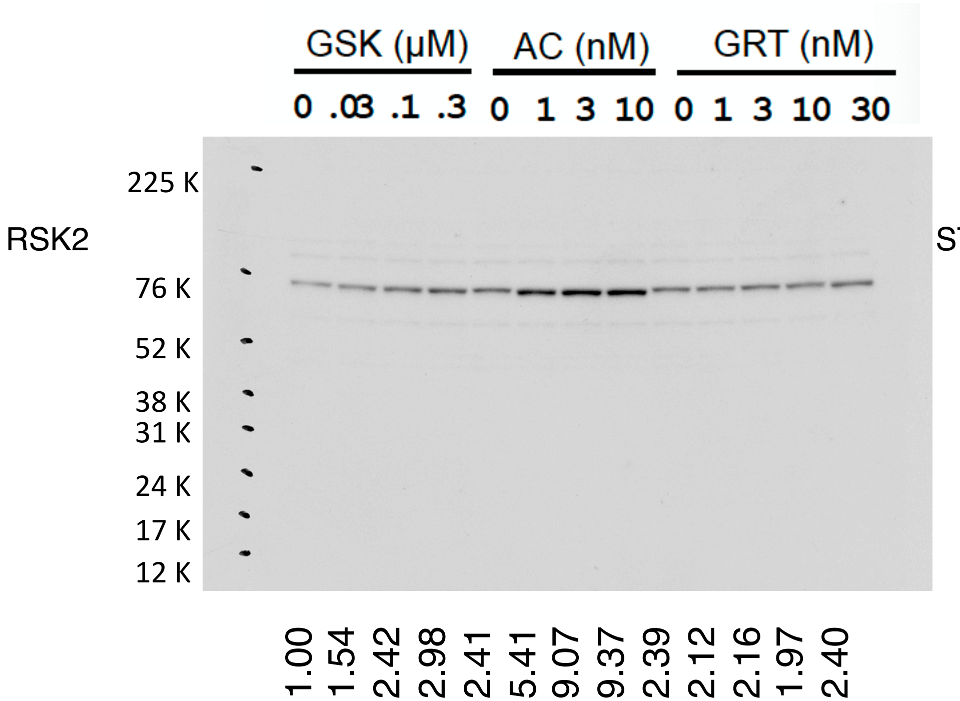
GSK (μM)
AC (nM)
GRT (nM)
0 .03 .1 .3 0 1 3 10 0 1 3 10 30
225 K
RSK2
76 K
52 K
38 K
31 K
24 K
17 K
12 K
1.00 1.54 2.42 2.98 2.41 5.41 9.07 9.37 2.39 2.12 2.16 1.97 2.40

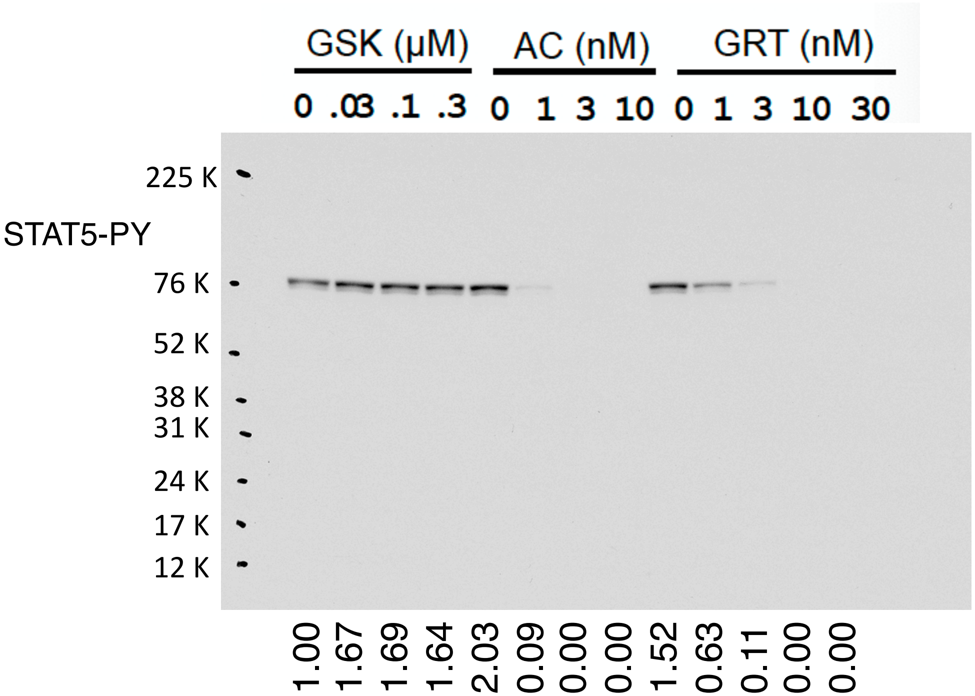
GSK (μM)
AC (nM)
GRT (nM)
0 .03 .1 .3 0 1 3 10 0 1 3 10 30
225 K
STAT5-PY
76 K
52 K
38 K
31 K
24 K
17 K
12 K
1.00 1.67 1.69 1.64 2.03 0.09 0.00 0.00 1.52 0.63 0.11 0.00 0.00

Figure 3A

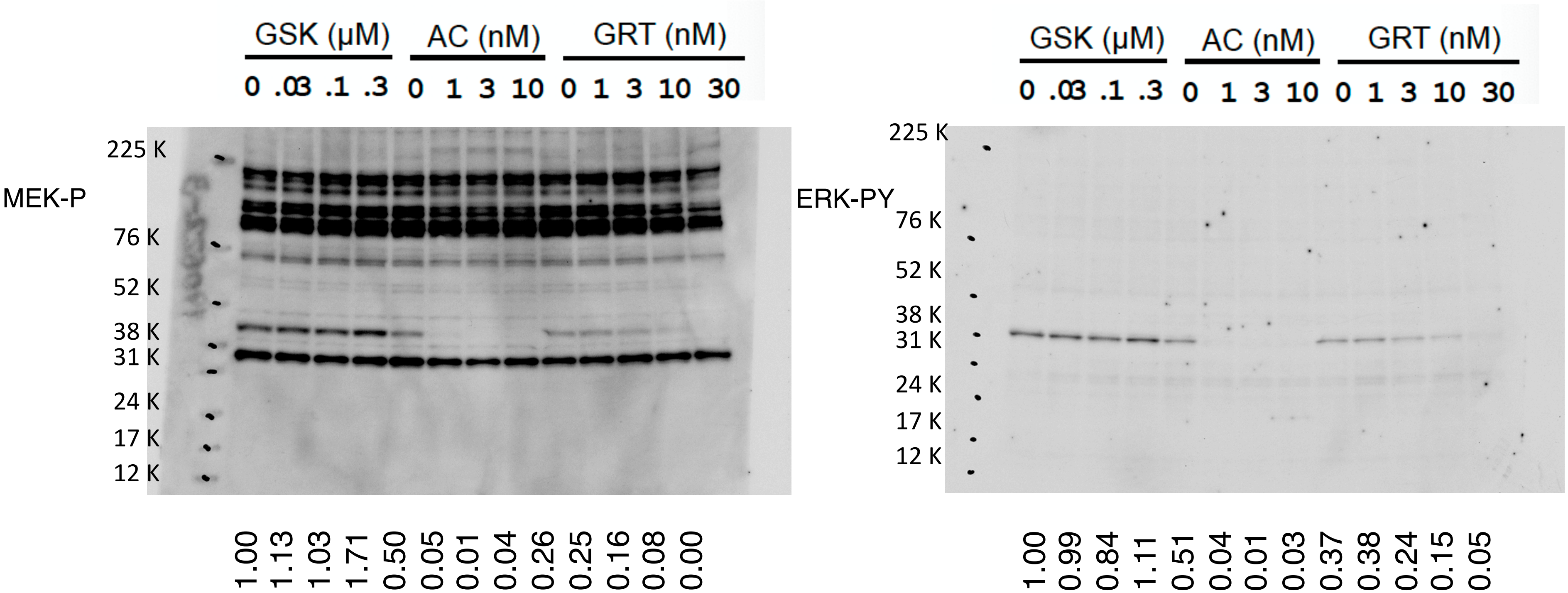

MEK-P
GSK (μM)
0 .03 .1 .3
AC (nM)
0 1 3 10
GRT (nM)
0 1 3 10 30
225 K
76 K
52 K
38 K
31 K
24 K
17 K
12 K
1.00 1.13 1.03 1.71 0.50 0.05 0.01 0.04 0.26 0.25 0.16 0.08 0.00
ERK-PY
GSK (μM)
0 .03 .1 .3
AC (nM)
0 1 3 10
GRT (nM)
0 1 3 10 30
225 K
76 K
52 K
38 K
31 K
24 K
17 K
12 K
1.00 0.99 0.84 1.11 0.51 0.04 0.01 0.03 0.37 0.38 0.24 0.15 0.05

Figure 3A

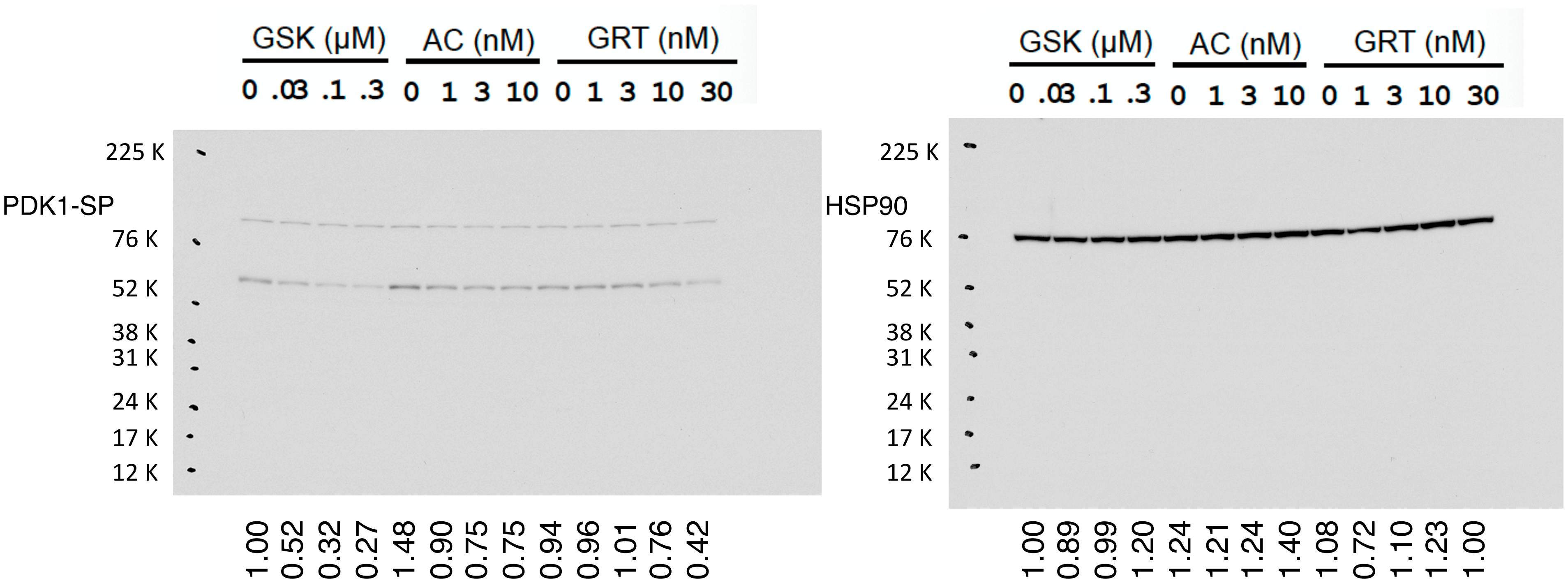
GSK (µM)
AC (nM)
GRT (nM)
0 .03 .1 .3 0 1 3 10 0 1 3 10 30
225 K
PDK1-SP
76 K
52 K
38 K
31 K
24 K
17 K
12 K
1.00 0.52 0.32 0.27 1.48 0.90 0.75 0.75 0.94 0.96 1.01 0.76 0.42
GSK (µM)
AC (nM)
GRT (nM)
0 .03 .1 .3 0 1 3 10 0 1 3 10 30
225 K
HSP90
76 K
52 K
38 K
31 K
24 K
17 K
12 K
1.00 0.89 0.99 1.20 1.24 1.21 1.24 1.40 1.08 0.72 1.10 1.23 1.00

Figure 3B

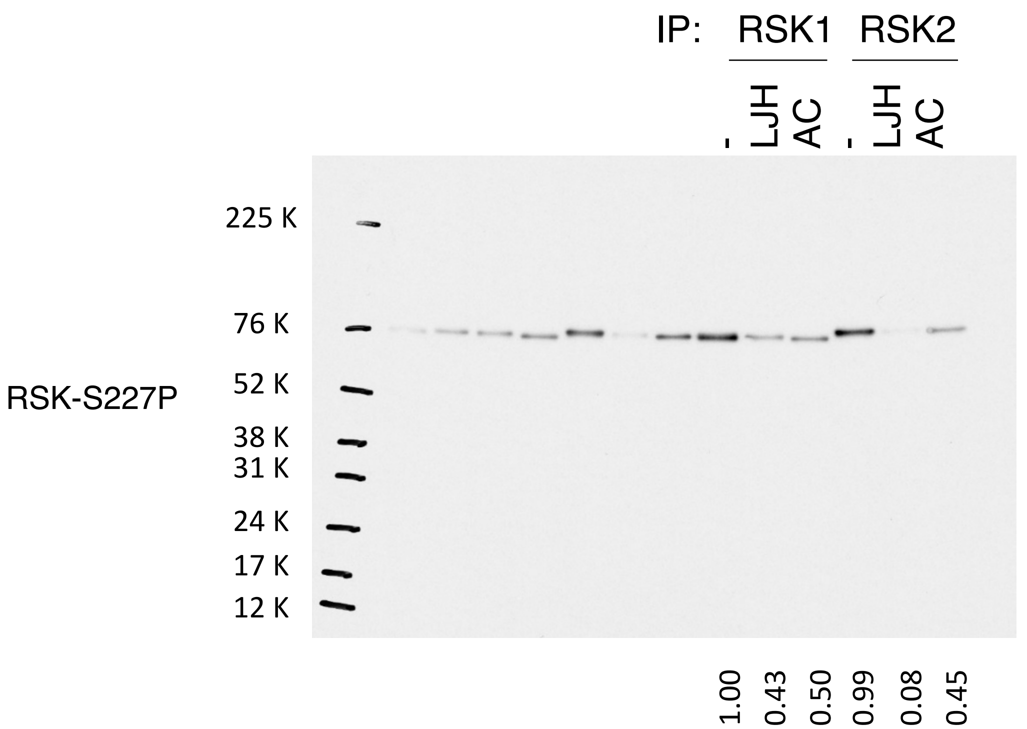

IP:
RSK1
RSK2
-
LJH
AC
-
LJH
AC
225 K
76 K
52 K
38 K
31 K
24 K
17 K
12 K
RSK-S227P
1.00
0.43
0.50
0.99
0.08
0.45

Figure 3B

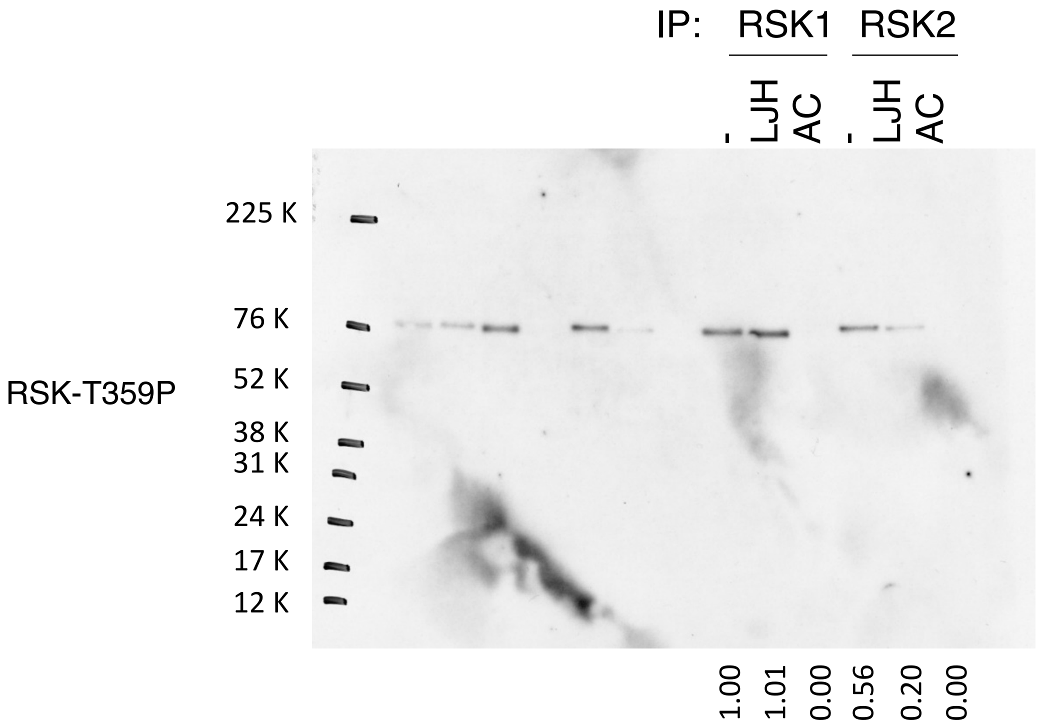
IP: RSK1 RSK2
-
LJH
AC
-
LJH
AC
225 K
76 K
52 K
38 K
31 K
24 K
17 K
12 K
RSK-T359P
1.00
1.01
0.00
0.56
0.20
0.00

Figure 3B

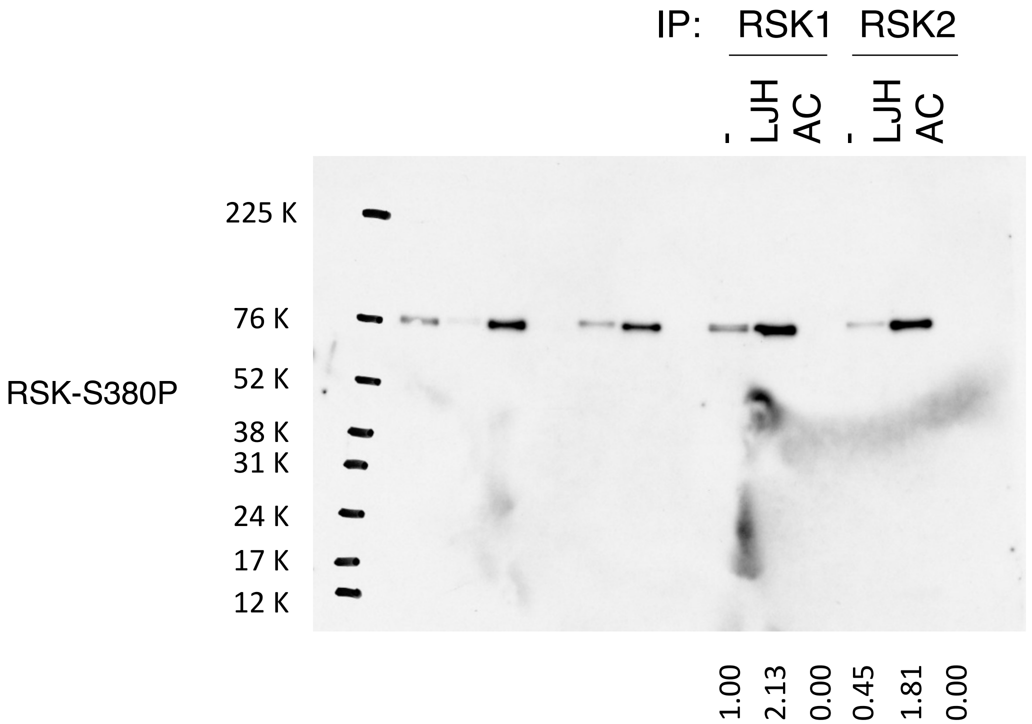

IP: RSK1 RSK2
-
LJH
AC
-
LJH
AC
225 K
76 K
52 K
38 K
31 K
24 K
17 K
12 K
RSK-S380P
1.00
2.13
0.00
0.45
1.81
0.00

Figure 3B

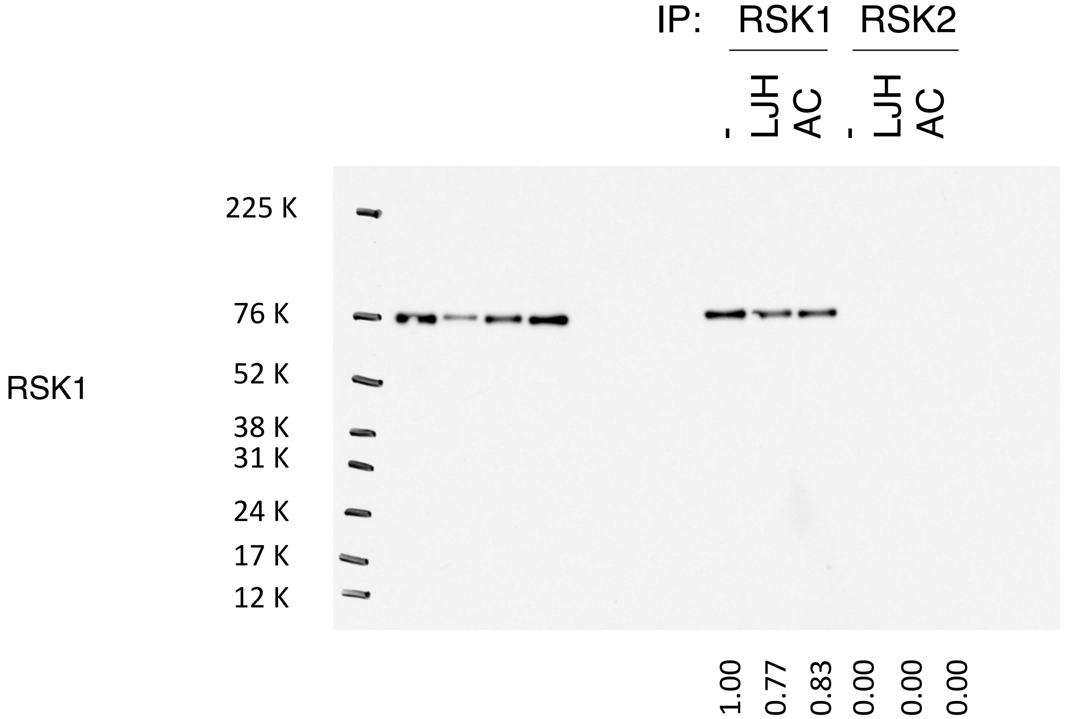
IP: RSK1 RSK2
- LJH AC - LJH AC
225 K
76 K
52 K
38 K
31 K
24 K
17 K
12 K
RSK1
1.00 0.77 0.83 0.00 0.00 0.00

Figure 3B

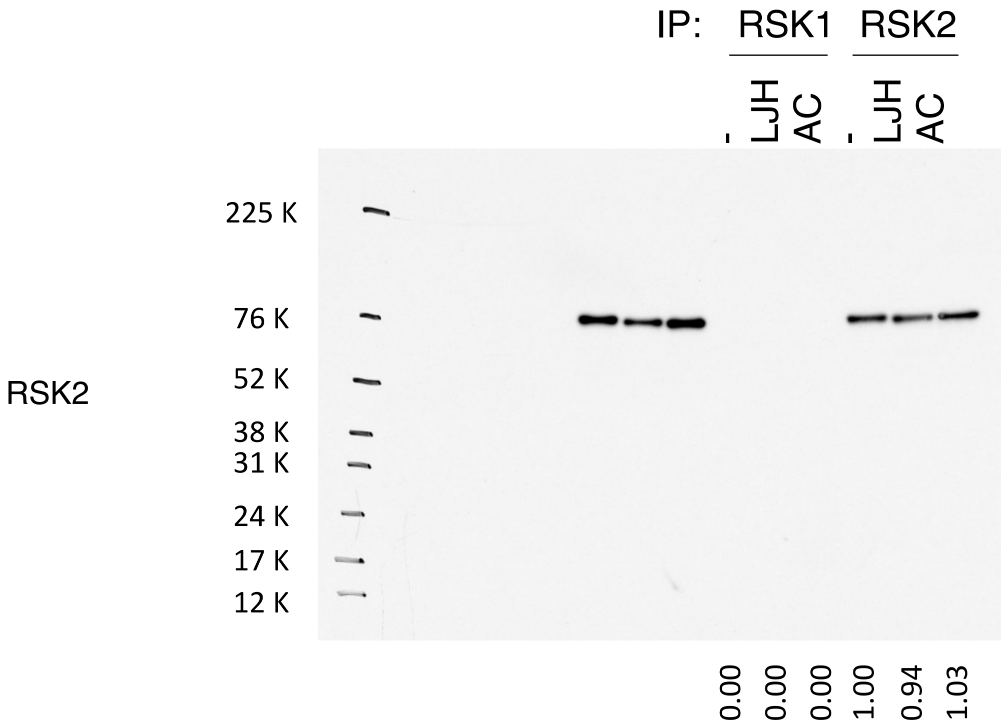
IP:
RSK1
RSK2
-
LJH
AC
-
LJH
AC
225 K
76 K
52 K
38 K
31 K
24 K
17 K
12 K
RSK2
0.00
0.00
0.00
1.00
0.94
1.03

Figure 3C

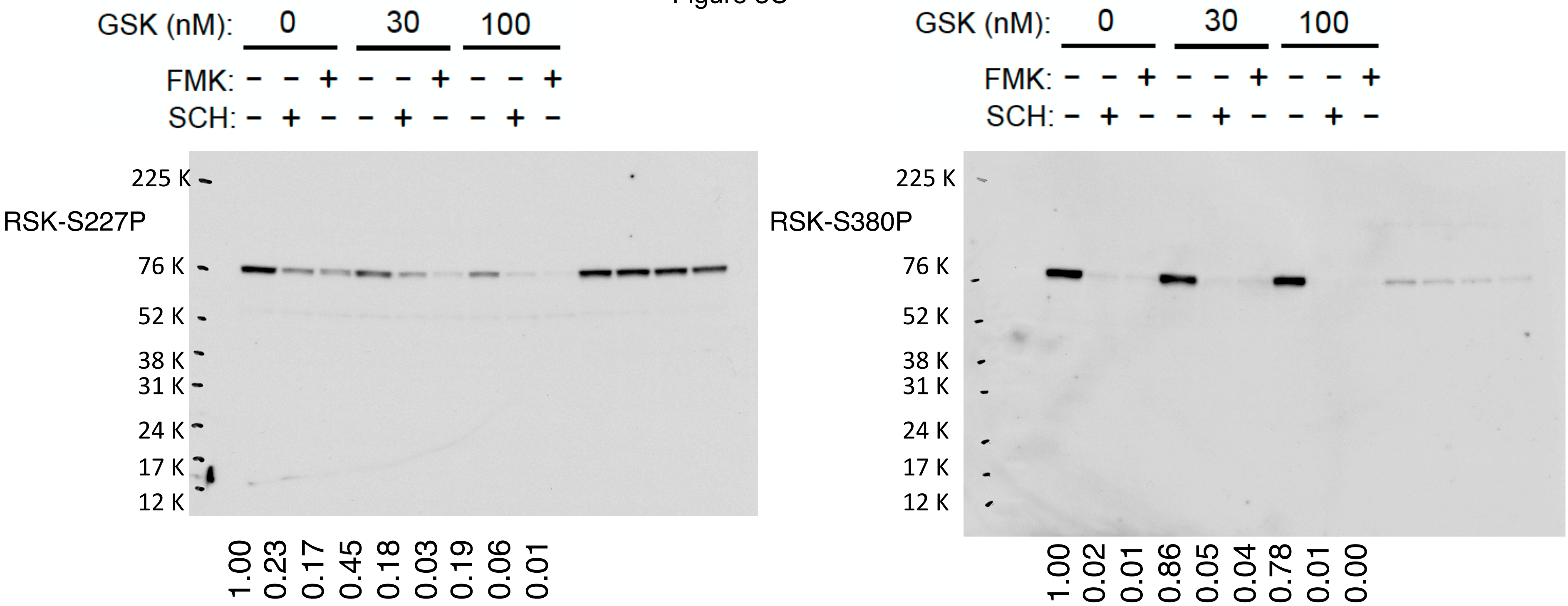
GSK (nM): 0 30 100
FMK: − − + − − + − − +
SCH: − + − − + − − + −
225 K
RSK-S227P
76 K
52 K
38 K
31 K
24 K
17 K
12 K
1.00 0.23 0.17 0.45 0.18 0.03 0.19 0.06 0.01
GSK (nM): 0 30 100
FMK: − − + − − + − − +
SCH: − + − − + − − + −
225 K
RSK-S380P
76 K
52 K
38 K
31 K
24 K
17 K
12 K
1.00 0.02 0.01 0.86 0.05 0.04 0.78 0.01 0.00

Figure 3C

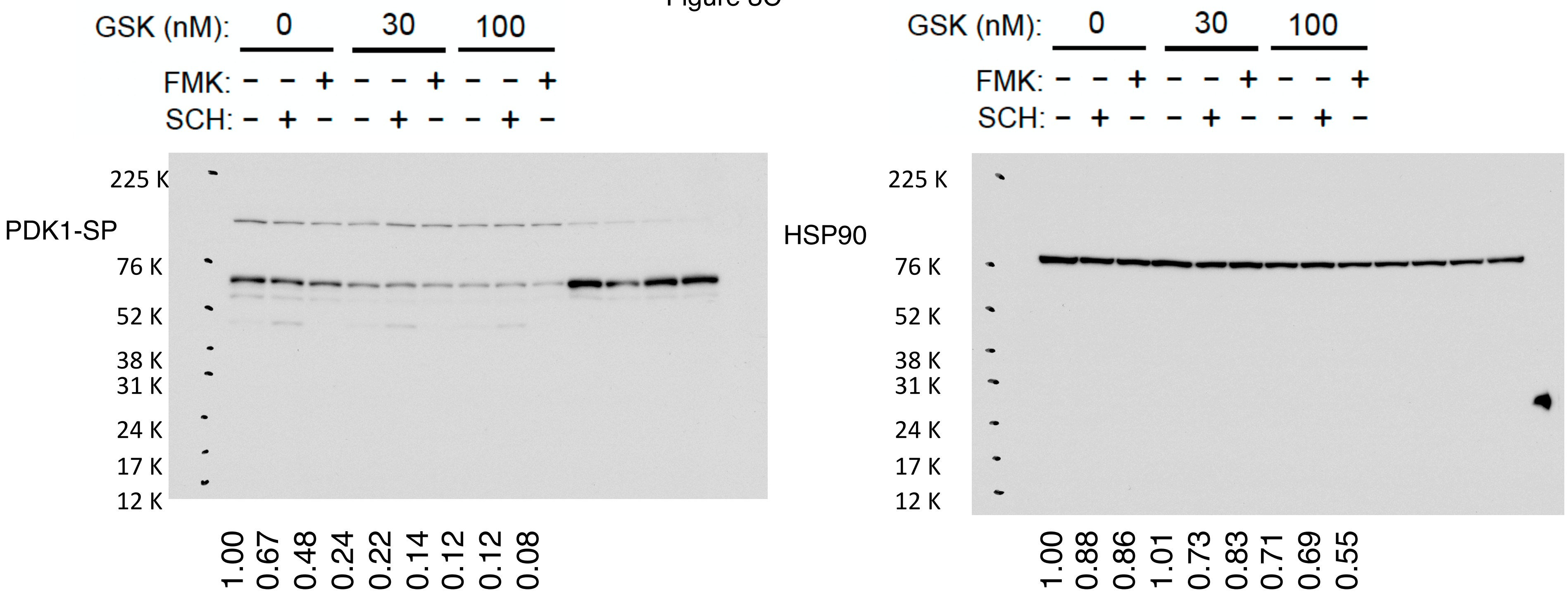

GSK (nM): 0 30 100
FMK: − − + − − + − − +
SCH: − + − − + − − + −
225 K
PDK1-SP
76 K
52 K
38 K
31 K
24 K
17 K
12 K
1.00 0.67 0.48 0.24 0.22 0.14 0.12 0.12 0.08
GSK (nM): 0 30 100
FMK: − − + − − + − − +
SCH: − + − − + − − + −
HSP90
225 K
76 K
52 K
38 K
31 K
24 K
17 K
12 K
1.00 0.88 0.86 1.01 0.73 0.83 0.71 0.69 0.55

Figure 3D

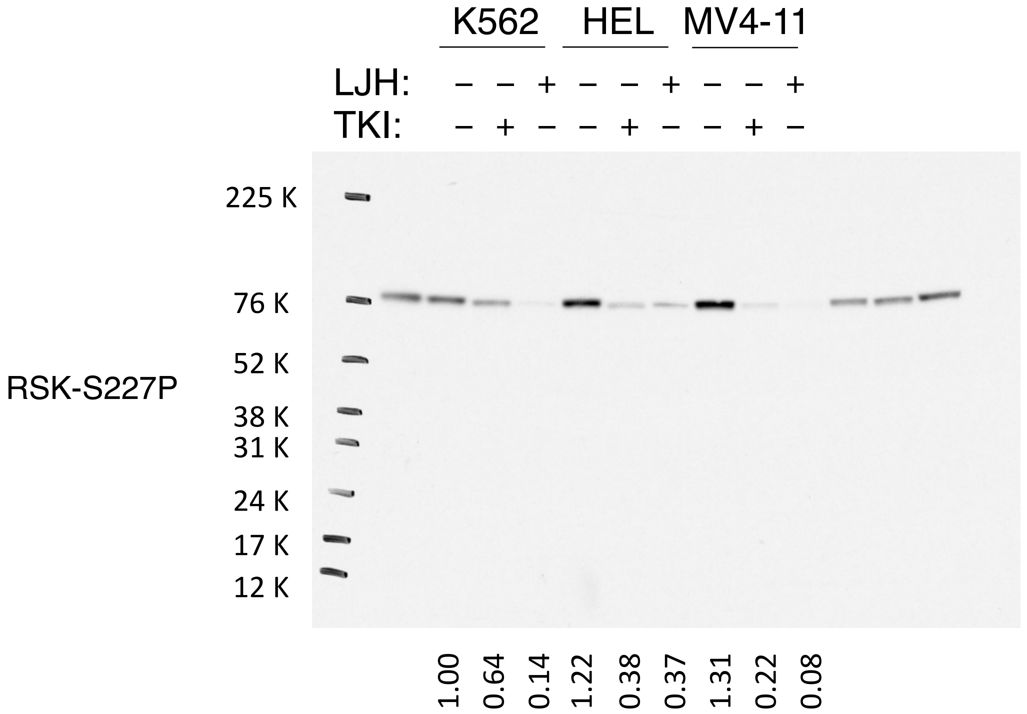
K562
HEL
MV4-11
LJH: − − + − − + − − +
TKI: − + − − + − − + −
225 K
76 K
52 K
38 K
31 K
24 K
17 K
12 K
RSK-S227P
1.00
0.64
0.14
1.22
0.38
0.37
1.31
0.22
0.08

Figure 3D

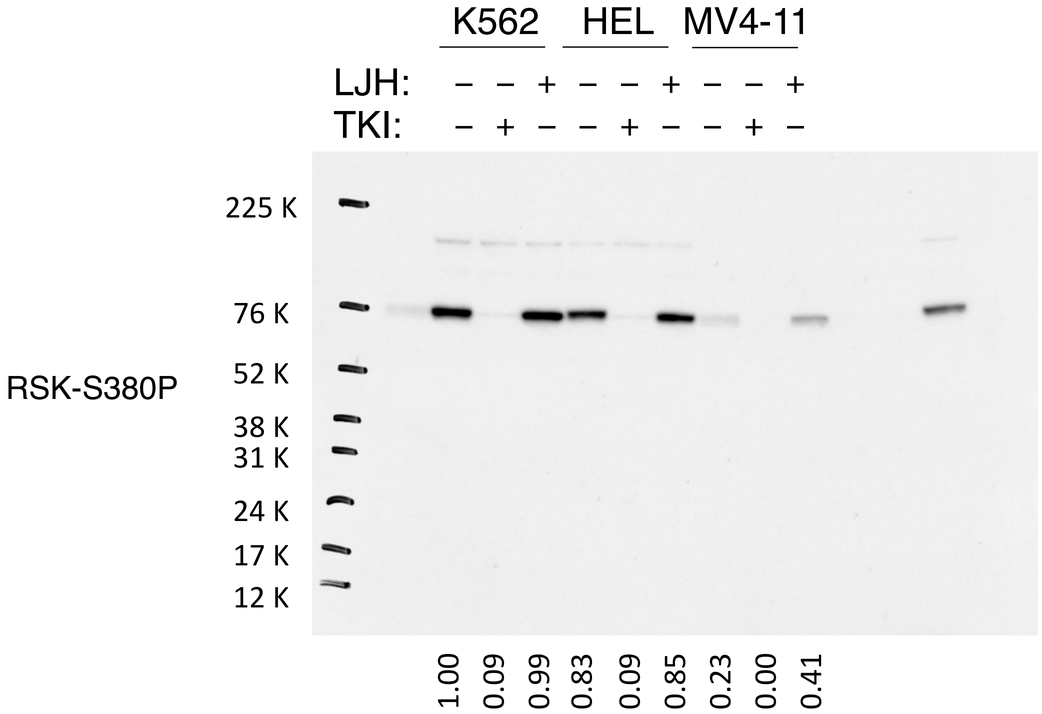
K562
HEL
MV4-11
LJH: − − + − − + − − +
TKI: − + − − + − − + −
225 K
76 K
52 K
38 K
31 K
24 K
17 K
12 K
RSK-S380P
1.00
0.09
0.99
0.83
0.09
0.85
0.23
0.00
0.41

Figure 3D

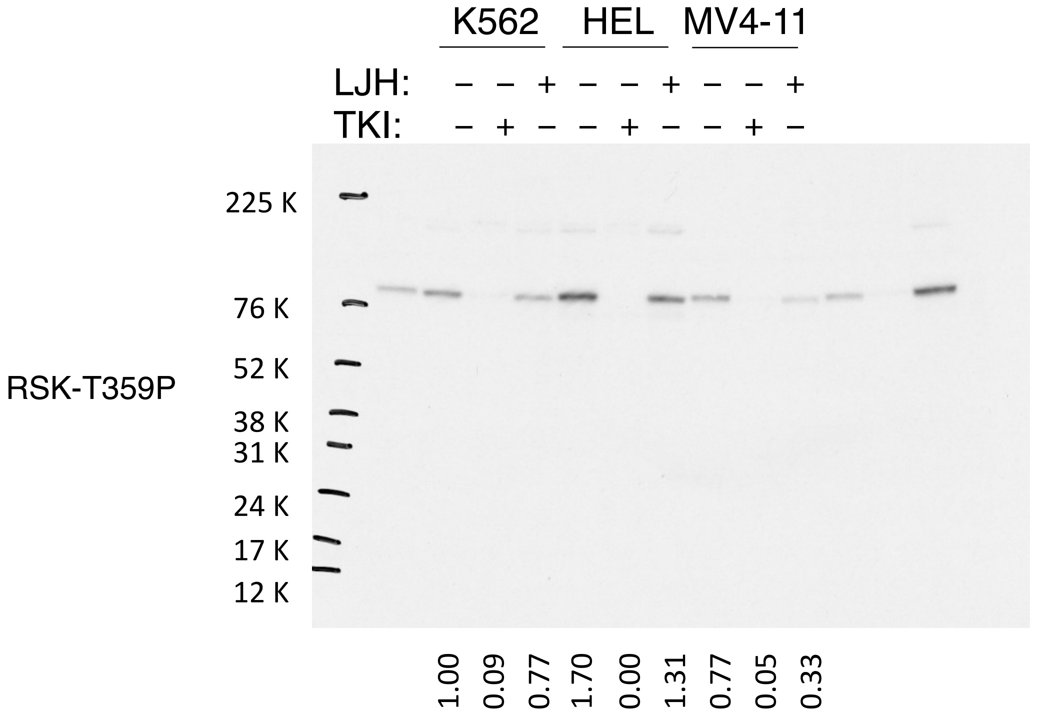
K562
HEL
MV4-11
LJH: − − + − − + − − +
TKI: − + − − + − − + −
225 K
76 K
52 K
38 K
31 K
24 K
17 K
12 K
RSK-T359P
1.00
0.09
0.77
1.70
0.00
1.31
0.77
0.05
0.33

Figure 3D

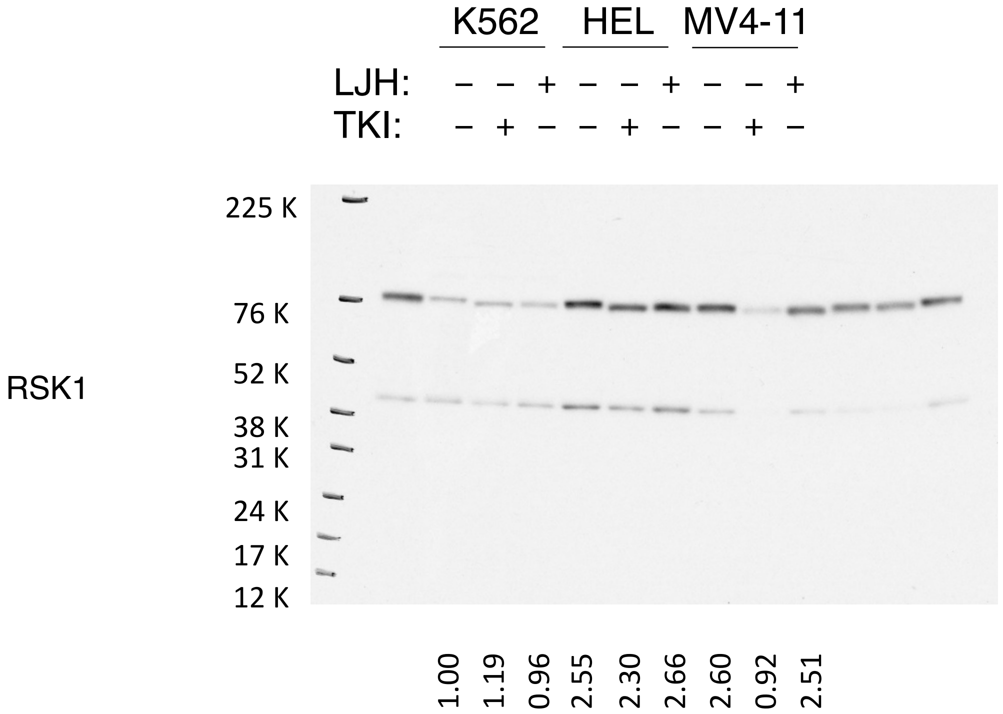
K562
HEL
MV4-11
LJH: − − + − − + − − +
TKI: − + − − + − − + −
225 K
76 K
52 K
38 K
31 K
24 K
17 K
12 K
RSK1
1.00
1.19
0.96
2.55
2.30
2.66
2.60
0.92
2.51

Figure 3D

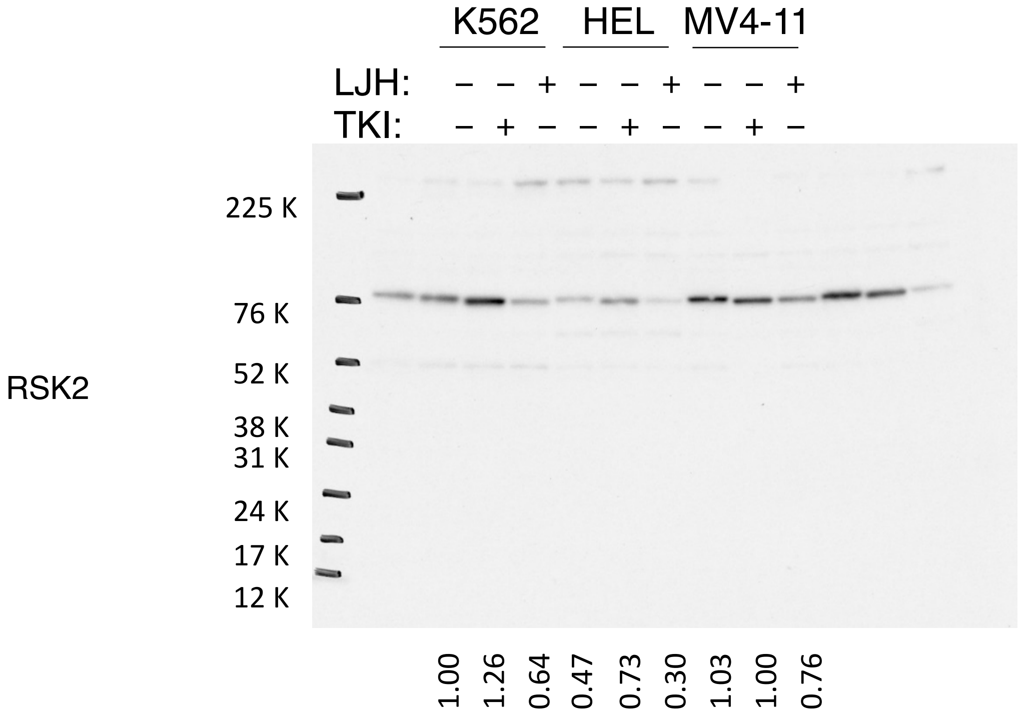
K562
HEL
MV4-11
LJH: − − + − − + − − +
TKI: − + − − + − − + −
225 K
76 K
52 K
38 K
31 K
24 K
17 K
12 K
RSK2
1.00
1.26
0.64
0.47
0.73
0.30
1.03
1.00
0.76

Figure 3D

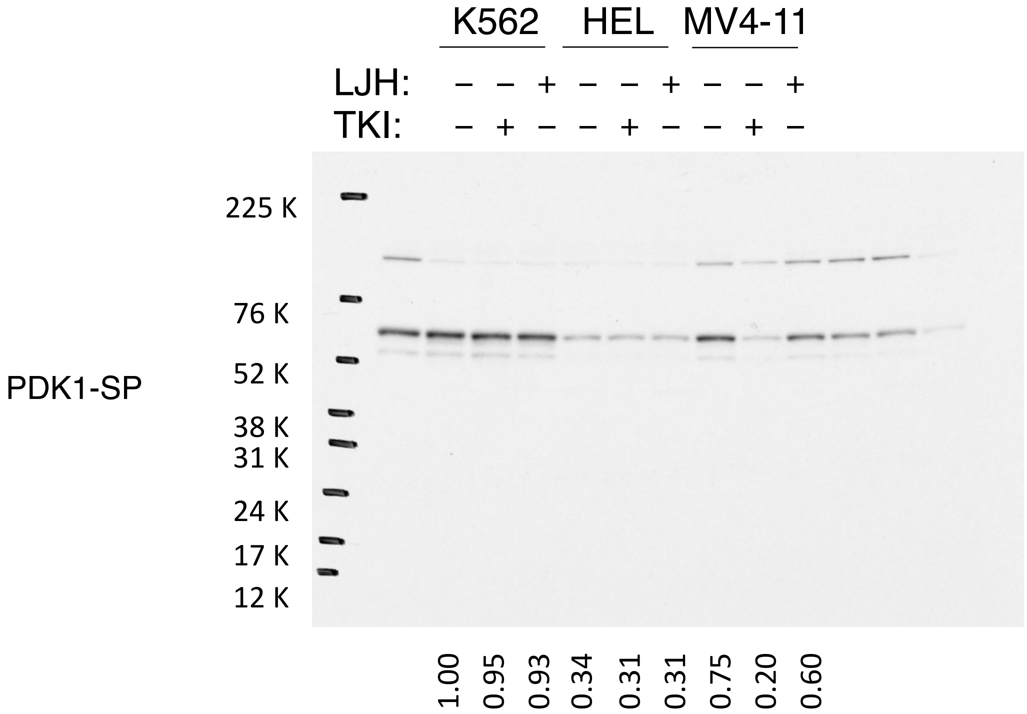

K562
HEL
MV4-11
LJH: − − + − − + − − +
TKI: − + − − + − − + −
225 K
76 K
52 K
38 K
31 K
24 K
17 K
12 K
PDK1-SP
1.00 0.95 0.93 0.34 0.31 0.31 0.75 0.20 0.60

Figure 3D

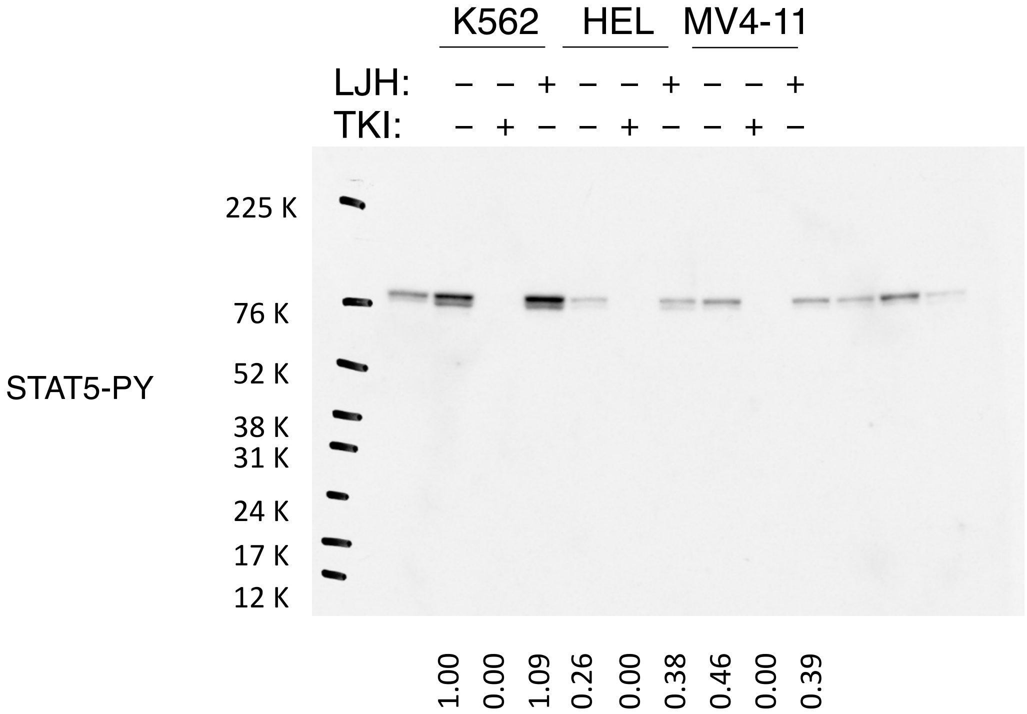
K562
HEL
MV4-11
LJH: − − + − − + − − +
TKI: − + − − + − − + −
225 K
76 K
52 K
38 K
31 K
24 K
17 K
12 K
STAT5-PY
1.00
0.00
1.09
0.26
0.00
0.38
0.46
0.00
0.39

Figure 3D

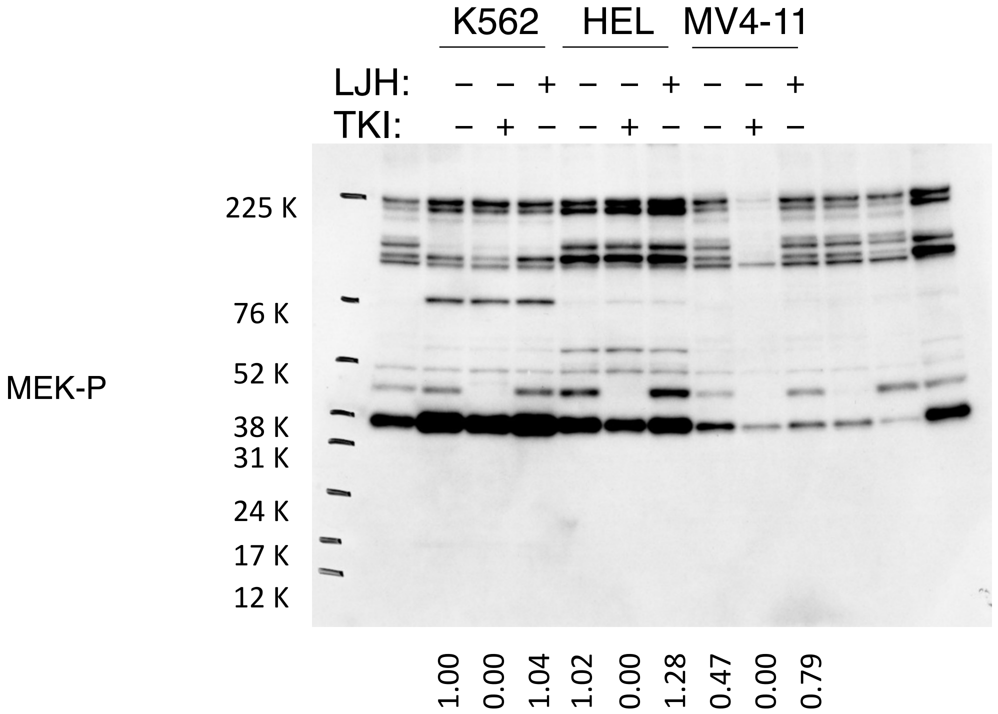
K562
HEL
MV4-11
LJH: − − + − − + − − +
TKI: − + − − + − − + −
225 K
76 K
52 K
38 K
31 K
24 K
17 K
12 K
MEK-P
1.00 0.00 1.04 1.02 0.00 1.28 0.47 0.00 0.79

Figure 3D

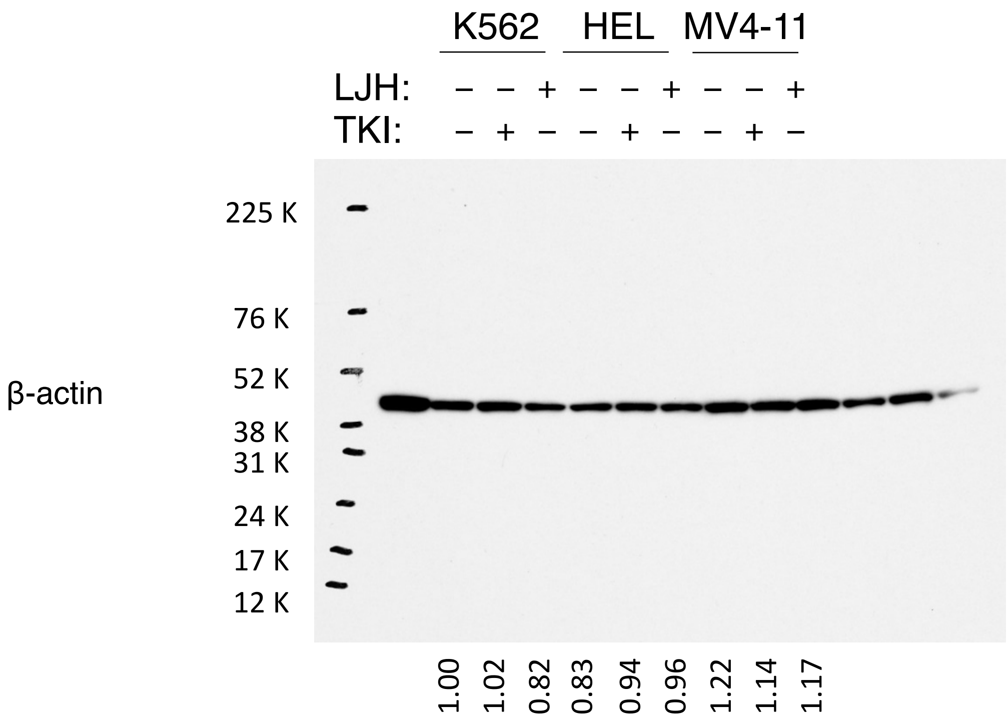

K562
HEL
MV4-11
LJH: − − + − − + − − +
TKI: − + − − + − − + −
225 K
76 K
52 K
38 K
31 K
24 K
17 K
12 K
β-actin
1.00
1.02
0.82
0.83
0.94
0.96
1.22
1.14
1.17

Figure 3E

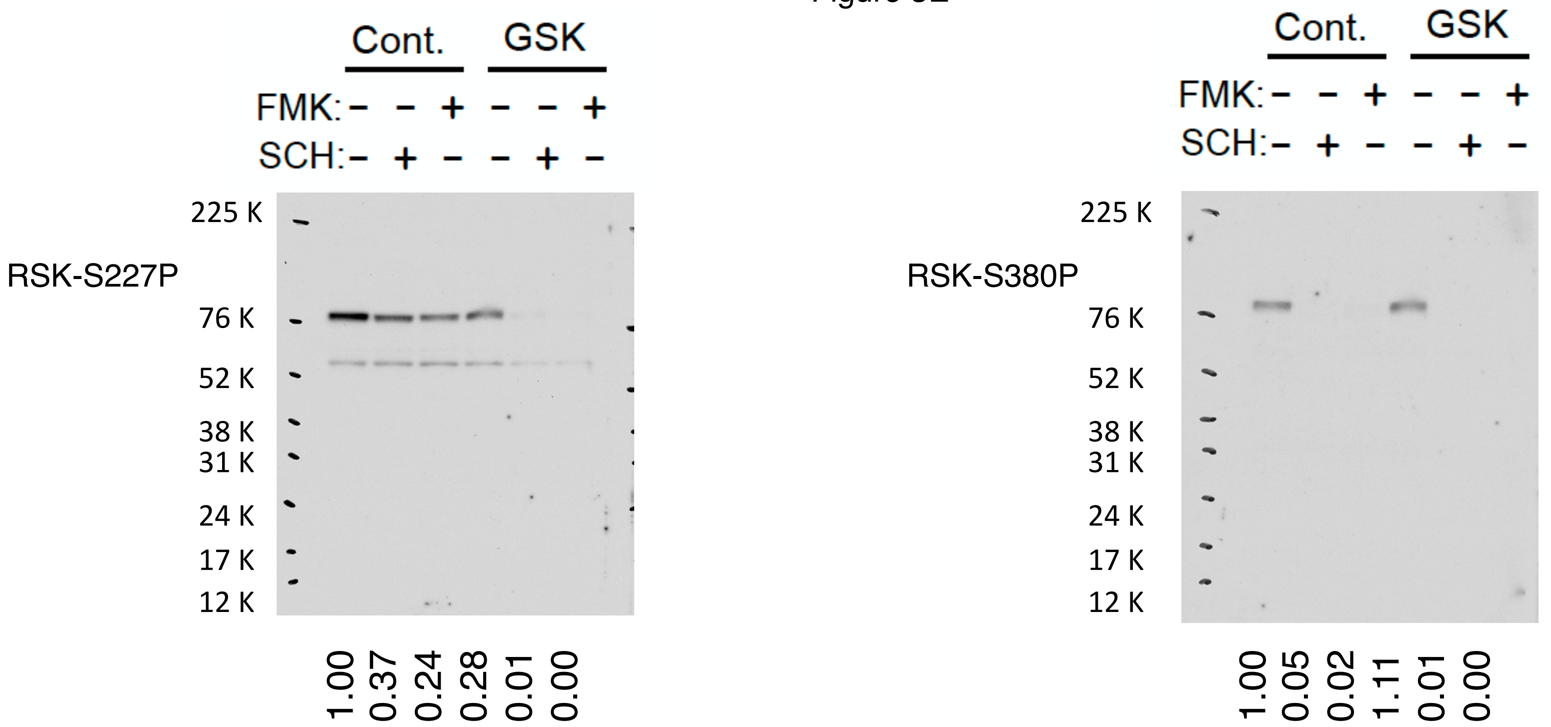
Cont.
GSK
FMK: – – + – – +
SCH: – + – – + –
225 K
RSK-S227P
76 K
52 K
38 K
31 K
24 K
17 K
12 K
1.00 0.37 0.24 0.28 0.01 0.00
Cont.
GSK
FMK: – – + – – +
SCH: – + – – + –
225 K
RSK-S380P
76 K
52 K
38 K
31 K
24 K
17 K
12 K
1.00 0.05 0.02 1.11 0.01 0.00

Figure 3E

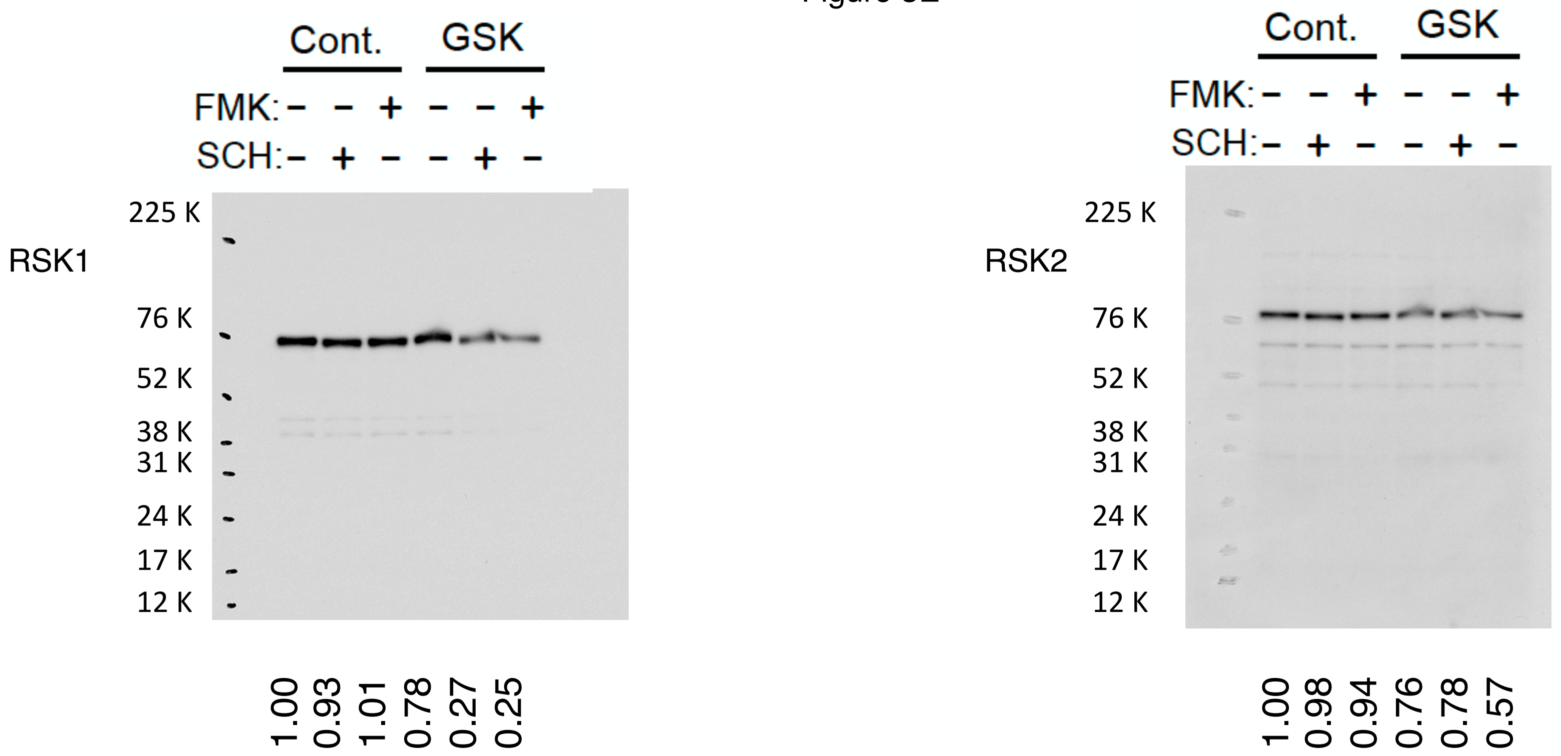

Cont.
GSK
FMK: − − + − − +
SCH: − + − − + −
225 K
RSK1
76 K
52 K
38 K
31 K
24 K
17 K
12 K
1.00 0.93 1.01 0.78 0.27 0.25
Cont.
GSK
FMK: − − + − − +
SCH: − + − − + −
225 K
RSK2
76 K
52 K
38 K
31 K
24 K
17 K
12 K
1.00 0.98 0.94 0.76 0.78 0.57

Figure 3E

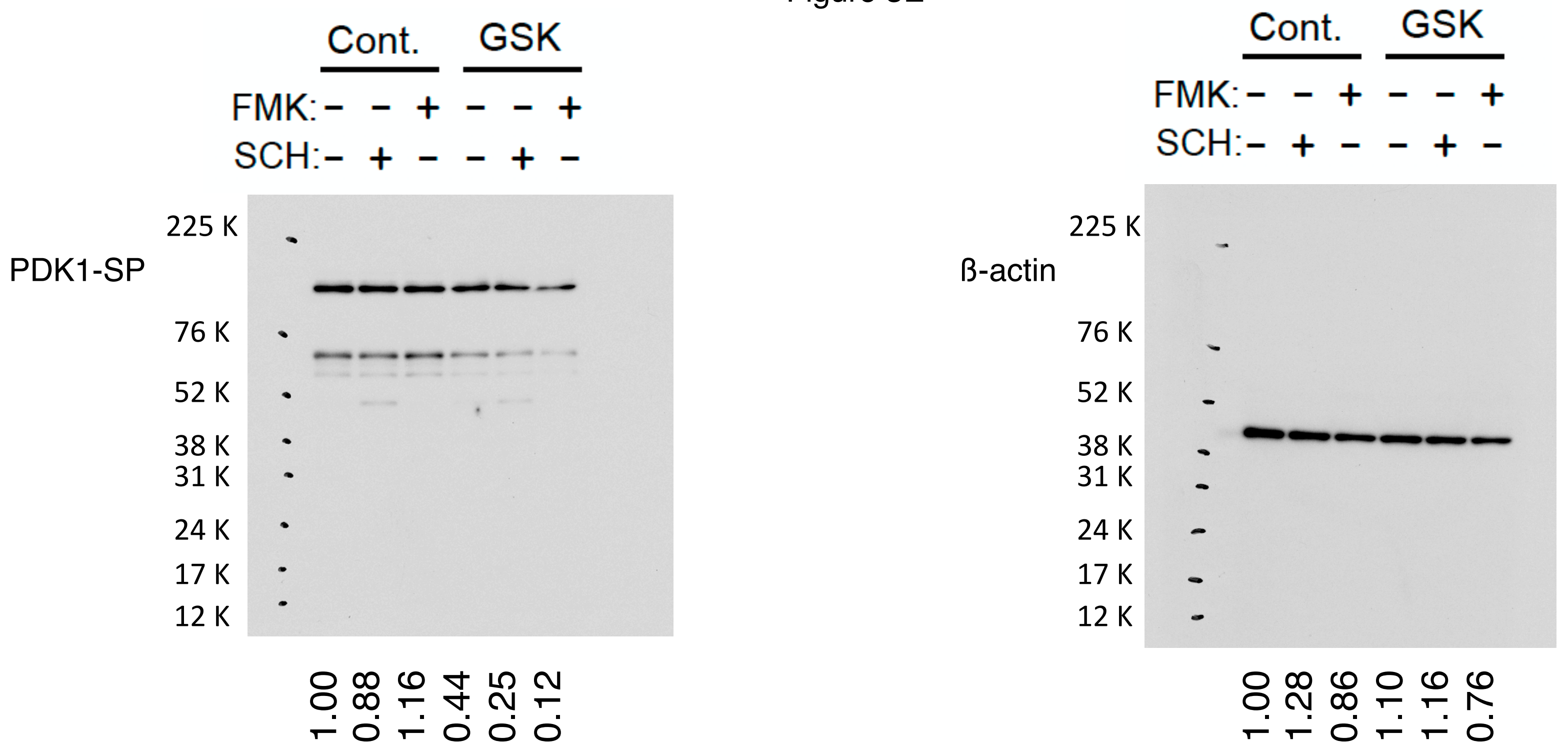

Cont.
GSK
FMK: − − + − − +
SCH: − + − − + −
225 K
76 K
52 K
38 K
31 K
24 K
17 K
12 K
PDK1-SP
1.00 0.88 1.16 0.44 0.25 0.12
ß-actin
1.00 1.28 0.86 1.10 1.16 0.76

Figure 3F

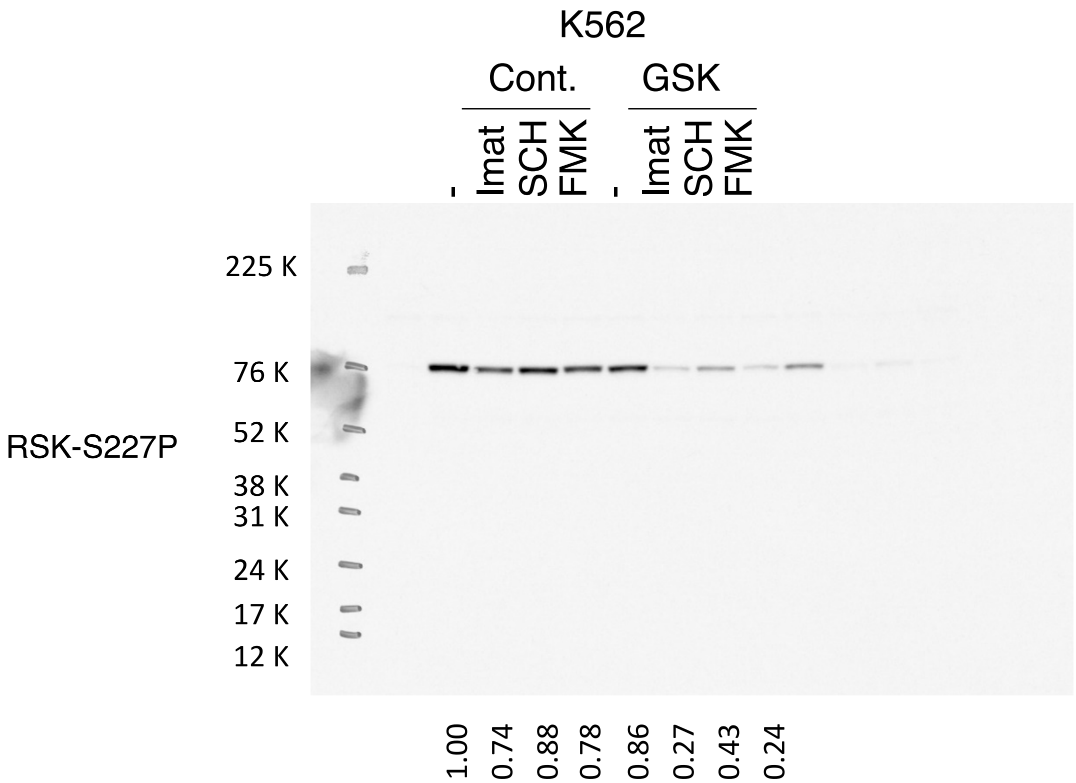
K562
Cont.
GSK
-
Imat
SCH
FMK
-
Imat
SCH
FMK
225 K
76 K
52 K
38 K
31 K
24 K
17 K
12 K
RSK-S227P
1.00
0.74
0.88
0.78
0.86
0.27
0.43
0.24

Figure 3F

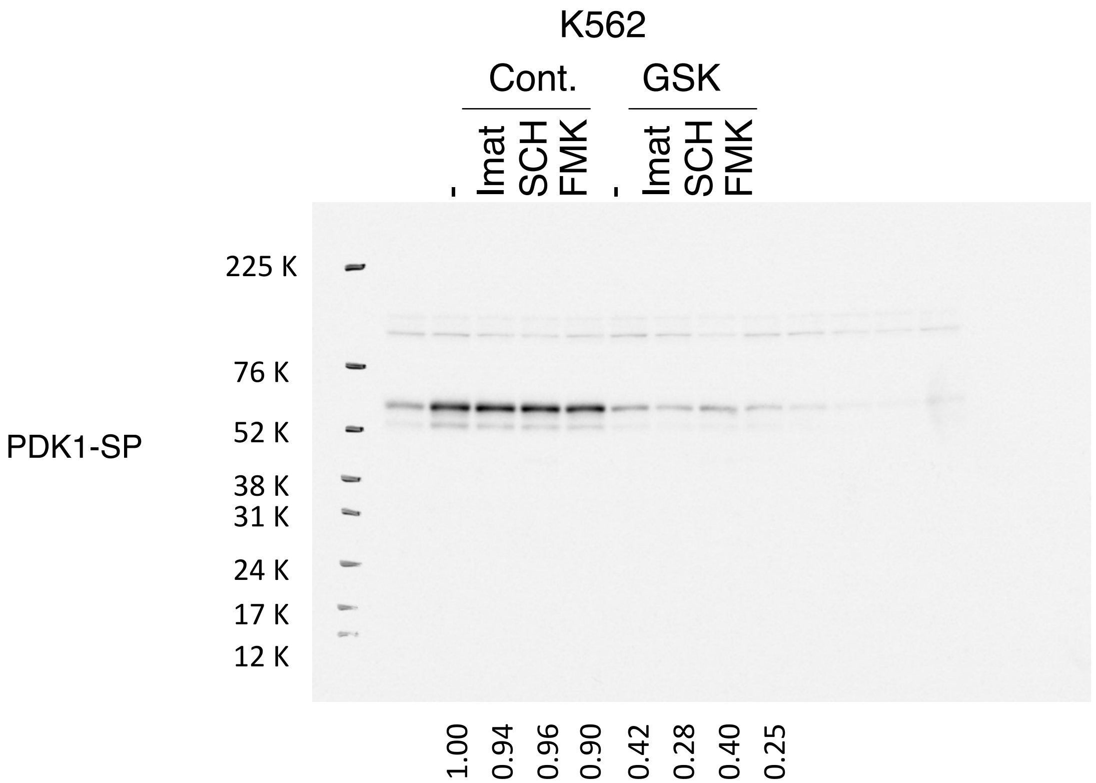
K562
Cont.
GSK
-
Imat
SCH
FMK
-
Imat
SCH
FMK
225 K
76 K
52 K
38 K
31 K
24 K
17 K
12 K
PDK1-SP
1.00
0.94
0.96
0.90
0.42
0.28
0.40
0.25

Figure 3F

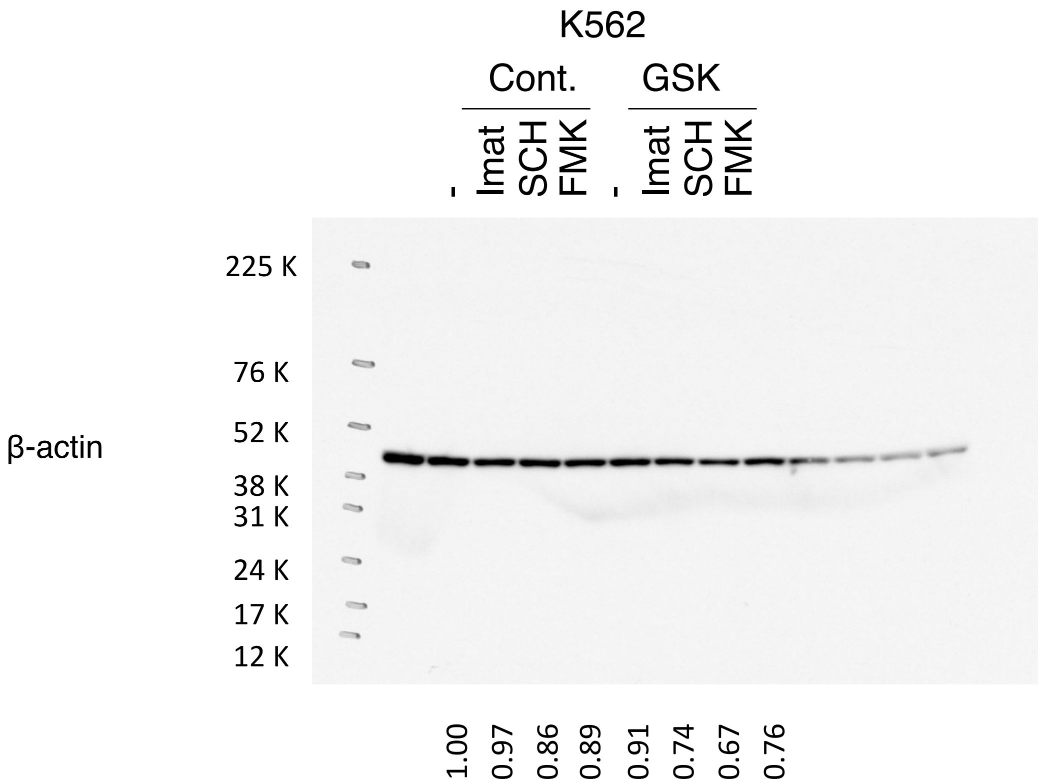

K562
Cont.
GSK
-
Imat
SCH
FMK
-
Imat
SCH
FMK
225 K
76 K
52 K
38 K
31 K
24 K
17 K
12 K
β-actin
1.00
0.97
0.86
0.89
0.91
0.74
0.67
0.76

Figure 3G

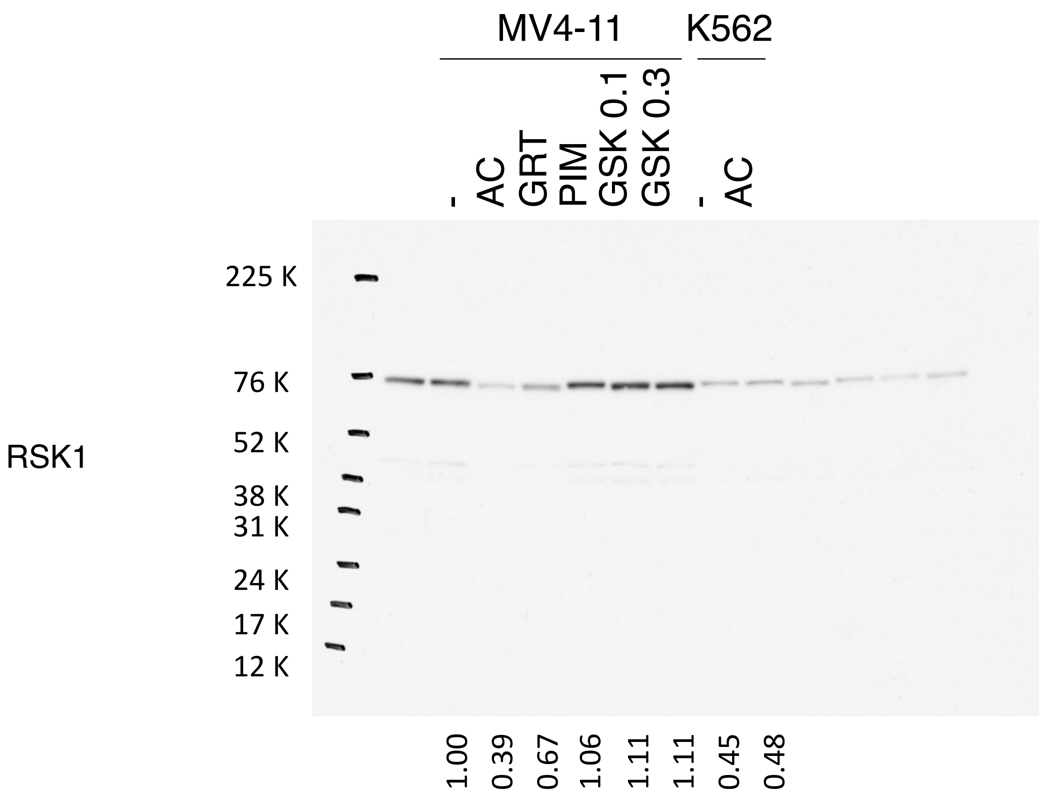
MV4-11
K562
-
AC
GRT
PIM
GSK 0.1
GSK 0.3
-
AC
225 K
76 K
52 K
38 K
31 K
24 K
17 K
12 K
RSK1
1.00
0.39
0.67
1.06
1.11
1.11
0.45
0.48

Figure 3G

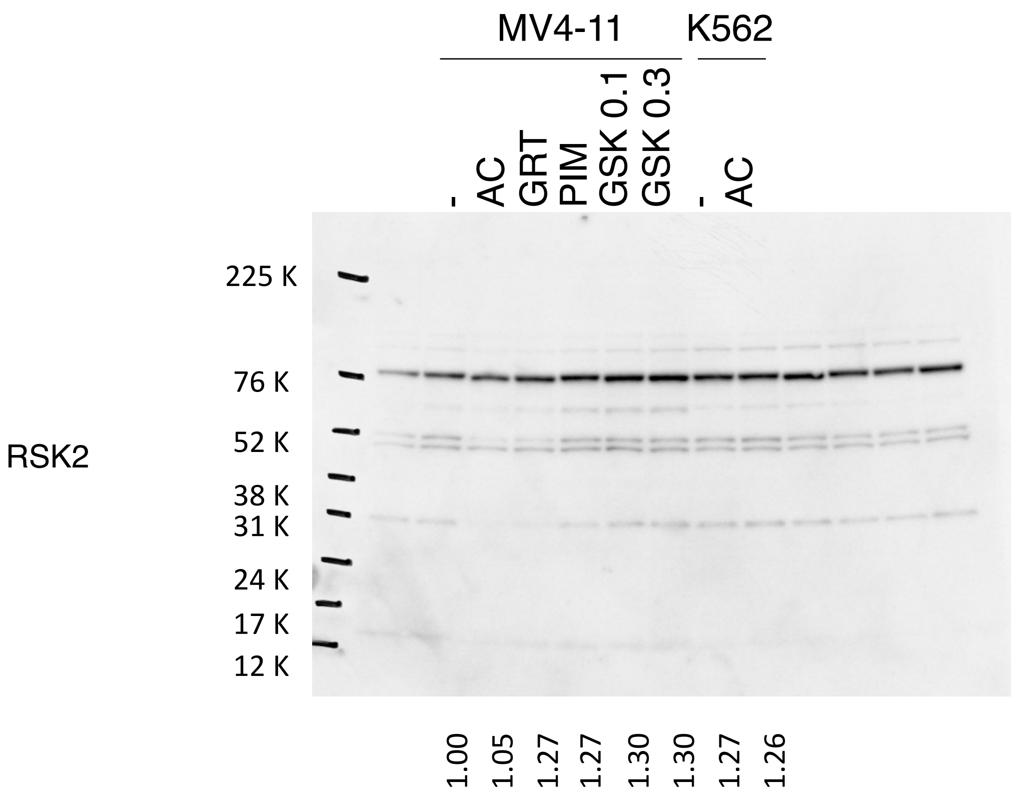
MV4-11
K562
-
AC
GRT
PIM
GSK 0.1
GSK 0.3
-
AC
225 K
76 K
52 K
38 K
31 K
24 K
17 K
12 K
RSK2
1.00
1.05
1.27
1.27
1.30
1.30
1.27
1.26

Figure 3G

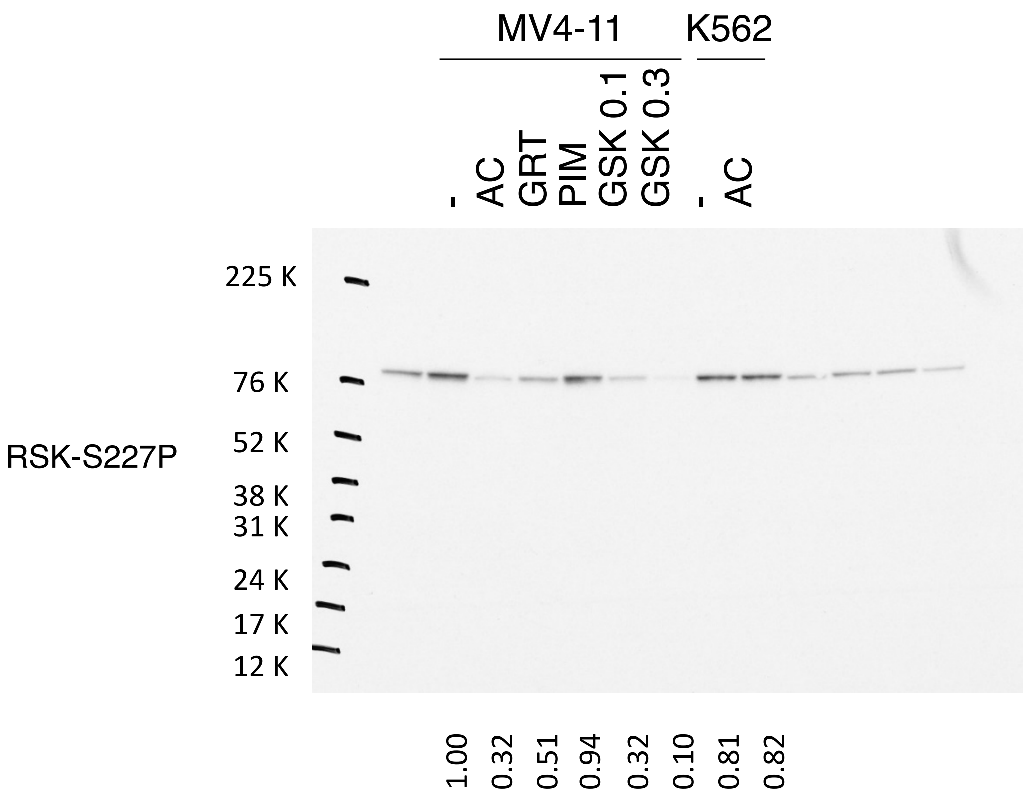
MV4-11
K562
-
AC
GRT
PIM
GSK 0.1
GSK 0.3
-
AC
225 K
76 K
52 K
38 K
31 K
24 K
17 K
12 K
RSK-S227P
1.00
0.32
0.51
0.94
0.32
0.10
0.81
0.82

Figure 3G

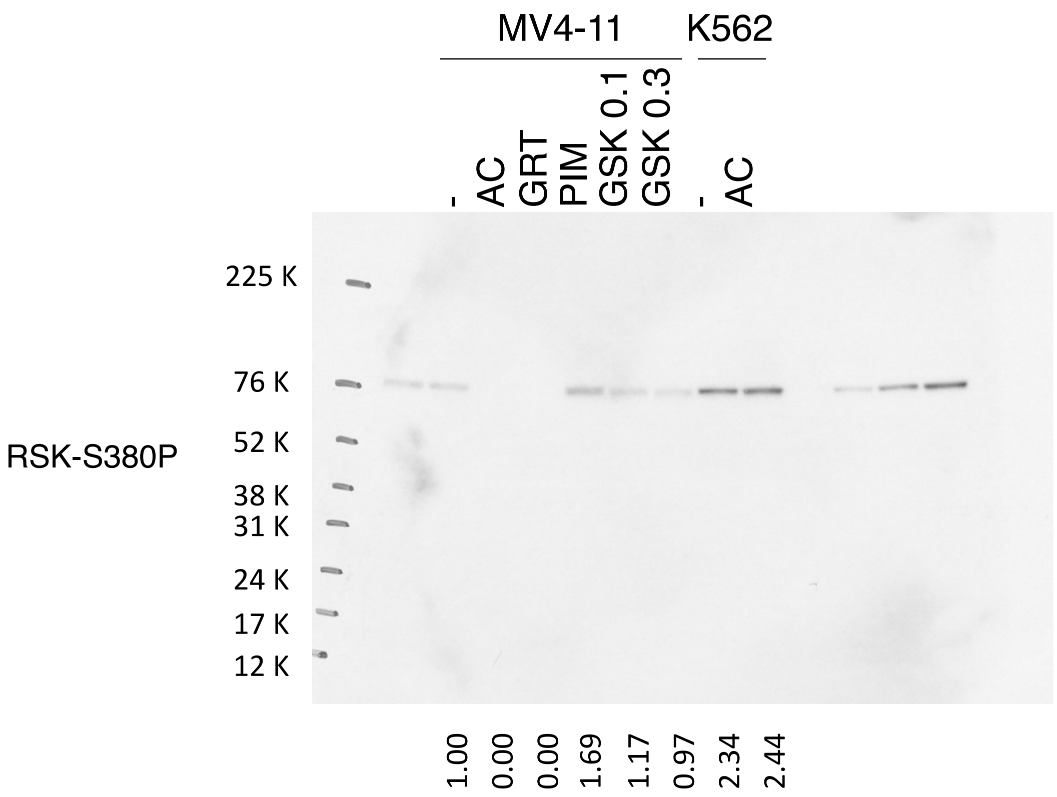
MV4-11
K562
-
AC
GRT
PIM
GSK 0.1
GSK 0.3
-
AC
225 K
76 K
52 K
38 K
31 K
24 K
17 K
12 K
RSK-S380P
1.00
0.00
0.00
1.69
1.17
0.97
2.34
2.44

Figure 3G

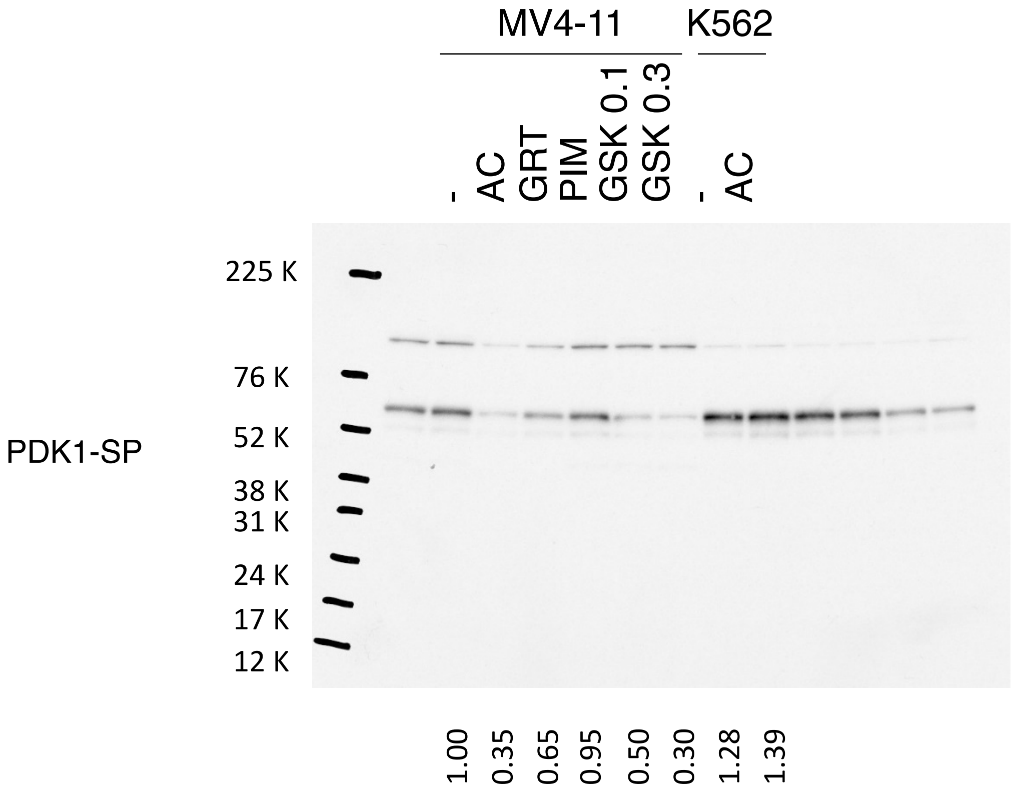
MV4-11
K562
-
AC
GRT
PIM
GSK 0.1
GSK 0.3
-
AC
225 K
76 K
52 K
38 K
31 K
24 K
17 K
12 K
PDK1-SP
1.00
0.35
0.65
0.95
0.50
0.30
1.28
1.39

Figure 3G

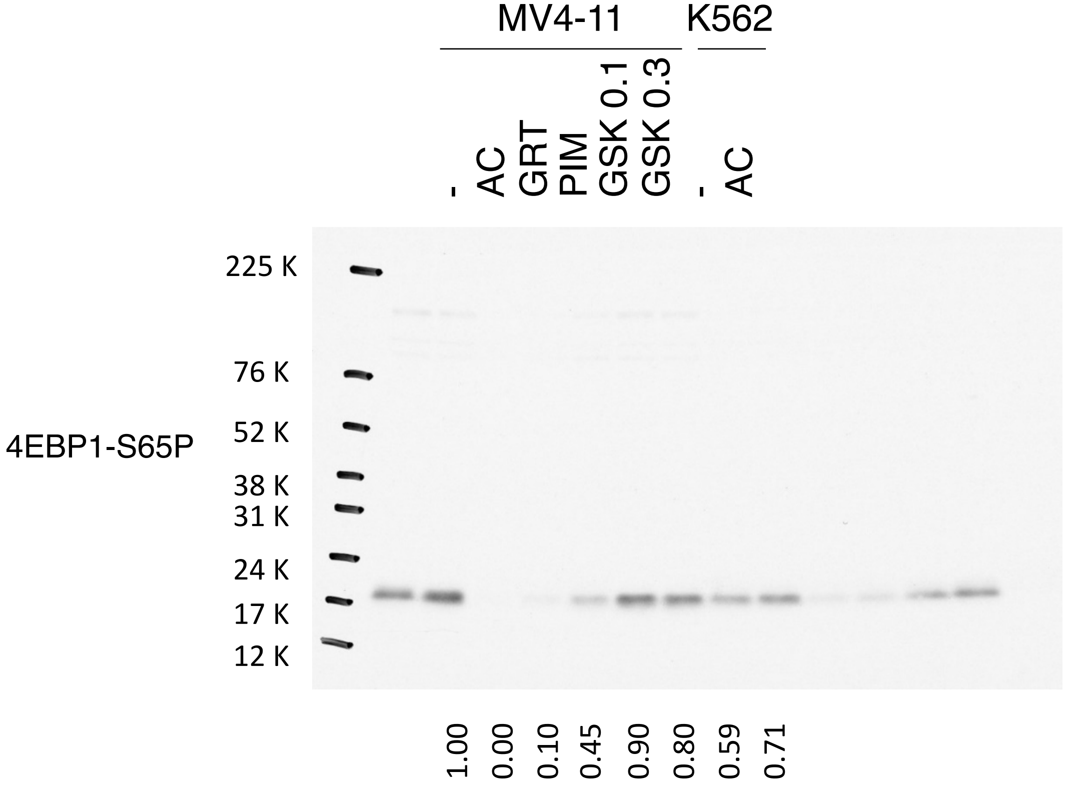
MV4-11
K562
-
AC
GRT
PIM
GSK 0.1
GSK 0.3
-
AC
225 K
76 K
52 K
38 K
31 K
24 K
17 K
12 K
4EBP1-S65P
1.00
0.00
0.10
0.45
0.90
0.80
0.59
0.71

Figure 3G

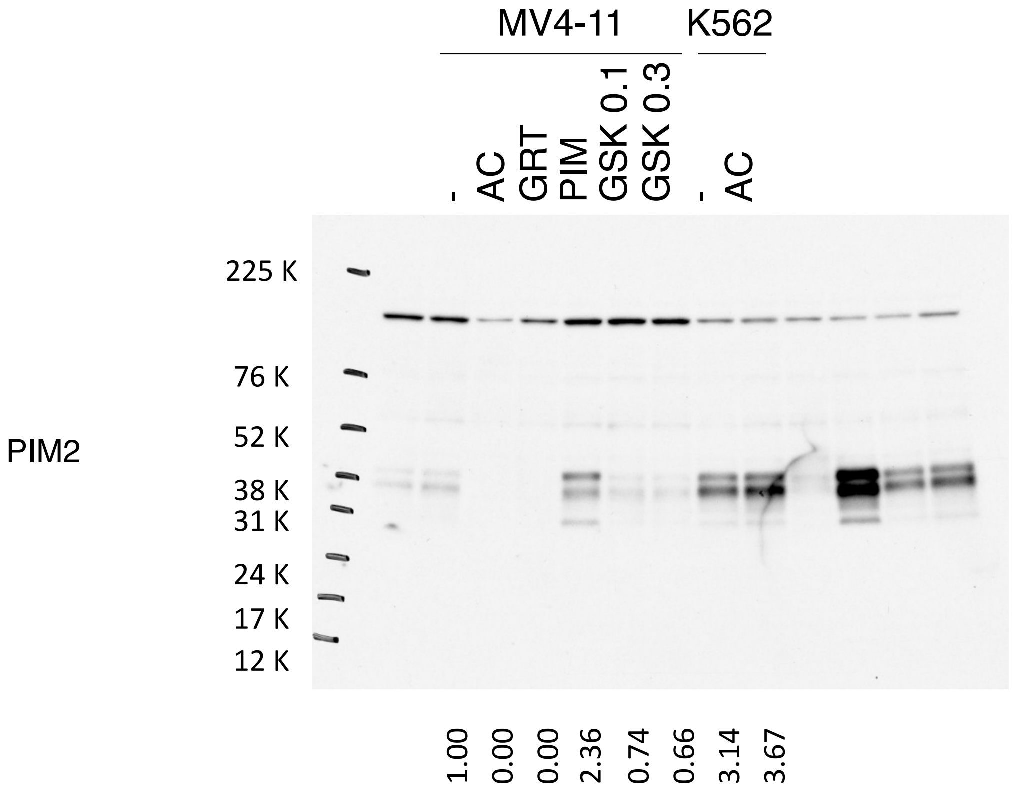
MV4-11
K562
-
AC
GRT
PIM
GSK 0.1
GSK 0.3
-
AC
225 K
76 K
52 K
38 K
31 K
24 K
17 K
12 K
PIM2
1.00
0.00
0.00
2.36
0.74
0.66
3.14
3.67

Figure 3G

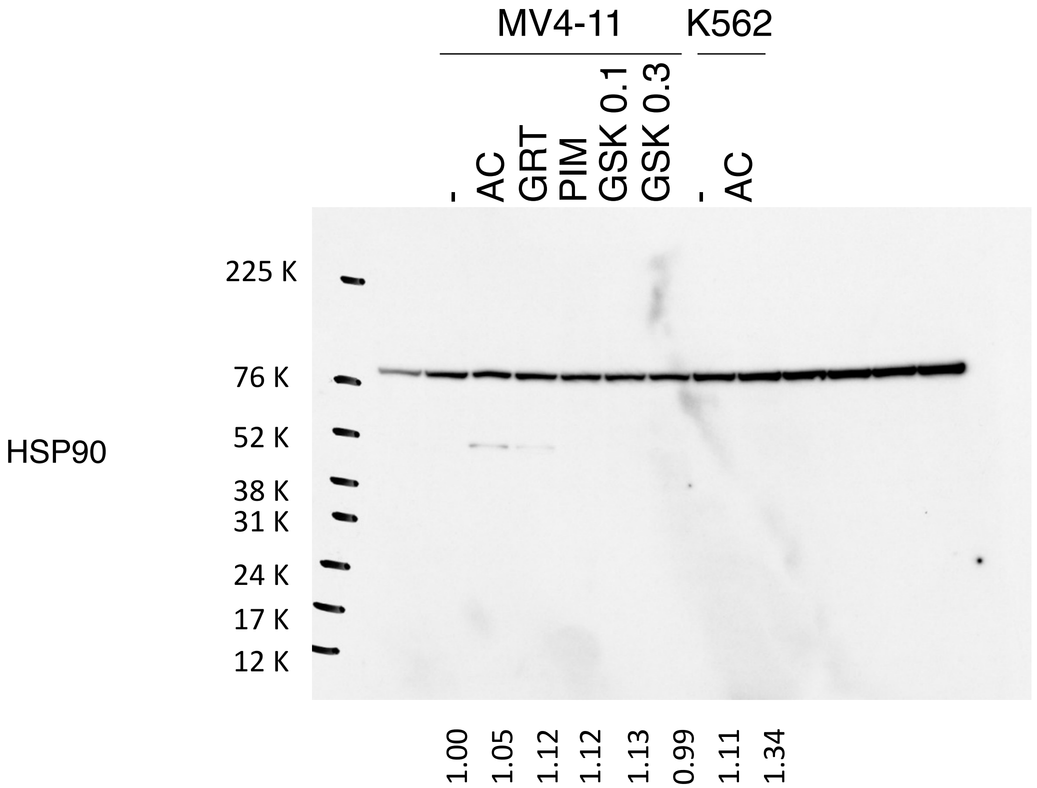
MV4-11
K562
-
AC
GRT
PIM
GSK 0.1
GSK 0.3
-
AC
225 K
76 K
52 K
38 K
31 K
24 K
17 K
12 K
HSP90
1.00
1.05
1.12
1.12
1.13
0.99
1.11
1.34

Figure 3J

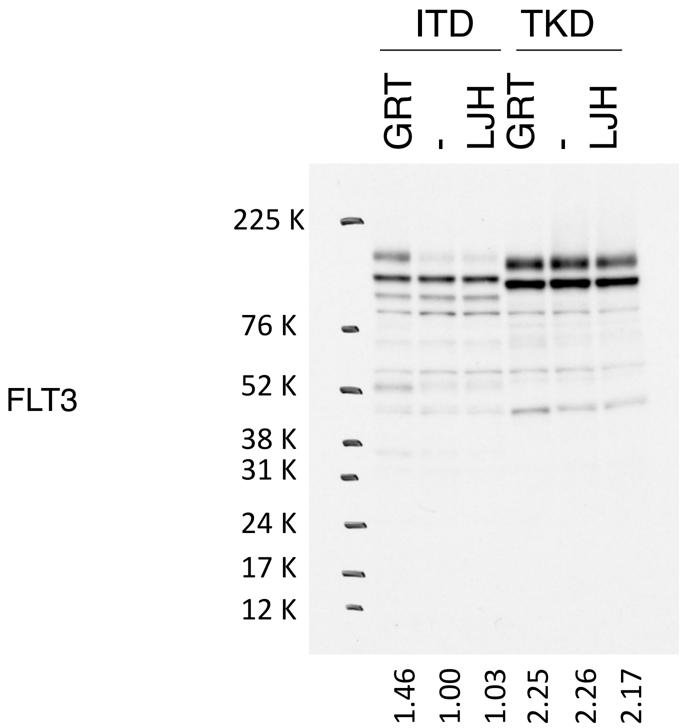
FLT3
ITD
TKD
GRT
-
LJH
GRT
-
LJH
225 K
76 K
52 K
38 K
31 K
24 K
17 K
12 K
1.46
1.00
1.03
2.25
2.26
2.17

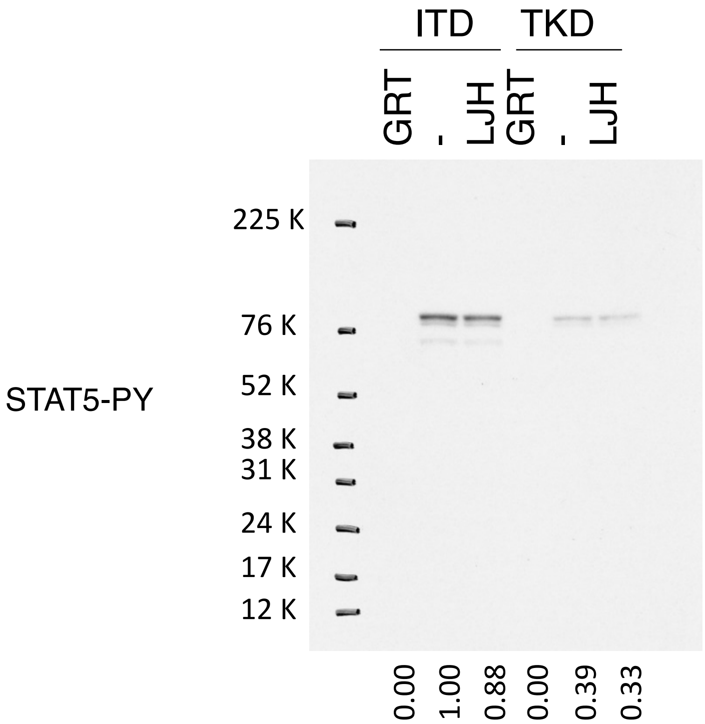
STAT5-PY
ITD
TKD
GRT
-
LJH
GRT
-
LJH
225 K
76 K
52 K
38 K
31 K
24 K
17 K
12 K
0.00
1.00
0.88
0.00
0.39
0.33

Figure 3J

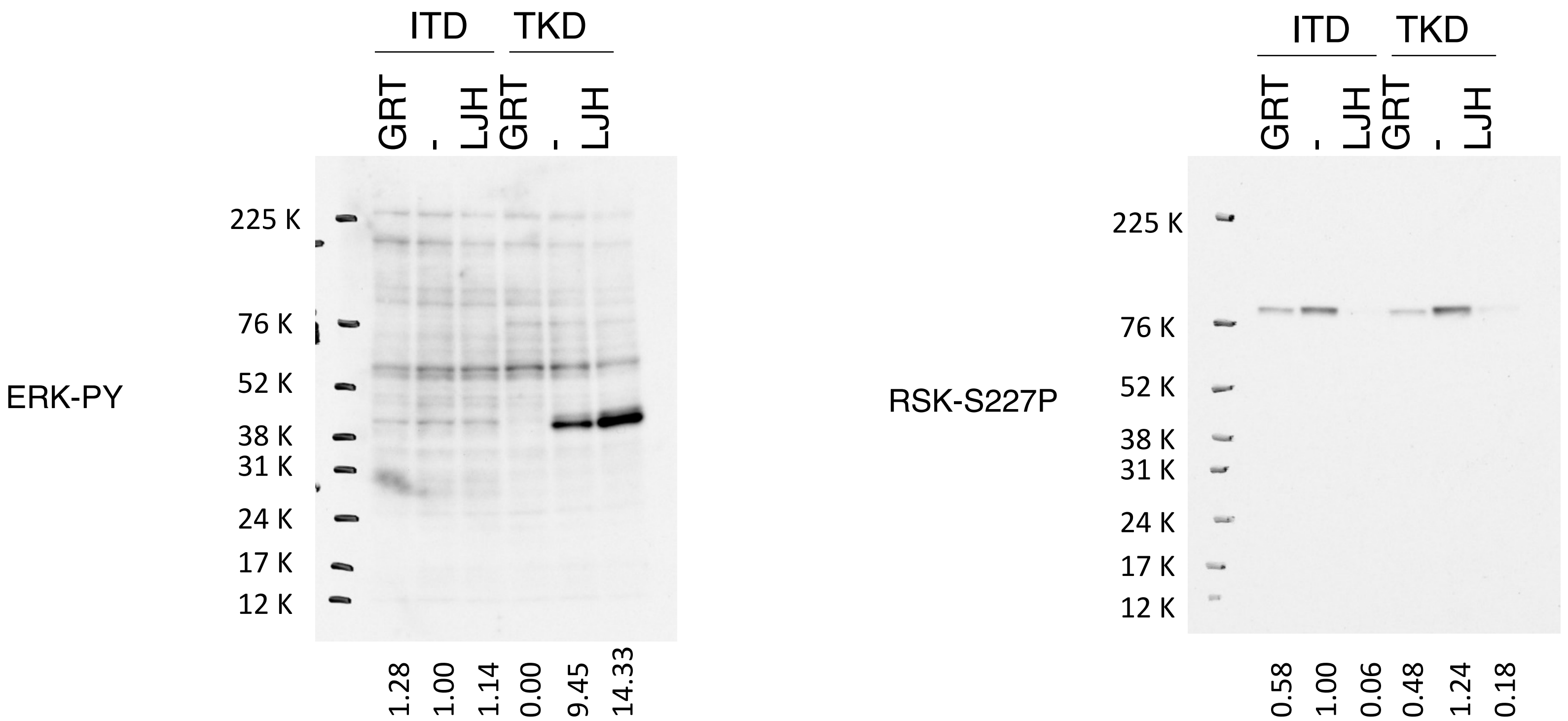
ITD
TKD
GRT
-
LJH
GRT
-
LJH
225 K
76 K
52 K
38 K
31 K
24 K
17 K
12 K
ERK-PY
1.28
1.00
1.14
0.00
9.45
14.33
ITD
TKD
GRT
-
LJH
GRT
-
LJH
225 K
76 K
52 K
38 K
31 K
24 K
17 K
12 K
RSK-S227P
0.58
1.00
0.06
0.48
1.24
0.18

Figure 3J

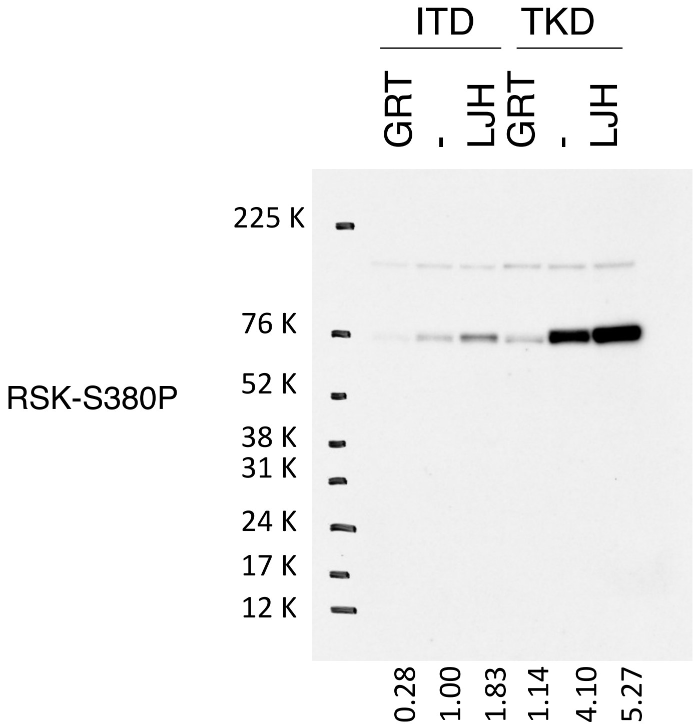

ITD
TKD
GRT
-
LJH
GRT
-
LJH
225 K
76 K
52 K
RSK-S380P
38 K
31 K
24 K
17 K
12 K
0.28
1.00
1.83
1.14
4.10
5.27

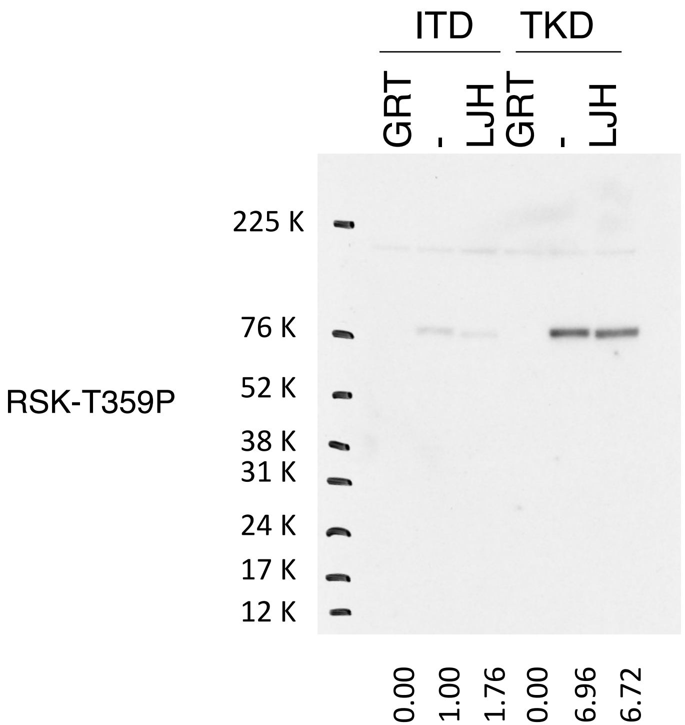

ITD
TKD
GRT
-
LJH
GRT
-
LJH
225 K
76 K
52 K
RSK-T359P
38 K
31 K
24 K
17 K
12 K
0.00
1.00
1.76
0.00
6.96
6.72

Figure 3J

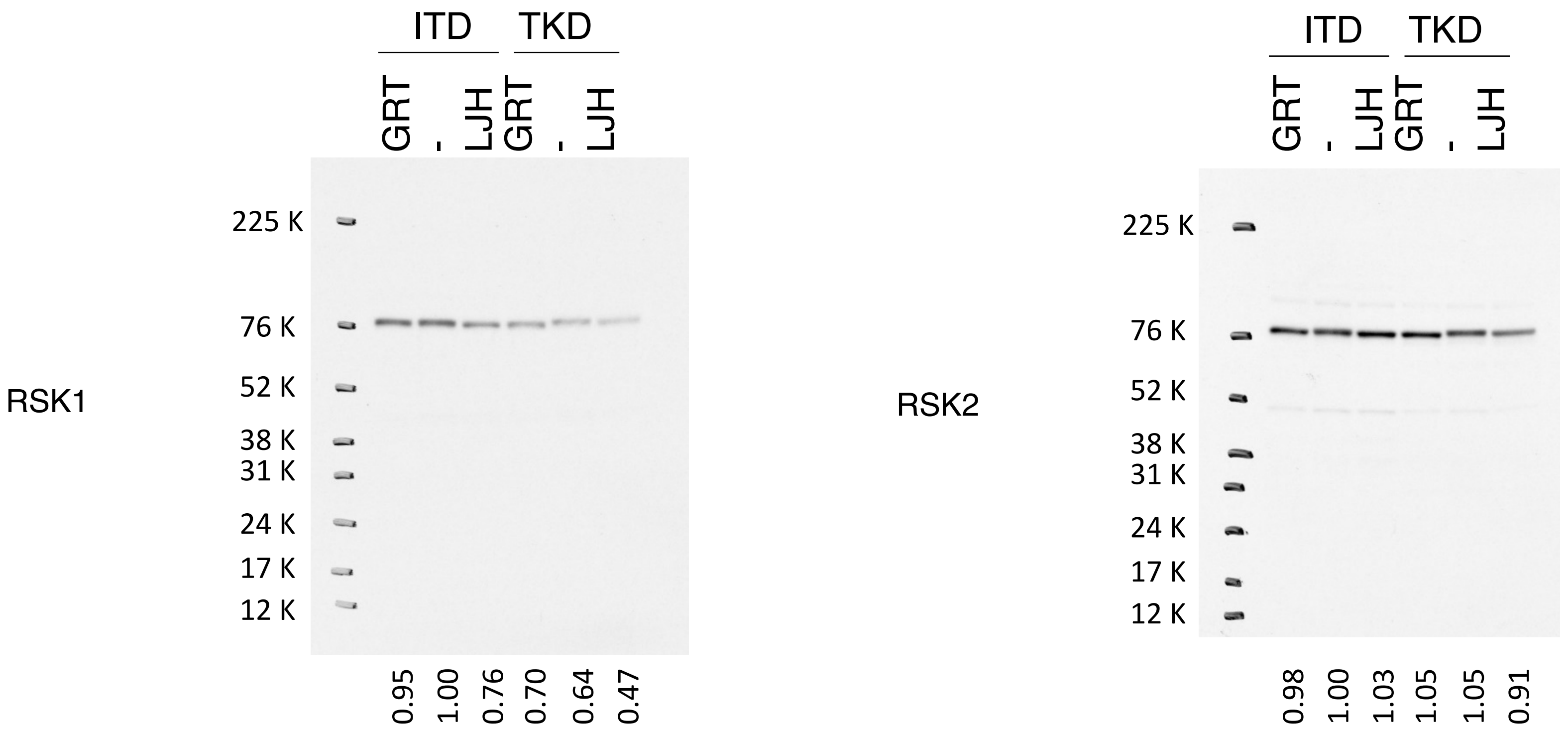
RSK1
ITD
TKD
GRT
-
LJH
GRT
-
LJH
225 K
76 K
52 K
38 K
31 K
24 K
17 K
12 K
0.95
1.00
0.76
0.70
0.64
0.47
RSK2
ITD
TKD
GRT
-
LJH
GRT
-
LJH
225 K
76 K
52 K
38 K
31 K
24 K
17 K
12 K
0.98
1.00
1.03
1.05
1.05
0.91

Figure 3J

HSP90

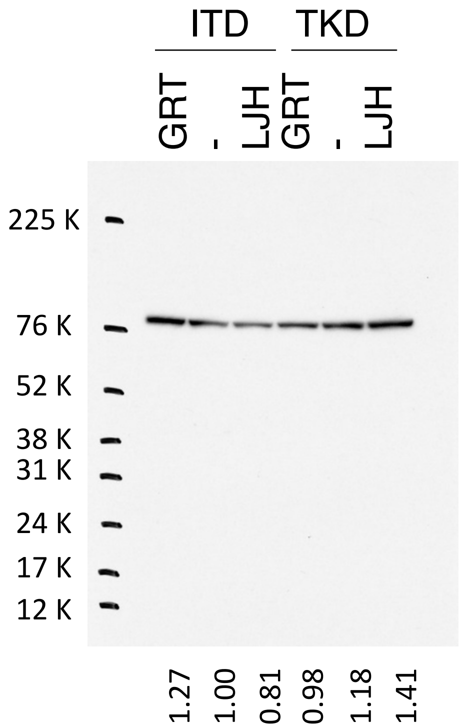

Figure 3H

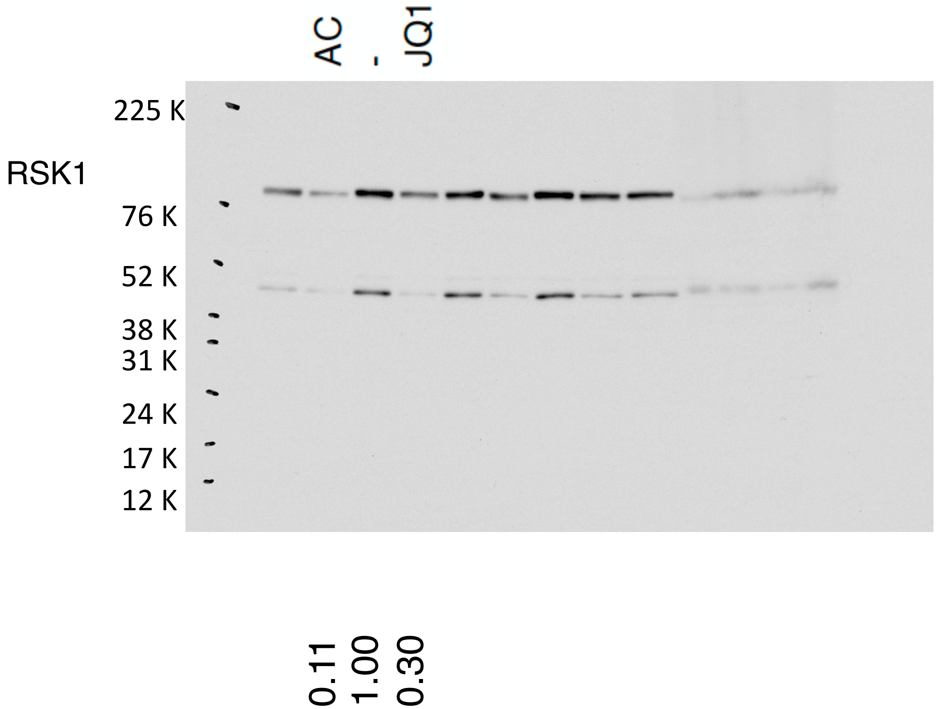
AC
-
JQ1
225 K
RSK1
76 K
52 K
38 K
31 K
24 K
17 K
12 K
0.11
1.00
0.30

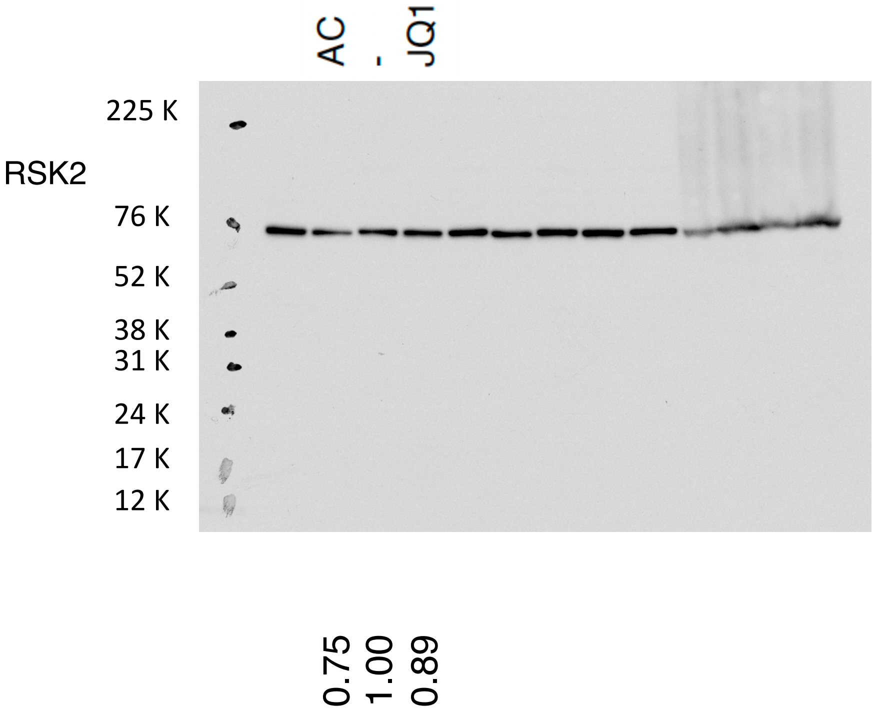
AC
-
JQ1
225 K
RSK2
76 K
52 K
38 K
31 K
24 K
17 K
12 K
0.75
1.00
0.89

Figure 3H

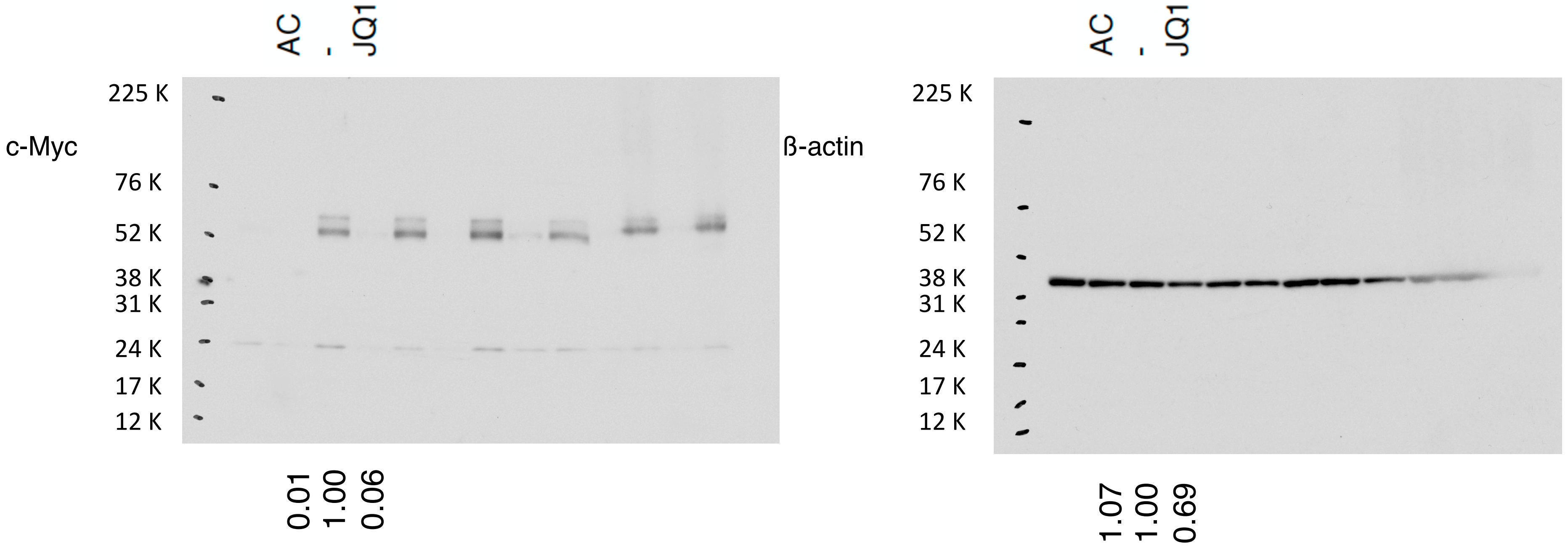
AC
-
JQ1
225 K
c-Myc
76 K
52 K
38 K
31 K
24 K
17 K
12 K
0.01
1.00
0.06
ß-actin
1.07
1.00
0.69

Figure 3I

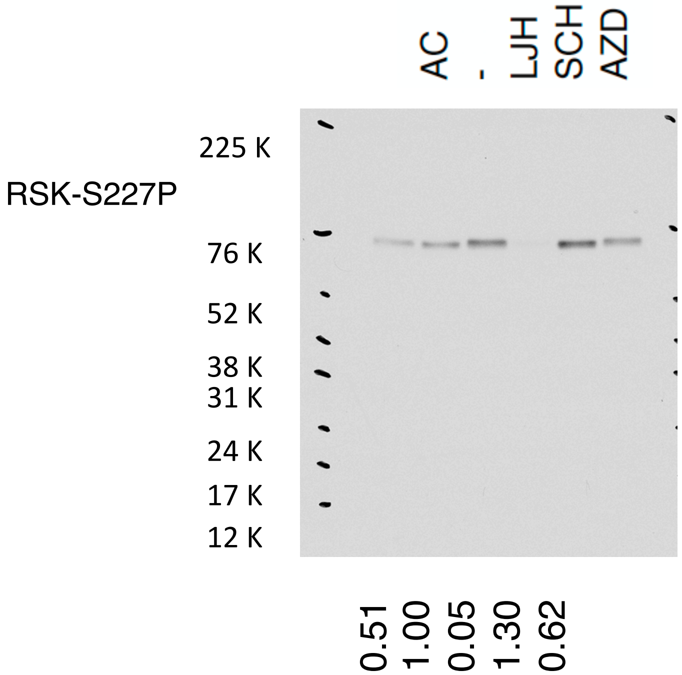

AC
-
LJH
SCH
AZD
225 K
RSK-S227P
76 K
52 K
38 K
31 K
24 K
17 K
12 K
0.51
1.00
0.05
1.30
0.62

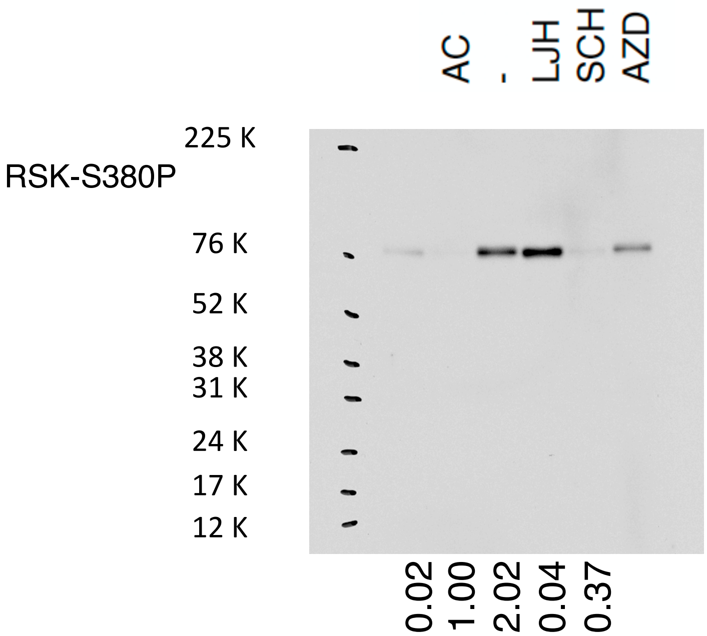

AC
-
LJH
SCH
AZD
225 K
RSK-S380P
76 K
52 K
38 K
31 K
24 K
17 K
12 K
0.02
1.00
2.02
0.04
0.37

Figure 3I

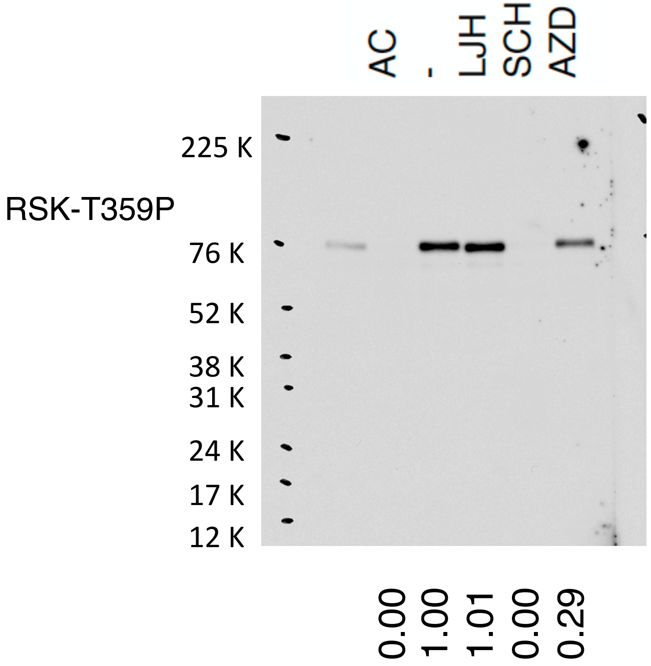

AC
-
LJH
SCH
AZD
225 K
RSK-T359P
76 K
52 K
38 K
31 K
24 K
17 K
12 K
0.00
1.00
1.01
0.00
0.29

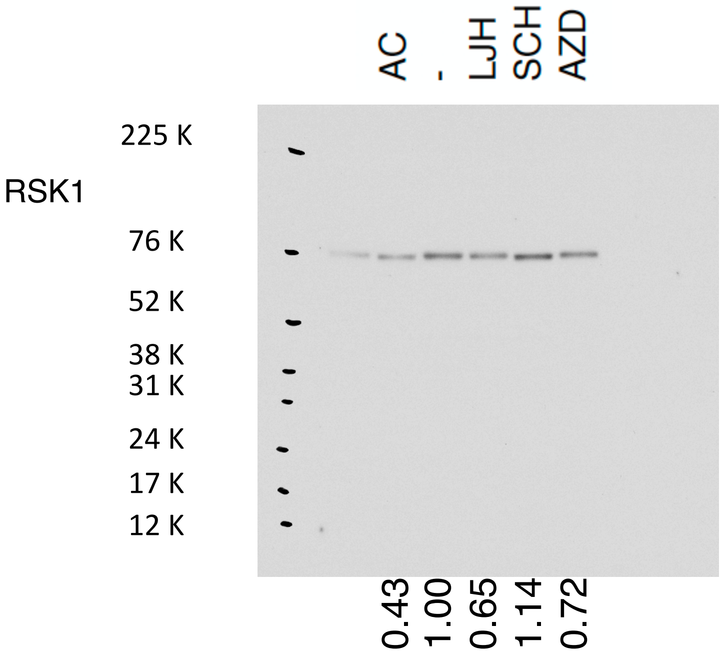

AC
-
LJH
SCH
AZD
225 K
RSK1
76 K
52 K
38 K
31 K
24 K
17 K
12 K
0.43
1.00
0.65
1.14
0.72

Figure 3I

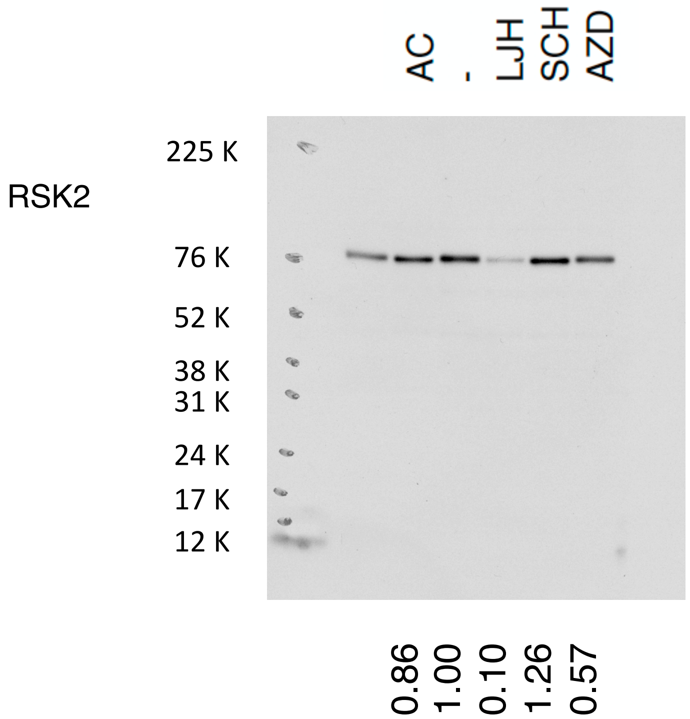
AC
-
LJH
SCH
AZD
225 K
RSK2
76 K
52 K
38 K
31 K
24 K
17 K
12 K
0.86
1.00
0.10
1.26
0.57

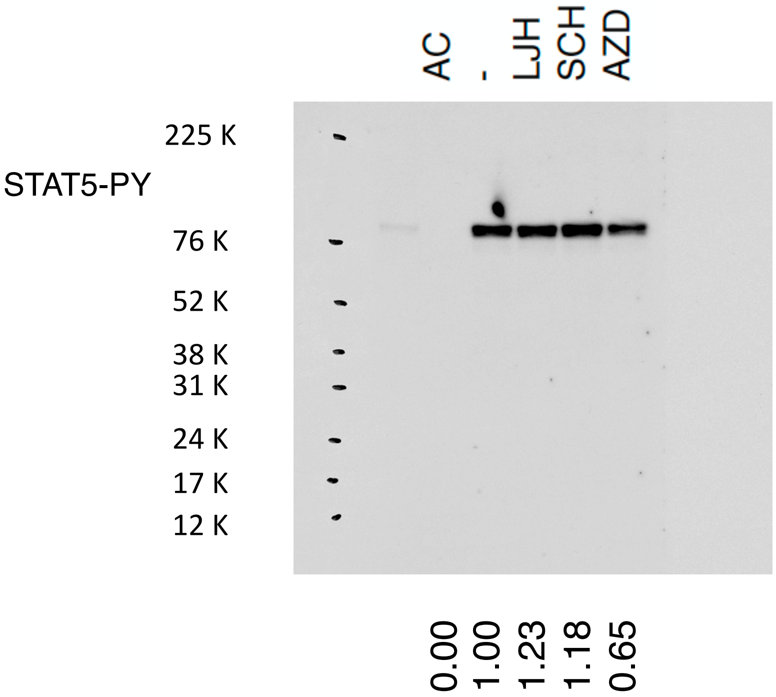
AC
-
LJH
SCH
AZD
225 K
STAT5-PY
76 K
52 K
38 K
31 K
24 K
17 K
12 K
0.00
1.00
1.23
1.18
0.65

Figure 3I

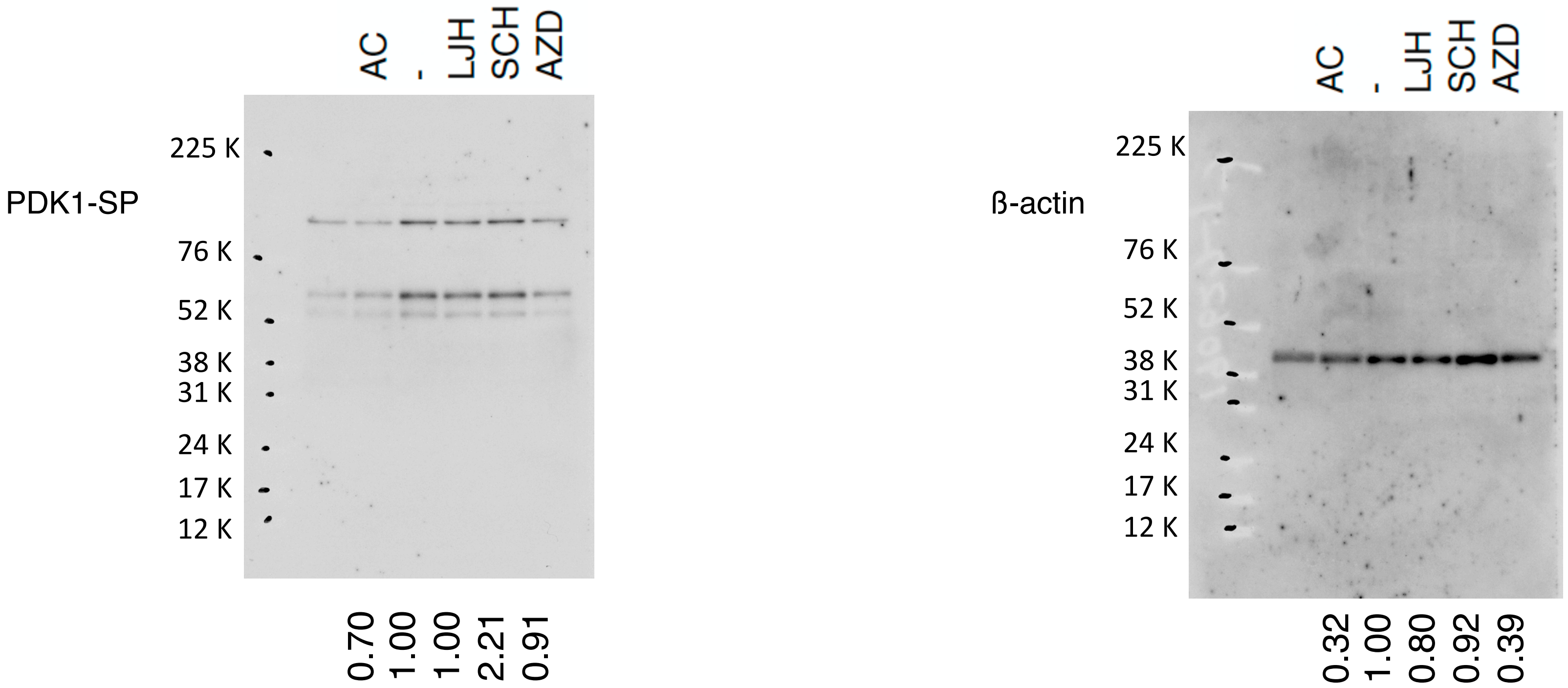

PDK1-SP
AC
-
LJH
SCH
AZD
225 K
76 K
52 K
38 K
31 K
24 K
17 K
12 K
0.70
1.00
1.00
2.21
0.91
ß-actin
AC
-
LJH
SCH
AZD
225 K
76 K
52 K
38 K
31 K
24 K
17 K
12 K
0.32
1.00
0.80
0.92
0.39

Figure 4A

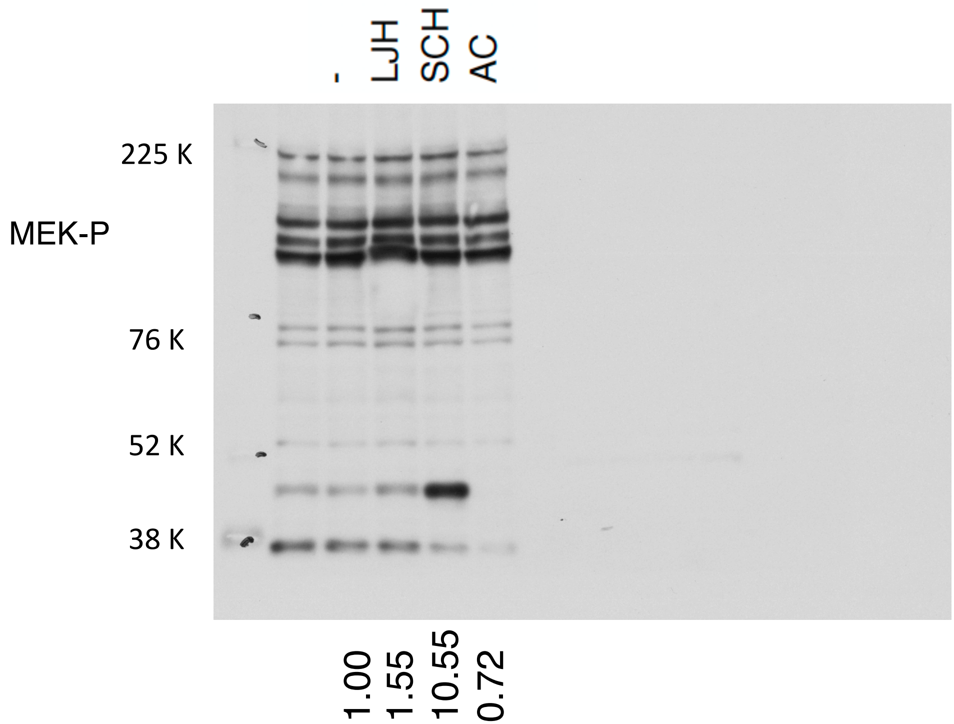

-
LJH
SCH
AC
225 K
MEK-P
76 K
52 K
38 K
1.00
1.55
10.55
0.72

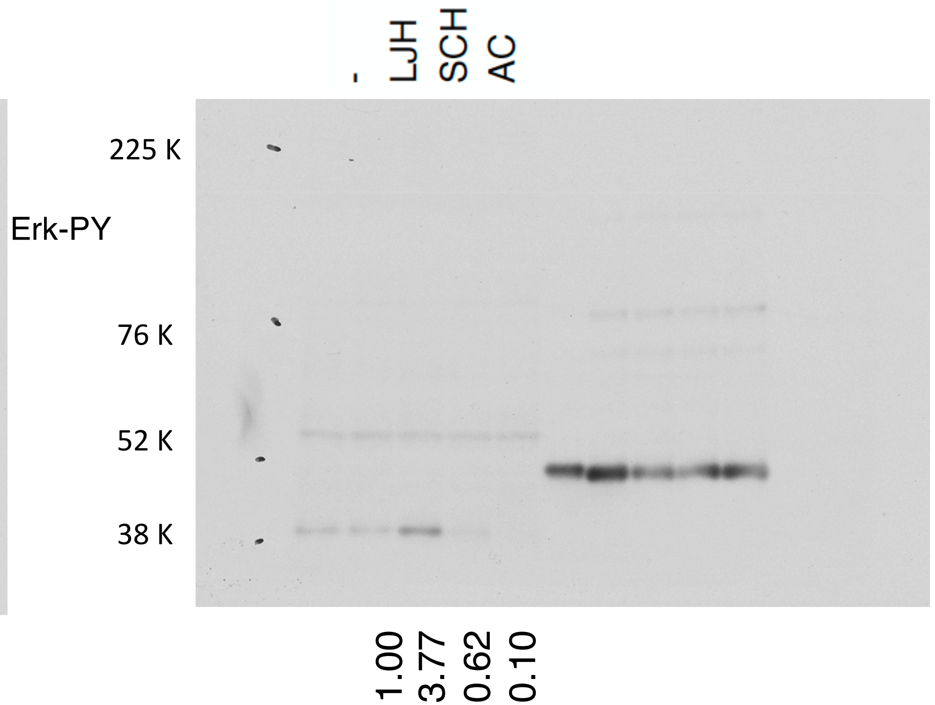

-
LJH
SCH
AC
225 K
Erk-PY
76 K
52 K
38 K
1.00
3.77
0.62
0.10

Figure 4A

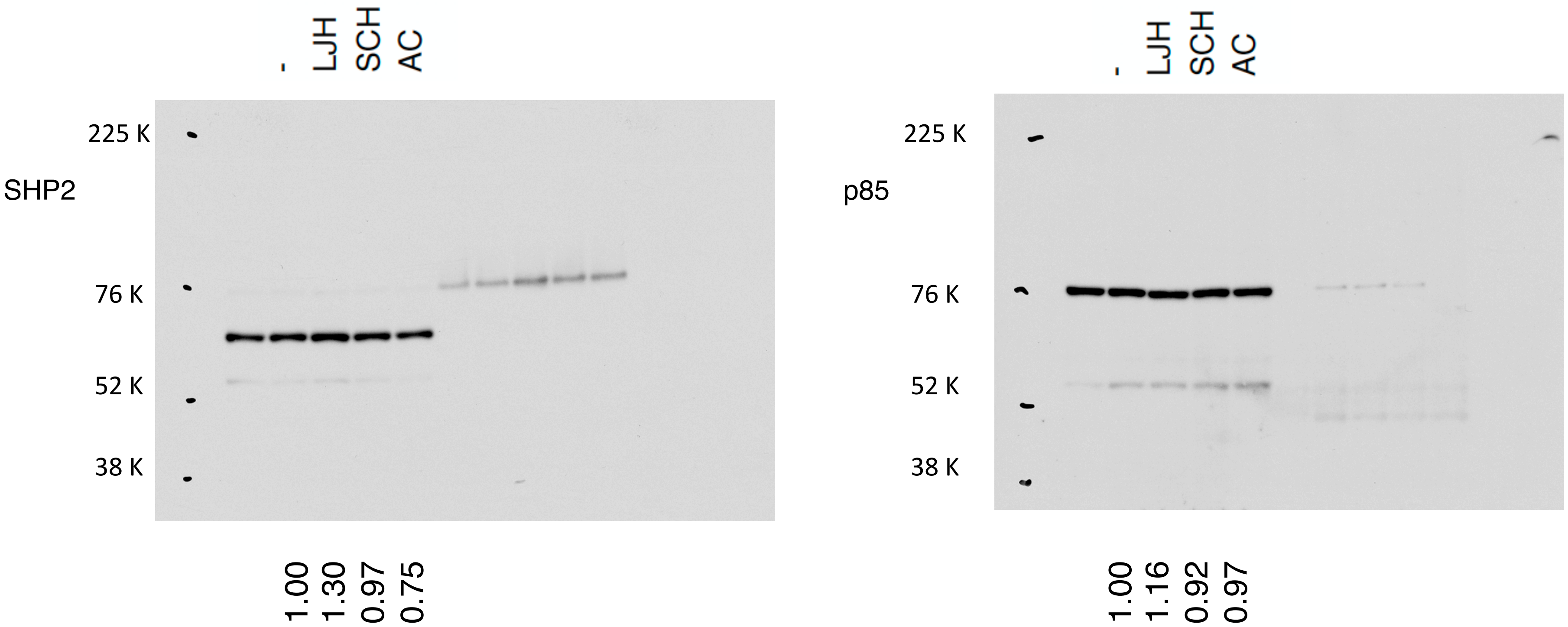

-
LJH
SCH
AC
225 K
SHP2
76 K
52 K
38 K
1.00
1.30
0.97
0.75
-
LJH
SCH
AC
225 K
p85
76 K
52 K
38 K
1.00
1.16
0.92
0.97

Figure 4A

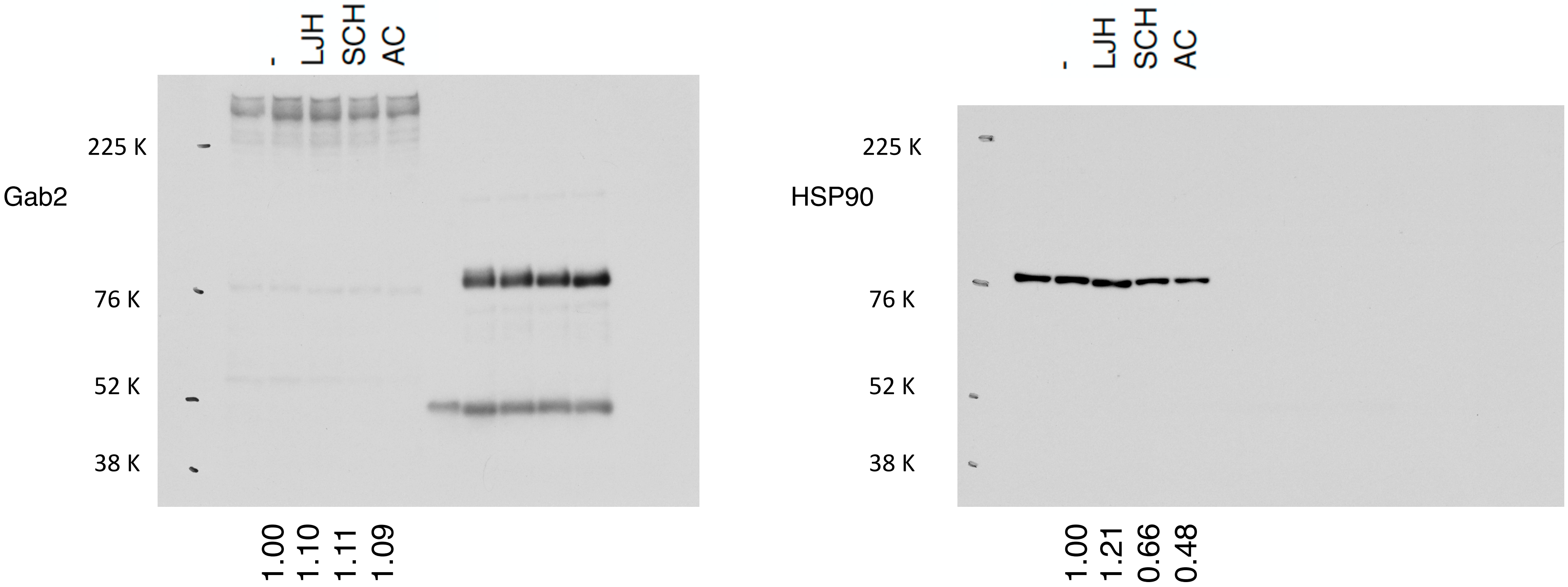
- LJH SCH AC
Gab2
225 K
76 K
52 K
38 K
1.00 1.10 1.11 1.09
- LJH SCH AC
HSP90
225 K
76 K
52 K
38 K
1.00 1.21 0.66 0.48

Figure 4B

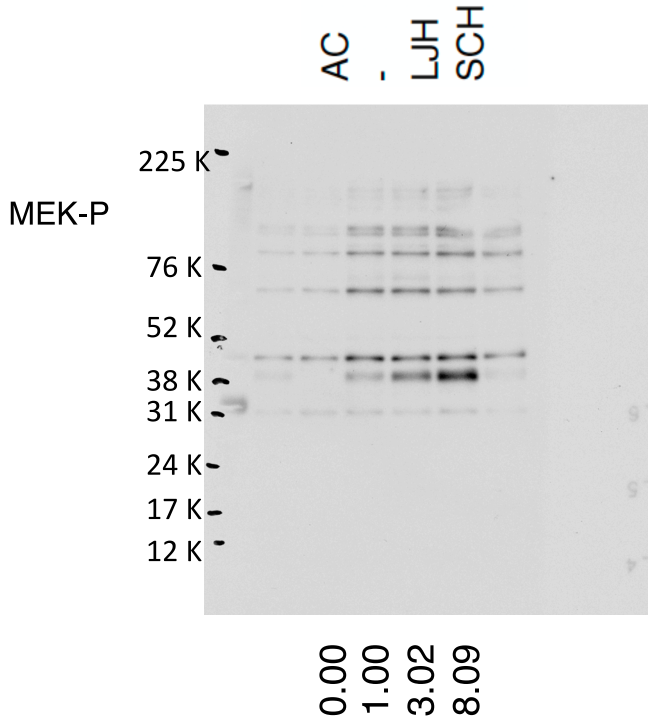

AC
-
LJH
SCH
MEK-P
225 K
76 K
52 K
38 K
31 K
24 K
17 K
12 K
0.00
1.00
3.02
8.09

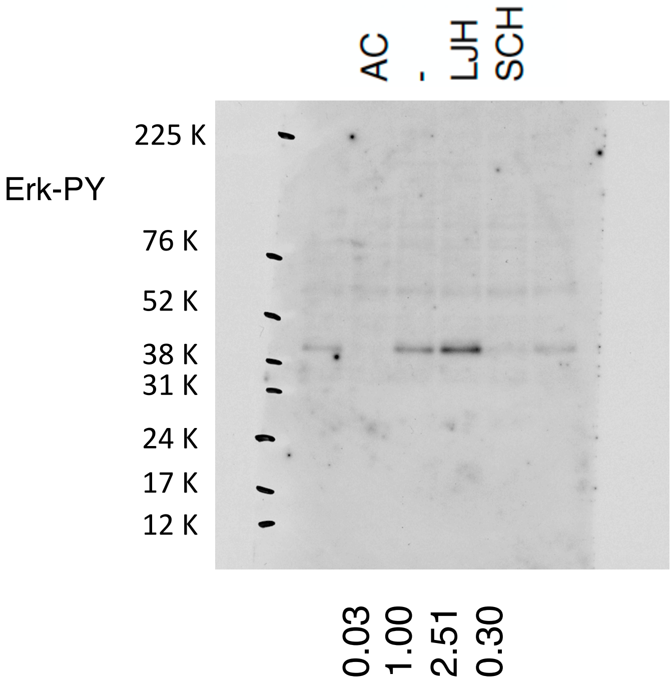

AC
-
LJH
SCH
Erk-PY
225 K
76 K
52 K
38 K
31 K
24 K
17 K
12 K
0.03
1.00
2.51
0.30

Figure 4B

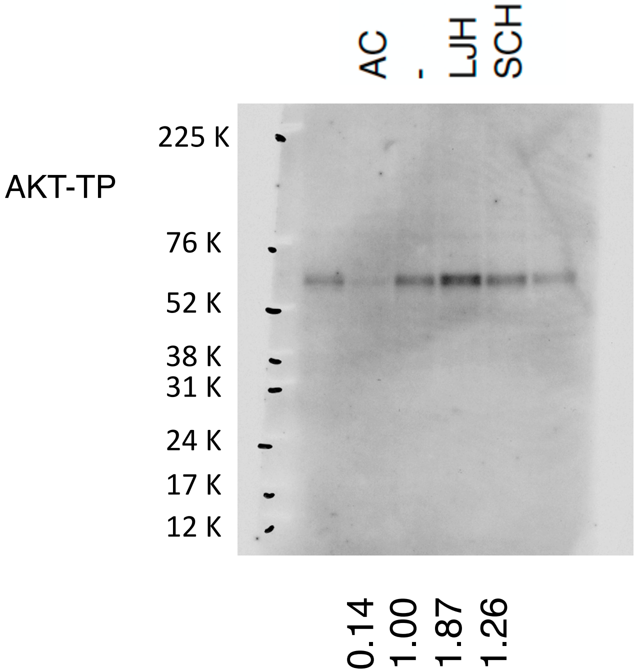
AC
-
LJH
SCH
225 K
AKT-TP
76 K
52 K
38 K
31 K
24 K
17 K
12 K
0.14
1.00
1.87
1.26

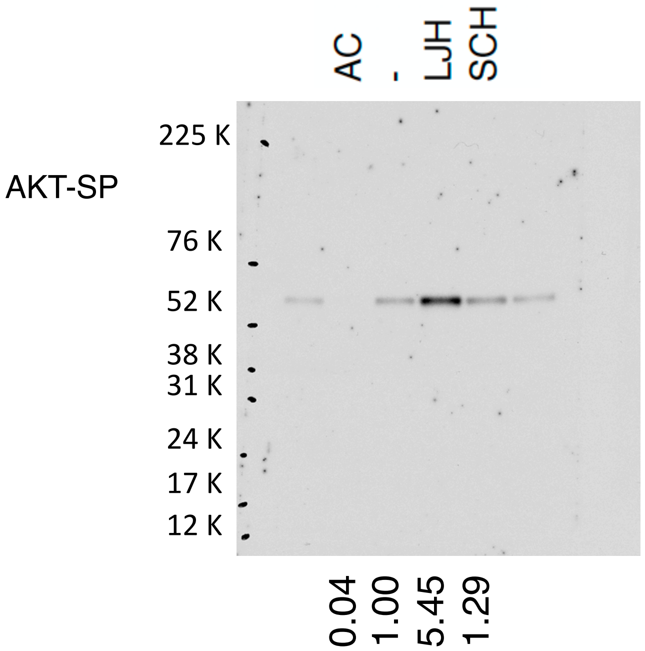
AC
-
LJH
SCH
225 K
AKT-SP
76 K
52 K
38 K
31 K
24 K
17 K
12 K
0.04
1.00
5.45
1.29

Figure 4B

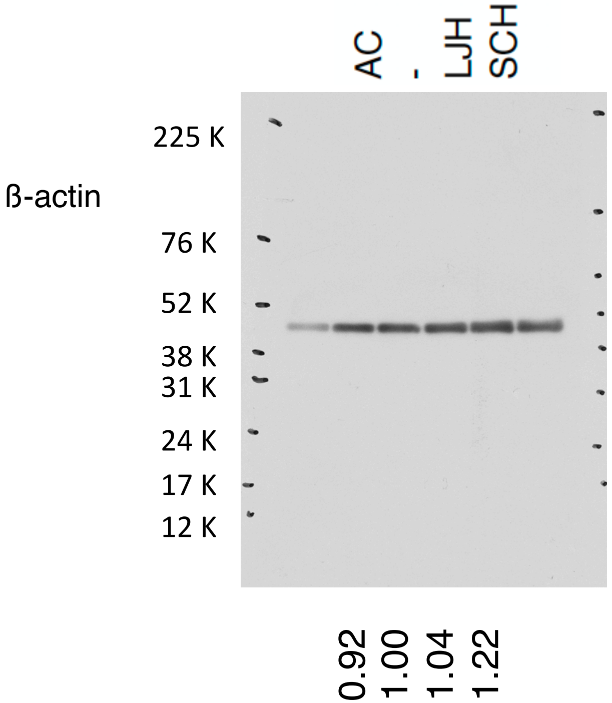
ß-actin
AC
-
LJH
SCH
225 K
76 K
52 K
38 K
31 K
24 K
17 K
12 K
0.92
1.00
1.04
1.22

Figure 4C

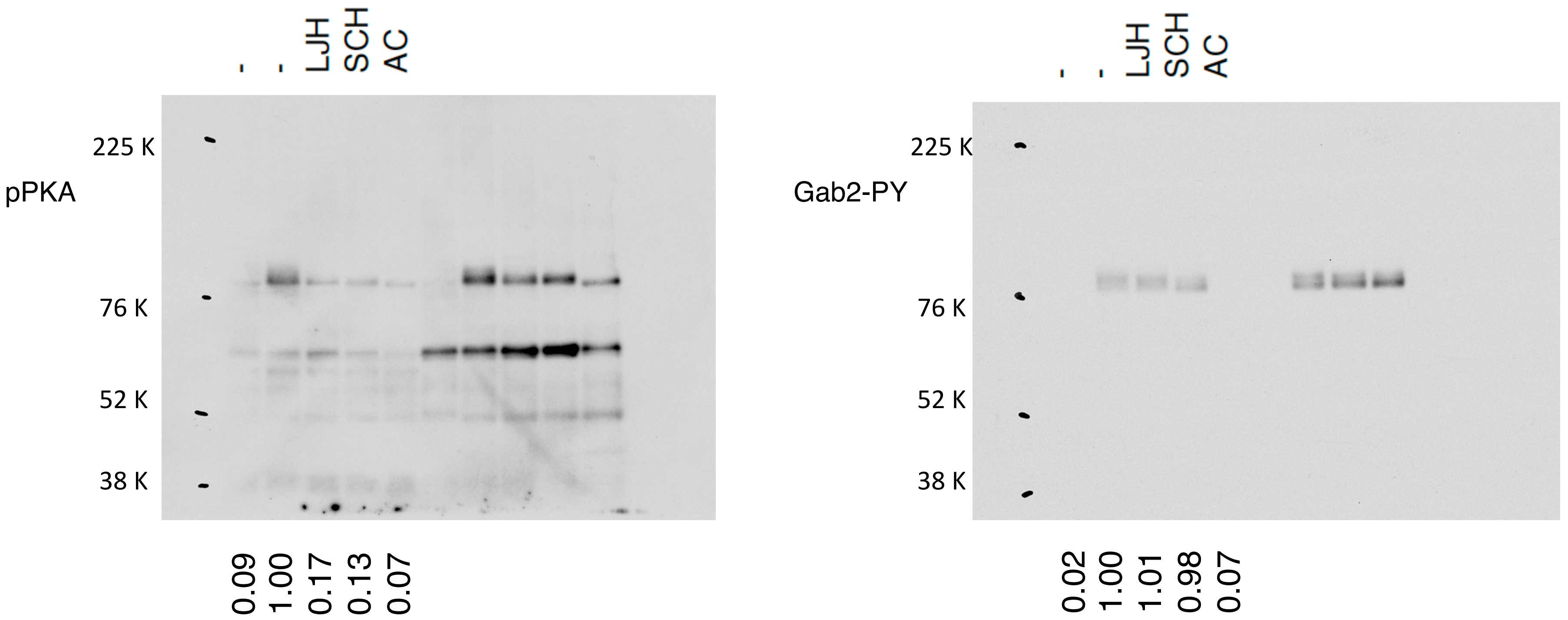

pPKA
225 K
76 K
52 K
38 K
-
-
LJH
SCH
AC
0.09
1.00
0.17
0.13
0.07
Gab2-PY
225 K
76 K
52 K
38 K
-
-
LJH
SCH
AC
0.02
1.00
1.01
0.98
0.07

Figure 4C

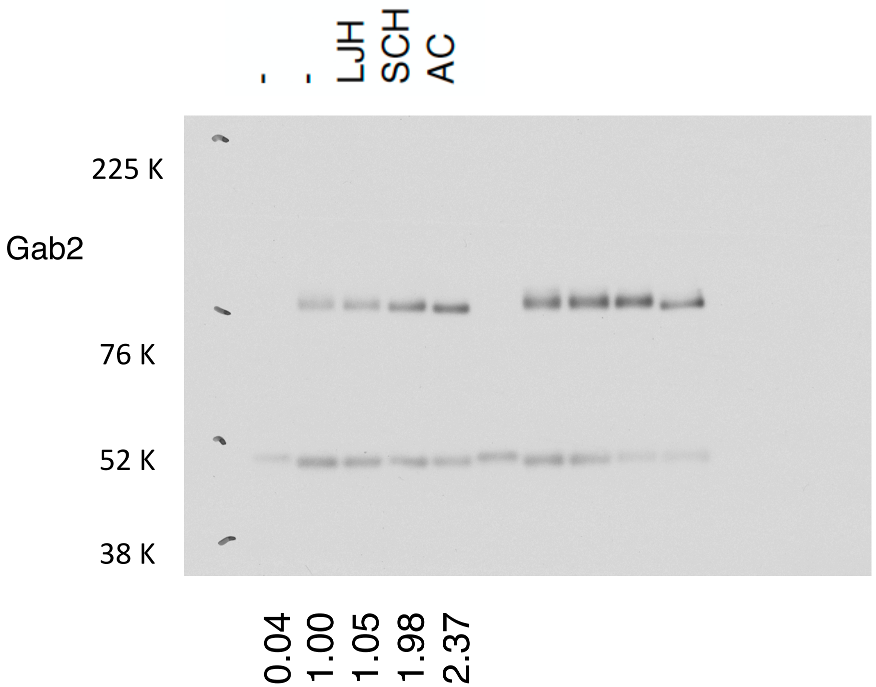

Figure 4D

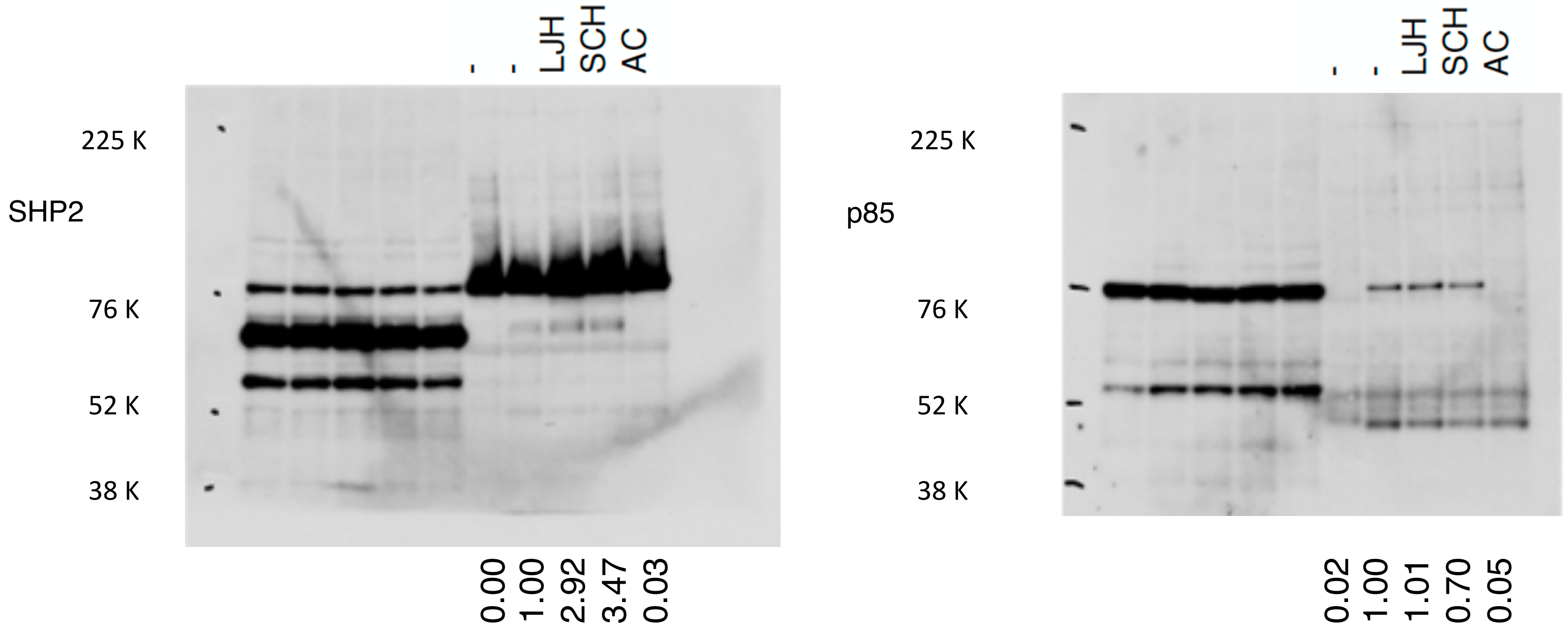

SHP2
225 K
76 K
52 K
38 K
-
-
LJH
SCH
AC
0.00
1.00
2.92
3.47
0.03
p85
225 K
76 K
52 K
38 K
-
-
LJH
SCH
AC
0.02
1.00
1.01
0.70
0.05

Figure 4D

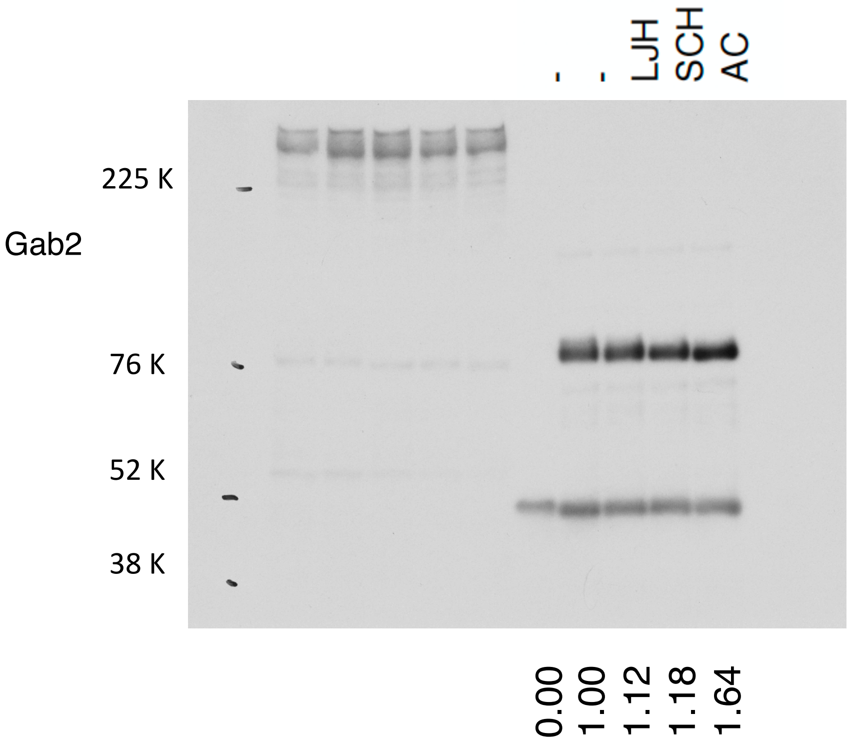

Figure 4E

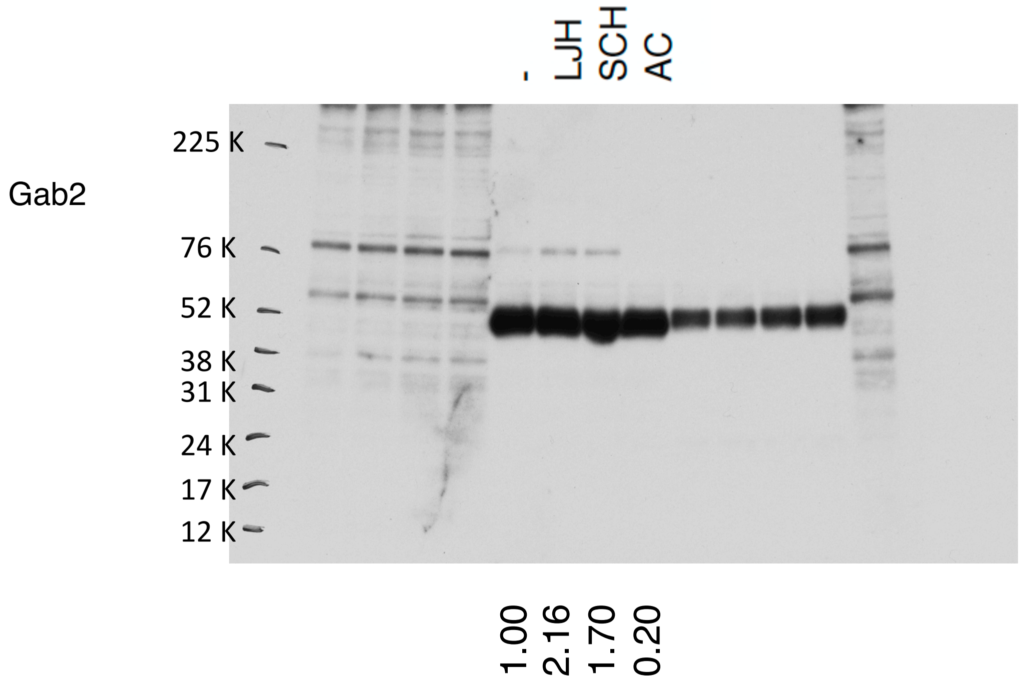

Gab2
-
LJH
SCH
AC
225 K
76 K
52 K
38 K
31 K
24 K
17 K
12 K
1.00
2.16
1.70
0.20

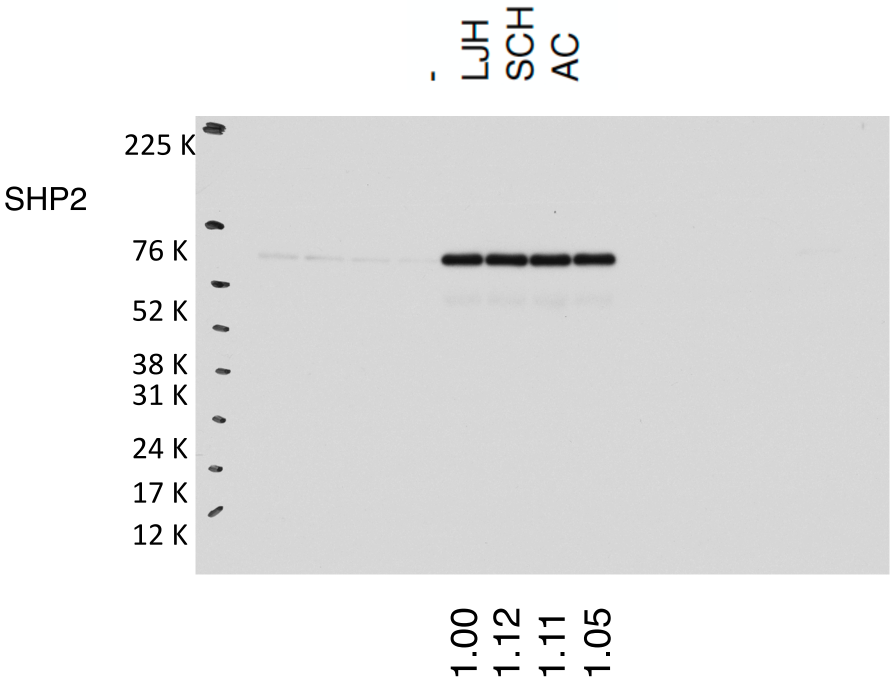

SHP2
-
LJH
SCH
AC
225 K
76 K
52 K
38 K
31 K
24 K
17 K
12 K
1.00
1.12
1.11
1.05

Figure 4F

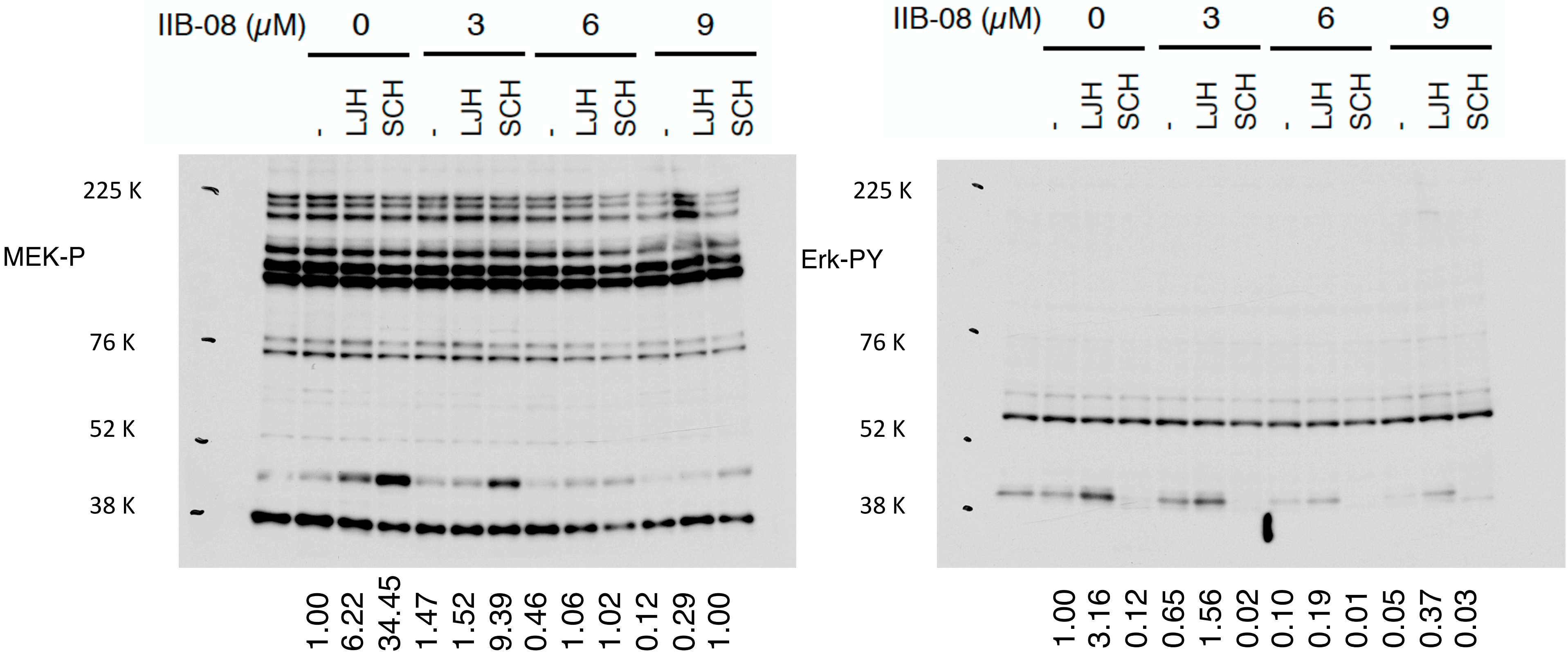
IIB-08 (μM) 0 3 6 9
- LJH SCH - LJH SCH - LJH SCH - LJH SCH
225 K
MEK-P
76 K
52 K
38 K
1.00 6.22 34.45 1.47 1.52 9.39 0.46 1.06 1.02 0.12 0.29 1.00
IIB-08 (μM) 0 3 6 9
- LJH SCH - LJH SCH - LJH SCH - LJH SCH
225 K
Erk-PY
76 K
52 K
38 K
1.00 3.16 0.12 0.65 1.56 0.02 0.10 0.19 0.01 0.05 0.37 0.03

Figure 4F

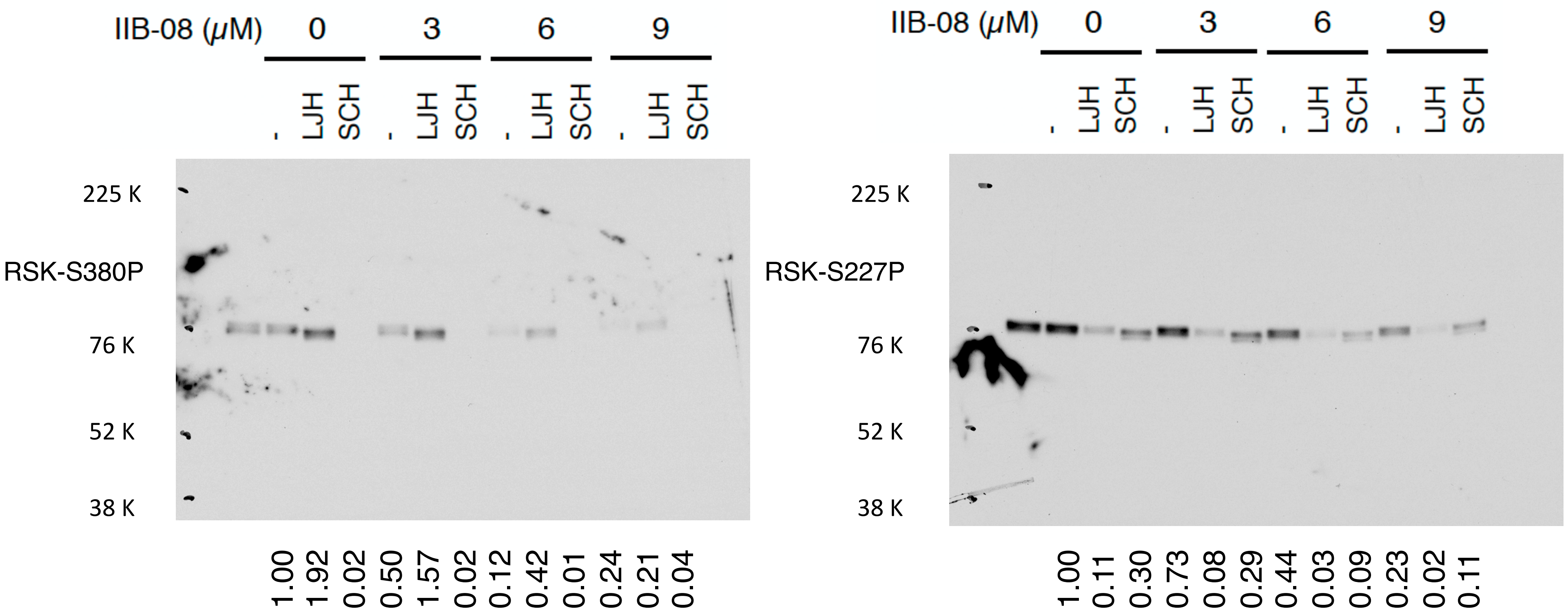
IIB-08 (μM)
0
3
6
9
-
LJH
SCH
225 K
RSK-S380P
76 K
52 K
38 K
1.00 1.92 0.02 0.50 1.57 0.02 0.12 0.42 0.01 0.24 0.21 0.04
IIB-08 (μM)
0
3
6
9
-
LJH
SCH
225 K
RSK-S227P
76 K
52 K
38 K
1.00 0.11 0.30 0.73 0.08 0.29 0.44 0.03 0.09 0.23 0.02 0.11

Figure 4F

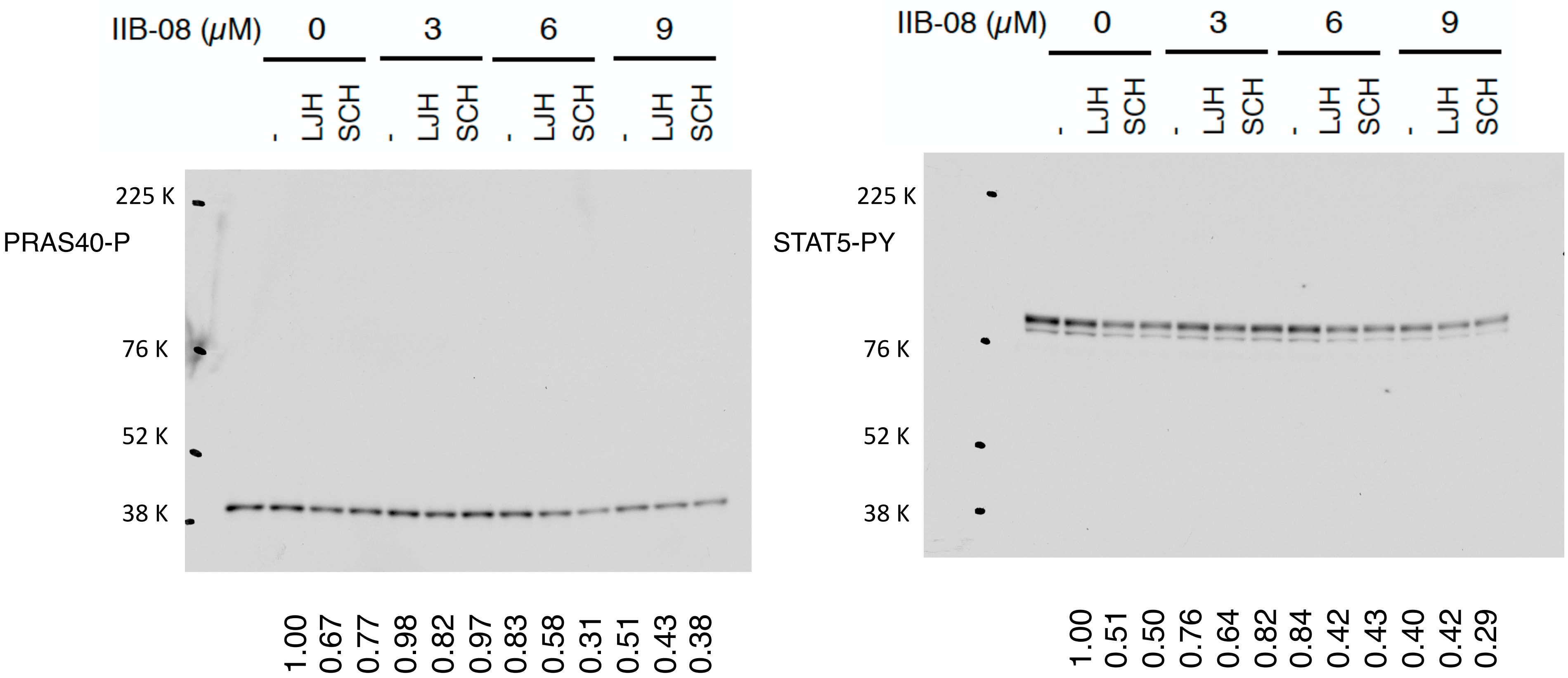

IIB-08 (μM) 0 3 6 9
- LJH SCH - LJH SCH - LJH SCH - LJH SCH
225 K
76 K
52 K
38 K
PRAS40-P
1.00 0.67 0.77 0.98 0.82 0.97 0.83 0.58 0.31 0.51 0.43 0.38
IIB-08 (μM) 0 3 6 9
- LJH SCH - LJH SCH - LJH SCH - LJH SCH
225 K
76 K
52 K
38 K
STAT5-PY
1.00 0.51 0.50 0.76 0.64 0.82 0.84 0.42 0.43 0.40 0.42 0.29

Figure 4F

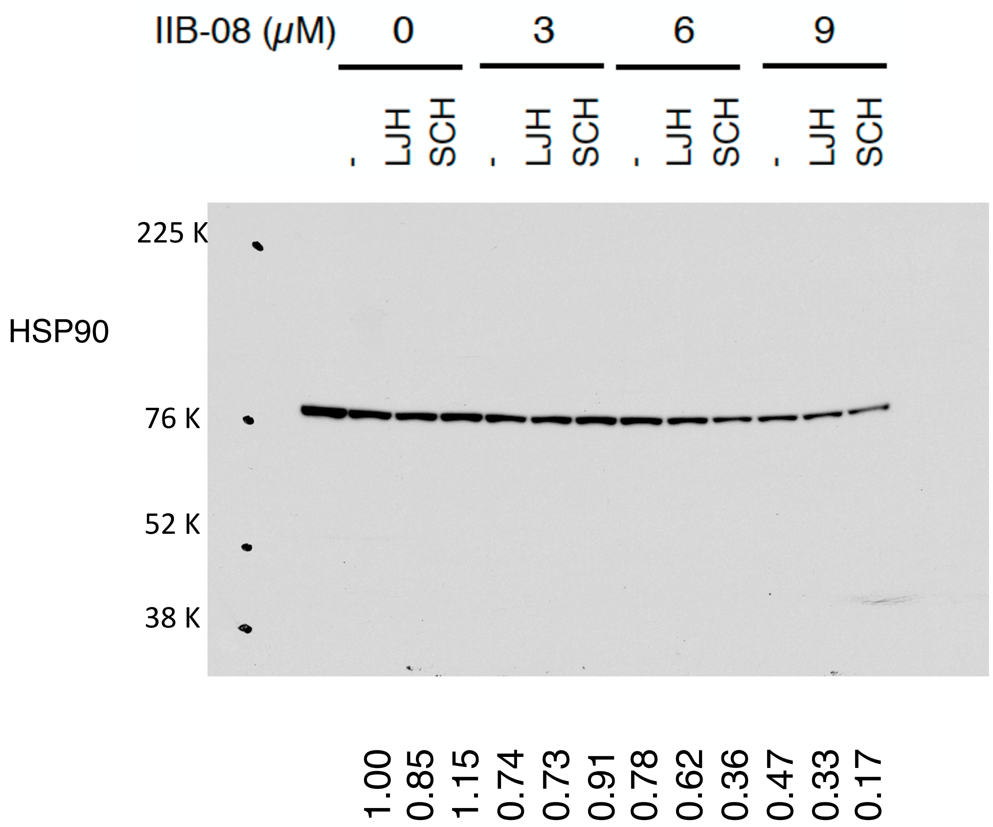
IIB-08 (μM)
0
3
6
9
-
LJH
SCH
-
LJH
SCH
-
LJH
SCH
-
LJH
SCH
225 K
HSP90
76 K
52 K
38 K
1.00
0.85
1.15
0.74
0.73
0.91
0.78
0.62
0.36
0.47
0.33
0.17

Figure 5A

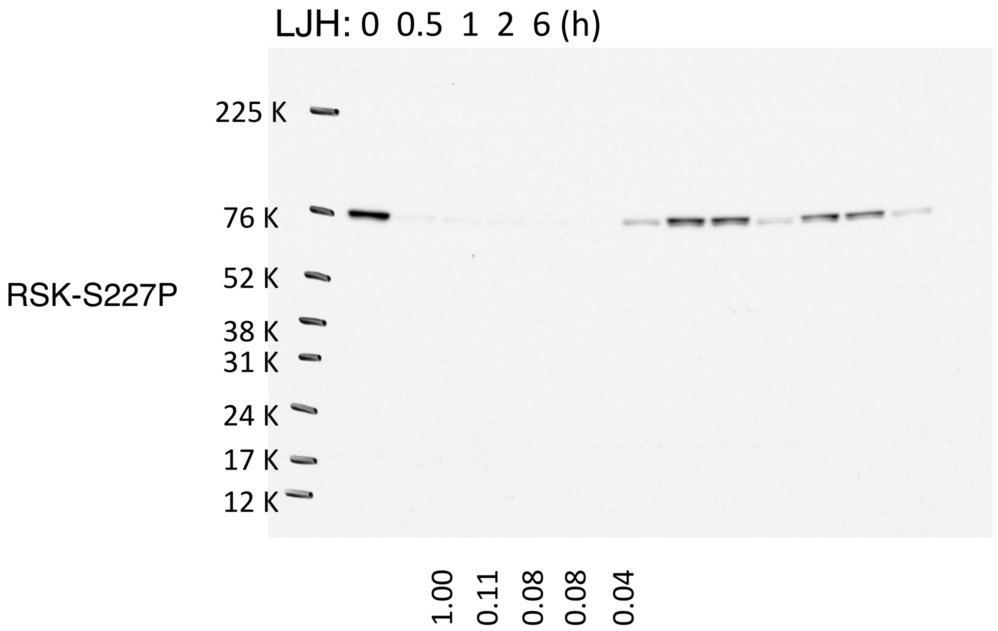
LJH: 0 0.5 1 2 6 (h)
225 K
76 K
52 K
38 K
31 K
24 K
17 K
12 K
RSK-S227P
1.00
0.11
0.08
0.08
0.04

Figure 5A

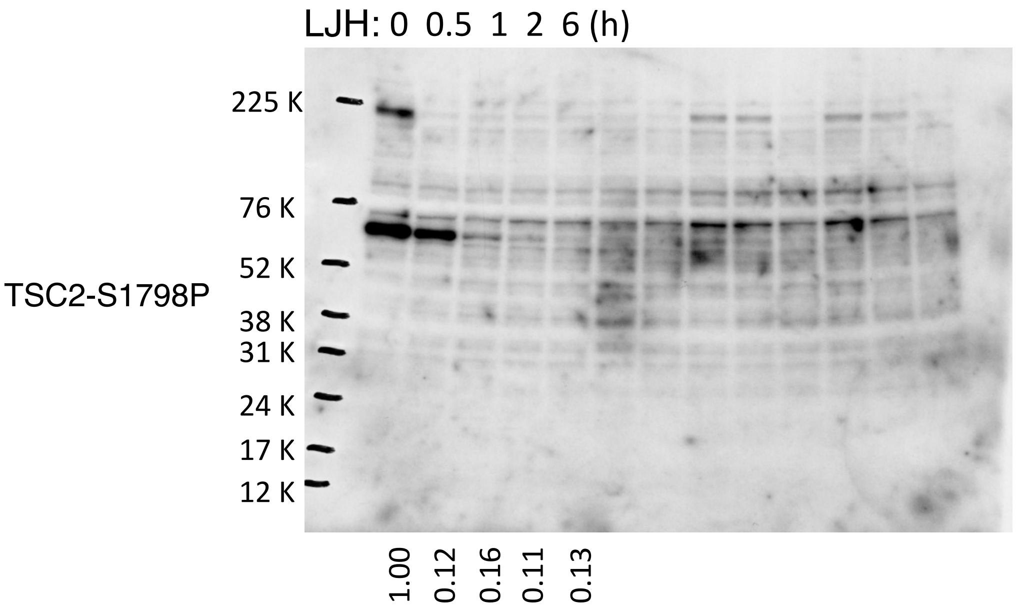
LJH: 0 0.5 1 2 6 (h)
TSC2-S1798P
225 K
76 K
52 K
38 K
31 K
24 K
17 K
12 K
1.00
0.12
0.16
0.11
0.13

Figure 5A

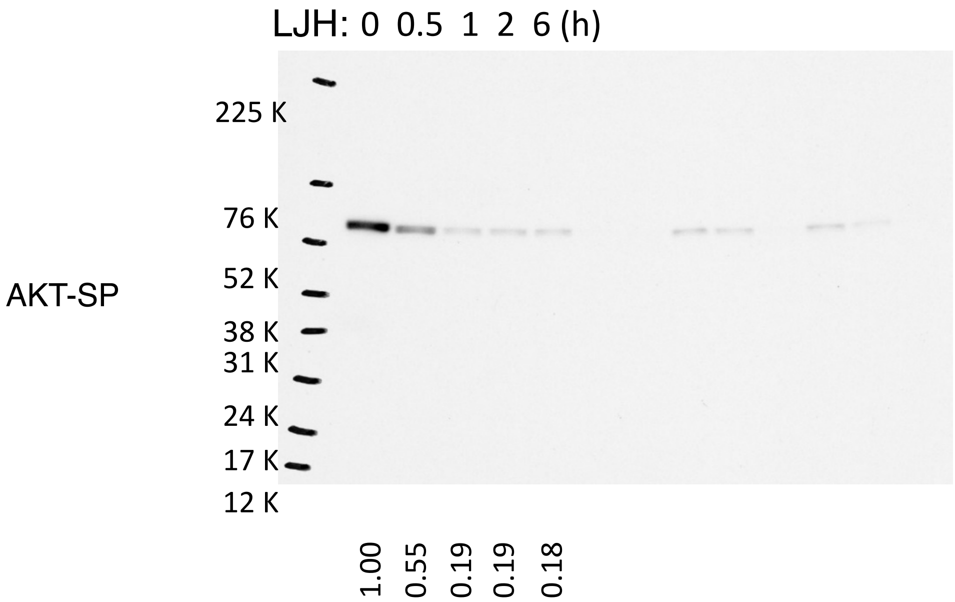

LJH: 0 0.5 1 2 6 (h)
225 K
76 K
52 K
38 K
31 K
24 K
17 K
12 K
AKT-SP
1.00
0.55
0.19
0.19
0.18

Figure 5A

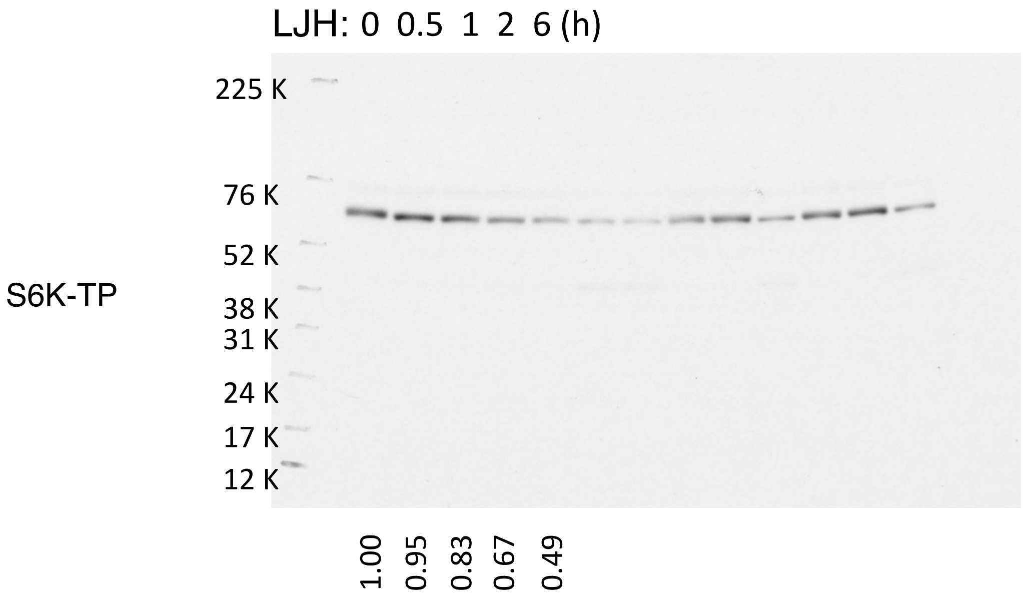
LJH: 0 0.5 1 2 6 (h)
225 K
76 K
52 K
38 K
31 K
24 K
17 K
12 K
S6K-TP
1.00
0.95
0.83
0.67
0.49

Figure 5A

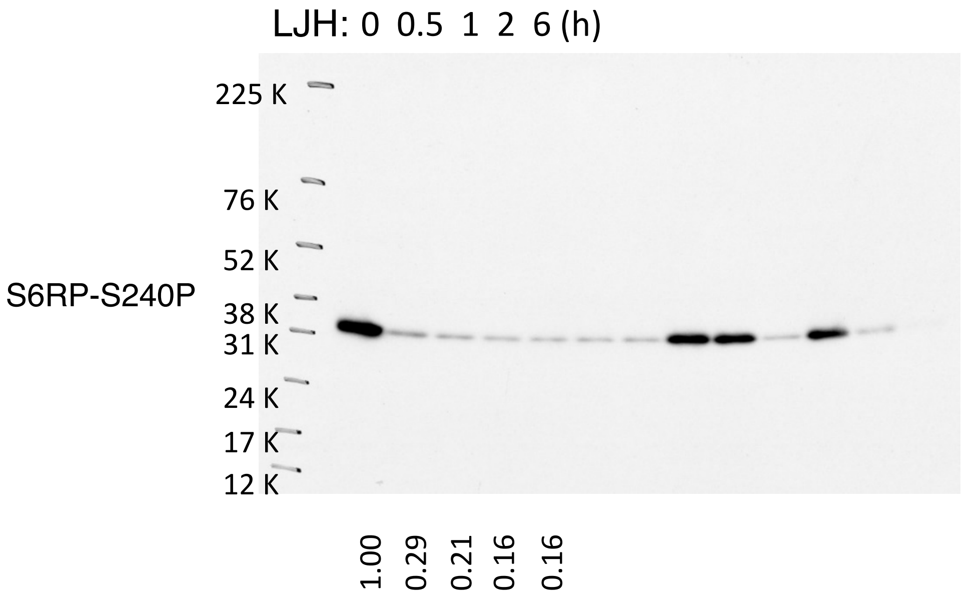
LJH: 0 0.5 1 2 6 (h)
225 K
76 K
52 K
38 K
31 K
24 K
17 K
12 K
S6RP-S240P
1.00
0.29
0.21
0.16
0.16

Figure 5A

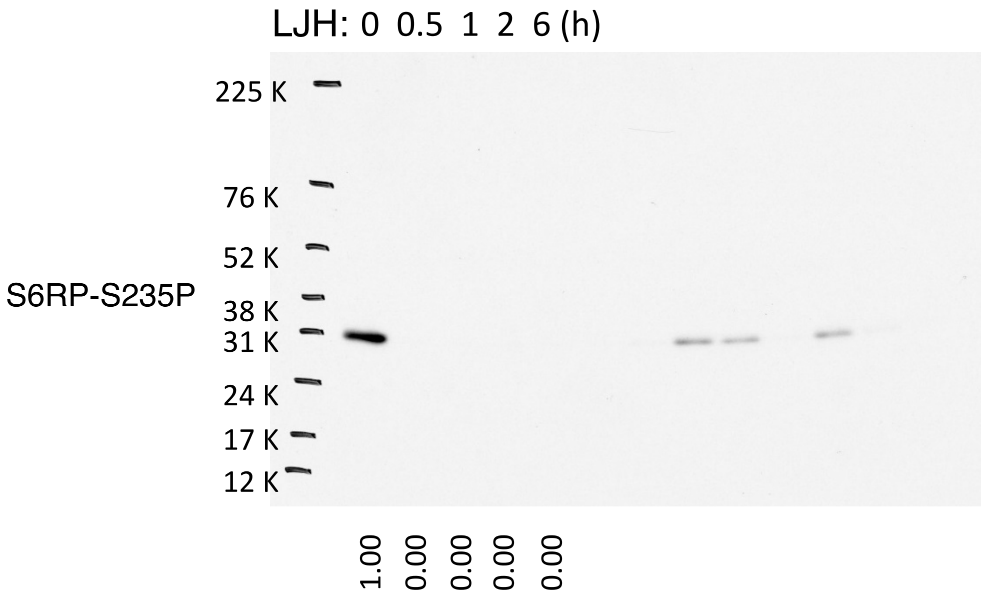

LJH: 0 0.5 1 2 6 (h)
225 K
76 K
52 K
S6RP-S235P
38 K
31 K
24 K
17 K
12 K
1.00
0.00
0.00
0.00
0.00

Figure 5A

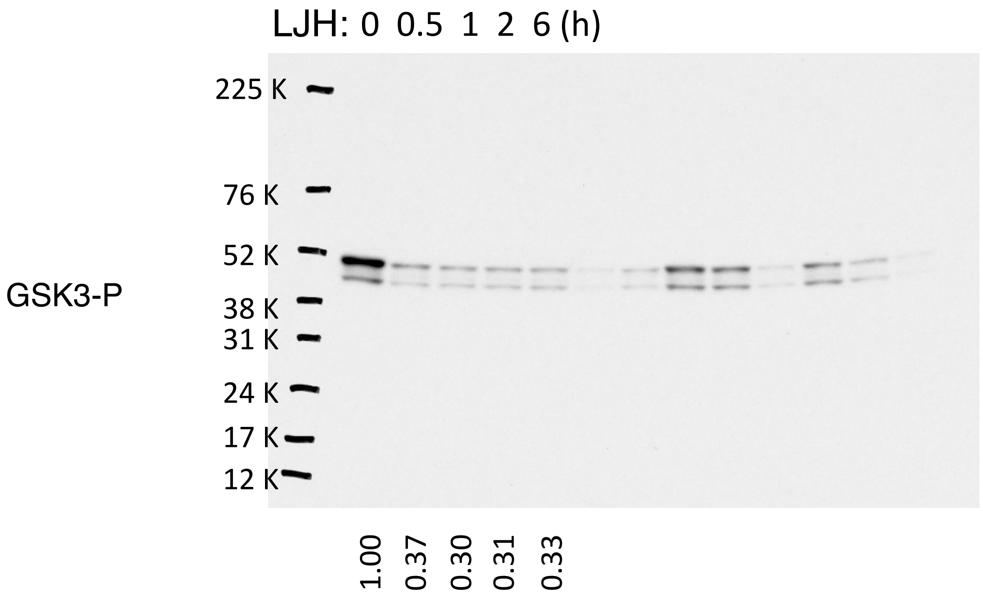
LJH: 0 0.5 1 2 6 (h)
225 K
76 K
52 K
38 K
31 K
24 K
17 K
12 K
GSK3-P
1.00
0.37
0.30
0.31
0.33

Figure 5A

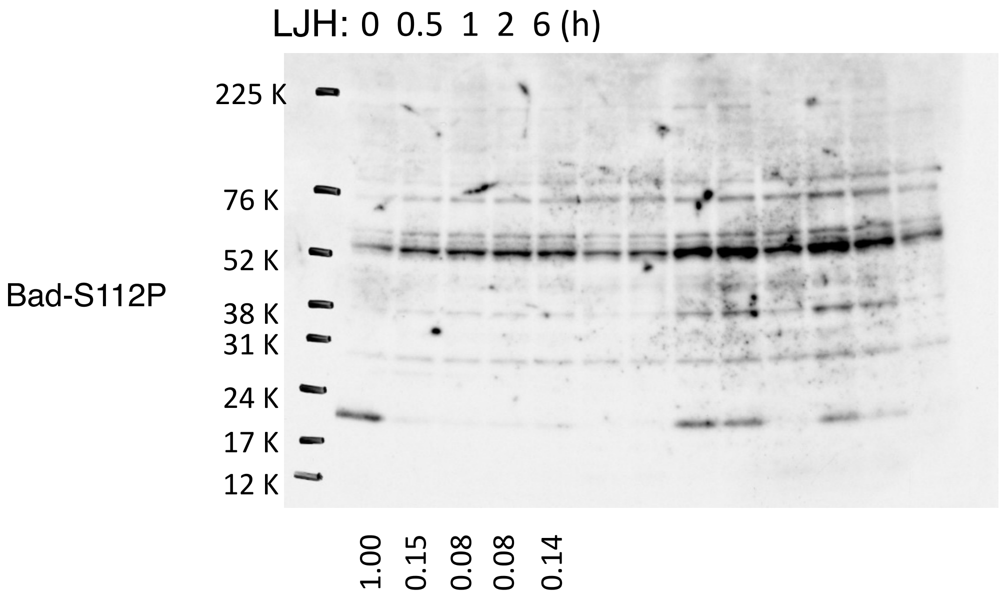

LJH: 0 0.5 1 2 6 (h)
225 K
76 K
52 K
38 K
31 K
24 K
17 K
12 K
Bad-S112P
1.00
0.15
0.08
0.08
0.14

Figure 5A

Bim

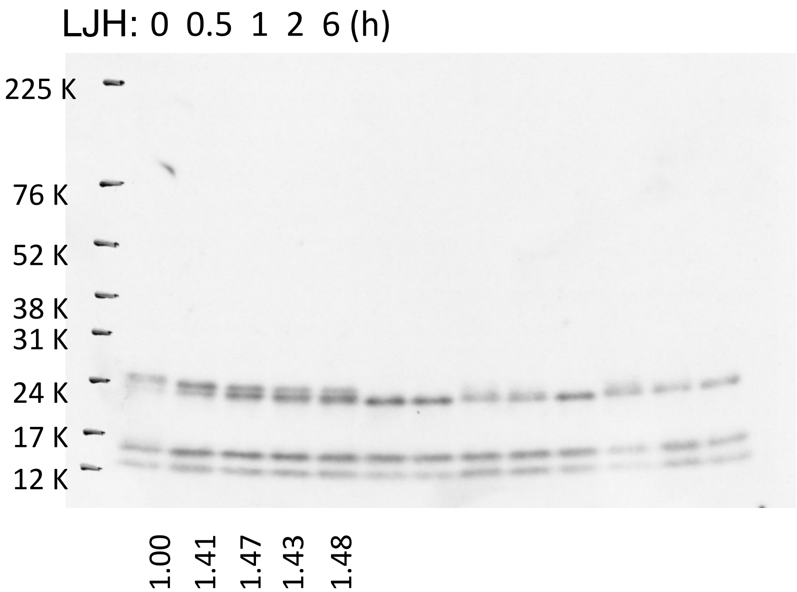

Figure 5A

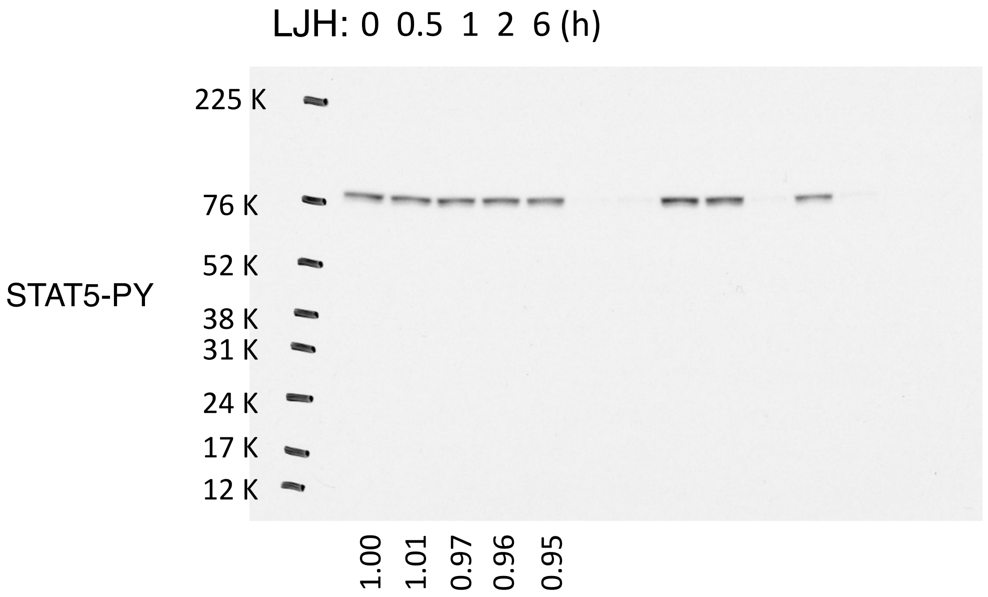

LJH: 0 0.5 1 2 6 (h)
225 K
76 K
52 K
STAT5-PY
38 K
31 K
24 K
17 K
12 K
1.00
1.01
0.97
0.96
0.95

Figure 5A

HSP90

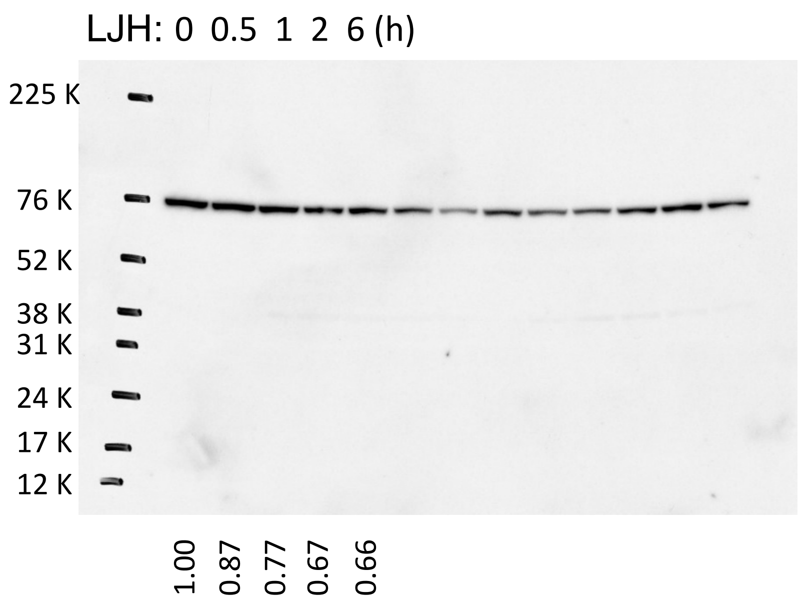

Figure 5B

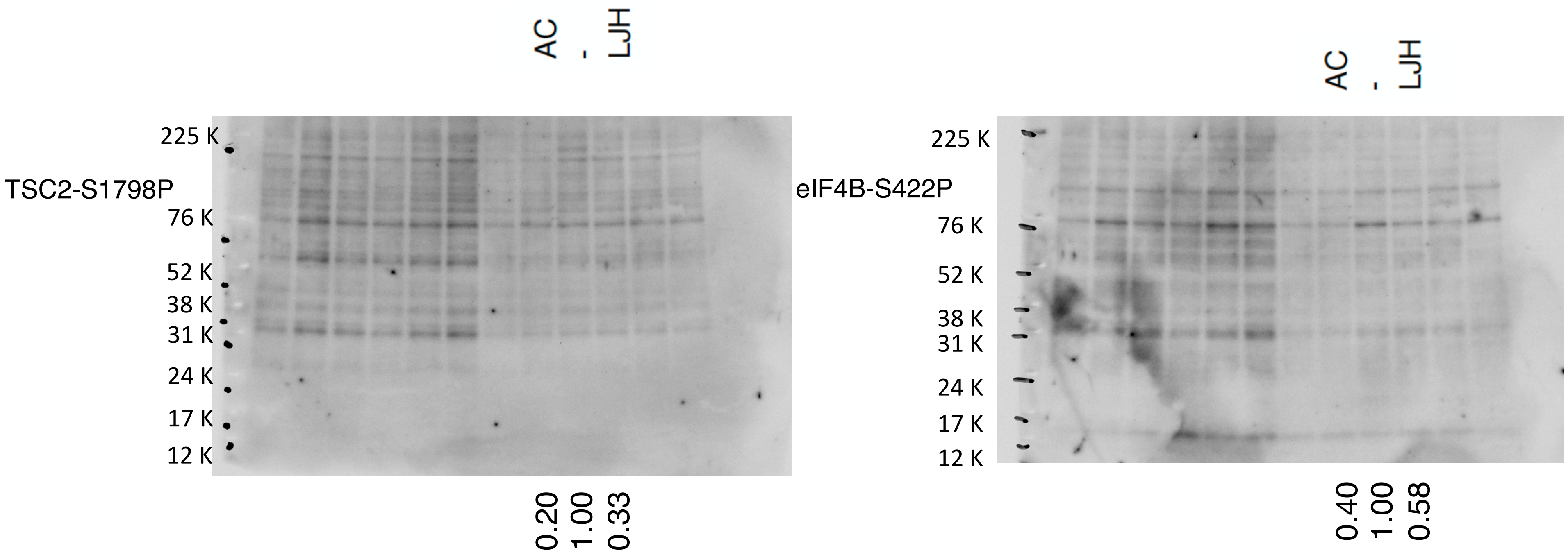
AC
-
LJH
TSC2-S1798P
225 K
76 K
52 K
38 K
31 K
24 K
17 K
12 K
0.20
1.00
0.33
AC
-
LJH
eIF4B-S422P
225 K
76 K
52 K
38 K
31 K
24 K
17 K
12 K
0.40
1.00
0.58

Figure 5B

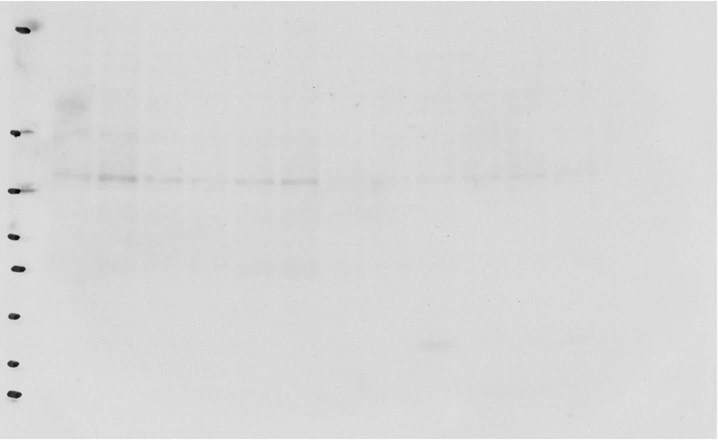

AC
-
LJH
225 K
Bad-S112P
76 K
52 K
38 K
31 K
24 K
17 K
12 K
0.17
1.00
0.24

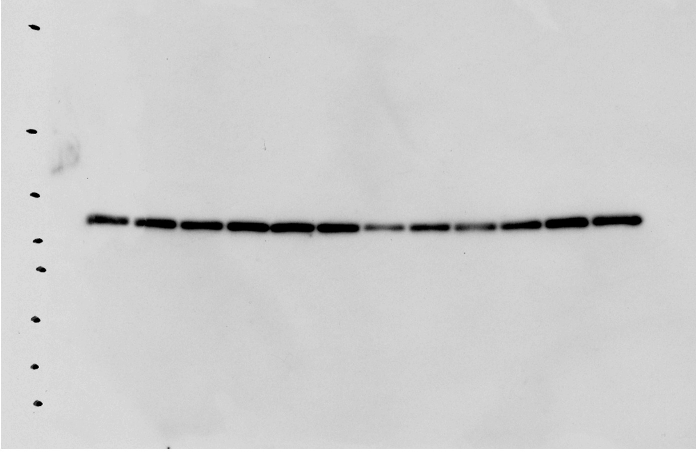

AC
-
LJH
225 K
ß-actin
76 K
52 K
38 K
31 K
24 K
17 K
12 K
1.74
1.00
2.11

Figure 5C

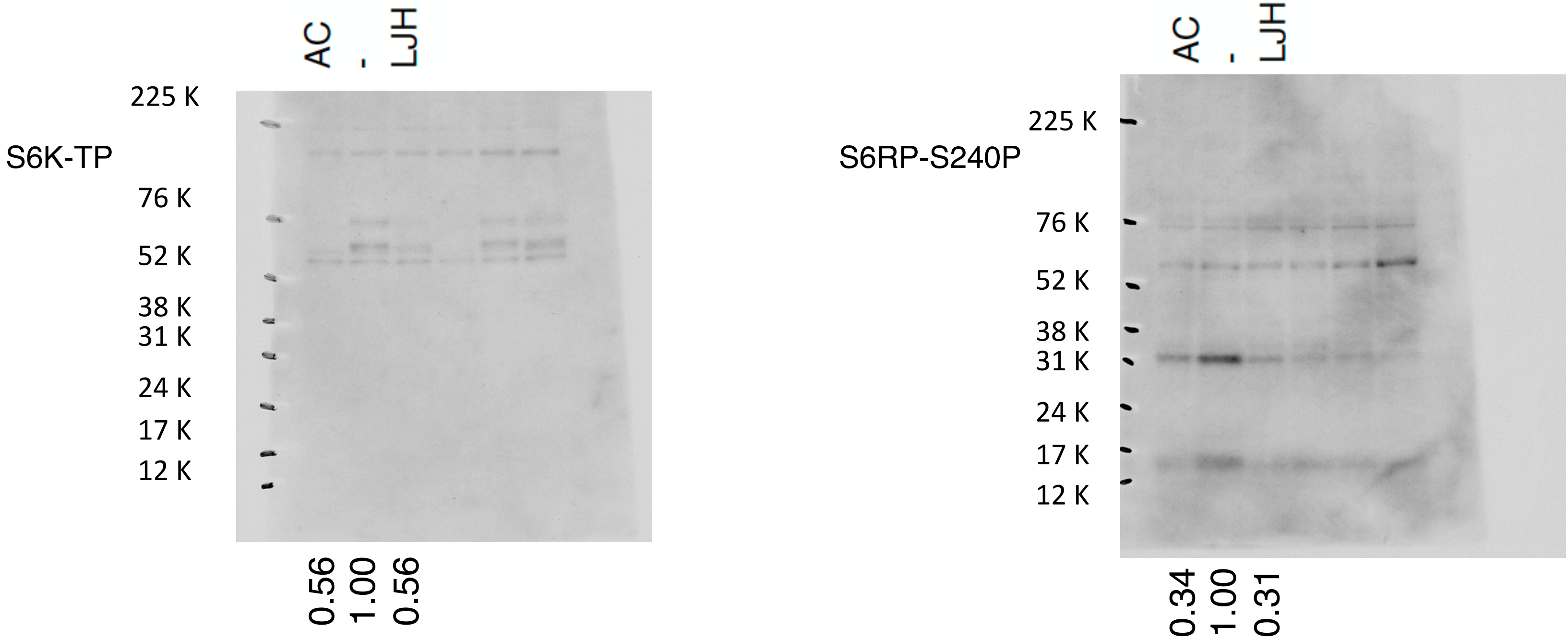

AC
-
LJH
225 K
S6K-TP
76 K
52 K
38 K
31 K
24 K
17 K
12 K
0.56
1.00
0.56
AC
-
LJH
225 K
S6RP-S240P
76 K
52 K
38 K
31 K
24 K
17 K
12 K
0.34
1.00
0.31

Figure 5C

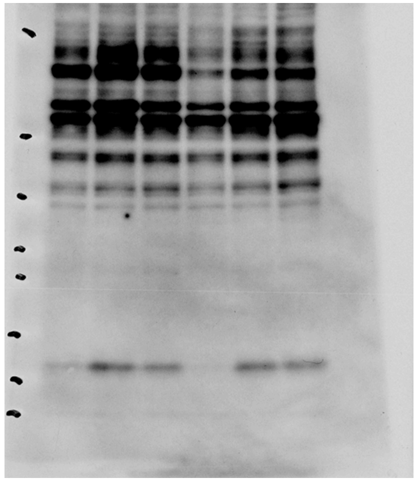

AC
-
LJH
4EBP1-S65P
225 K
76 K
52 K
38 K
31 K
24 K
17 K
12 K
0.25
1.00
0.57

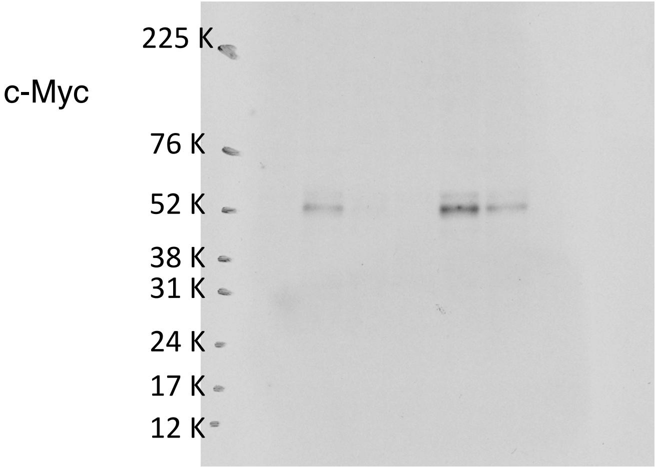

AC
-
LJH
c-Myc
225 K
76 K
52 K
38 K
31 K
24 K
17 K
12 K
0.04
1.00
0.18

Figure 5C

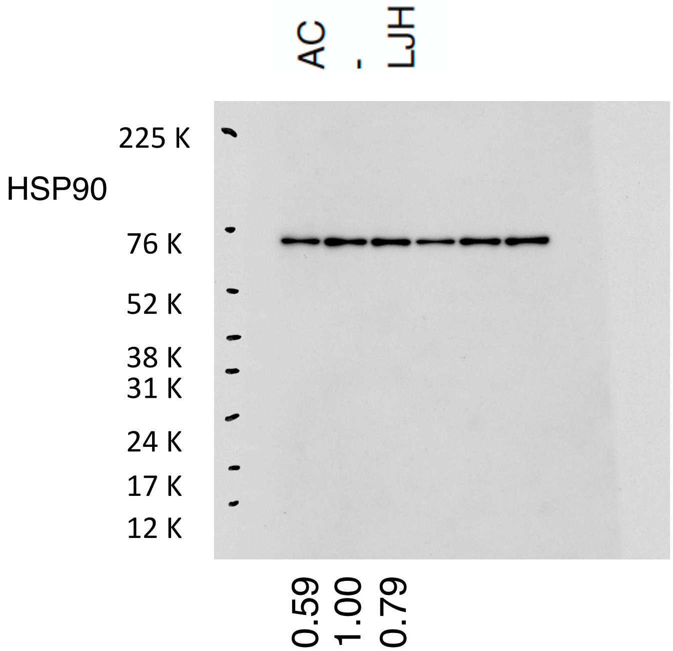

Figure 5D

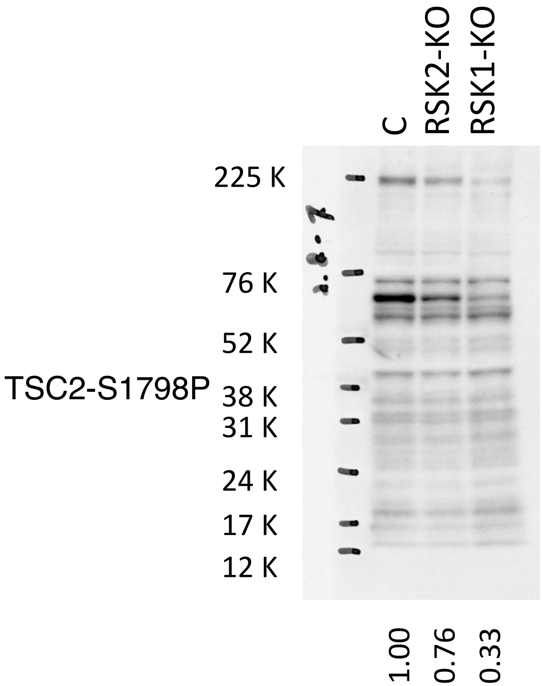

C
RSK2-KO
RSK1-KO
225 K
76 K
52 K
TSC2-S1798P
38 K
31 K
24 K
17 K
12 K
1.00
0.76
0.33

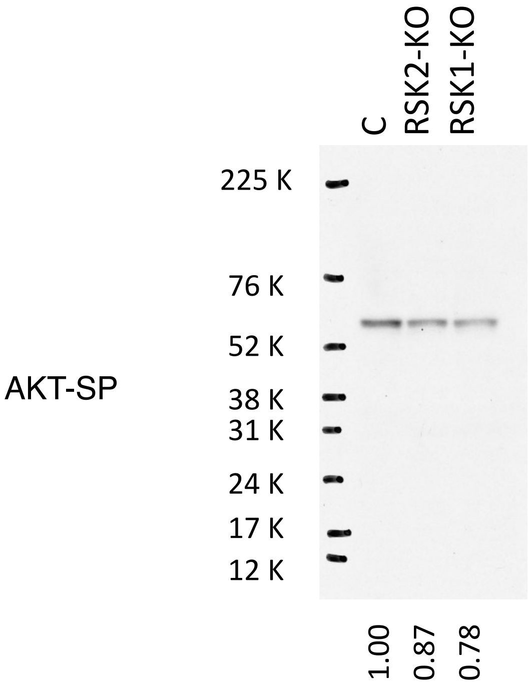

C
RSK2-KO
RSK1-KO
225 K
76 K
52 K
AKT-SP
38 K
31 K
24 K
17 K
12 K
1.00
0.87
0.78

Figure 5D

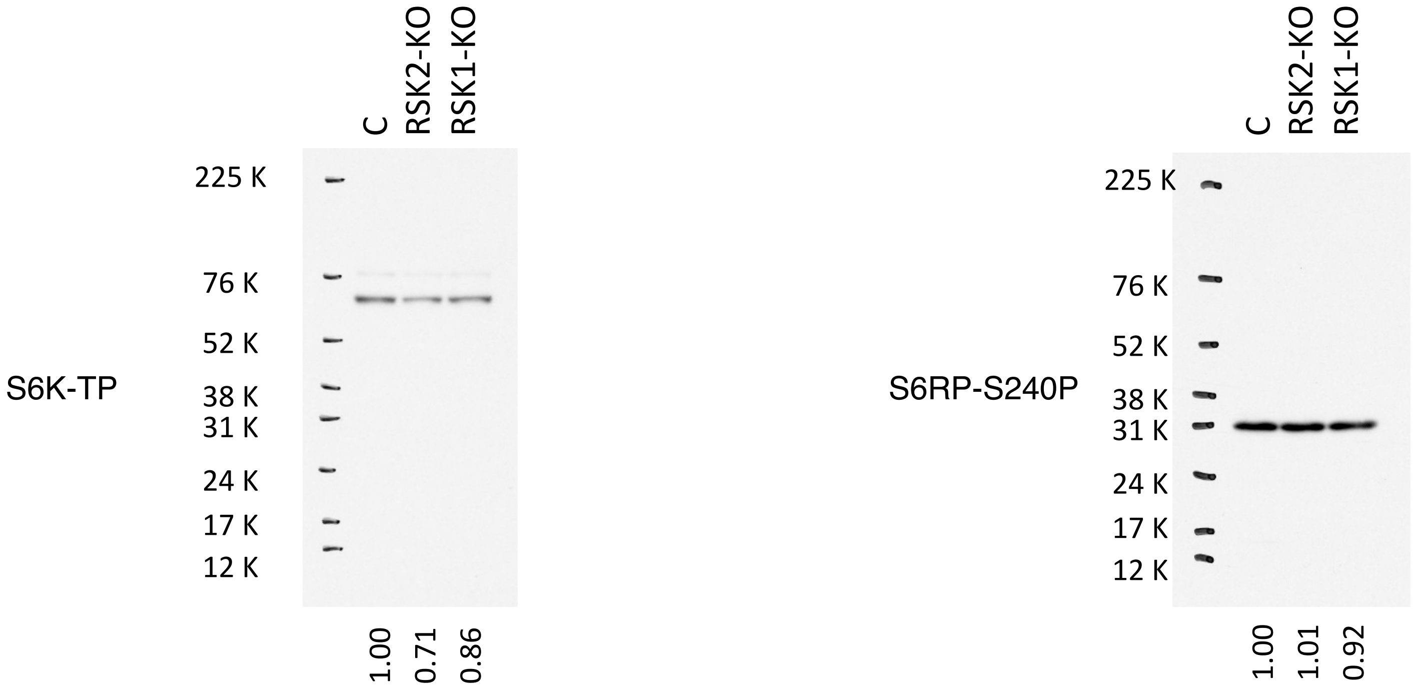
C
RSK2-KO
RSK1-KO
225 K
76 K
52 K
38 K
31 K
24 K
17 K
12 K
S6K-TP
1.00
0.71
0.86
C
RSK2-KO
RSK1-KO
225 K
76 K
52 K
38 K
31 K
24 K
17 K
12 K
S6RP-S240P
1.00
1.01
0.92

Figure 5D

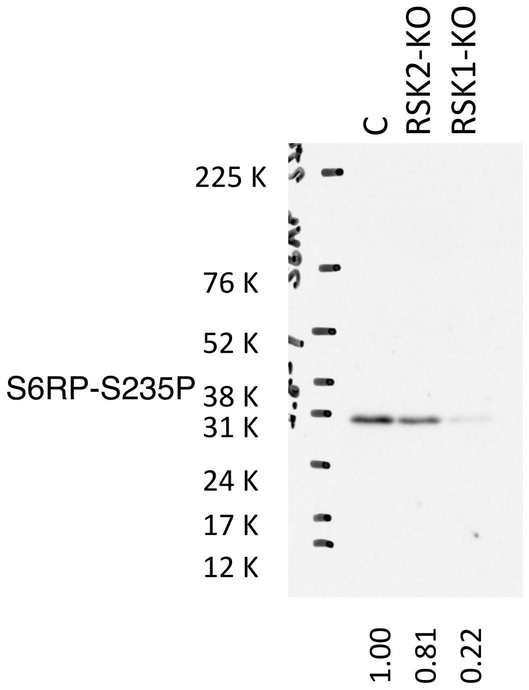
C
RSK2-KO
RSK1-KO
225 K
76 K
52 K
S6RP-S235P
38 K
31 K
24 K
17 K
12 K
1.00
0.81
0.22

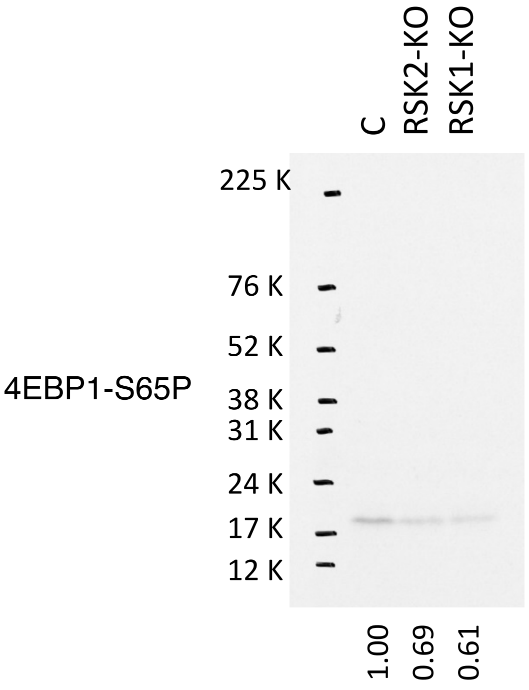
C
RSK2-KO
RSK1-KO
225 K
76 K
52 K
4EBP1-S65P
38 K
31 K
24 K
17 K
12 K
1.00
0.69
0.61

Figure 5D

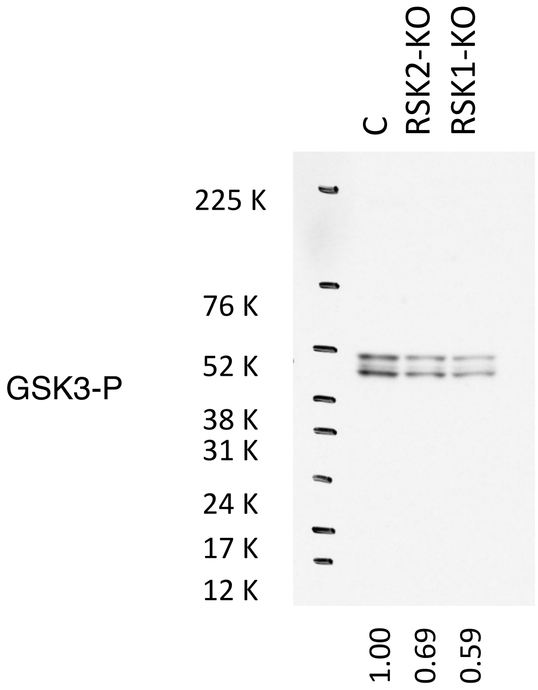
C
RSK2-KO
RSK1-KO
225 K
76 K
52 K
38 K
31 K
24 K
17 K
12 K
GSK3-P
1.00
0.69
0.59

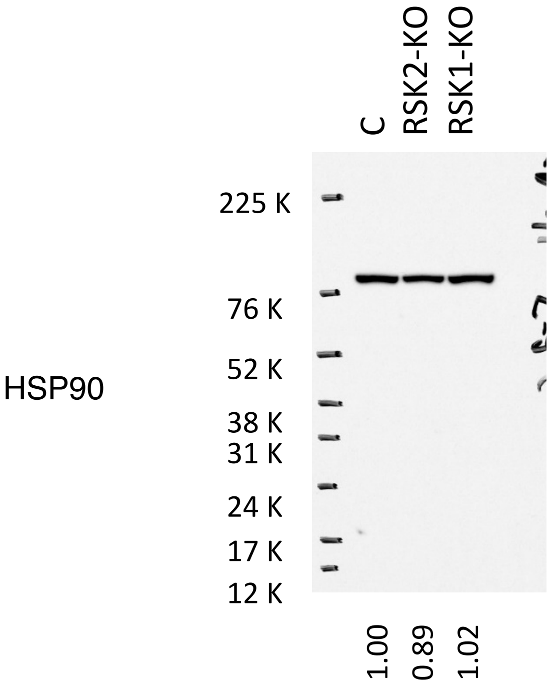
C
RSK2-KO
RSK1-KO
225 K
76 K
52 K
38 K
31 K
24 K
17 K
12 K
HSP90
1.00
0.89
1.02

Figure 5D

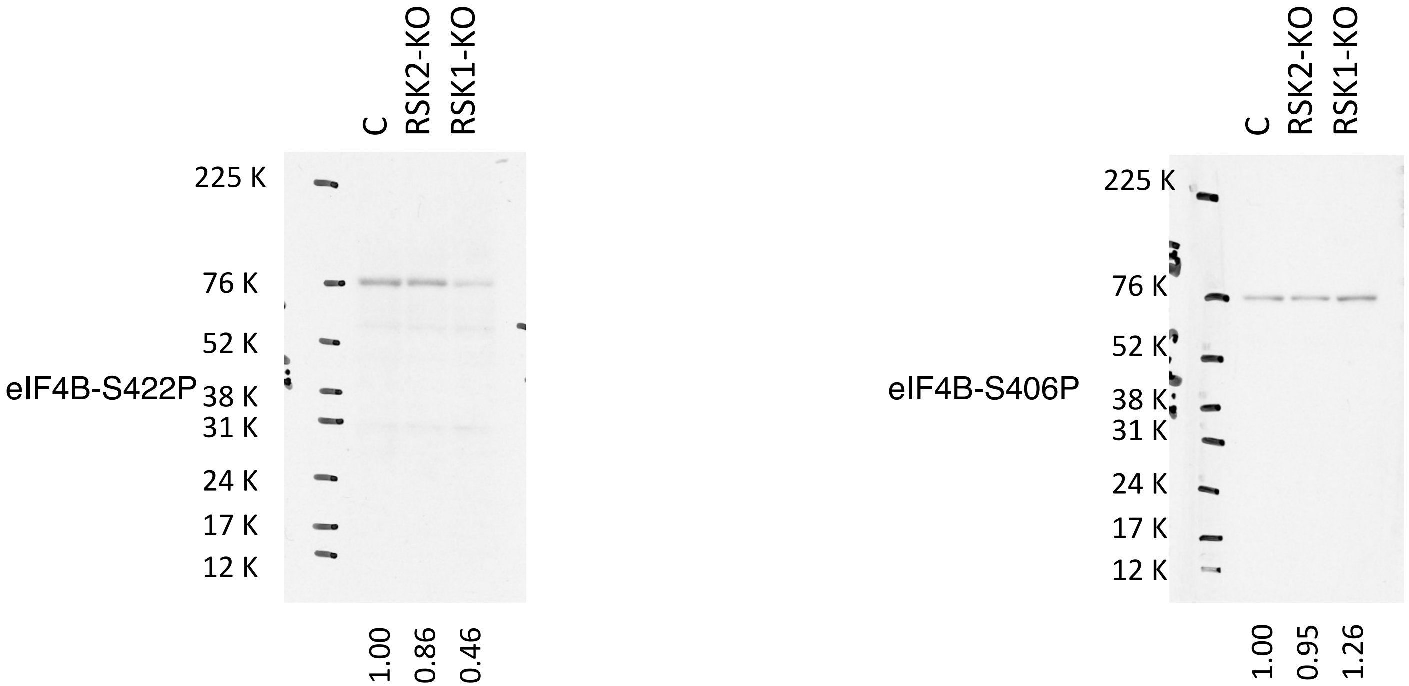
C
RSK2-KO
RSK1-KO
225 K
76 K
52 K
eIF4B-S422P
38 K
31 K
24 K
17 K
12 K
1.00
0.86
0.46
C
RSK2-KO
RSK1-KO
225 K
76 K
52 K
eIF4B-S406P
38 K
31 K
24 K
17 K
12 K
1.00
0.95
1.26

Figure 5D

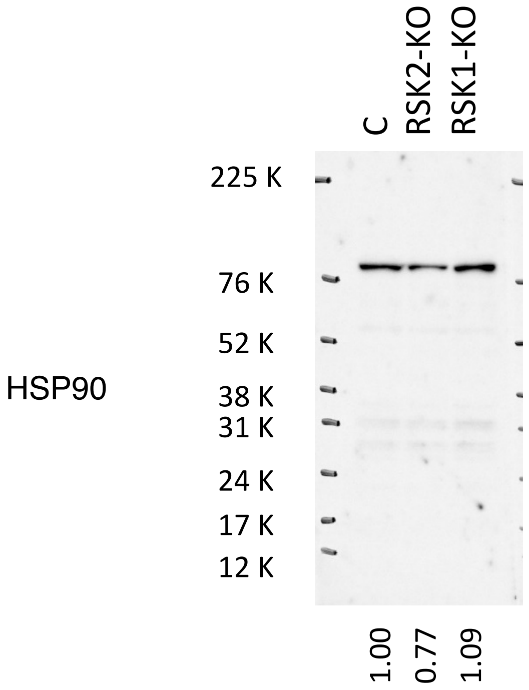

HSP90
C
RSK2-KO
RSK1-KO
225 K
76 K
52 K
38 K
31 K
24 K
17 K
12 K
1.00
0.77
1.09

Figure 5E

RSK1

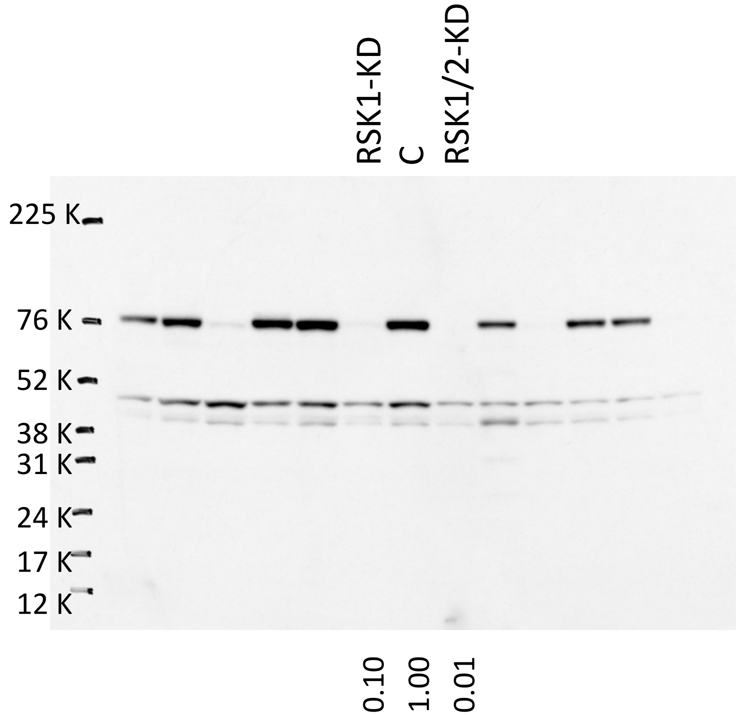

Figure 5E

RSK2

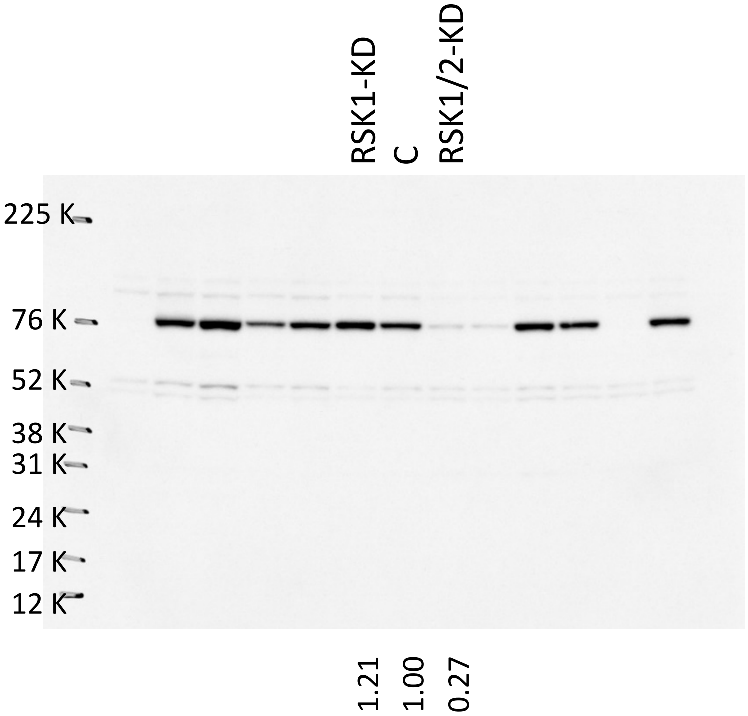

Figure 5E

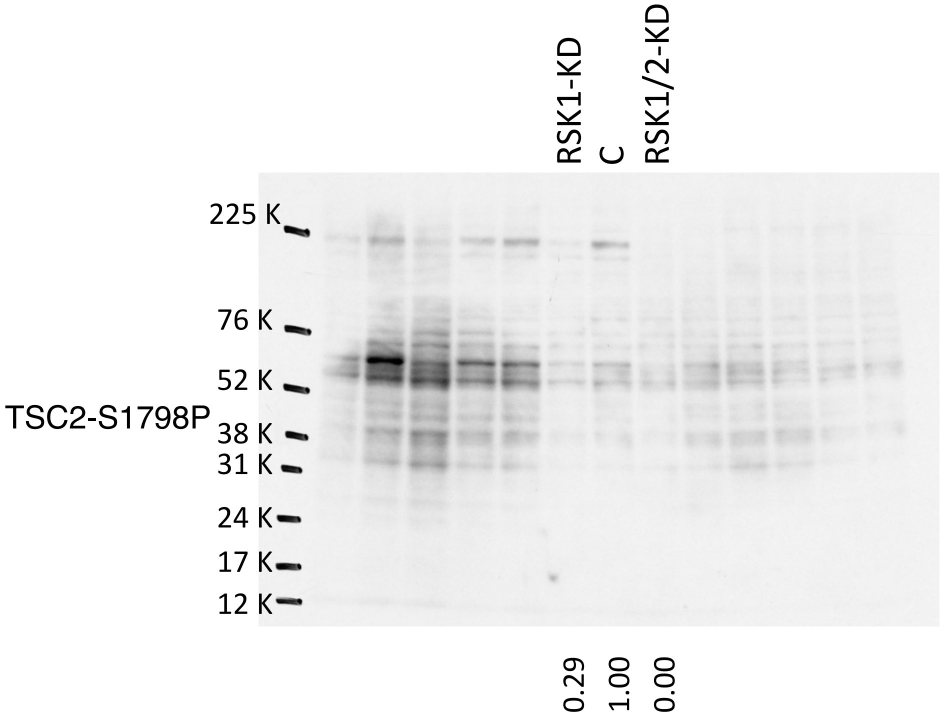

RSK1-KD
C
RSK1/2-KD
225 K
76 K
52 K
TSC2-S1798P
38 K
31 K
24 K
17 K
12 K
0.29
1.00
0.00

Figure 5E

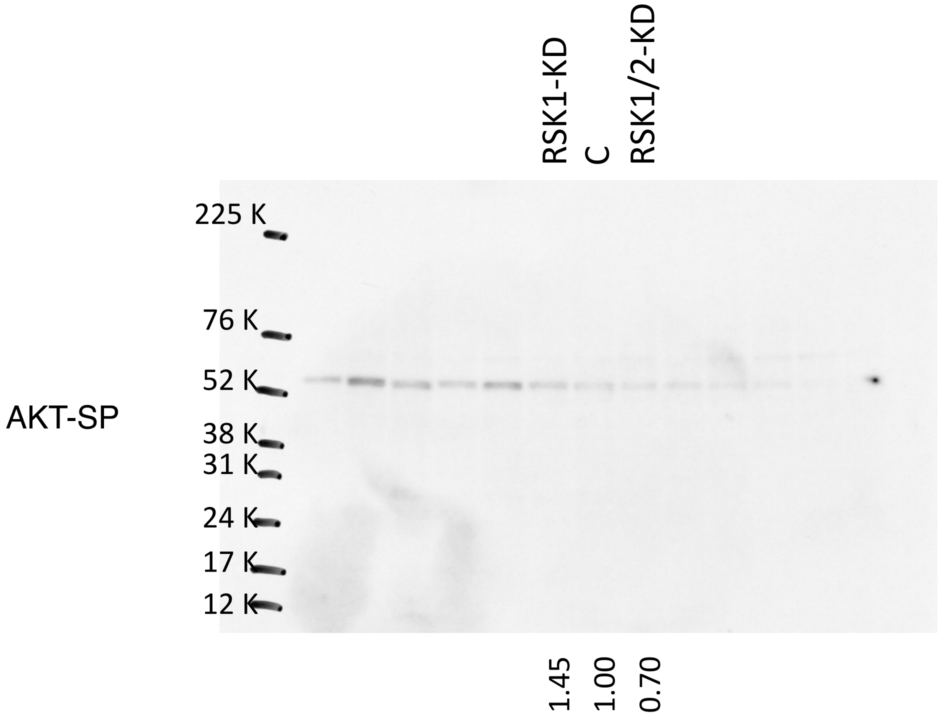
RSK1-KD
C
RSK1/2-KD
225 K
76 K
52 K
38 K
31 K
24 K
17 K
12 K
AKT-SP
1.45
1.00
0.70

Figure 5E

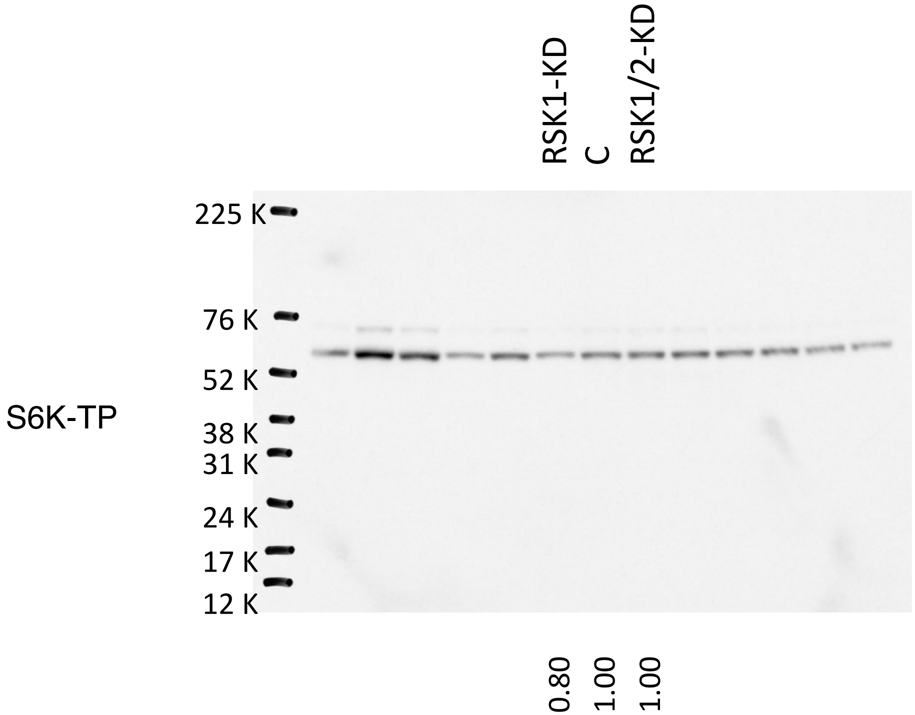
RSK1-KD
C
RSK1/2-KD
225 K
76 K
52 K
S6K-TP
38 K
31 K
24 K
17 K
12 K
0.80
1.00
1.00

Figure 5E

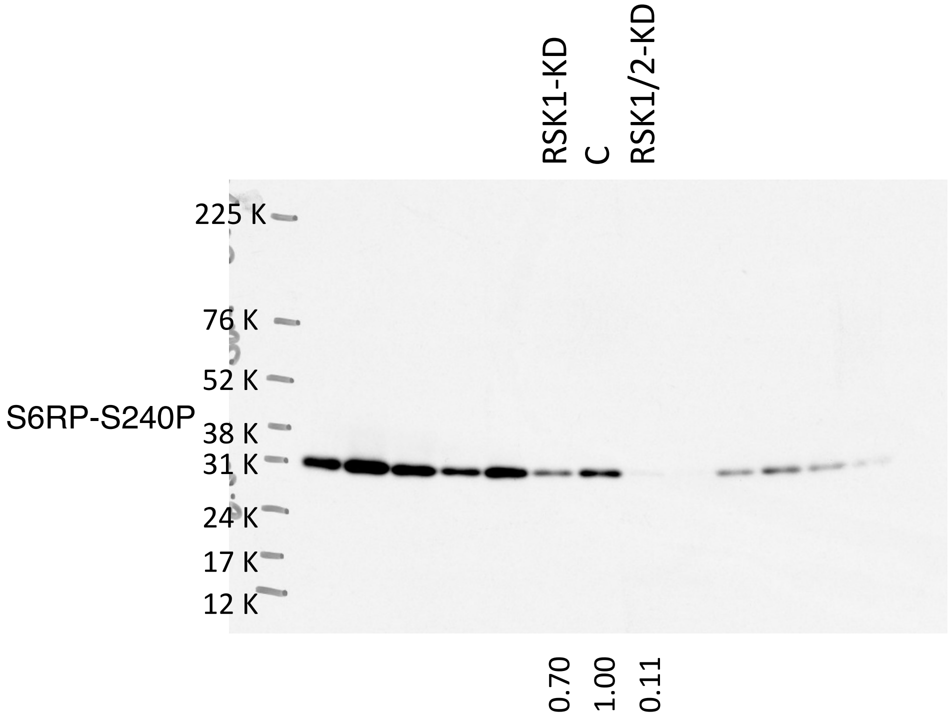
RSK1-KD
C
RSK1/2-KD
225 K
76 K
52 K
S6RP-S240P
38 K
31 K
24 K
17 K
12 K
0.70
1.00
0.11

Figure 5E

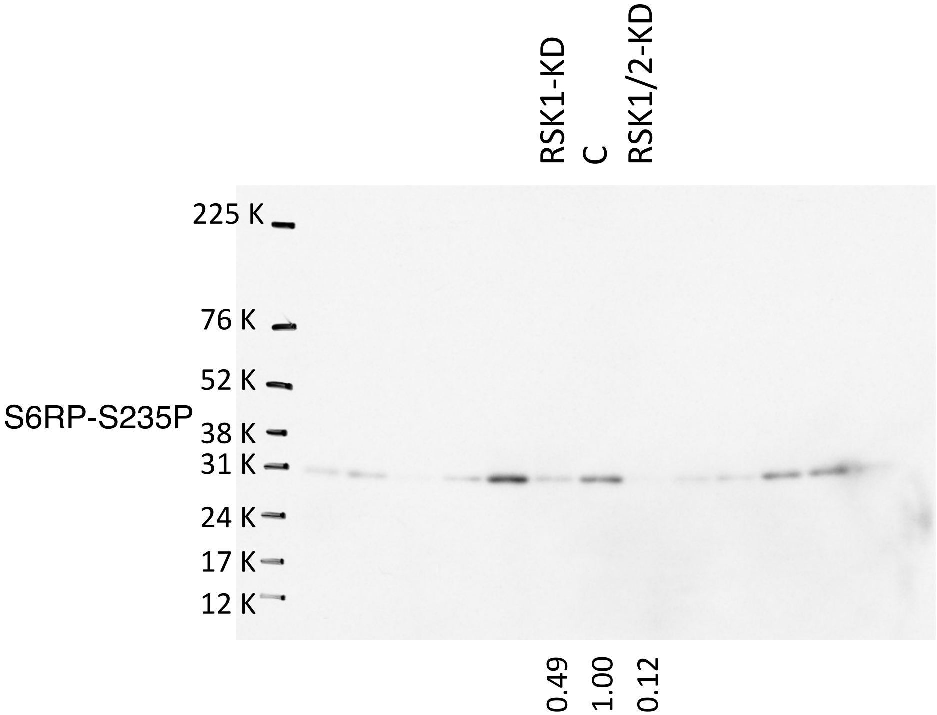
RSK1-KD
C
RSK1/2-KD
225 K
76 K
52 K
S6RP-S235P
38 K
31 K
24 K
17 K
12 K
0.49
1.00
0.12

Figure 5E

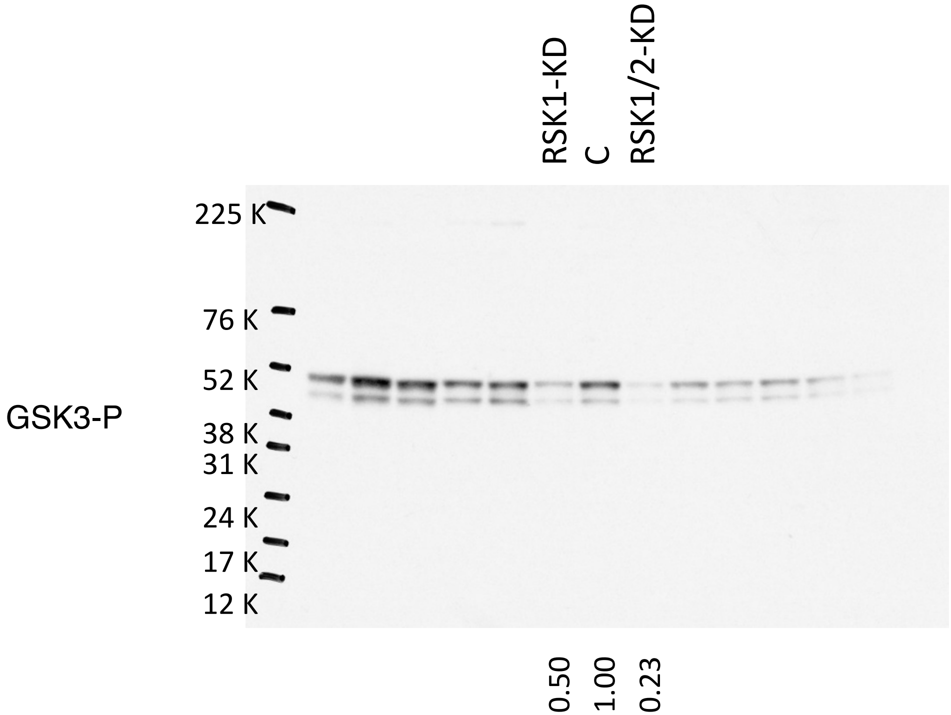
RSK1-KD
C
RSK1/2-KD
225 K
76 K
52 K
GSK3-P
38 K
31 K
24 K
17 K
12 K
0.50
1.00
0.23

Figure 5E

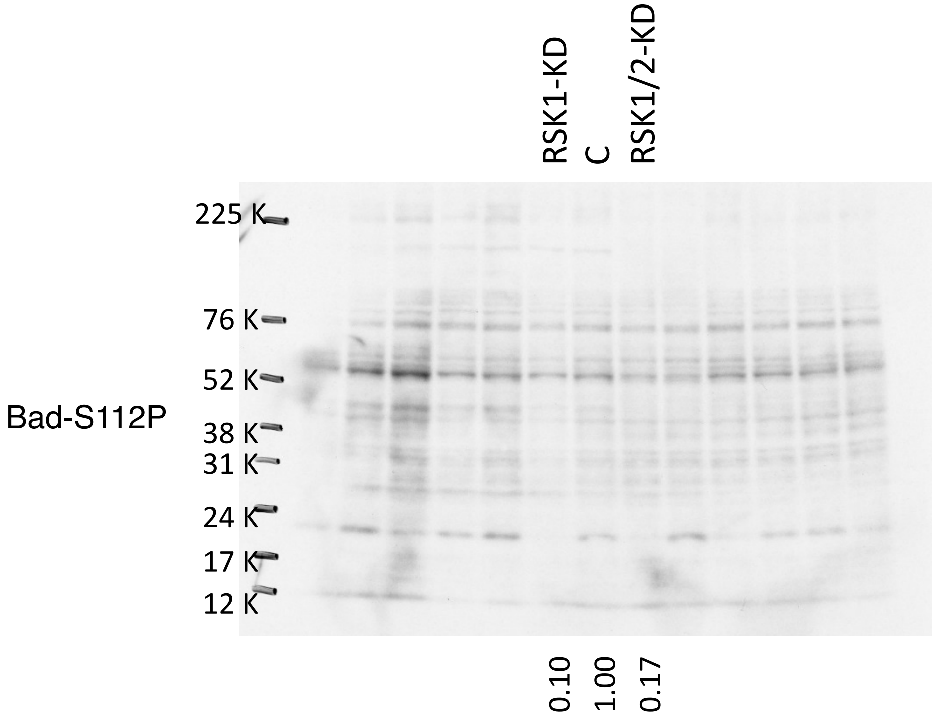
RSK1-KD
C
RSK1/2-KD
225 K
76 K
52 K
38 K
31 K
24 K
17 K
12 K
Bad-S112P
0.10
1.00
0.17

Figure 5E

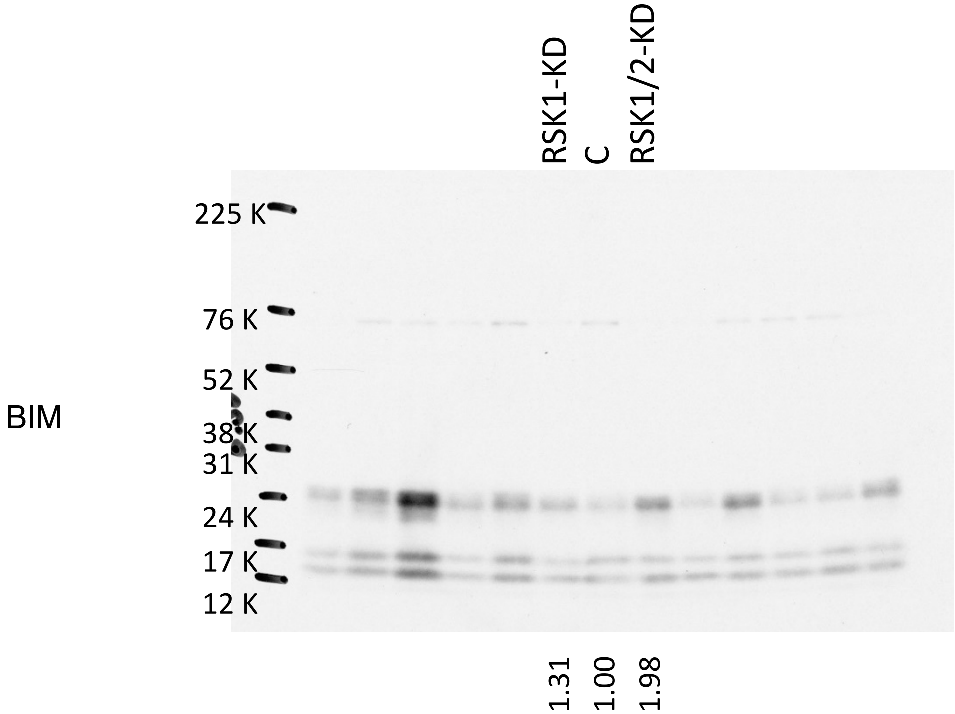
BIM
RSK1-KD
C
RSK1/2-KD
225 K
76 K
52 K
38 K
31 K
24 K
17 K
12 K
1.31
1.00
1.98

Figure 5E

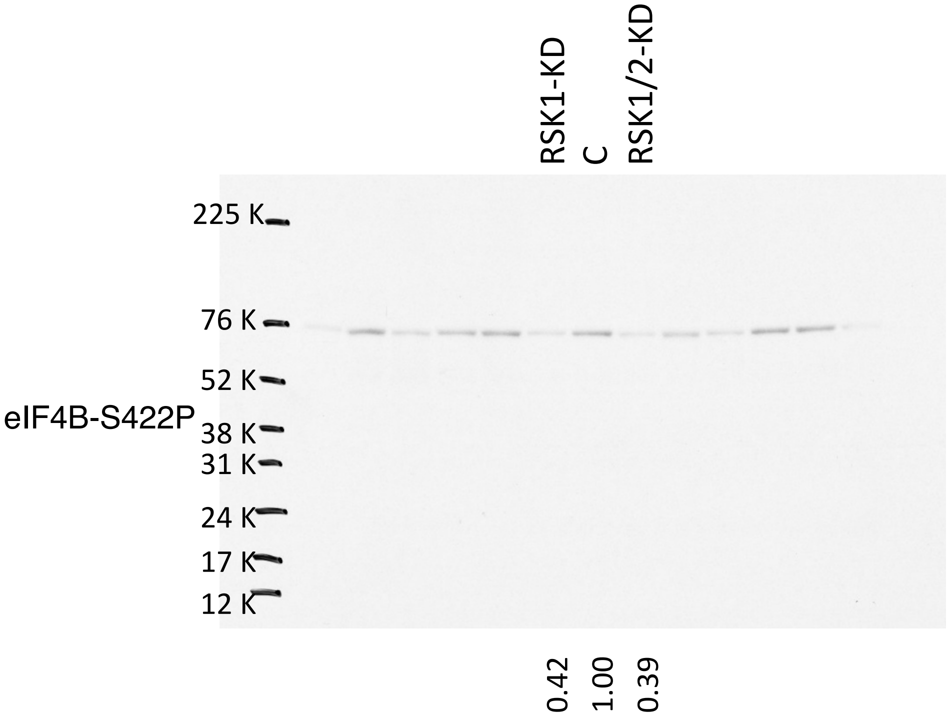
RSK1-KD
C
RSK1/2-KD
225 K
76 K
52 K
38 K
31 K
24 K
17 K
12 K
eIF4B-S422P
0.42
1.00
0.39

Figure 5E

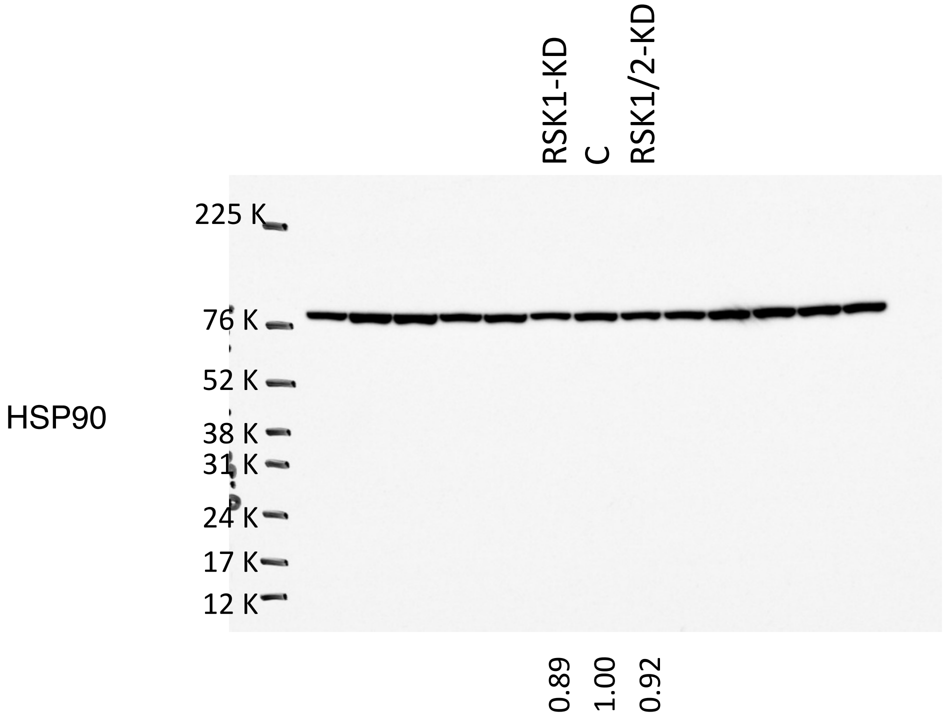
RSK1-KD
C
RSK1/2-KD
225 K
76 K
52 K
HSP90
38 K
31 K
24 K
17 K
12 K
0.89
1.00
0.92

Figure 5F

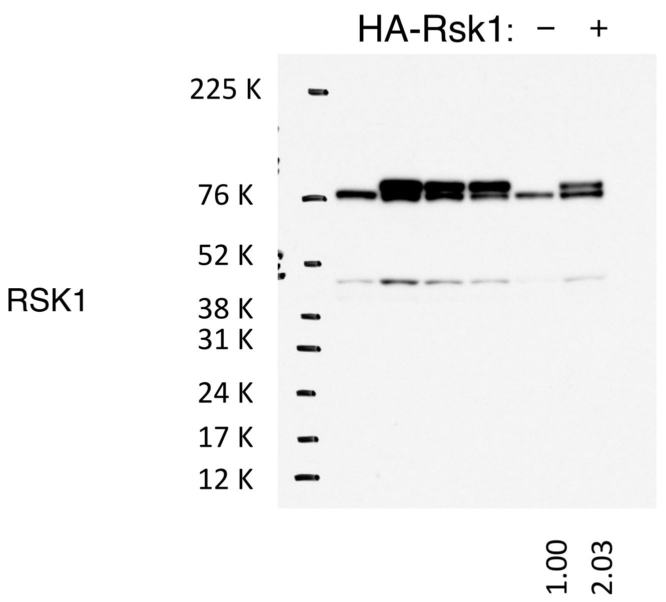
HA-Rsk1: − +
225 K
76 K
52 K
38 K
31 K
24 K
17 K
12 K
RSK1
1.00
2.03

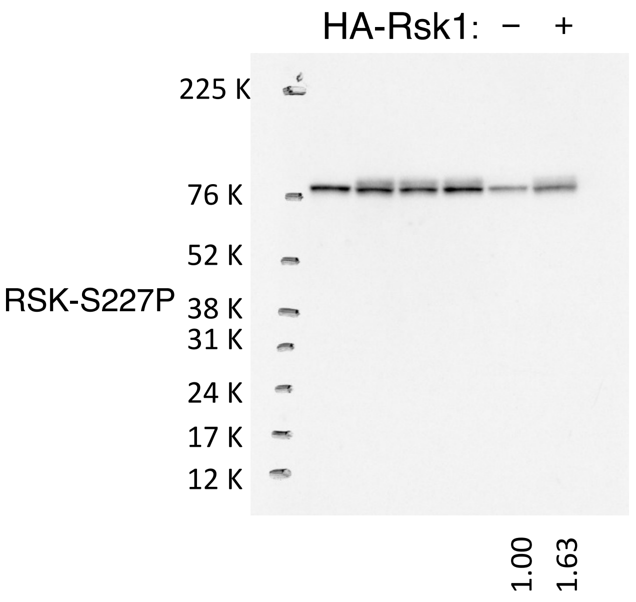
HA-Rsk1: − +
225 K
76 K
52 K
38 K
31 K
24 K
17 K
12 K
RSK-S227P
1.00
1.63

Figure 5F

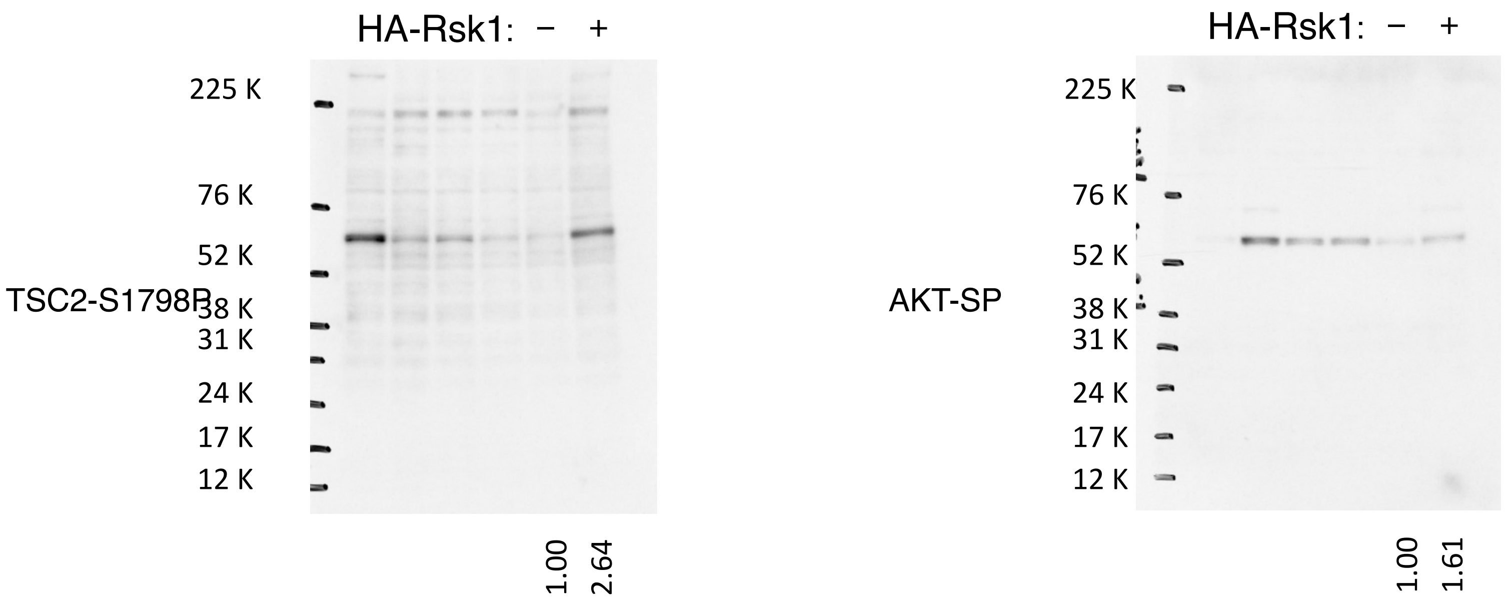
HA-Rsk1: − +
225 K
76 K
52 K
TSC2-S1798P
38 K
31 K
24 K
17 K
12 K
1.00
2.64
HA-Rsk1: − +
225 K
76 K
52 K
AKT-SP
38 K
31 K
24 K
17 K
12 K
1.00
1.61

Figure 5F

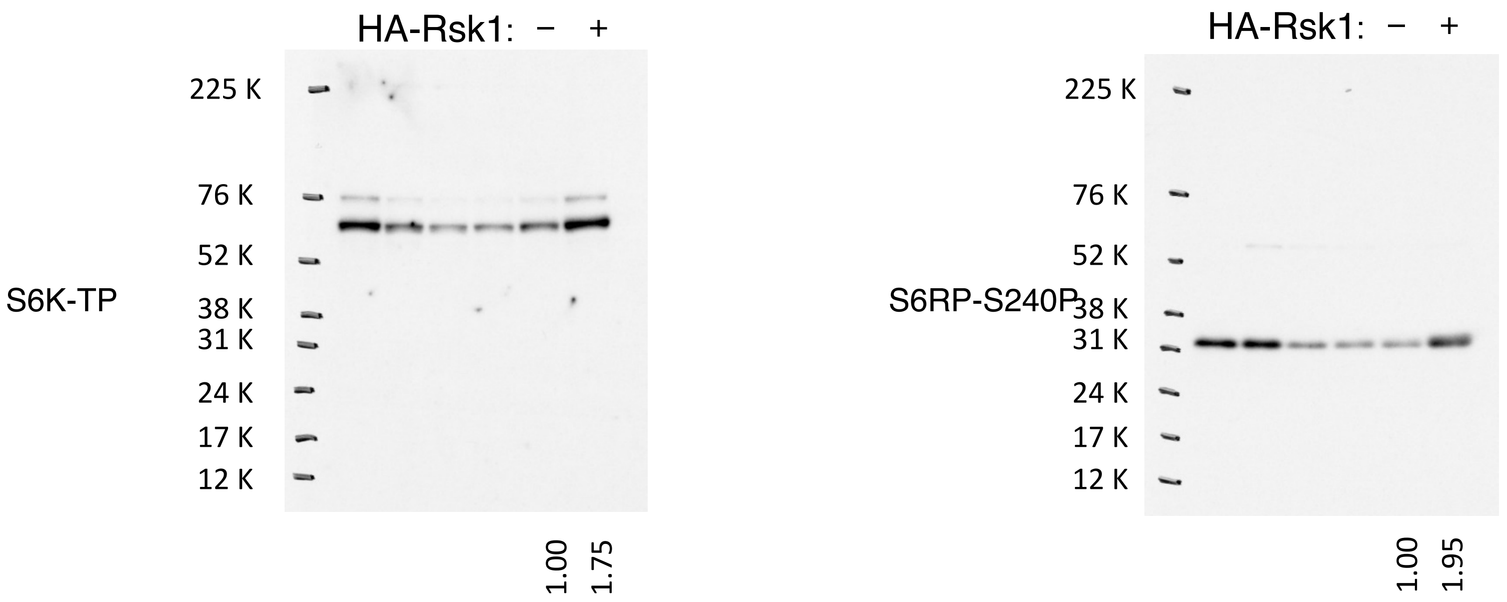
HA-Rsk1: − +
225 K
76 K
52 K
S6K-TP
38 K
31 K
24 K
17 K
12 K
1.00
1.75
HA-Rsk1: − +
225 K
76 K
52 K
S6RP-S240P
38 K
31 K
24 K
17 K
12 K
1.00
1.95

Figure 5F

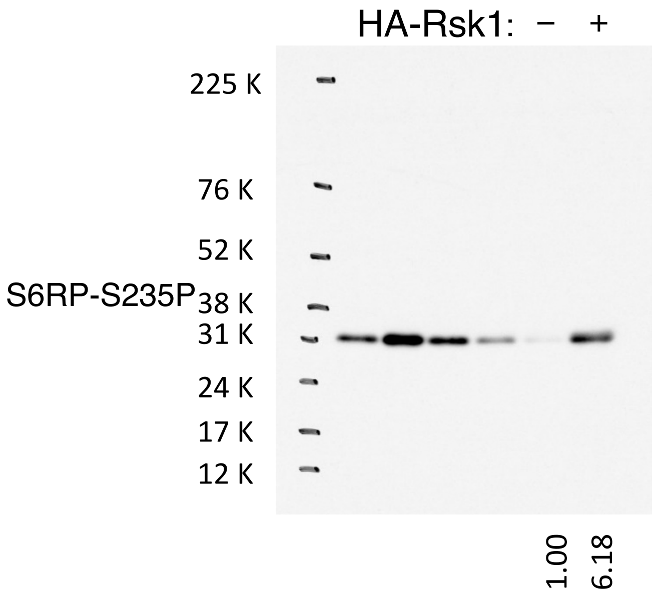
HA-Rsk1: − +
225 K
76 K
52 K
S6RP-S235P
38 K
31 K
24 K
17 K
12 K
1.00
6.18

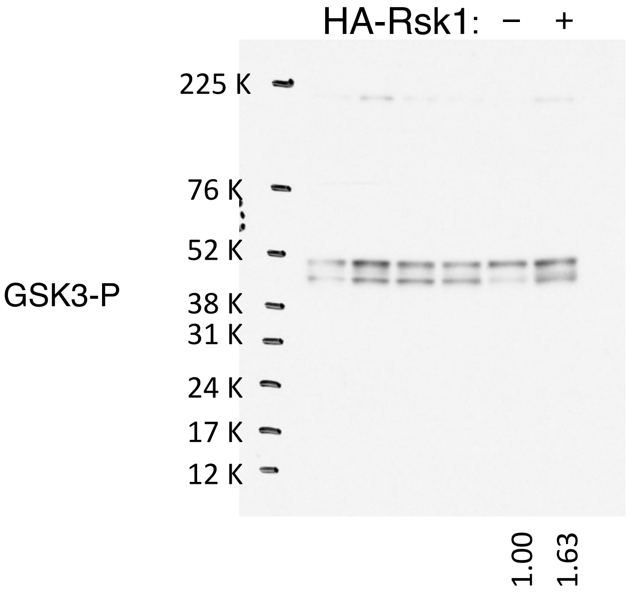
HA-Rsk1: − +
225 K
76 K
52 K
GSK3-P
38 K
31 K
24 K
17 K
12 K
1.00
1.63

Figure 5F

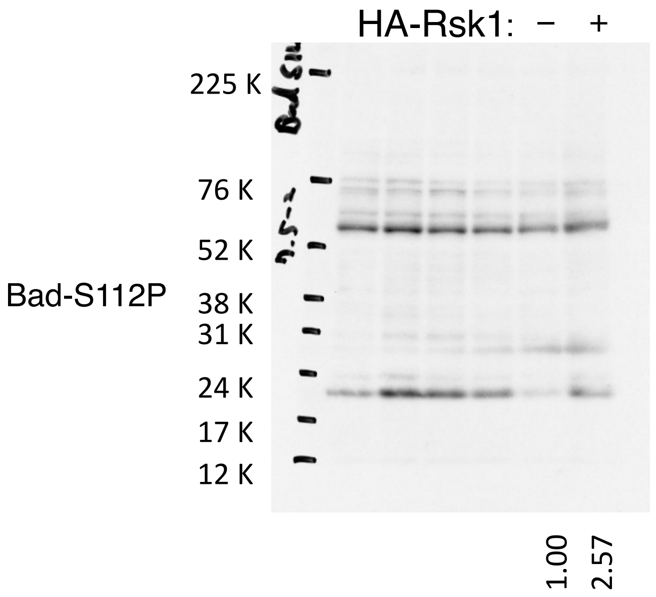
HA-Rsk1: − +
225 K
76 K
52 K
Bad-S112P
38 K
31 K
24 K
17 K
12 K
1.00
2.57

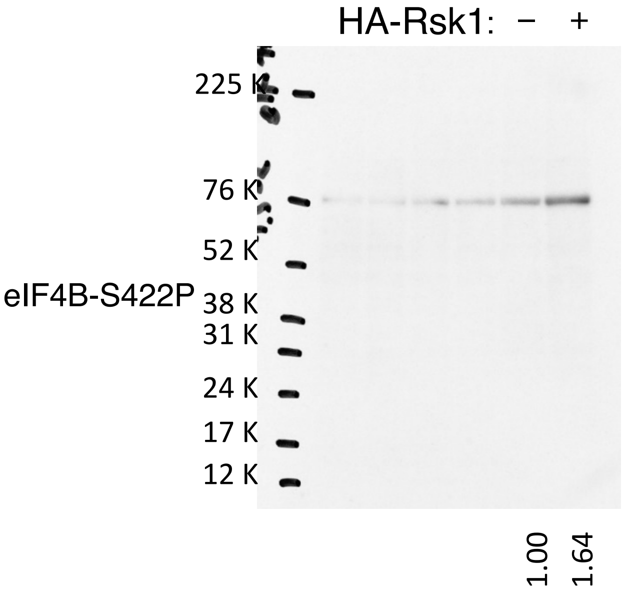
HA-Rsk1: − +
225 K
76 K
52 K
eIF4B-S422P
38 K
31 K
24 K
17 K
12 K
1.00
1.64

Figure 5F

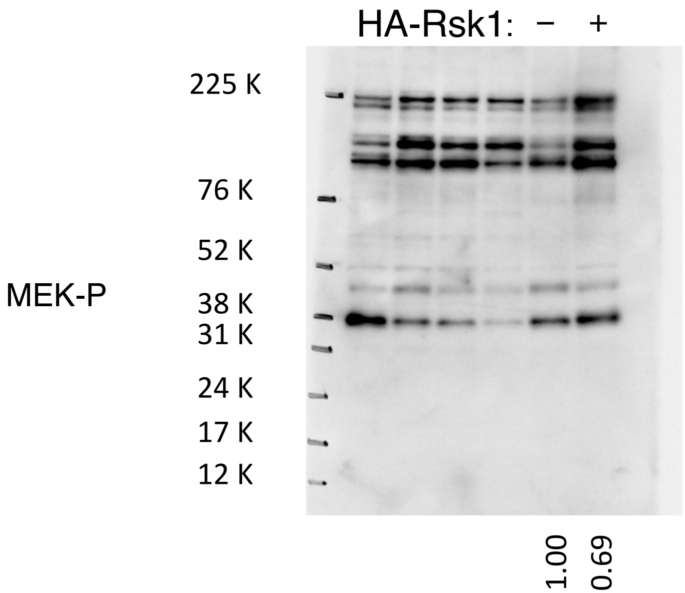
HA-Rsk1: − +
225 K
76 K
52 K
38 K
31 K
24 K
17 K
12 K
MEK-P
1.00
0.69

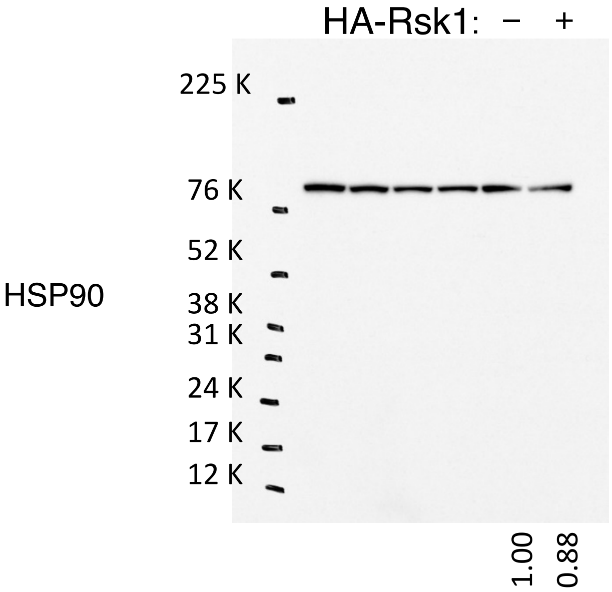
HA-Rsk1: − +
225 K
76 K
52 K
38 K
31 K
24 K
17 K
12 K
HSP90
1.00
0.88

Figure 5G

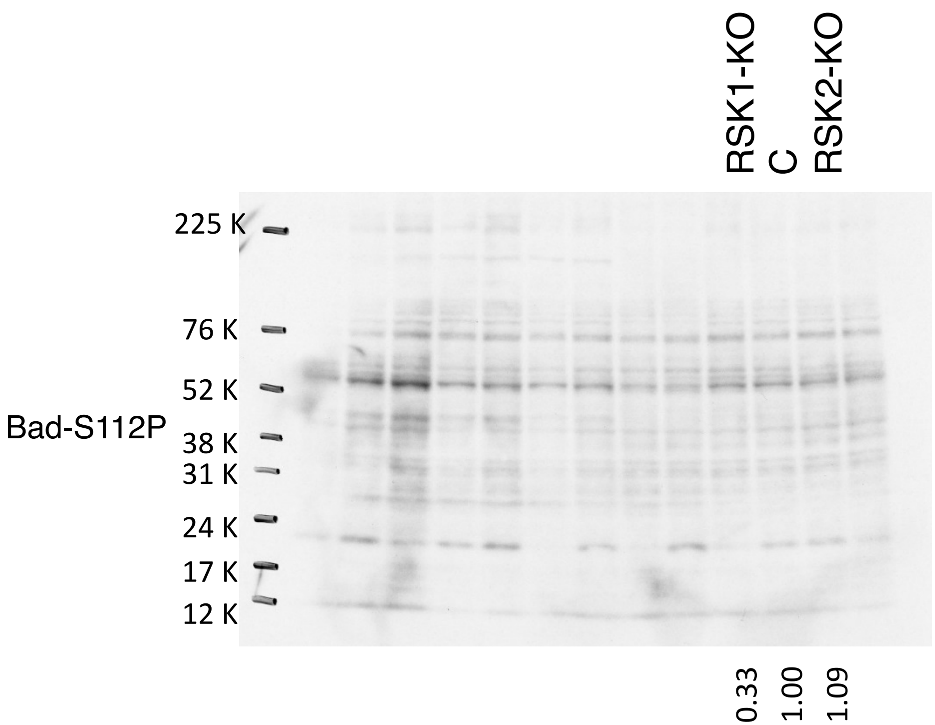

RSK1-KO
C
RSK2-KO
225 K
76 K
52 K
Bad-S112P
38 K
31 K
24 K
17 K
12 K
0.33
1.00
1.09

Figure 5G

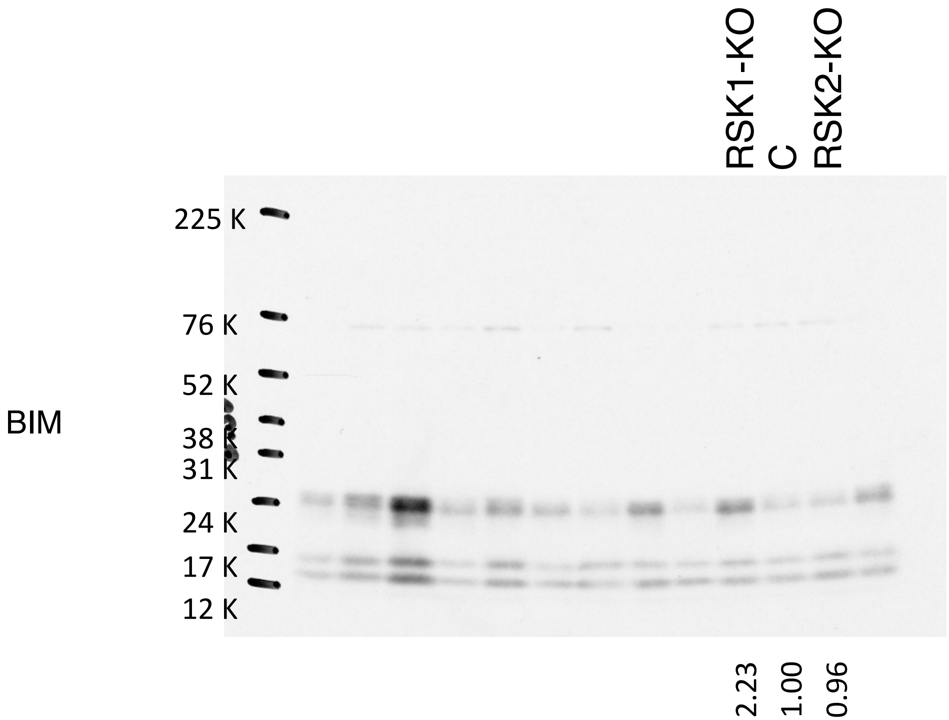
RSK1-KO
C
RSK2-KO
BIM
225 K
76 K
52 K
38 K
31 K
24 K
17 K
12 K
2.23
1.00
0.96

Figure 5G

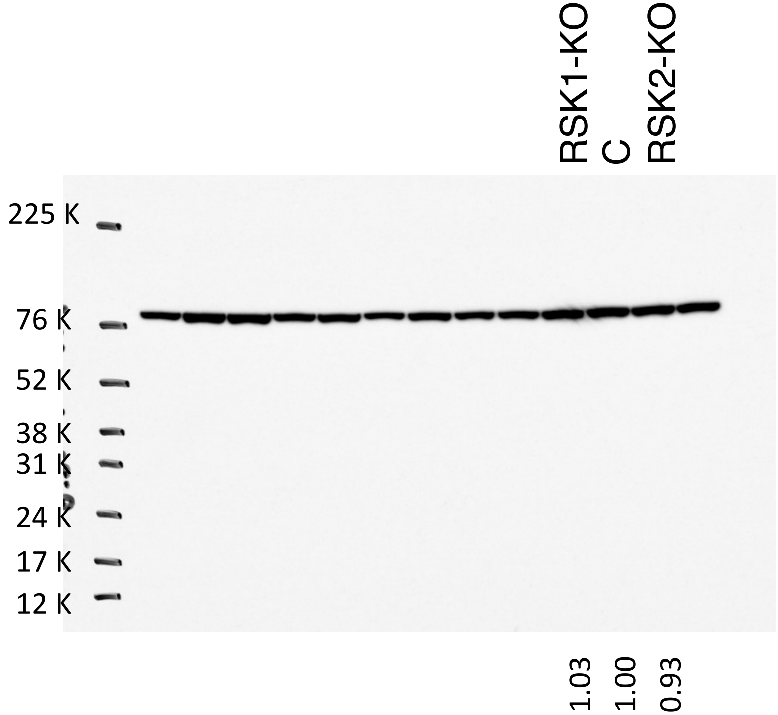

HSP90

Figure 5H

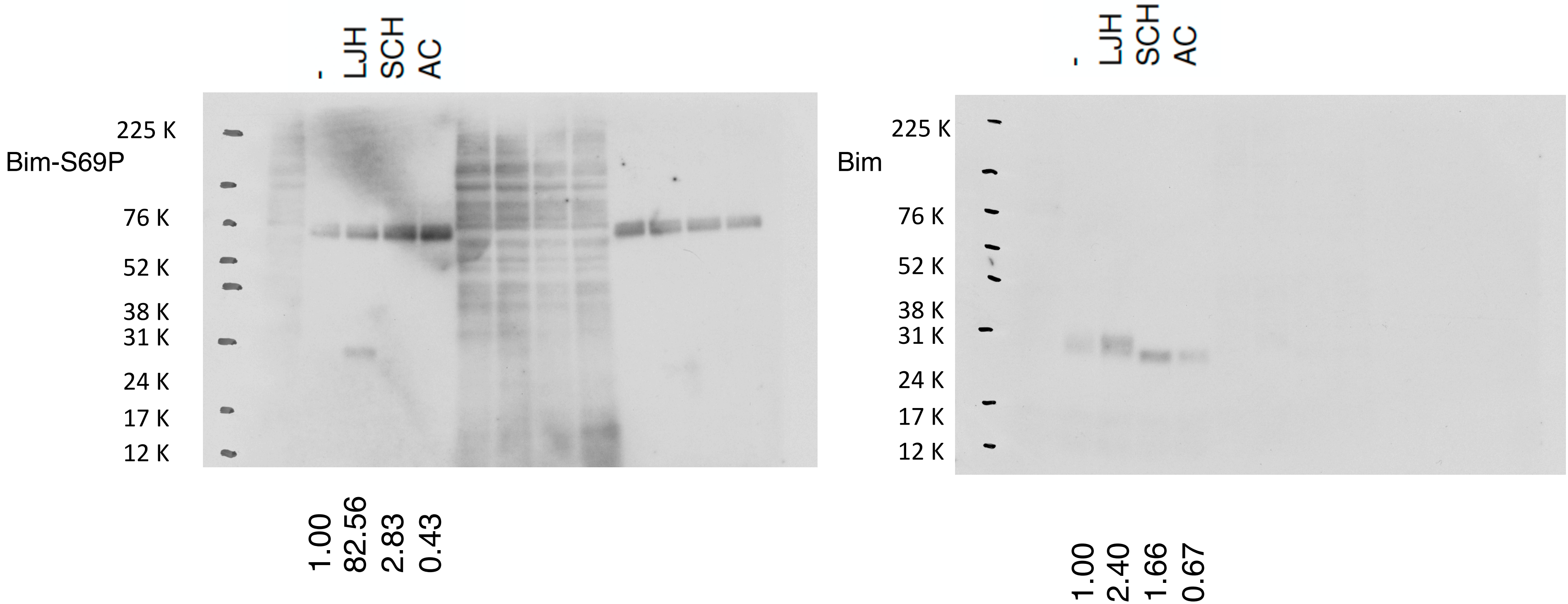

-
LJH
SCH
AC
225 K
Bim-S69P
76 K
52 K
38 K
31 K
24 K
17 K
12 K
1.00
82.56
2.83
0.43
-
LJH
SCH
AC
225 K
Bim
76 K
52 K
38 K
31 K
24 K
17 K
12 K
1.00
2.40
1.66
0.67

Figure 5I

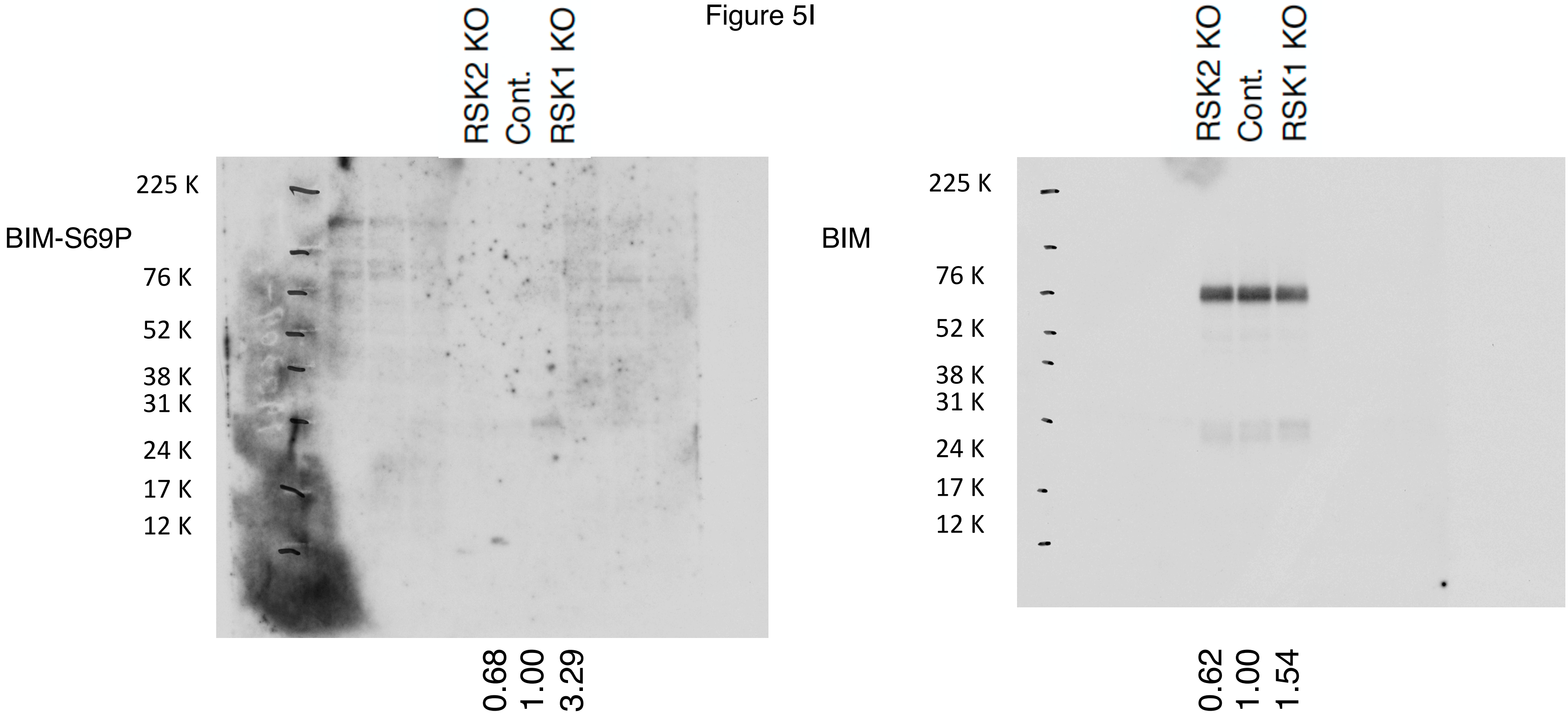
RSK2 KO
Cont.
RSK1 KO
225 K
BIM-S69P
76 K
52 K
38 K
31 K
24 K
17 K
12 K
0.68
1.00
3.29
RSK2 KO
Cont.
RSK1 KO
225 K
BIM
76 K
52 K
38 K
31 K
24 K
17 K
12 K
0.62
1.00
1.54

Figure 5J

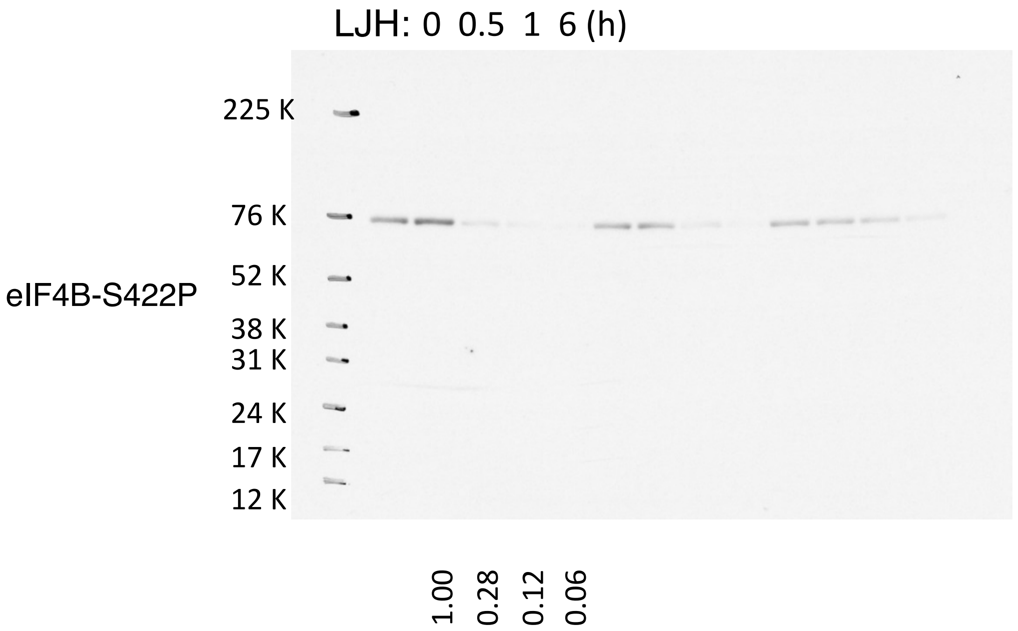
LJH: 0 0.5 1 6 (h)
225 K
76 K
52 K
38 K
31 K
24 K
17 K
12 K
eIF4B-S422P
1.00
0.28
0.12
0.06

Figure 5J

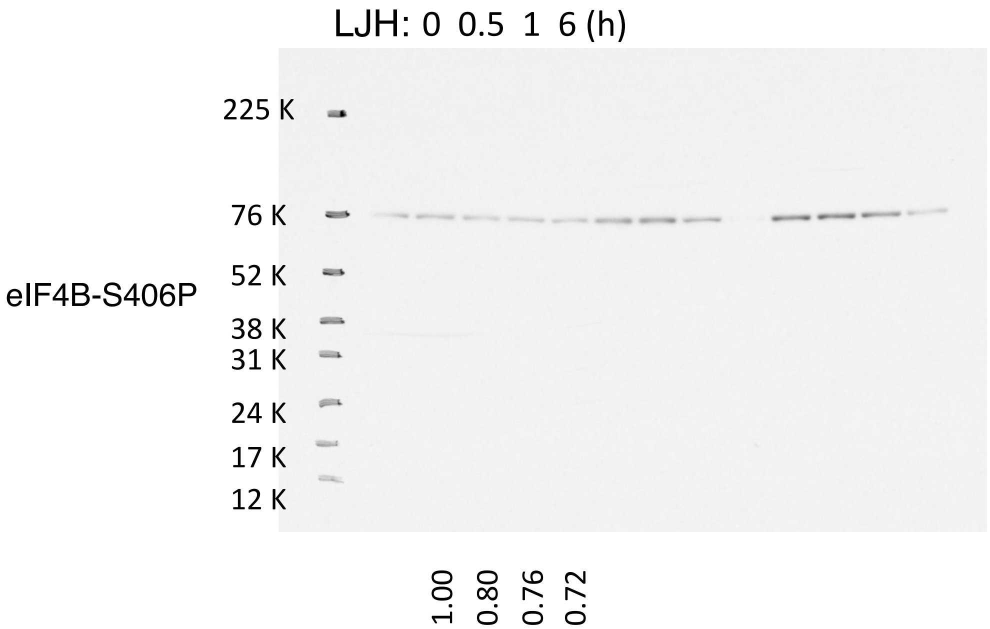
LJH: 0 0.5 1 6 (h)
225 K
76 K
52 K
38 K
31 K
24 K
17 K
12 K
eIF4B-S406P
1.00
0.80
0.76
0.72

Figure 5J

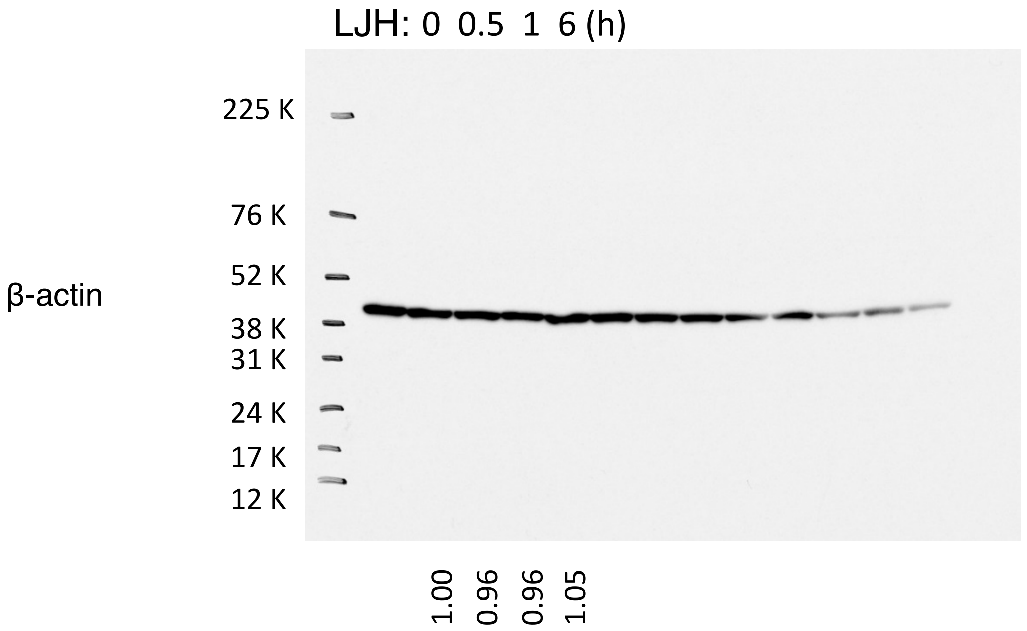
LJH: 0 0.5 1 6 (h)
β-actin
225 K
76 K
52 K
38 K
31 K
24 K
17 K
12 K
1.00
0.96
0.96
1.05

Figure 5K

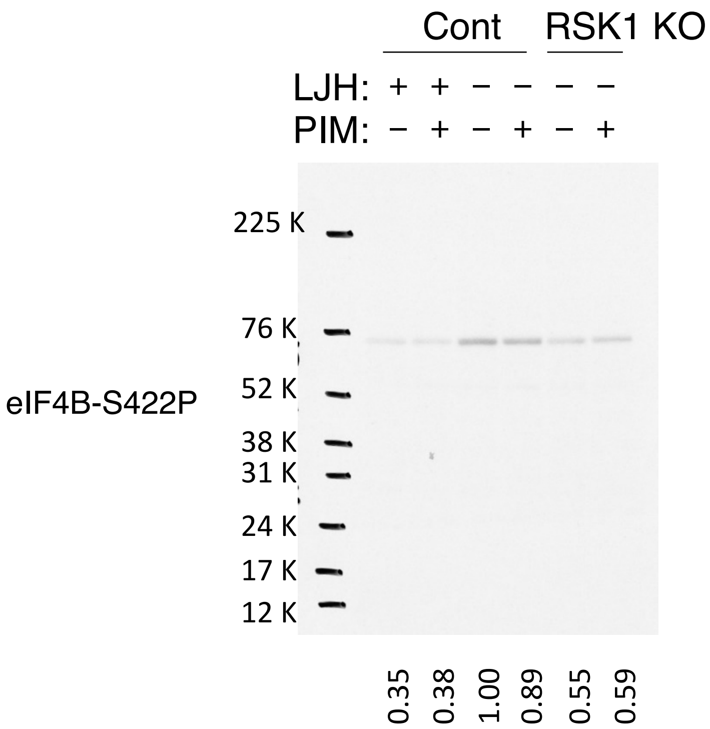

Cont
RSK1 KO
LJH: + + − − − −
PIM: − + − + − +
225 K
76 K
52 K
38 K
31 K
24 K
17 K
12 K
eIF4B-S422P
0.35 0.38 1.00 0.89 0.55 0.59

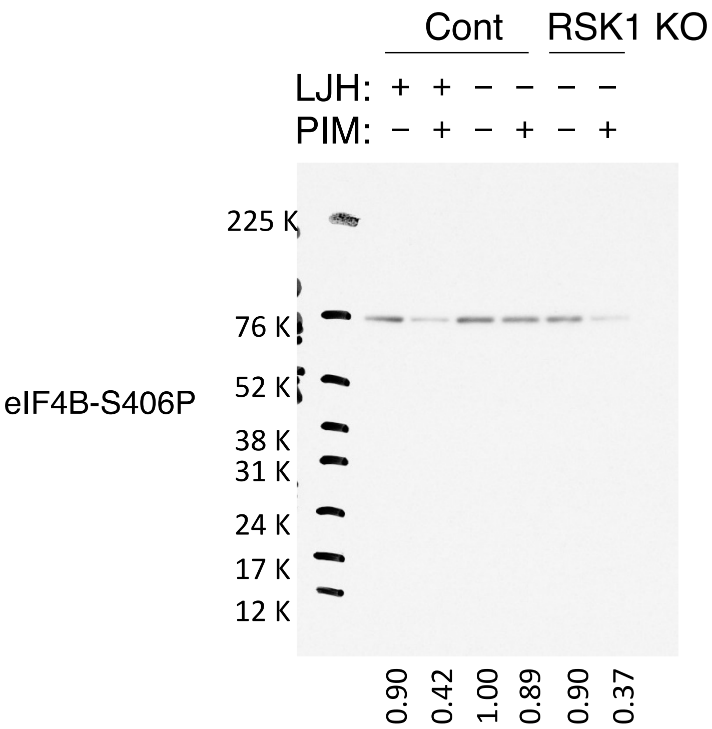

Cont
RSK1 KO
LJH: + + − − − −
PIM: − + − + − +
225 K
76 K
52 K
38 K
31 K
24 K
17 K
12 K
eIF4B-S406P
0.90 0.42 1.00 0.89 0.90 0.37

Figure 5K

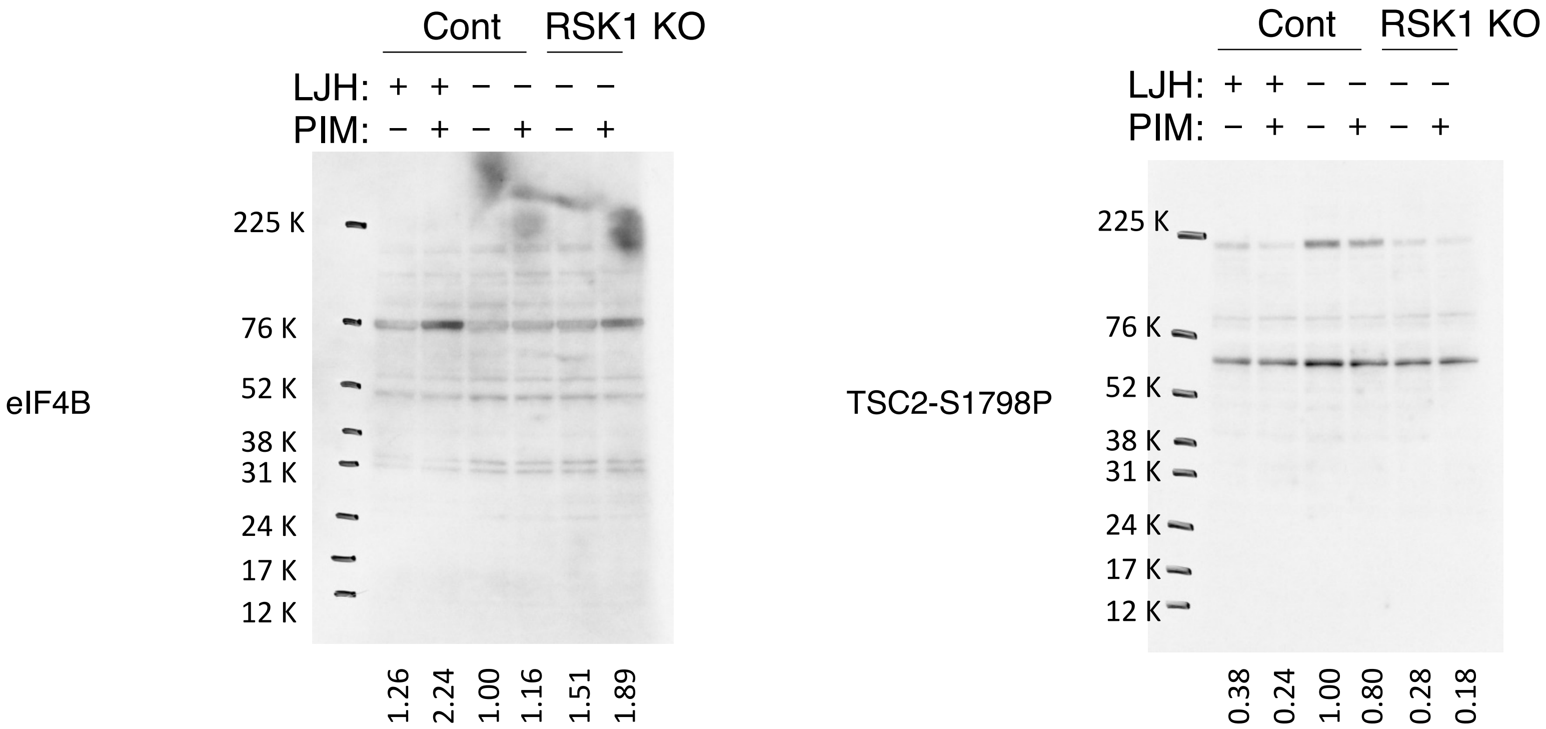

Cont
RSK1 KO
LJH: + + − − − −
PIM: − + − + − +
225 K
76 K
52 K
38 K
31 K
24 K
17 K
12 K
eIF4B
1.26
2.24
1.00
1.16
1.51
1.89
Cont
RSK1 KO
LJH: + + − − − −
PIM: − + − + − +
225 K
76 K
52 K
38 K
31 K
24 K
17 K
12 K
TSC2-S1798P
0.38
0.24
1.00
0.80
0.28
0.18

Figure 5K

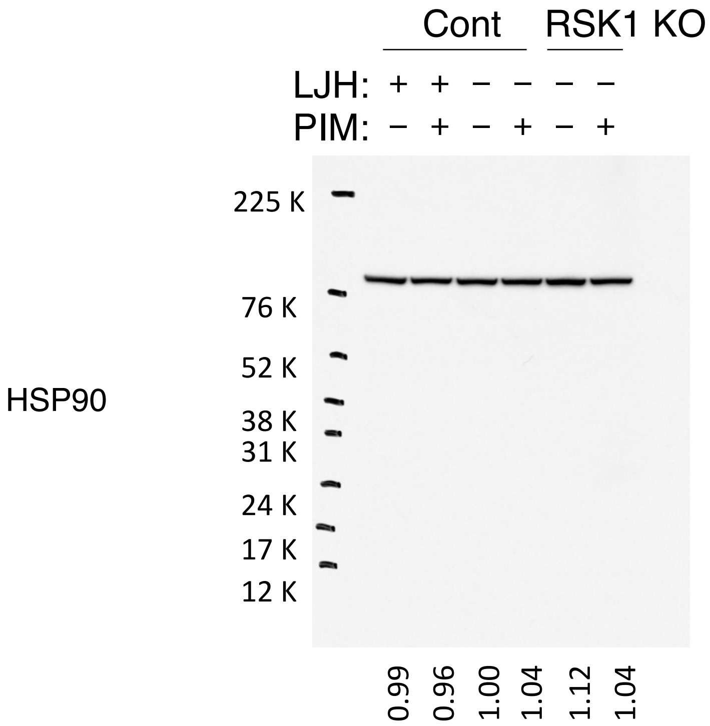
Cont
RSK1 KO
LJH: + + − − − −
PIM: − + − + − +
225 K
76 K
52 K
38 K
31 K
24 K
17 K
12 K
HSP90
0.99
0.96
1.00
1.04
1.12
1.04

Figure 5L

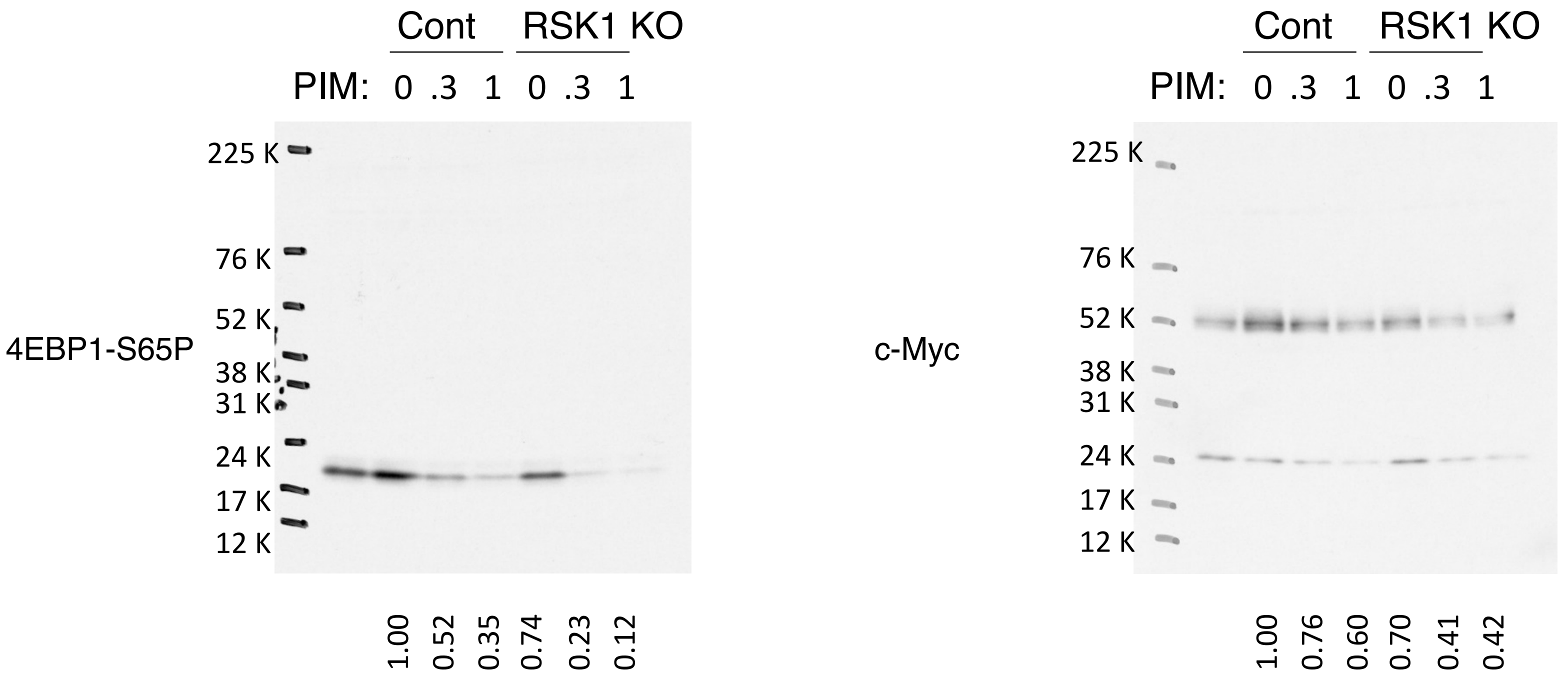

Cont
RSK1 KO
PIM: 0 .3 1 0 .3 1
225 K
76 K
52 K
38 K
31 K
24 K
17 K
12 K
4EBP1-S65P
1.00 0.52 0.35 0.74 0.23 0.12
Cont
RSK1 KO
PIM: 0 .3 1 0 .3 1
225 K
76 K
52 K
38 K
31 K
24 K
17 K
12 K
c-Myc
1.00 0.76 0.60 0.70 0.41 0.42

Figure 5L

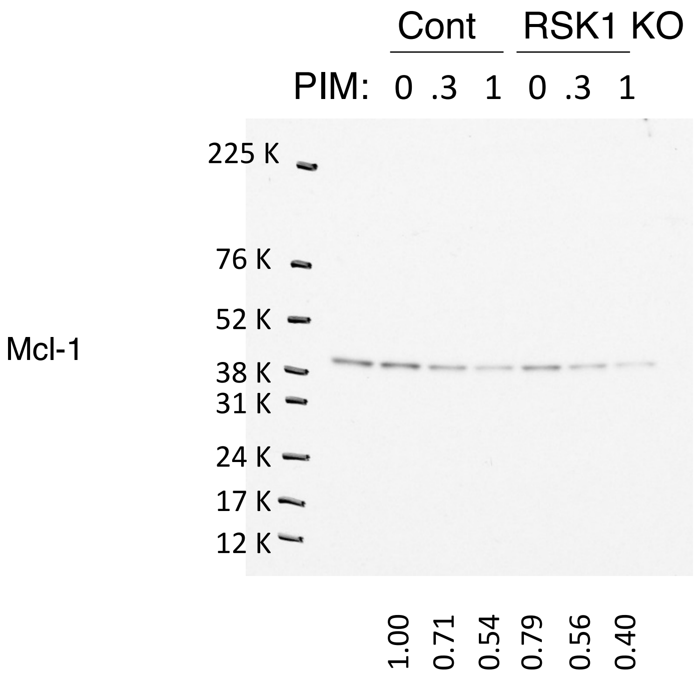
Cont
RSK1 KO
PIM: 0 .3 1 0 .3 1
225 K
76 K
52 K
38 K
31 K
24 K
17 K
12 K
Mcl-1
1.00 0.71 0.54 0.79 0.56 0.40

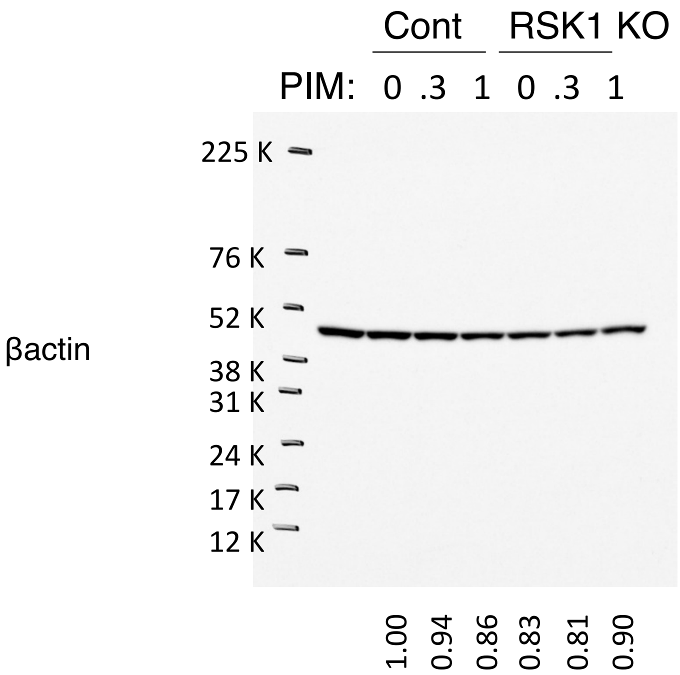
Cont
RSK1 KO
PIM: 0 .3 1 0 .3 1
225 K
76 K
52 K
38 K
31 K
24 K
17 K
12 K
βactin
1.00 0.94 0.86 0.83 0.81 0.90

Figure 6A

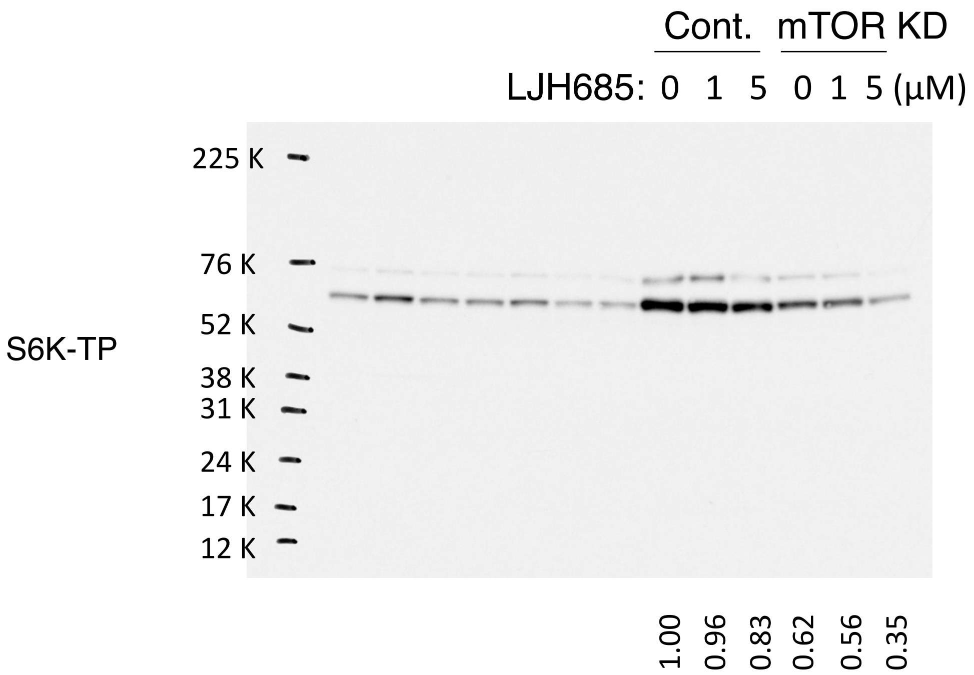
Cont.
mTOR KD
LJH685: 0 1 5 0 1 5 (μM)
225 K
76 K
52 K
38 K
31 K
24 K
17 K
12 K
S6K-TP
1.00
0.96
0.83
0.62
0.56
0.35

Figure 6A

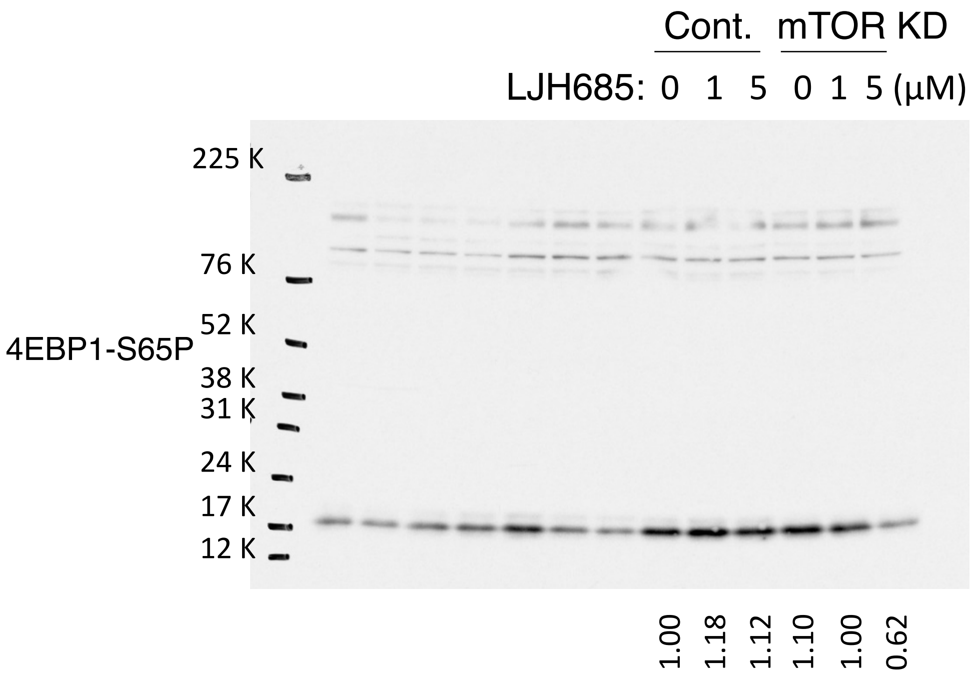
Cont.
mTOR KD
LJH685: 0 1 5 0 1 5 (μM)
225 K
76 K
52 K
4EBP1-S65P
38 K
31 K
24 K
17 K
12 K
1.00
1.18
1.12
1.10
1.00
0.62

Figure 6A

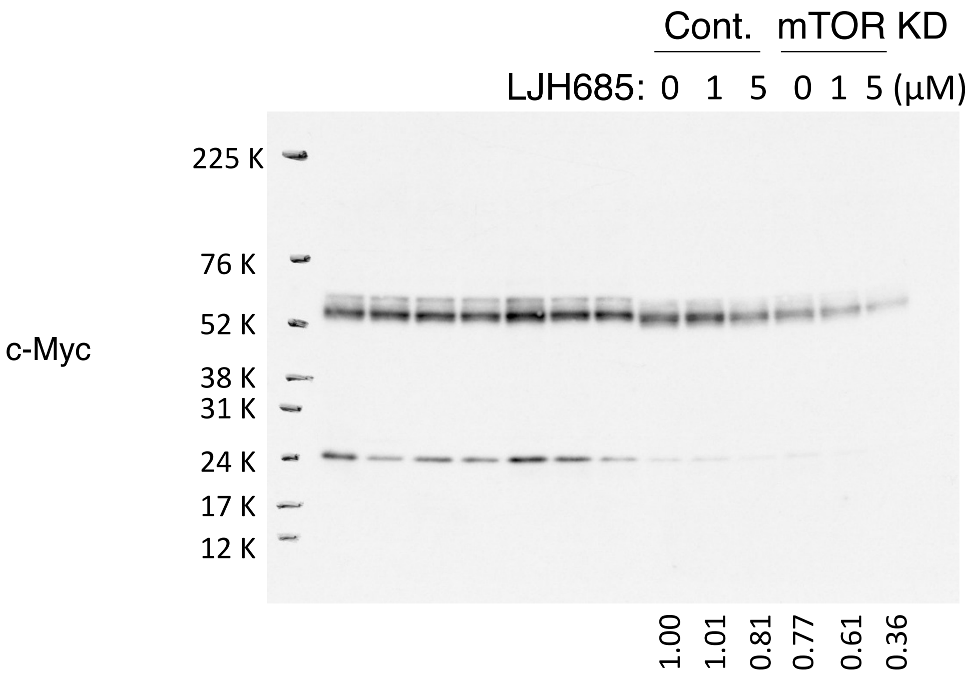

Cont.
mTOR KD
LJH685: 0 1 5 0 1 5 (μM)
225 K
76 K
52 K
38 K
31 K
24 K
17 K
12 K
c-Myc
1.00
1.01
0.81
0.77
0.61
0.36

Figure 6A

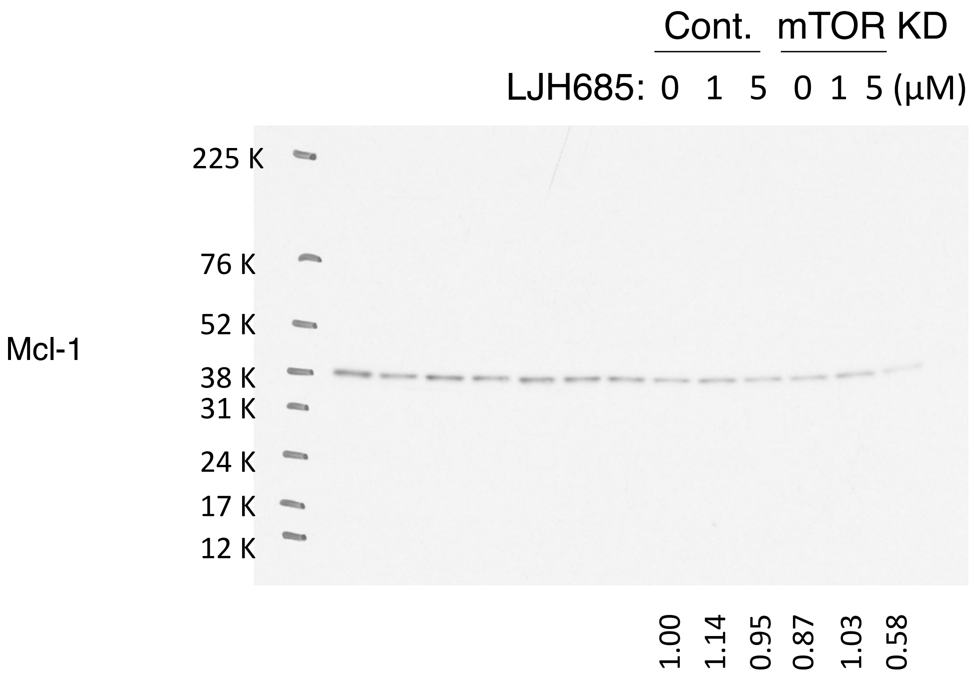
Cont.
mTOR KD
LJH685: 0 1 5 0 1 5 (μM)
225 K
76 K
52 K
Mcl-1
38 K
31 K
24 K
17 K
12 K
1.00
1.14
0.95
0.87
1.03
0.58

Figure 6A

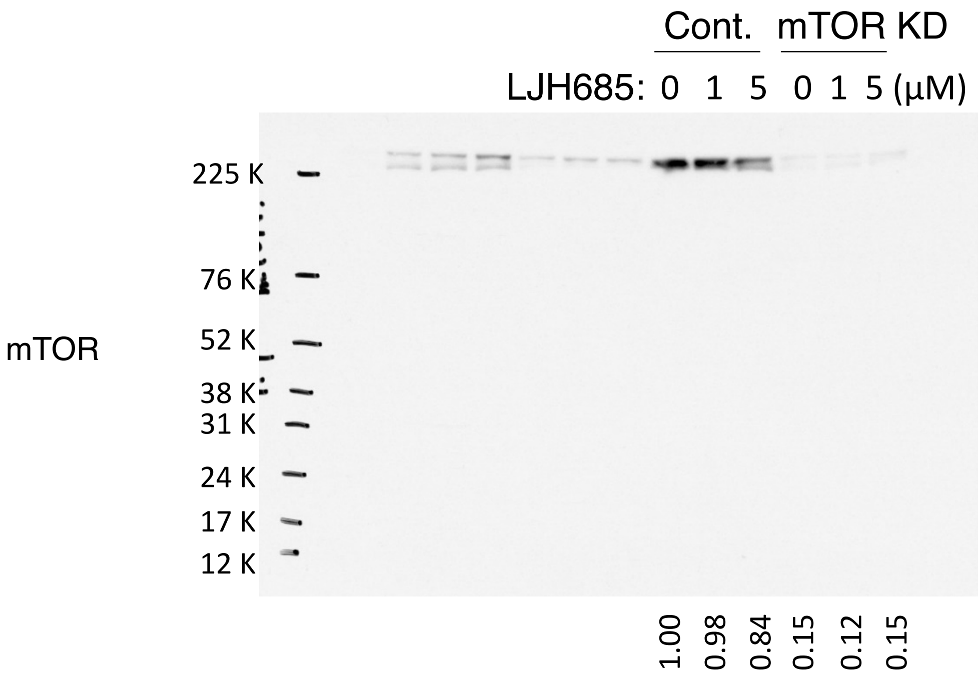

Cont.
mTOR KD
LJH685: 0 1 5 0 1 5 (µM)
225 K
76 K
52 K
38 K
31 K
24 K
17 K
12 K
mTOR
1.00
0.98
0.84
0.15
0.12
0.15

Figure 6A

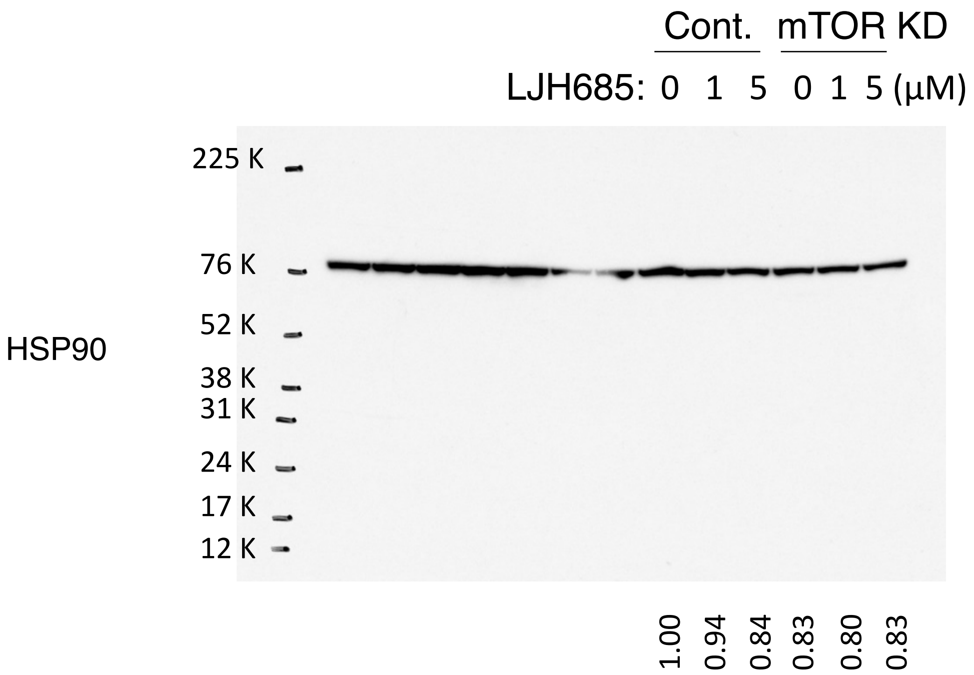
Cont.
mTOR KD
LJH685: 0 1 5 0 1 5 (μM)
225 K
76 K
52 K
HSP90
38 K
31 K
24 K
17 K
12 K
1.00
0.94
0.84
0.83
0.80
0.83

Figure 6I

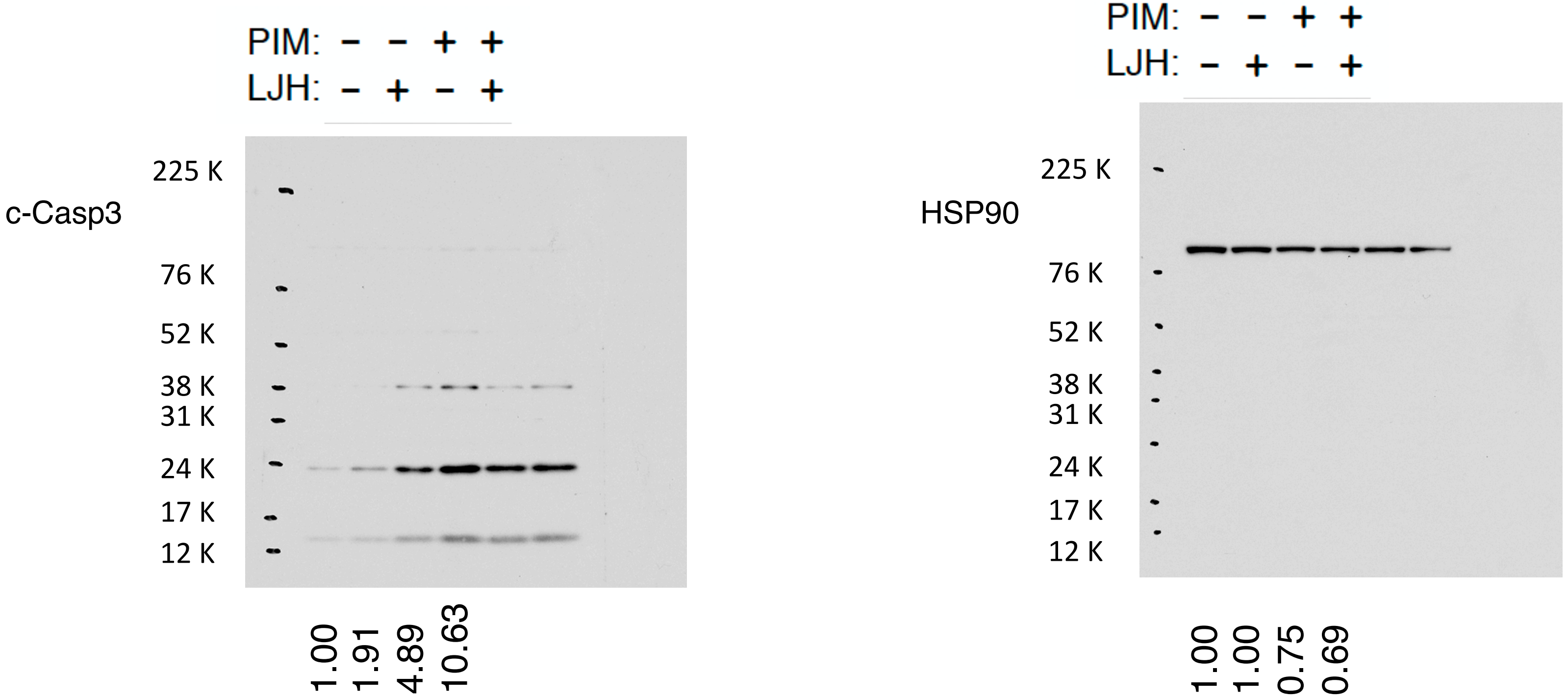
PIM: − − + +
LJH: − + − +
c-Casp3
225 K
76 K
52 K
38 K
31 K
24 K
17 K
12 K
1.00
1.91
4.89
10.63
PIM: − − + +
LJH: − + − +
HSP90
225 K
76 K
52 K
38 K
31 K
24 K
17 K
12 K
1.00
1.00
0.75
0.69
